# Design, Synthesis, and Biological Evaluation of Novel Vinyl Selenone Derivatives as Potent Nrf2 Activators for Atopic Dermatitis

**DOI:** 10.1021/acs.jmedchem.5c02838

**Published:** 2026-03-10

**Authors:** Jushin Kim, Yoowon Kim, Byungeun Kim, Elijah Hwejin Lee, Rium Kim, Jiwoo Park, Jaehwan Kim, Yonghan Kim, Sang In Park, Minsik Kang, Jaeick Lee, Hyeon Jeong Kim, Jong-Hyun Park, Ji Won Choi, Dong-Woo Lee, Ki Duk Park

**Affiliations:** † Center for Brain Disorders, Brain Science Institute, 58975 Korea Institute of Science & Technology (KIST), Seoul 02792, Republic of Korea; ‡ Department of Biotechnology, College of Life Science and Biotechnology, 26721 Yonsei University, Seoul 03722, Republic of Korea; § Division of Bio-Medical Science & Technology, KIST School, University of Science and Technology, Seoul 02792, Republic of Korea; ∥ Doping Control Center, KIST, Seoul 02792, Republic of Korea

## Abstract

Atopic dermatitis (AD) is an inflammatory skin disease characterized by barrier dysfunction, immune dysregulation, and elevated oxidative stress. Since oxidative stress and inflammation are central to AD pathogenesis, activation of the Keap1-Nrf2 pathway, a regulator of antioxidant and cytoprotective defenses, has emerged as a promising therapeutic strategy for AD. We previously developed vinyl sulfone and sulfoximine compounds as potent Nrf2 activators with antioxidant and anti-inflammatory properties. In this study, we introduced a vinyl selenone core as an isosteric replacement to enhance Nrf2 activation potency. Among the synthesized compounds, **5w** exhibited excellent potency (EC_50_ = 4.9 nM), inducing Nrf2-dependent antioxidant enzymes and suppressing cytokine-driven inflammation in HaCaT keratinocytes. In Raw264.7 macrophages, **5w** attenuated inflammatory, nitrosative, and oxidative stress responses. Therapeutic efficacy was validated in a DNCB-induced AD mouse model, where **5w** alleviated local inflammation and AD-like symptoms. Collectively, these findings highlight **5w** as a novel therapeutic agent for inflammatory skin diseases such as AD.

## Graphical abstract

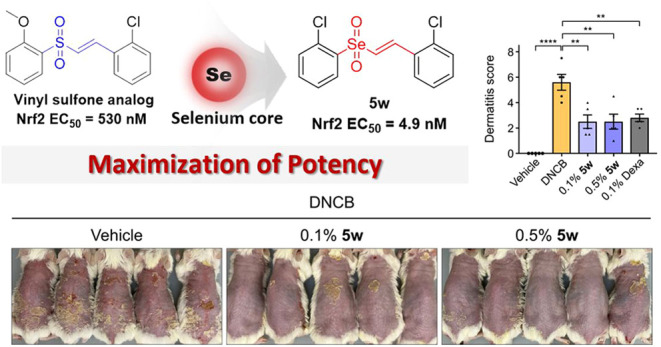

## Introduction

Atopic dermatitis (AD) is a chronic inflammatory skin disorder characterized by erythema, eczema, pruritus, and discomfort, which are closely associated with skin barrier dysfunction and immune dysregulation.^1,2^ In particular, an imbalance between T helper 1 (Th1) and T helper 2 (Th2) immune responses plays a pivotal role in the pathogenesis of AD. When allergens penetrate a compromised skin barrier, immune cells such as T cells, mast cells, and dendritic cells infiltrate the lesional skin, triggering elevated levels of Th2 cytokines and serum immunoglobulin E (IgE).^3–6^ This immune dysregulation underlies the clinical manifestations of AD, which affects up to 20% of children worldwide and occurs or recurs in approximately 3% of adults.^7,8^ In clinical practice, patients with AD are commonly managed with corticosteroids and calcineurin inhibitors. However, corticosteroids are associated with adverse effects such as cutaneous and systemic atrophy and striae, while calcineurin inhibitors may cause local side effects including stinging and irritation.^9–13^ Therefore, the development of novel and safe anti-inflammatory agents remains imperative for the effective treatment of AD.

The involvement of oxidative and inflammatory stress in AD highlights the importance of endogenous cytoprotective defense systems such as the Kelch-like ECH-associated protein (Keap1)-nuclear factor erythroid 2-related factor 2 (Nrf2) pathway.^14,15^ Under basal conditions, Nrf2 is sequestered in the cytoplasm by Keap1, which facilitates its ubiquitination and subsequent proteasomal degradation. However, upon exposure to oxidative or electrophilic stress, Nrf2 dissociates from Keap1 and translocates into the nucleus, where it binds to antioxidant response elements (AREs) and promotes the transcription of various cytoprotective genes encoding antioxidant and phase II detoxifying enzymes. Thus, this pathway plays a crucial role in maintaining cellular homeostasis and protecting tissues from inflammation-mediated damage.^15–17^

Given its pivotal role in regulating cellular responses to oxidative and inflammatory stress, the Keap1-Nrf2 pathway has emerged as a promising therapeutic target for inflammatory skin diseases, including AD. In patients with AD, elevated oxidative stress contributes to skin barrier dysfunction and immune dysregulation, both of which are central features of the disease.^18–20^ In this context, activation of Nrf2 has been shown to enhance antioxidant enzyme expression, attenuate inflammatory signaling,^21,22^ and reinforce epidermal barrier integrity.^23,24^ Hence, pharmacological activation of the Keap1-Nrf2 pathway may offer therapeutic benefit in AD by mitigating oxidative damage and suppressing chronic inflammation. Notably, topical application of Nrf2 activators has been shown to effectively ameliorate AD-like symptoms. In an oxazolone-induced AD mouse model, topical administration of cardamonin significantly decreased Th2 cytokine expression and suppressed epidermal thickening through Nrf2 activation,^25^ while resveratrol attenuated inflammation by modulating the Nrf2 pathway.^26,27^ Moreover, the topical agent tapinarof (Vtama), an activator of aryl hydrocarbon receptor (AhR) and Nrf2 pathways, has been approved by the FDA for the treatment of psoriasis and AD.^28–30^ Together, these findings highlight the Keap1-Nrf2 pathway as a promising therapeutic target in AD.

Selenium-containing compounds have gained increasing attention in medicinal chemistry owing to their unique redox properties, which are closely associated with their antioxidative and anti-inflammatory activities.^31–34^ As a group 16 element in the chalcogen family, selenium shares chemical similarities with sulfur, yet offers distinct electronic and chemical properties that can enhance the biological activity of small molecules.^31–33,35^ In our previous efforts to develop novel Nrf2 activators, we integrated vinyl sulfone and sulfoximine moieties into the chalcone scaffold, resulting in compounds with potent antioxidant and anti-inflammatory activities.^36–41^ Building on this work, we introduced an isosteric vinyl selenone core to exploit its unique electronic properties and redox potential, and synthesized a series of analogs to further enhance Nrf2 activation potency. The synthesized compounds were evaluated for their ability to modulate Keap1-Nrf2 pathway and for their topical therapeutic efficacy, to investigate their potential as a novel and effective treatment strategy for inflammatory skin diseases such as AD.

## Results and Discussion

### Design

Our previous studies have identified vinyl sulfone and subsequent vinyl sulfoximine derivatives of a chalcone scaffold as potent Nrf2 activators,^36–41^ highlighting core optimization as an effective approach to improve the potency and drug-like properties of related analogs. In that regard, selenium emerged as an attractive candidate due to its isosteric relationship with sulfur as a member of the group 16 chalcogen family.^31–33^ Owing to its larger atomic size and greater polarizability, selenium exhibits unique physicochemical properties—such as higher redox potential and increased electrophilicity compared to its sulfur analog—that can be exploited in medicinal chemistry to enhance the biological activity of small molecules.^31–33,35^ The increased lipophilicity and enhanced cell membrane permeability associated with the lower polarity of selenium offer additional advantages for modulating the pharmacological activity of small-molecule drugs.^31,42^ Moreover, numerous natural and synthetic organoselenium compounds have been explored and identified as antioxidant, anti-inflammatory, antitumor, antiviral, antibacterial, and neuroprotective agents,^31,43–46^ suggesting that selenium-containing small molecules represent a promising strategy for the development of therapeutics targeting a broad range of diseases, including AD. In this study, a vinyl selenone core was incorporated along with various functional groups, such as pyridine rings and halogen atoms, to evaluate their potential as Nrf2 activators and to investigate structure–activity relationships (SAR) in comparison to their vinyl sulfone counterparts. We also aimed to provide a novel perspective by systematically assessing the impact of isosteric replacement of the vinyl sulfone core with a vinyl selenone moiety, leveraging the distinct redox potential and electronic properties of selenium to enhance the pharmacological efficacy of the synthesized compounds (Figure 1).

**Figure 1:**
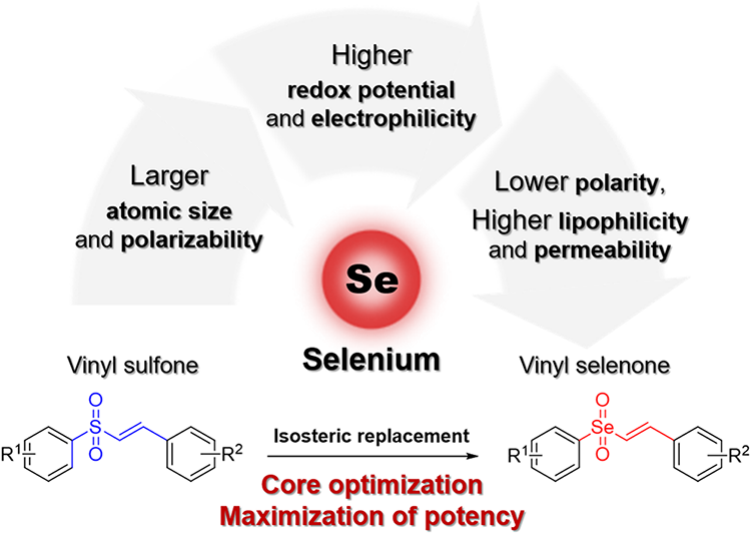
Isosteric replacement of the vinyl sulfone scaffold with a vinyl selenone core to enhance the pharmacological efficacy of the compounds.

### Chemical Synthesis

Based on the structural framework of previously developed vinyl sulfonyl and sulfoximinyl derivatives,^36,37,39–41^ selenium was introduced into the core structure of the synthesized compounds (Scheme 1). Initially, boronic acid derivatives were employed to prepare selenocyanate derivatives **1a**–**1e** through a reaction with selenium powder and trimethylsilyl cyanide (TMSCN). Aryl iodide derivatives were also used to synthesize diaryl diselenides **2a**–**2e** by reacting them with copper­(II) oxide (CuO) and potassium hydroxide (KOH). The resulting selenocyanate and diaryl diselenide intermediates were then reacted with sodium borohydride (NaBH_4_) and diethyl (*p*-toluenesulfonyloxymethyl)­phosphonate to introduce a phosphonate moiety, yielding compounds **3a**–**3k** for use in the Horner–Wadsworth–Emmons (HWE) reaction. The HWE reaction, conducted with *n*-butyllithium (*n*-BuLi) and the corresponding aldehyde derivatives, afforded vinyl selenide intermediates **4a**–**4aa** and **6a**–**6u**. Vinyl selenone derivatives were synthesized via a modified oxidation procedure, differing from previously reported methods.^36–40^ In contrast to the oxidation conditions typically employed for sulfone synthesis, higher temperatures were required for the oxidation of selenium to selenone (Scheme 1). Final compounds **5a**–**5aa** and **7a**–**7u** were obtained through oxidation with *m*-chloroperbenzoic acid (mCPBA) under reflux conditions.

**Scheme 1:**
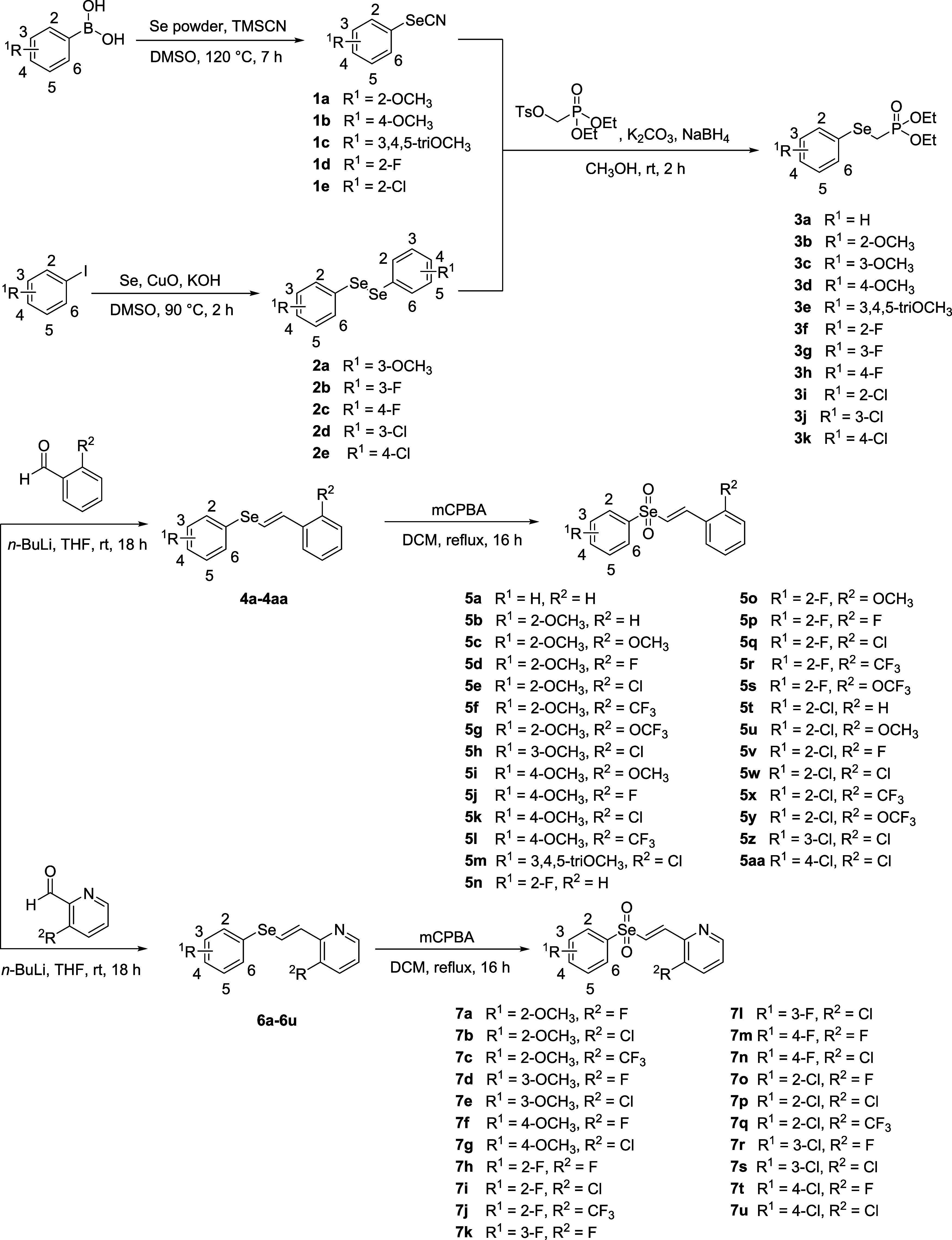
Synthesis of Final Compounds **5a**–**5aa** and **7a**–**7u**

### Structure–Activity Relationship (SAR) Analysis of Vinyl Selenone Derivatives and Physicochemical Profiles of the Selected Compound

In pursuit of novel Nrf2 activators, we previously developed vinyl sulfoxide and vinyl sulfone compounds, and found that among the three scaffolds—vinyl sulfoxide, vinyl sulfone, and the chalcone analog—the vinyl sulfone exhibited the strongest induction of the heme oxygenase-1 (HO-1) gene, a key Nrf2-dependent antioxidative gene, followed by the chalcone, with the vinyl sulfoxide being the least potent.^36^ Consistent with previous findings, vinyl selenoxide derivatives were evaluated but demonstrated no significant Nrf2-activating activity (data not shown). Thus, an initial investigation of the Nrf2 activation potency of the vinyl selenone compounds was mainly conducted using an established cell-based assay system,^37–41^ which assesses their ability to promote the release of Nrf2 from Keap1 and its subsequent translocation into the nucleus. The assay was performed using a commercially available kit, following the manufacturer’s instructions. Half-maximal effective concentration (EC_50_) values were determined to quantify the Nrf2-activating efficacy of all synthesized vinyl selenone compounds (Table 1).

**Table 1:**
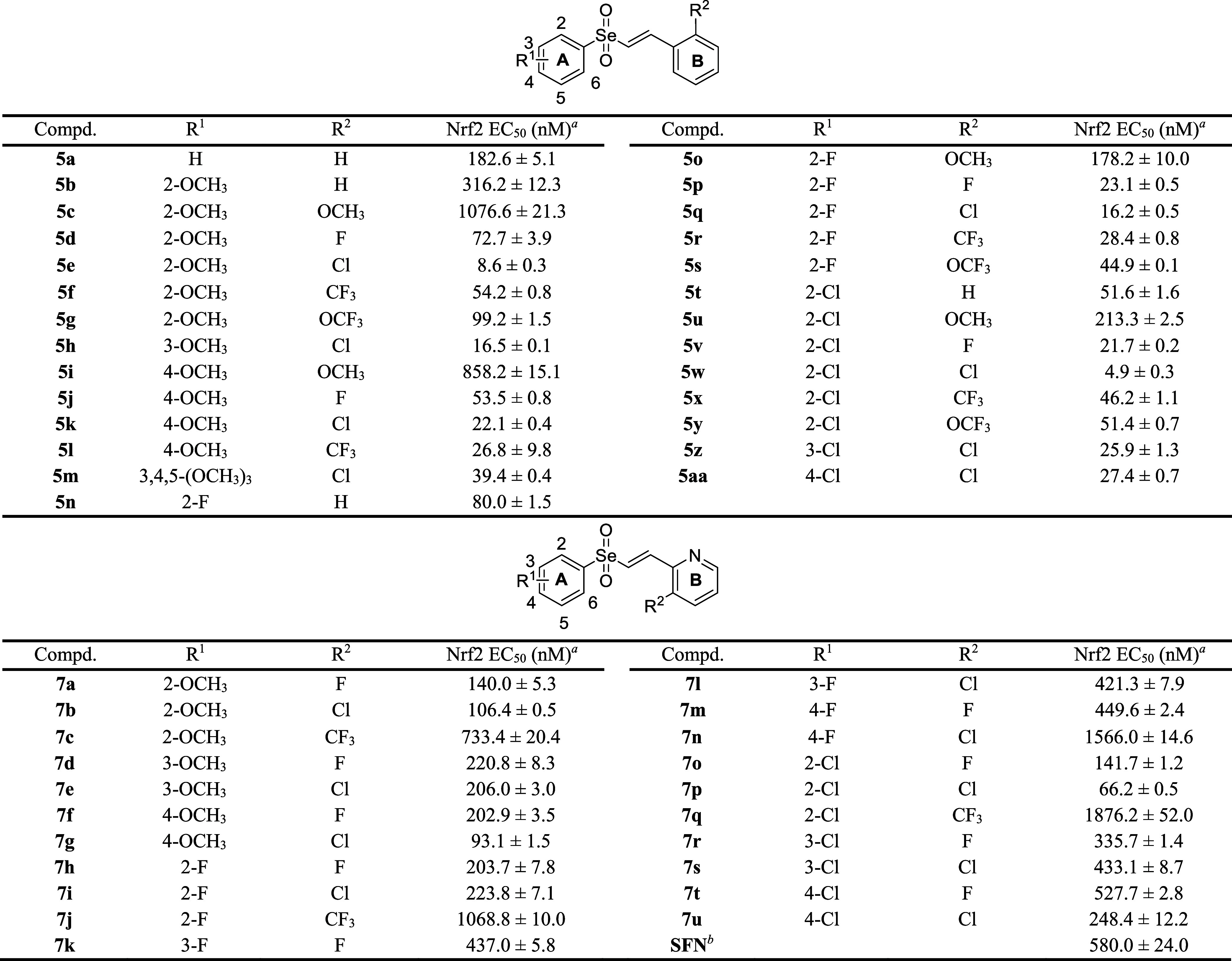
Effects of Synthesized Compounds **5a**–**5aa** and **7a**–**7u** on Nrf2 Activation

a The Keap1-Nrf2 functional assay was performed using a PathHunter U2OS Keap1-Nrf2 nuclear translocation cell line (93–0821C3, DiscoverX). U2OS cells were plated at 13,000 cells/well in triplicate with various compound concentrations for 6 h. The activation-dependent nuclear factor (erythroid-derived 2)-like 2 (Nrf2) translocation was determined using a cell-based functional assay, with mean ± standard error of the mean half-maximal effective concentration (EC_50_) values.

b SFN, sulforaphane, a positive control; EC_50_ value previously reported in ref 37.

Based on previous SAR analyses of vinyl sulfones,^36^ compounds bearing a methoxy group on ring A, in combination with electron-withdrawing substituents (F, Cl, CF_3_, or OCF_3_) on ring B, demonstrated the highest Nrf2-activating potency. Therefore, the corresponding set of vinyl selenone analogs was first synthesized, yielding compounds **5b**–**5l** (Scheme 1, Table 1). Similar to the SAR observed for vinyl sulfone compounds, vinyl selenone analogs with electron-withdrawing groups (F, Cl, CF_3_, or OCF_3_) on ring B exhibited significantly greater potency in Nrf2 activation compared to those with an electron-donating group (OCH_3_), showing a 125-fold difference between the least and most potent compounds (**5c**: EC_50_ = 1076.6 nM vs **5d**: EC_50_ = 72.7 nM, **5e**: EC_50_ = 8.6 nM, **5f**: EC_50_ = 54.2 nM, **5g**: EC_50_ = 99.2 nM). Of the electron-withdrawing groups investigated on ring B, the Cl substituent exerted the greatest influence on Nrf2 activation potency, with the observed trend as follows: Cl > CF_3_ > F > OCF_3_. Introduction of a methoxy group at the *ortho* position on ring A resulted in the most potent Nrf2 activation, with compound **5e** exhibiting single-digit nanomolar potency (**5e**: EC_50_ = 8.6 nM), followed by the *meta*- and *para*-substituted analogs (2-OCH_3_ > 3-OCH_3_ > 4-OCH_3_). This trend was consistent with the SAR analysis of the vinyl sulfone series, highlighting that the position of the electron-donating group on ring A is one of the critical determinants of Nrf2 modulation. In light of core optimization, replacement of the sulfone core with a selenone core led to a significant 62-fold increase in Nrf2 activation potency (corresponding vinyl sulfone analog: EC_50_ = 530 nM vs **5e**: EC_50_ = 8.6 nM; Table 1 and Table S1 in the Supporting Information), proving that the unique chemical properties of selenium, distinct from those of sulfur, can markedly enhance the biological activity of small molecules.

Substitution of the methoxy group on ring A with halogens (F or Cl) resulted in comparable or increased Nrf2 activation potency among the vinyl selenone compounds (Scheme 1, Table 1; **5n**–**5aa**), a finding notably inconsistent with previous SAR analyses of vinyl sulfone analogs, in which halogen substitution led to similar or slightly reduced HO-1 inducing activity. Of particular note, replacement of the methoxy group with a Cl atom on ring A led to nearly a 2-fold increase in Nrf2 activation potency (**5e**: EC_50_ = 8.6 nM vs **5w**: 4.9 nM), while substitution with F maintained comparable activity (**5q**: EC_50_ = 16.2 nM). Further modification of the Cl substituent from the *ortho* to the *meta* and *para* positions on ring A revealed a similar trend in potency, with the 2-Cl being the most potent, followed by the *meta*- and *para*-substituted counterparts (2-Cl > 3-Cl > 4-Cl). Consistent with the aforementioned trend, modifications on ring B with electron-withdrawing groups (F, Cl, CF_3_, or OCF_3_) also resulted in greater potency compared to an electron-donating group (OCH_3_), albeit to a lesser extent (**5u**: EC_50_ = 213.3 nM vs **5v**: EC_50_ = 21.7 nM, **5w**: EC_50_ = 4.9 nM, **5x**: EC_50_ = 46.2 nM, **5y**: EC_50_ = 51.4 nM). Among the electron-withdrawing groups on ring B, Cl conferred the highest potency in Nrf2 activation, yielding compound **5w** which demonstrated the greatest Nrf2-modulating activity with an EC_50_ value of 4.9 nM.

Since insertion of a pyridine into ring B of the vinyl sulfone series resulted in up to a 4-fold increase in Nrf2-activating potency (previously reported vinyl sulfone derivatives: EC_50_ = 530 nM vs EC_50_ = 142 nM; Table S1 in the Supporting Information), the corresponding vinyl selenone analogs bearing a pyridine moiety on ring B were similarly synthesized and evaluated (Scheme 1, Table 1; **7a**–**7u**). Surprisingly, the synthesized compounds with a pyridine ring on ring B revealed substantially reduced potency compared to those with benzene rings on both sides, regardless of the functional groups present on either ring A or B (**7b**: EC_50_ = 106.4 nM, **7i**: EC_50_ = 223.8 nM, **7p**: 66.2 nM). This result directly contrasts not only with the SAR analysis of the vinyl sulfone compounds but also with that of the vinyl sulfoximine series, in which compounds with benzene rings on both sides exhibited only moderate Nrf2 activation potency,^41^ suggesting that vinyl selenone analogs possess a distinct SAR profile compared to other synthesized series of Nrf2-modulating compounds.

Through optimization of the core structure from sulfone and sulfoximine to selenone, combined with SAR analysis of the synthesized vinyl selenone derivatives, we identified compound **5w**—bearing benzene rings substituted with 2-Cl on both sides—as the most potent Nrf2 activator with an EC_50_ value of 4.9 nM. Hence, compound **5w** was selected for further profiling to evaluate its potential as a topical therapeutic agent for AD. The metabolic stability and physicochemical properties of **5w** were examined prior to evaluating its therapeutic efficacy as a topical agent. Microsomal stability studies revealed moderate metabolic stability after a 30 min incubation with human liver microsomes, suggesting limited systemic exposure due to relatively rapid hepatic clearance (Table 2). The calculated partition coefficient (cLog*P*) and topological polar surface area (tPSA) of **5w** were 5.14 and 34.1 Å^2^, respectively, indicating favorable lipophilicity for skin penetration (Table 2). Consistent with these properties, **5w** demonstrated good skin permeability (*P*_e_ = 4.24 × 10^–6^ cm/s) in the skin parallel artificial membrane permeation assay (skin-PAMPA) assay, supporting its feasibility for topical delivery (Table 2). Collectively, these findings highlight the favorable physicochemical profile of **5w** and support its potential as a topical therapeutic agent for AD.

**Table 2:**
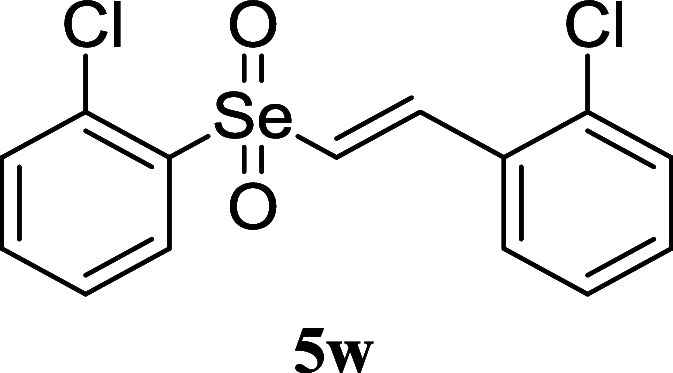
Metabolic Stability and Physicochemical Properties of Compound **5w**

Molecular weight (g/mol)	360.10
Nrf2-activating potency (EC_50_, nM)	4.9 ± 0.3
Microsomal stability (% remaining)^a^	55.7
cLog*P* ^b^	5.14
tPSA (Å^2^)^b^	34.1
Skin-PAMPA (*P*_e_, 10^–6^ cm/s)^c^	4.24 ± 0.15

a *In vitro* microsomal stability of **5w**; % remaining was determined after 30 min incubation with human microsomes. The % of parent compound remaining was calculated by comparing peak areas.

b cLog*P*, calculated partition coefficient; tPSA, topological polar surface area; the predicted values were calculated using ChemDraw v23.1.2.7.

c Skin permeability was assessed using a transwell system by incubating compounds (50 μM) for 5 h and measuring the amount that permeated through the artificial membrane.

### **5w** Activates Nrf2 Nuclear Translocation, and Induces the Expression of Antioxidant Genes

The most potent Nrf2 activator, **5w**, exhibited an EC_50_ of 4.9 nM in the Keap1-Nrf2 nuclear translocation assay (Figure 2A). Upon activation, Nrf2 accumulates in the cytoplasm and translocates into the nucleus. There, it forms heterodimers with small Maf (sMAF) proteins and binds to ARE in the promoter regions of target genes, thereby inducing the expression of cytoprotective genes involved in antioxidant defense, glutathione synthesis, and xenobiotic detoxification.^15–17^ Based on this mechanism, we investigated whether **5w** activates Nrf2 and induces the expression of key antioxidant and detoxification genes in HaCaT keratinocytes. To exclude cytotoxicity as a confounding factor, the cytotoxicity of **5w** was initially evaluated in HaCaT keratinocytes and showed no toxic effects at concentrations up to 1 μM (Figure 2B). We then assessed whether **5w** induces nuclear translocation of Nrf2 by evaluating its subcellular localization in HaCaT keratinocytes. Nrf2 nuclear localization peaked at 3 h following treatment with 0.1 μM of **5w** (4.84 ± 0.38-fold) and gradually decreased thereafter up to 24 h (Figure 2C). Having established the time-dependent activation of Nrf2, we next examined its concentration-dependent nuclear translocation. Under these conditions, nuclear Nrf2 increased in a concentration-dependent manner, with a significant effect already observed at 0.003 μM (3.60 ± 0.11-fold) (Figure 2D). These findings confirmed that **5w** induces Nrf2 nuclear translocation in HaCaT keratinocytes.

**Figure 2:**
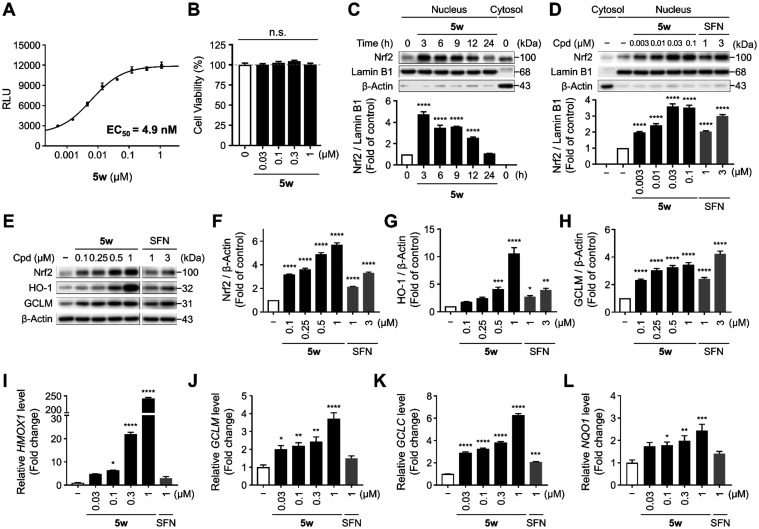
**5w** Induces Nrf2 Activation and Downstream Antioxidant Gene Expression. (A) Concentration–response curve of **5w** for Nrf2 activation in the Keap1-Nrf2 nuclear translocation assay using engineered U2OS cells, with Nrf2 activation quantified as relative light units (RLU) from the translocation signal. (B) Cell viability at 24 h after treatment of **5w** in HaCaT keratinocytes. Western blot analysis of Nrf2 in the nuclear fraction of HaCaT keratinocytes following time-dependent exposure (C) and concentration-dependent exposure for 3 h (D) to **5w**; Relative expression of Nrf2 was normalized to Lamin B1 as a nuclear loading control. (E) Nrf2 accumulation in whole-cell lysates (6 h) and expression of its downstream proteins HO-1 and GCLM (12 h) following **5w** treatment. (F–H) Relative expression of Nrf2, HO-1, and GCLM normalized to β-Actin as an internal control. (I–L) Relative mRNA expression of *HMOX1*, *GCLM*, *GCLC*, and *NQO1* after 6 h treatment with various concentrations of **5w** in HaCaT keratinocytes. All experiments were performed at least twice. Data are presented as mean ± SEM. Statistical significance is indicated as follows: *p < 0.05, **p < 0.01, ***p < 0.001, and ****p < 0.0001, one-way ANOVA with *Dunnett*’s test compared to untreated control.

To further investigate Nrf2 accumulation and potential functional activation by **5w**, HaCaT keratinocytes were treated with increasing concentrations of the compound, and protein levels in whole-cell lysates were analyzed. Sulforaphane (SFN), a naturally occurring and well-established activator of the Nrf2 signaling pathway, was included as a positive control for comparison. As shown in Figure 2E–F, **5w** increased Nrf2 protein accumulation in a concentration-dependent manner, reaching 5.71 ± 0.10-fold at 1 μM. In comparison, SFN induced a 3.31 ± 0.05-fold increase at 3 μM, supporting robust Nrf2 accumulation by **5w** and suggesting its potential for downstream transcriptional activation. We next examined the expression of two representative Nrf2-dependent antioxidant enzymes, HO-1 and glutamate–cysteine ligase modifier subunit (GCLM). HO-1 exerts cytoprotective effects by catalyzing the degradation of pro-oxidant heme into biliverdin, carbon monoxide, and free iron, while GCLM, a component of the glutamate–cysteine ligase (GCL) complex, plays a critical role in glutathione biosynthesis as a rate-limiting enzyme;^47,48^ together, these enzymes constitute key mediators of cellular antioxidative defenses in the Keap1-Nrf2 pathway. Treatment with **5w** for 12 h significantly upregulated the expression of HO-1 and GCLM by 10.58 ± 0.74-fold and 3.44 ± 0.11-fold, respectively (Figure 2E,G,H), thereby substantiating that compound-mediated Nrf2 activation elicits the upregulation of downstream antioxidant defense pathways at the protein level. To further validate transcriptional activation of the Keap1-Nrf2 pathway, we examined a broader panel of cytoprotective genes and confirmed a concentration-dependent increase in *HMOX1* and *GCLM* mRNA expression after 6 h of **5w** treatment, consistent with the protein-level findings (Figure 2I,J). In addition, the mRNA level of glutamate-cysteine ligase catalytic subunit (GCLC), the rate-limiting enzyme for glutathione biosynthesis, and NAD­(P)H oxidoreductase 1 (NQO1), which detoxifies reactive quinones and prevents reactive oxygen species (ROS) generation,^47,48^ were also gradually increased by 6.28 ± 0.13-fold and 2.45 ± 0.28-fold upon 6 h treatment with **5w** (Figure 2K,L). Despite variations in cell models and time points, these findings concur with earlier reports of Nrf2 activation by vinyl sulfone analogs and further emphasize the enhanced potency of the vinyl selenone analog **5w**. The reported vinyl sulfone analog (Table S1 in the Supporting Information) elicited ∼3.5-fold increase in Nrf2 nuclear translocation with maximal induction of total Nrf2-dependent gene expressions at 10 μM,^36^ whereas **5w** achieved comparable or greater Nrf2 activation at submicromolar concentrations, underscoring the superior Nrf2-activating efficacy of the vinyl selenone derivative. Altogether, the results suggest that **5w** activates Nrf2 and promotes its nuclear translocation, thereby inducing the expression of downstream targets in HaCaT keratinocytes.

### **5w** Suppresses Inflammatory Responses in TNF-α+IFN-γ-stimulated HaCaT Keratinocytes

In the pathogenesis of AD, keratinocytes not only serve as structural components of the skin barrier, but also actively contribute to immune regulation and inflammatory processes. Upon exposure to early inflammatory mediators such as tumor necrosis factor-α (TNF-α) and interferon-γ (IFN-γ), keratinocytes release pro-inflammatory cytokines and chemokines including IL-1β, IL-6, CCL17, and CCL22.^4,49–51^ These mediators amplify local inflammation and facilitate the recruitment and activation of CCR4^+^ Th2 lymphocytes, thereby perpetuating the inflammatory cascade in AD lesions.^4,49–51^ Given this central role of keratinocytes in the pathogenesis of AD, we investigated whether **5w** exerts anti-inflammatory effects on the production of pro-inflammatory mediators by modulating keratinocyte responses under cytokine-stimulated inflammatory conditions.

To mimic an AD-relevant inflammatory environment, HaCaT keratinocytes were stimulated with TNF-α and IFN-γ, a combination known to synergistically enhance the transcription of multiple pro-inflammatory mediators via activation of NF-κB and STAT1 pathways.^52,53^ Prior to cytokine stimulation, HaCaT keratinocytes were pretreated for 3 h with **5w** or SFN, a well-established anti-inflammatory agent^23^ used as a positive control. Western blot analysis revealed a significant upregulation of IL-1β following 24 h stimulation with TNF-α and IFN-γ; however, treatment with **5w** suppressed IL-1β protein expression in a concentration-dependent manner and exhibited greater efficacy than SFN (Figure 3A). Similarly, **5w** markedly reduced cytokine-induced IL-6 secretion, as confirmed by enzyme-linked immunosorbent assay (ELISA) (Figure 3B). To gain a comprehensive view of the anti-inflammatory profile of **5w**, we next examined a broader panel of inflammatory mediators at the transcriptional level. CCL17 and CCL22, in particular, are responsible for recruiting Th2 cells, which subsequently secrete IL-4, IL-5, and IL-13, thereby promoting immunoglobulin E (IgE) production, eosinophil infiltration, and chronic allergic inflammation within AD lesions.^4,6^ Notably, qRT-PCR analysis revealed that **5w** attenuated TNF-α+IFN-γ-induced expression of *IL6*, *TNF*, *CCL17*, and *CCL22* mRNA (Figure 3C–F). Compared with the modest activity of 1 μM SFN, **5w** remained robustly effective within the same concentration window, underscoring its superior anti-inflammatory efficacy. Overall, **5w** exhibited broad anti-inflammatory activity by suppressing both Th1-related cytokines and Th2-inducing chemokines in response to TNF-α+IFN-γ stimulation, leading to a simultaneous modulation of multiple inflammatory factors. Together, these findings demonstrate that **5w** blocks key pathogenic mechanisms of AD by regulating keratinocyte-derived inflammatory responses through activation of the Keap1-Nrf2 pathway.

**Figure 3:**
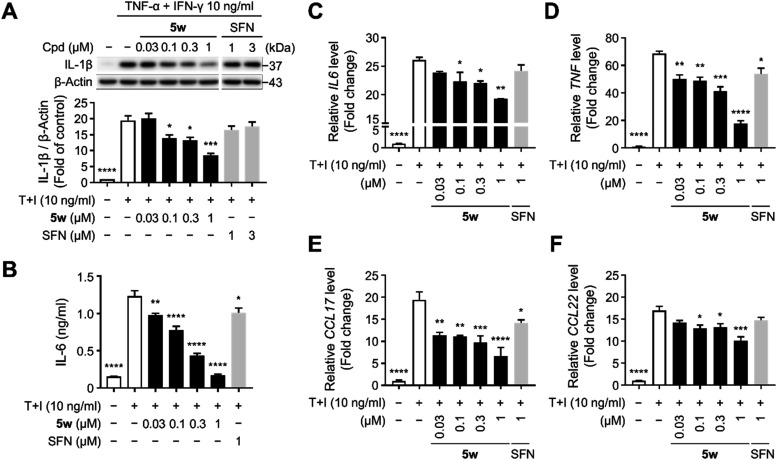
**5w** Suppresses Inflammatory Mediators in TNF-α+IFN-γ-stimulated HaCaT Keratinocytes. (A) Western blot analysis of IL-1β in TNF-α+IFN-γ-stimulated HaCaT keratinocytes. Cells were pretreated with **5w** or SFN for 3 h, followed by stimulation with 10 ng/mL T+I for 24 h. Relative expression of IL-1β was normalized to β-actin. (B) Concentration of secreted IL-6 in culture medium of T+I-stimulated HaCaT cells were measured by ELISA. After a 3 h pretreatment with **5w** or SFN, cells were exposed to 10 ng/mL T+I for 24 h. (C–F) Relative mRNA expression of *IL6*, *TNF*, *CCL17*, and *CCL22* in HaCaT keratinocytes. (C, D) For *IL6* and *TNF*, cells were pretreated with **5w** or SFN for 6 h, followed by T+I stimulation for 3 h. (E, F) For *CCL17* and *CCL22*, cells cotreated with **5w** or SFN and T+I for 24 h. Relative mRNA levels were normalized to *ACTB*. All experiments were performed at least twice. Data are presented as mean ± SEM. Statistical significance is indicated as follows: *p < 0.05, **p < 0.01, ***p < 0.001, and ****p < 0.0001, one-way ANOVA with *Dunnett*’s test compared to T+I-treated control.

### **5w** Alleviates Lipopolysaccharide (LPS)-induced Inflammatory, Nitrosative, and Oxidative Stress Responses in Raw264.7 Macrophages

Having demonstrated the Nrf2-activating and anti-inflammatory properties of **5w** in HaCaT keratinocyte cells, we next sought to validate its efficacy in immune cells that are more directly involved in inflammatory processes. Macrophages are a major population of skin-resident and infiltrating immune cells that play essential roles in both the initiation and perpetuation of inflammation in AD.^4–6^ Activated macrophages contribute to disease progression by producing high levels of pro-inflammatory cytokines, such as IL-1β, IL-6, and TNF-α. Upon activation, macrophages also generate ROS via NADPH oxidase^54^ and reactive nitrogen species (RNS) through inducible nitric oxide synthase (iNOS),^55^ both of which exacerbate tissue inflammation and oxidative damage in AD lesions. To further evaluate the immunomodulatory potential of **5w**, we utilized an LPS-stimulated macrophage model to examine whether it attenuates inflammatory signaling, oxidative stress, and nitrosative stress.

We initially assessed the effect of **5w** on NO production using LPS, a well-established stimulator of macrophages that induces inflammatory responses by promoting iNOS expression and cytokine production via NF-κB activation.^56^ Raw264.7 macrophages were pretreated with increasing concentrations of **5w** or SFN for 3 h, and subsequently exposed to LPS to induce inflammation. 24 h LPS stimulation markedly increased iNOS protein expression in Raw264.7 macrophages, as shown by Western blot analysis, and this upregulation was significantly suppressed by **5w** in a concentration-dependent manner (Figure 4A). Consistent with these results, **5w** also reduced LPS-induced NO production (Figure 4B) and attenuated mRNA expression of *Nos2*, which encodes iNOS, exhibiting greater efficacy than SFN (Figure 4D).

**Figure 4:**
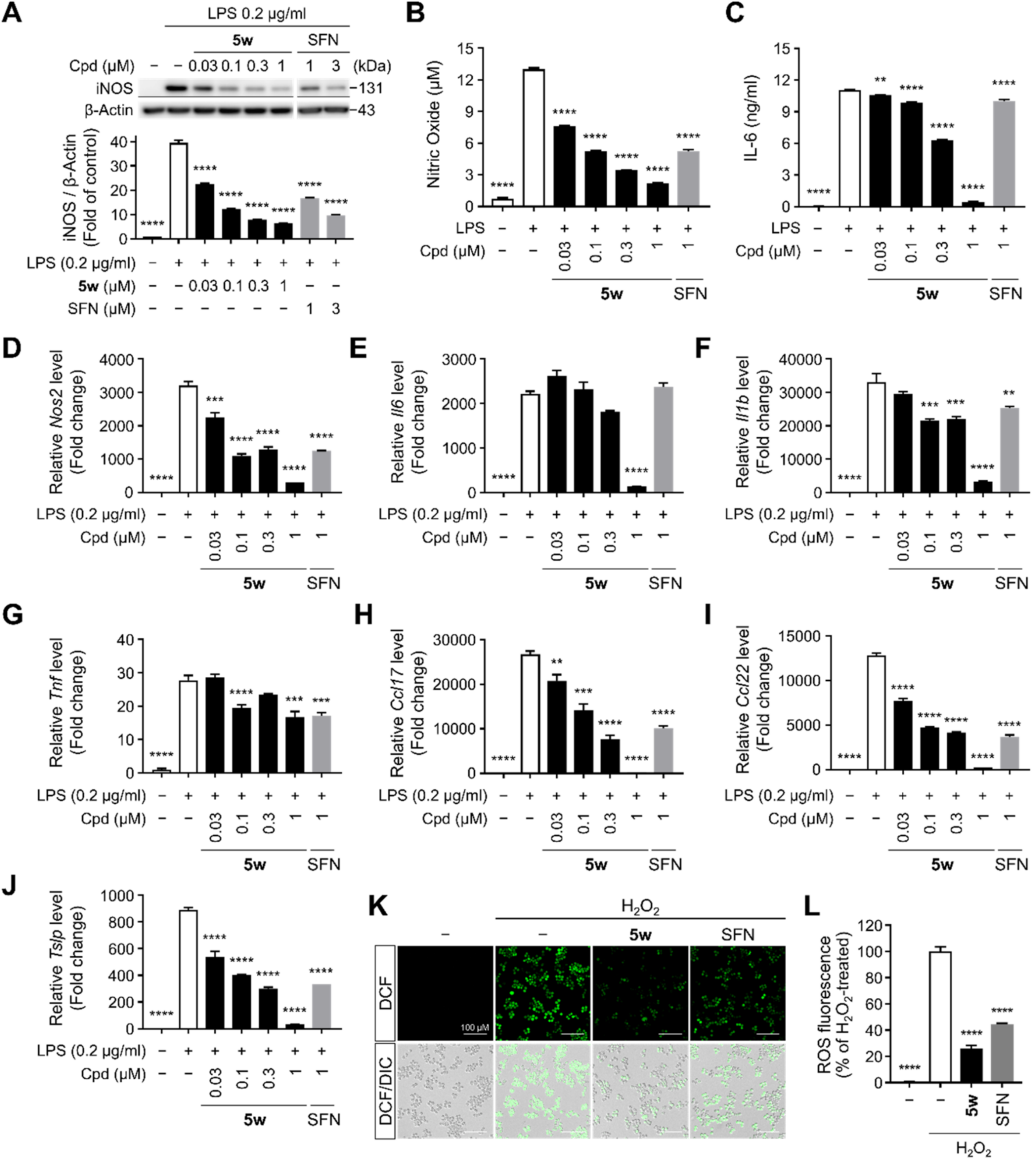
**5w** Attenuates LPS-induced Inflammatory and Redox Stress Responses in Raw264.7 Macrophages. (A) Western blot analysis of iNOS in LPS-stimulated Raw264.7 macrophages after 3 h exposure to **5w** prior to 24 h stimulation. Relative expression of iNOS was normalized to β-actin. (B) Concentration of nitric oxide in the culture medium of LPS-stimulated Raw264.7 cells. Cells were pretreated with **5w** or SFN for 3 h and stimulated with LPS for 24 h. (C) Concentration of IL-6 secreted in culture medium of Raw264.7 cells was measured by ELISA. After 3 h pretreatment with **5w** or SFN, cells were stimulated with LPS for 24 h. (D–J) Relative mRNA expression of inflammatory mediators, *Nos2*, *Il6*, *Il1b*, *Tnf*, *Ccl17*, *Ccl22*, and *Tslp* in Raw264.7 cells. Cells were pretreated with **5w** or SFN for 6 h and subsequently stimulated with LPS for 6 h. Relative mRNA levels were normalized to *Hprt* expression. (K, L) Intracellular ROS levels measured via DCFH-DA staining. Raw264.7 cells were pretreated with 0.1 μM **5w** or 1 μM SFN for 9 h, and subsequently exposed to 300 μM H_2_O_2_ for 20 min. (K) Representative fluorescence microscopy images of DCFH-DA-stained Raw264.7 cells (DCF, intracellular ROS) with corresponding DIC images. (L) Quantitative analysis of DCF fluorescence intensity from (K). All experiments were performed at least twice. Data are presented as mean ± SEM. Statistical significance is indicated as follows: *p < 0.05, **p < 0.01, ***p < 0.001, and ****p < 0.0001, one-way ANOVA with *Dunnett*’s test compared to LPS-treated control.

The anti-inflammatory effects of **5w** were further investigated by measuring the expression of pro-inflammatory cytokines in LPS-stimulated macrophages. Similar to the findings in HaCaT keratinocytes, ELISA results demonstrated that IL-6 secretion induced by 24 h LPS stimulation was remarkably reduced by **5w** in a concentration-dependent manner following 3 h pretreatment, with almost complete suppression observed at 1 μM, comparable to the untreated control (Figure 4C). To further assess the anti-inflammatory activity of **5w** at the transcriptional level, we examined the mRNA expression of relevant cytokines and chemokines. The mRNA expression of pro-inflammatory cytokines, including *Il6*, *Il1b*, and *Tnf*, was markedly elevated upon LPS stimulation and significantly attenuated by **5w** in a concentration-dependent manner (Figure 4E–G). Additionally, the Th2-associated chemokines *Ccl17* and *Ccl22*, previously shown to be suppressed by **5w** in keratinocytes, were likewise downregulated in macrophages (Figure 4H–I). We further evaluated the expression of thymic stromal lymphopoietin (TSLP), an epithelial-derived cytokine critical for dendritic cell activation and Th2 cell polarization.^57^ Although TSLP is predominantly produced by epithelial cells, recent evidence suggest that macrophages may also express TSLP under inflammatory conditions in certain contexts, potentially contributing to the pathogenesis of AD.^57,58^ Notably, **5w** effectively reduced the mRNA expression of *Tslp* in LPS-stimulated macrophages, highlighting its potential to modulate immunological circuits underlying AD pathogenesis (Figure 4J). These results demonstrate that **5w** broadly attenuates AD-related inflammatory responses in Raw264.7 macrophages by downregulating key cytokines and chemokines.

Excessive ROS production not only exacerbates epidermal damage but also amplifies inflammatory signaling, thereby playing a pathogenic role in chronic AD lesions.^54^ To address this, we assessed the antioxidant capacity of **5w** by measuring its ability to reduce intracellular ROS levels in Raw264.7 macrophages subjected to hydrogen peroxide (H_2_O_2_)-induced oxidative stress. As shown in Figure 4K–L, intracellular ROS levels were markedly elevated upon 20 min of H_2_O_2_ stimulation in Raw264.7 macrophages compared with untreated controls. However, pretreatment with 0.1 μM **5w** for 9 h led to a more pronounced reduction in ROS than 1 μM SFN, underscoring its capacity to mitigate oxidative stress implicated in AD pathology.

Together, these data suggest that **5w** acts on both epithelial and innate immune compartments, thereby potentially modulating the keratinocyte-macrophage feed-forward loop that sustains AD inflammation. By suppressing T+I-induced cytokines and Th2-recruiting chemokines in keratinocytes, and attenuating LPS-evoked RNS, ROS, and pro-inflammatory cytokines in macrophages, **5w** may collectively blunt the local initiation and the myeloid amplification of inflammation, thereby enhancing Nrf2-driven cytoprotective defenses.

### **5w** Alleviates AD-like Symptoms by Suppressing Local Inflammation, Thereby Mitigating Systemic Immune Response in DNCB-induced AD-like Conditions

Having confirmed that **5w** activates the Keap1-Nrf2 pathway and exerts anti-inflammatory and antioxidant effects in keratinocytes and macrophages, we sought to determine whether these effects translate into a disease-relevant *in vivo* context. The 2,4-dinitrochlorobenzene (DNCB)-induced mouse model of AD is a widely used system that recapitulates key pathological features of the disease—including barrier dysfunction, inflammatory cell infiltration, Th2-biased immune responses, and IgE elevation—providing a physiologically relevant platform to evaluate the therapeutic potential of **5w** under conditions that mimic human disease.^59–62^ As a chemical allergen, DNCB initiates immune sensitization and subsequently boosts the cutaneous inflammation upon re-exposure. During the sensitization phase, antigen-presenting cells and T lymphocytes are primed, and repeated challenge amplifies a Th2-skewed immune response, collectively reproducing the immunopathological hallmarks of AD.^63^ Accordingly, in this study, **5w** was administered during the challenge phase, concurrently with repeated DNCB application, to evaluate its therapeutic efficacy after AD-like symptoms had been established.

As depicted in the experimental scheme (Figure 5A), BALB/c mice were sensitized and repeatedly challenged with DNCB to induce AD-like symptoms, followed by topical administration with **5w** (0.1 or 0.5%) or dexamethasone as a glucocorticoid positive control.^2,7,9,64^ Although a slight reduction was observed in DNCB-treated groups, changes in body weight across all groups were minimal and not statistically significant, indicating that treatment had no apparent toxicity or impact on general health status during the study (Figure 5B). To evaluate disease severity, clinical dermatitis scores were assessed at the end of the experiment using an established scoring system with slight modifications.^7,64–66^ As shown in Figure 5C, mice treated with DNCB exhibited a marked increase in dermatitis severity compared with the vehicle group (dermatitis score: 5.75 ± 0.61 vs 0.0 ± 0.0, *p* < 0.001), whereas topical administration of **5w** significantly alleviated these symptoms (dermatitis score: 2.75 ± 0.50 in the 0.1% group and 2.83 ± 0.59 in the 0.5% group), with efficacy comparable to dexamethasone. Both 0.1 and 0.5% formulations significantly reduced dermatitis scores, indicating that even the lower dose achieved a therapeutic threshold. These observations were visually supported by representative skin images (Figure 5D), in which **5w**-treated mice exhibited visibly less severe lesions than the DNCB group. In line with the reduction in dermatitis scores, **5w** also suppressed scratching behavior, reflecting mitigation of pruritus, a defining symptom of AD (Figure 5E). This effect may not only reflect general anti-inflammatory activity but also attenuation of the TSLP/IL-31–sensory neuron axis, although this possibility remains to be tested.

**Figure 5:**
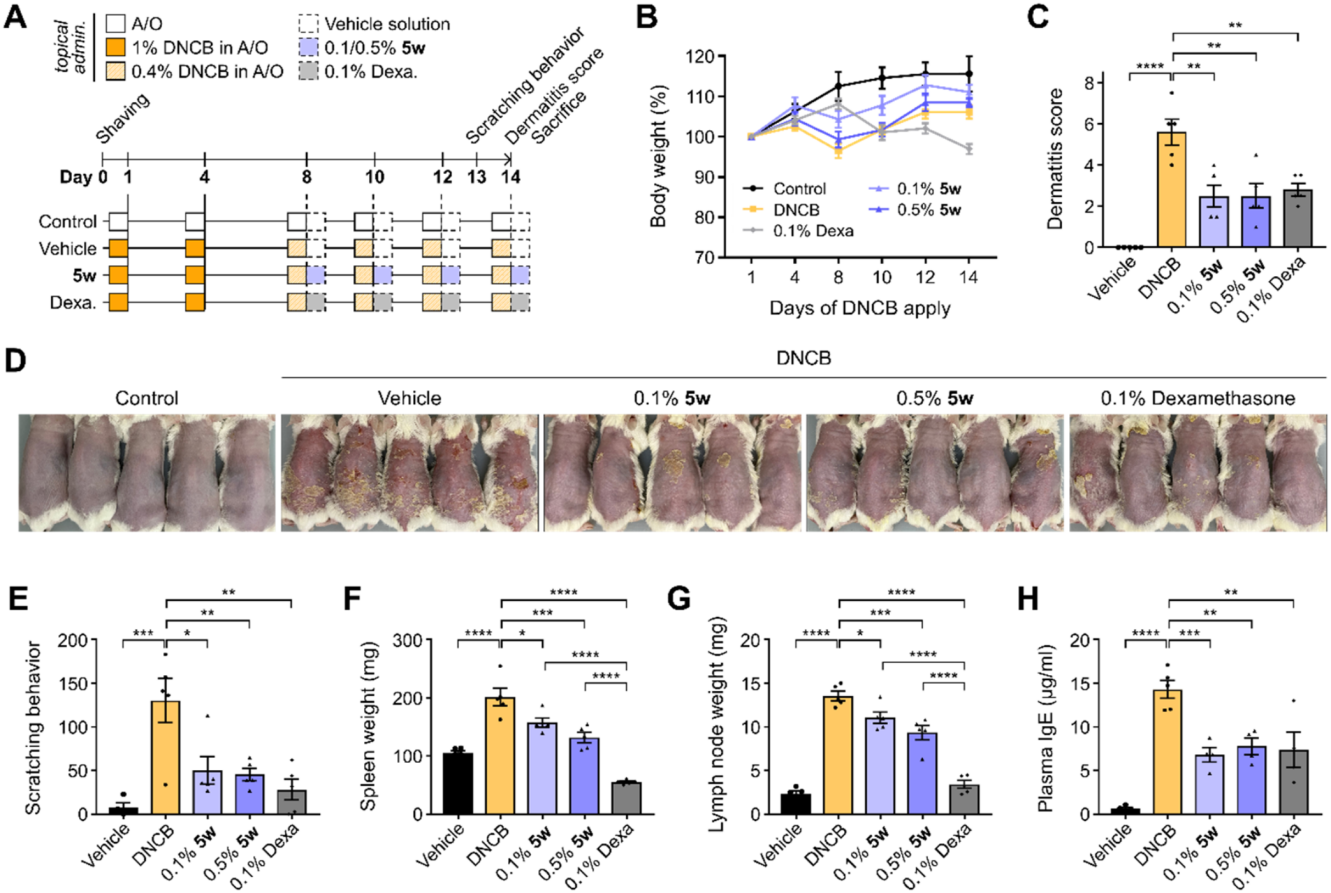
**5w** Alleviates AD-like Symptoms by Suppressing Local and Systemic Inflammation. (A) Experimental scheme of DNCB-induced AD mouse model. Mice were topically treated with 1% DNCB in acetone/olive oil (A/O, 3:1) twice during the first 7 days, followed by 0.4% DNCB in A/O four times over the subsequent 7 days. During the latter period, 0.1 or 0.5% **5w**, or 0.1% dexamethasone, was coapplied topically. Each experimental group consisted of five mice (*n* = 5). (B) Body weight changes over the experimental period, normalized to baseline. (C) Dermatitis scores assessed at the end point (day 14), with each parameter—erythema, dryness, edema, erosion, and lichenification—scored from 0 to 3. (D) Dorsal skin images of each group. (E) Number of scratching episodes for 30 min from each group (day 13). (F, G) Weight of spleen and lymph node (day 14). (H) IgE level in mouse plasma measured by ELISA at the end of the experiment (day 14). Data are presented as mean ± SEM. Statistical significance is indicated as follows: *p < 0.05, **p < 0.01, ***p < 0.001, ****p < 0.0001, one-way ANOVA with *Tukey*’s test.

To further assess the systemic effects of **5w** on the immune system, spleen and draining lymph node weights were measured postsacrifice. The spleen and lymph node weights were significantly increased in the DNCB group compared with the vehicle group, indicating splenomegaly^7,64^ and lymphadenopathy,^7,9,66^ which are commonly associated with systemic immune activation. Treatment with **5w** reduced immune organ weights in a concentration-dependent manner, likely as a consequence of dampened local skin inflammation, thereby indirectly alleviating systemic immune organ hypertrophy (Figure 5F,G). Unlike dexamethasone, which induces strong systemic immunosuppression, **5w** did not markedly reduce spleen or lymph node weights despite its comparable anti-inflammatory efficacy. This observation suggests that **5w** may exert its effects through a more localized or selective modulation of inflammatory pathways rather than broad suppression of immune organ function. Statistical analysis confirmed that the reductions in spleen and lymph node weights observed with 0.1 and 0.5% **5w** were significantly less pronounced than those induced by dexamethasone. Consistent with its local anti-inflammatory efficacy, **5w** treatment significantly lowered plasma IgE levels, which were markedly elevated in the DNCB group as a result of enhanced Th2 immune activation (Figure 5H). Notably, this reduction was comparable to that observed with dexamethasone, a potent immunosuppressant. Given the central role of IgE in mediating allergen-specific immune responses, these findings suggest that **5w** may indirectly exert systemic immunomodulatory effects by attenuating type 2 immunity originating from allergic skin inflammation. As IgE is a key clinical marker often elevated in AD patients,^67^ these results underscore the therapeutic potential of Keap1-Nrf2 activation for alleviating AD symptoms. Altogether, administration of **5w** effectively ameliorated local cutaneous inflammation, which in turn suppressed systemic immune activation, thereby exerting therapeutic effects against DNCB-induced AD-like symptoms.

### **5w** Ameliorates Skin Inflammation and Enhances Barrier Function in DNCB-induced Mouse Dorsal Skin Tissue

Following the improvement of AD-like symptoms and systemic immune parameters by topical **5w** treatment in the DNCB-induced mouse model, we examined whether these effects were also associated with changes in local skin pathology, including immune cell infiltration, pro-inflammatory cytokine expression, and skin barrier integrity. We first conducted histopathological analysis of lesional skin to assess changes in immune cell infiltration in the dermis following topical administration with **5w** or dexamethasone. After sacrifice, dorsal skin tissue was collected and subjected to immunohistological staining to evaluate epidermal thickness and immune cell infiltration,^64,66^ a key contributor to the initiation and amplification of allergic inflammation in AD.

In line with the dermatitis score results, epidermal thickness was markedly increased in the DNCB group compared to the vehicle group (Figure 6A,B). In contrast, **5w** treatment significantly attenuated the thickening in a concentration-dependent manner, while dexamethasone achieved the greatest reduction. Subsequent H&E staining analysis of immune cell infiltration revealed markedly increased eosinophil counts in the DNCB group, whereas **5w** induced a concentration-dependent reduction in eosinophil infiltration, with effects evident at both 0.1 and 0.5%. (Figure 6A,C). The stronger reduction in eosinophil counts observed with dexamethasone may be attributed to its glucocorticoid activity, which is known to induce eosinophil apoptosis.^68^ In comparison, **5w** significantly attenuated inflammatory responses while exerting a more limited effect on eosinophil infiltration, suggesting a distinct anti-inflammatory profile compared with dexamethasone. Mast cell infiltration was next examined by toluidine blue staining to assess whether similar trends were observed in this key effector cell type in AD pathology. As expected, **5w** decreased mast cell counts in a concentration-dependent manner, with a near-significant reduction in the 0.1% group (*p* = 0.0662) and a significant reduction in the 0.5% group (Figure 6D,E). The positive control produced a nonsignificant reduction, indicating that **5w** provided more effective suppression of allergic inflammatory cell influx into inflamed skin (Figure 6E).

**Figure 6:**
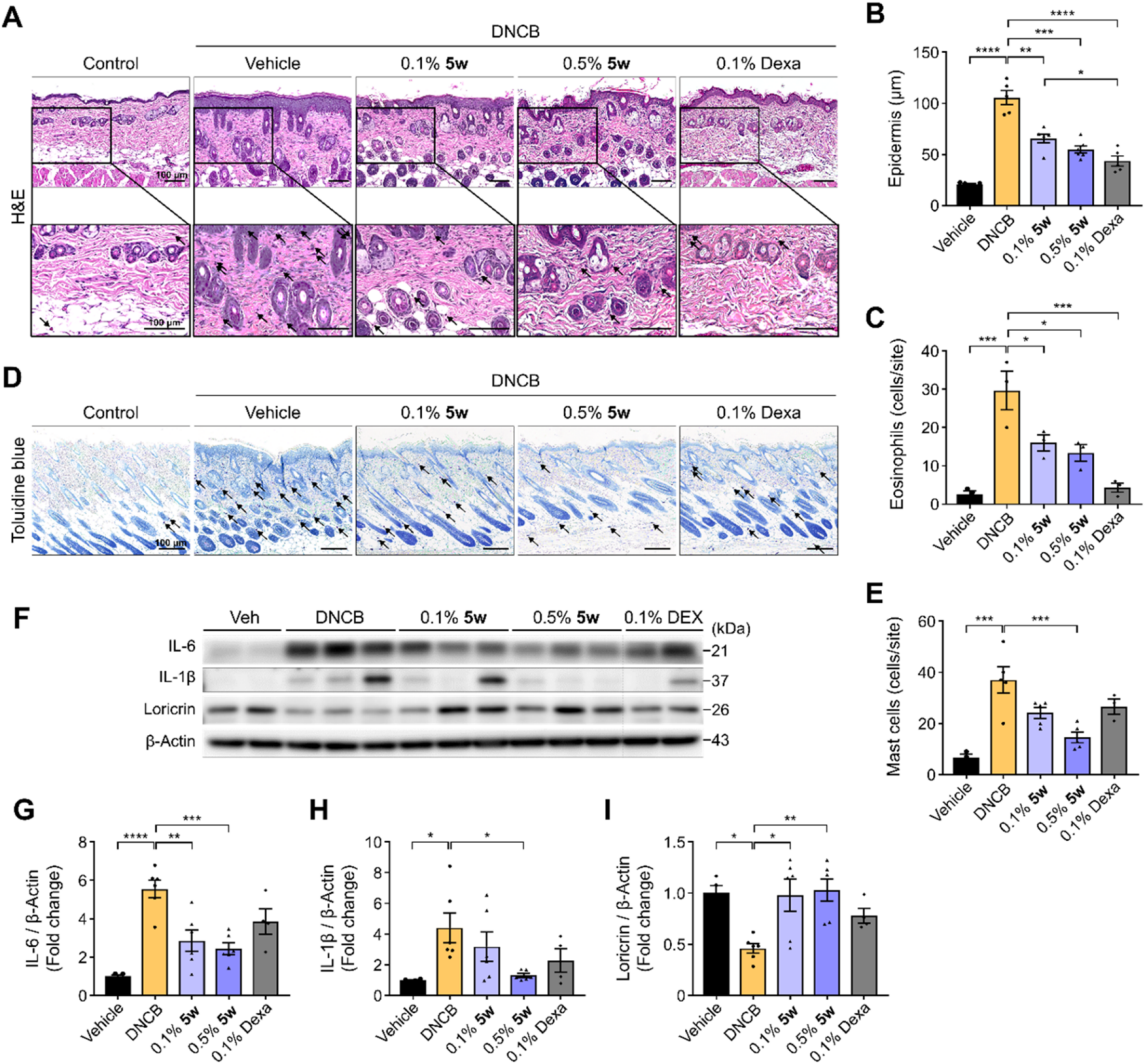
**5w** Mitigates Inflammation and Restores Barrier Integrity in Dorsal Skin of DNCB-Induced Mice. (A) Representative images of dorsal skin tissue stained with H&E from each group at a magnification of 20× (upper) and 40× (lower). The black arrows denote eosinophils. Scale bars, 100 μm. (B) Quantification of epidermal thickness of skin from each group. (C) Quantification of infiltrated eosinophils based on H&E staining. (D) Representative images of dorsal skin tissue stained with toluidine blue from each group at a magnification of 20×. The black arrows indicate mast cells. Scale bars, 100 μm. (E) Quantification of infiltrated mast cells based on toluidine blue staining. (F) Western blot analysis of IL-6, IL-1β, and loricrin in dorsal skin tissue of each group. Relative expression of IL-6 (G), IL-1β (H), and loricrin (I) were normalized to β-actin. Statistical significance is indicated as follows: *p < 0.05, **p < 0.01, ***p < 0.001 and ****p < 0.0001, one-way ANOVA with *Tukey*’s test. Data are presented as mean ± SEM. Five animals were used per group; epidermal thickness was analyzed in all animals, whereas other analyses were performed using 2–3 animals per group.

To determine whether the reduction in immune cell infiltration by **5w** was accompanied by changes in inflammatory signaling, we analyzed the expression of pro-inflammatory cytokines in skin tissue by Western blot. In the DNCB group, IL-6 and IL-1β levels were markedly elevated; however, treatment with **5w** remarkably reduced IL-6 in a concentration-dependent manner and suppressed IL-1β, with a significant reduction at 0.5% (Figure 6F–H). In contrast, dexamethasone treatment produced only a nonsignificant reduction in both cytokines, indicating that **5w** exerted stronger suppression of pro-inflammatory cytokine production in skin lesions. Furthermore, given that loricrin, a major component of the cornified envelope of skin barrier,^64,69^ is frequently reduced in AD lesions due to inflammation-driven suppression, we subsequently assessed its expression in skin tissues by Western blot to evaluate the barrier-preserving potential of **5w**. In the DNCB group, loricrin expression was markedly reduced compared with the vehicle control, whereas topical administration of **5w** restored its levels toward those of the control group (Figure 6F,I). Dexamethasone treatment showed only a nonsignificant trend toward recovery. Restoration of loricrin suggests genuine barrier support beyond anti-inflammatory action and hints that Nrf2 activation may confer more durable epidermal resilience, although direct *in vivo* evidence for this mechanism remains to be shown.

Taken together, these findings suggest that activating Nrf2 to counter inflammation—a key driver of AD pathophysiology—may represent an alternative or complementary strategy to conventional immunosuppressive approaches. While glucocorticoids act primarily through immune suppression to relieve symptoms, **5w** attenuates inflammation and restores barrier integrity *in vivo*, suggesting its potential impact on underlying AD pathology. Overall, the alignment between *in vitro* Nrf2-mediated cytoprotective effects and *in vivo* findings of reduced inflammation and improved barrier integrity highlights **5w** as a topical candidate with therapeutic potential in AD.

## Conclusions

To exploit the distinct electronic properties and redox potential of selenone, a vinyl selenone core was introduced as an isosteric replacement for vinyl sulfone and sulfoximine cores in scaffolds previously reported to exhibit anti-inflammatory and antioxidant activities. Consistent with our design rationale, the synthesized vinyl selenone derivatives exhibited markedly improved Nrf2-activating potency, attributable to the unique physicochemical properties of selenium that likely enhanced their biological activities. Among the synthesized compounds, **5w** emerged as the most potent Nrf2 activator *in vitro*, exhibiting more than 100-fold increase in potency relative to the previously reported vinyl sulfone analog. Its therapeutic potential as a regulator of inflammation and oxidative damage was further confirmed in both *in vitro* (HaCaT keratinocytes and Raw264.7 macrophages) and *in vivo* (DNCB-induced AD mouse model). **5w** promoted Nrf2 activation and nuclear translocation, resulting in robust induction of antioxidant enzymes such as HO-1, GCLM, GCLC, and NQO1 in HaCaT keratinocytes. Moreover, **5w** suppressed cytokine-driven inflammatory responses by downregulating Th1- and Th2-associated mediators, likely involving Nrf2 activation. In inflammatory macrophages, **5w** exerted antioxidant, antinitrosative, and anti-inflammatory activities by reducing ROS and NO production and suppressing pro-inflammatory cytokines. Consistent with the *in vitro* findings, topical administration of **5w** effectively restored skin barrier function and ameliorated AD-like symptoms by inhibiting local and systemic inflammation in the DNCB-induced AD mouse model. Collectively, these findings underscore the potential of selenium-based Nrf2 activators as a novel and effective therapeutic strategy for the treatment of inflammatory skin diseases such as AD.

## Experimental Section

### General Methods

All chemicals, reagents, and solvents were purchased from commercial suppliers as reagent-grade materials and used without further purification. Reported yields refer to purified compounds and were not optimized. The synthesized compounds were characterized using thin-layer chromatography (TLC), ^1^H and ^13^C nuclear magnetic resonance (NMR), melting point (MP), high-resolution mass spectrometry (HRMS), and high-performance liquid chromatography (HPLC). Reactions were monitored using analytical thin-layer chromatography plates (Merck, Cat No. 1.05715), with visualization under ultraviolet light at 254 and 280 nm. Compound purification was performed by MPLC (Biotage, Isolera one). Melting points were determined in open capillary tubes using OptiMelt melting point apparatus (Stanford Research Systems, Inc.). NMR spectra were acquired on Bruker spectrometers at 400 MHz (^1^H)/100 MHz (^13^C). Chemical shifts (δ) were reported in parts per million (ppm) downfield from tetramethylsilane (TMS). HPLC analyses were conducted on a Waters E2695 system equipped with a YMC-Triart C18 column/S-5 μm/12 nm/Lot No. 17452 (150 mm × 4.6 mm diameter). The HPLC parameters included: flow rate of 1.0 mL/min, DW (0.1% AcOH)/acetonitrile, 10/90 → 100/0 gradient in 15 min, +5 min isocratic, λ = 254 and 280 nm. All compounds were >95% pure. HRMS was conducted using electrospray ionization on a Q-Exactive mass spectrometer (Thermo Fisher Scientific, Vanquish UHPLC system) connected to a triple quadrupole mass spectrometer (Thermo Finnigan, TSQ Altis Triple Quadrupole Mass Spectrometer) with an ESI source.

### General Procedure for the Intermediate Compounds **1a–1e** (Method A)

Selenium powder (3.0 equiv) and TMSCN (2.0 equiv) were added to a solution of the desired aryl boronic acid derivative (1.0 equiv) in dimethyl sulfoxide (DMSO). The reaction mixture was stirred at 120 °C (7 h). After completion, the reaction was diluted with distilled water and extracted with ethyl acetate. The combined organic layers were dried over anhydrous Na_2_SO_4_ and concentrated under reduced pressure. The resulting residue was purified by column chromatography on silica gel (SiO_2_). Detailed experimental procedures and data for each intermediate are provided in the Supporting Information.

### General Procedure for the Intermediate Compounds **2a–2e** (Method B)

Selenium powder (2.0 equiv), KOH (2.0 equiv), and CuO (0.1 equiv) were added to a solution of the desired aryl halide (1.0 equiv) in DMSO. The reaction mixture was stirred at room temperature (2 h) and then diluted with distilled water. The aqueous phase was extracted with ethyl acetate, and the combined organic layers were dried over anhydrous Na_2_SO_4_. The solution was concentrated under reduced pressure, and the crude product was purified by column chromatography on SiO_2_. Detailed experimental procedures and data for each intermediate are provided in the Supporting Information.

### General Procedure for the Intermediate Compounds **3a–3h** (Method C)

To a solution of aryl selenocyanide intermediates (**1a**–**1e**) (1.0 equiv) or diaryl diselenide intermediates (**2a**–**2e**) (0.5 equiv) in methanol were added potassium carbonate (K_2_CO_3_) (1.0–2.0 equiv), diethyl (*p*-toluenesulfonyloxymethyl)­phosphonate (1.0 equiv), and NaBH_4_ (1.0–1.3 equiv) at room temperature and stirred (2 h). After completion, the reaction mixture was diluted with distilled water and extracted with dichloromethane (DCM). The combined organic layers were dried over anhydrous Na_2_SO_4_ and concentrated under reduced pressure. The resulting residue was purified by column chromatography on SiO_2_. Detailed experimental procedures and data for each intermediate are provided in the Supporting Information.

### General Procedure for the Intermediate Compounds **4a–4aa** and **6a–6u** (Method D)

To a solution of intermediates (**3a**–**3h**) (1.0 equiv) in anhydrous tetrahydrofuran (THF) was added benzaldehyde or picolinaldehyde derivatives (1.2 equiv) and either a 2.0 M *n*-BuLi solution in cyclohexane (2.2 equiv) dropwise under a nitrogen atmosphere at −78 °C. The reaction mixture was stirred at room temperature (2–48 h), then diluted with water and extracted with ethyl acetate. The combined organic layers were dried over anhydrous Na_2_SO_4_ and concentrated under reduced pressure. The crude product was purified by column chromatography on SiO_2_. Detailed experimental procedures and data for each intermediate are provided in the Supporting Information.

### General Procedure for the Final Compounds **5a–5aa** and **7a–7u** (Method E)

70–75% mCPBA (1.5–5.0 equiv) was added to a solution of vinyl selenide derivatives (**4a**–**4aa** and **6a**–**6u**) (1.0 equiv) in DCM at 0 °C. The reaction mixture was then refluxed (18–48 h). Upon completion, excess mCPBA was quenched with a saturated sodium hydrogen carbonate (NaHCO_3_) solution, and the organic phase was extracted with ethyl acetate. The combined organic layers were dried over anhydrous Na_2_SO_4_ and concentrated under reduced pressure. The crude product was purified by column chromatography on SiO_2_.

#### Preparation of (*E*)-(2-(Phenylselenonyl)­vinyl)­benzene (**5a**)

Using Method E, **4a** (0.58 g, 2.24 mmol) and 70–75% mCPBA (0.77 g, 3.36 mmol) gave 0.27 g (44%) of **5a** as a white solid; *R*_*f*_ = 0.25 (*n*-hexane:EtOAc = 3:1); mp: 93.3–95.6 °C; ^1^H NMR (400 MHz, DMSO-*d*_6_) δ 8.17 (d, *J* = 15.4 Hz, 1H), 8.00 (d, *J* = 7.9 Hz, 2H), 7.84–7.74 (m, 6H), 7.50–7.47 (m, 3H); ^13^C NMR (100 MHz, CDCl_3_) δ 145.4, 142.2, 134.2, 131.9, 131.6, 130.3, 129.3, 128.8, 127.5, 126.9; HPLC purity: 10.3 min, 99.1%; HRMS (M + H)^+^ (ESI^+^) 293.0069 [M + H]^+^ (calcd for C_14_H_12_O_2_SeH^+^ 292.0003).

#### Preparation of (*E*)-1-Methoxy-2-(styrylselenonyl)­benzene (**5b**)

Using Method E, **4b** (0.12 g, 0.42 mmol) and 70–75% mCPBA (0.31 g, 1.27 mmol) gave 0.03 g (24%) of **5b** as a white solid; *R*_*f*_ = 0.42 (100% EtOAc); mp: 146.5–147.1 °C; ^1^H NMR (400 MHz, CDCl_3_) δ 8.12 (d, *J* = 7.9 Hz, 1H), 7.88 (d, *J* = 15.5 Hz, 1H), 7.63 (t, *J* = 7.6 Hz, 1H), 7.56–7.50 (m, 2H), 7.28–7.22 (m, 4H), 7.19 (t, *J* = 7.6 Hz, 1H), 7.08 (d, *J* = 8.4 Hz, 1H), 4.00 (s, 3H); ^13^C NMR (100 MHz, CDCl_3_) δ 157.1, 144.6, 136.0, 132.0, 131.6, 130.2, 129.2, 128.9, 128.8, 128.6, 121.7, 112.8, 56.7; HPLC purity: 9.2 min, 100.0%; HRMS (M + H)^+^ (ESI^+^) 323.0177 [M + H]^+^ (calcd for C_15_H_14_O_3_SeH^+^ 323.0108).

#### Preparation of (*E*)-1-Methoxy-2-(2-((2-methoxyphenyl)­selenonyl)­vinyl)­benzene (**5c**)

Using Method E, **4c** (0.089 g, 0.28 mmol) and 70–75% mCPBA (0.20 g, 0.84 mmol) gave 0.05 g (52%) of **5c** as a white solid; *R*_*f*_ = 0.42 (100% EtOAc); mp: 140.7–143.3 °C; ^1^H NMR (400 MHz, DMSO-*d*_6_) δ 7.97 (d, *J* = 15.4 Hz, 1H), 7.94–7.89 (m, 2H), 7.79–7.73 (m, 2H), 7.52–7.47 (m, 1H), 7.35 (d, *J* = 7.8 Hz, 1H), 7.26 (td, *J* = 7.4, 1.0 Hz, 1H), 7.15 (d, *J* = 8.0 Hz, 1H), 7.03 (td, *J* = 7.2, 0.7 Hz, 1H), 3.94 (s, 3H), 3.91 (s, 3H); ^13^C NMR (100 MHz, CDCl_3_) 159.0, 157.1, 140.8, 135.8, 132.8, 131.6, 130.4, 130.1, 128.9, 121.6, 120.9, 120.8, 112.7, 111.3, 56.5, 55.5; HPLC purity: 11.5 min, 95.1%; HRMS (M + H)^+^ (ESI^+^) 353.0277 [M + H]^+^ (calcd for C_16_H_16_O_4_SeH^+^ 353.0214).

#### Preparation of (*E*)-1-Fluoro-2-(2-((2-methoxyphenyl)­selenonyl)­vinyl)­benzene (**5d**)

Using Method E, **4d** (0.027 g, 0.084 mmol) and 70–75% mCPBA (0.058 g, 0.25 mmol) gave 0.008 g (28%) of **5d** as a white solid; *R*_*f*_ = 0.51 (100% EtOAc); mp: 117.0–120.1 °C; ^1^H NMR (400 MHz, CDCl_3_) δ 8.12 (d, *J* = 7.9 Hz, 1H), 7.92 (d, *J* = 15.6 Hz, 1H), 7.66–7.57 (m, 2H), 7.52–7.41 (m, 2H), 7.27–7.07 (m, 4H), 4.00 (s, 3H); ^13^C NMR (100 MHz, CDCl_3_) 161.6 (d, *J*_C–F_ = 254.5 Hz), 157.1, 138.1 (d, *J*_C–F_ = 1.3 Hz), 136.1, 133.1 (d, *J*_C–F_ = 9.1 Hz), 132.2 (d, *J*_C–F_ = 9.8 Hz), 130.9 (d, *J*_C–F_ = 2.3 Hz), 130.0, 128.9, 124.9 (d, *J*_C–F_ = 3.6 Hz), 121.7, 120.2 (d, *J*_C–F_ = 11.2 Hz), 116.5 (d, *J*_C–F_ = 21.5 Hz), 112.8, 56.6; HPLC purity: 11.5 min, 99.3%; HRMS (M + H)^+^ (ESI^+^) 341.0085 [M + H]^+^ (calcd for C_15_H_13_FO_3_SeH^+^ 341.0014).

#### Preparation of (*E*)-1-Chloro-2-(2-((2-methoxyphenyl)­selenonyl)­vinyl)­benzene (**5e**)

Using Method E, **4e** (2.8 g, 8.53 mmol) and 70–75% mCPBA (6.3 g, 25.6 mmol) gave 1.2 g (39%) of **5e** as a white solid; *R*_*f*_ = 0.42 (100% EtOAc); mp: 132.1–138.3 °C; ^1^H NMR (400 MHz, DMSO-*d*_6_) δ 8.23 (d, *J* = 15.2 Hz, 1H), 8.06–7.99 (m, 2H), 7.95 (dd, *J* = 7.9, 1.6 Hz, 1H), 7.80–7.76 (m, 1H), 7.62 (dd, *J* = 8.0, 1.2 Hz, 1H), 7.53 (td, *J* = 7.4, 1.6 Hz, 1H), 7.45 (td, *J* = 7.5, 0.9 Hz, 1H), 7.37 (dd, *J* = 8.1, 0.5 Hz, 1H), 7.29 (td, *J* = 0.9, 7.5 Hz, 1H), 3.94 (s, 3H); ^13^C NMR (100 MHz, CDCl_3_) 157.2, 140.9, 136.2, 135.2, 132.3, 131.8, 130.6, 130.4, 129.8, 129.0, 128.7, 127.4, 121.8, 112.8, 56.6; HPLC purity: 11.2 min, 99.4%; HRMS (M + H)^+^ (ESI^+^) 356.9781 [M + H]^+^ (calcd for C_15_H_13_ClO_3_SeH^+^ 356.9718).

#### Preparation of (*E*)-1-Methoxy-2-((2-(trifluoromethyl)­styryl)­selenonyl)­benzene (**5f**)

Using Method E, **4f** (0.058 g, 0.16 mmol) and 70–75% mCPBA (0.11 g, 0.47 mmol) gave 0.029 g (47%) of **5f** as a white solid; *R*_*f*_ = 0.50 (100% EtOAc); mp: 144.4–149.8 °C; ^1^H NMR (400 MHz, CDCl_3_) δ 8.10 (dd, *J* = 25.9, 1.6 Hz, 1H), 8.01 (d, *J* = 15.6 Hz, 1H), 7.67–7.59 (m, 2H), 7.57 (d, *J* = 15.6 Hz, 1H), 7.50 (td, *J* = 7.9, 1.7 Hz, 1H), 7.39–7.33 (m, 2H), 7.20 (td, *J* = 7.7, 1.0 Hz, 1H), 7.10 (dd, *J* = 8.1, 0.6 Hz, 1H), 3.99 (s, 3H); ^13^C NMR (100 MHz, CDCl_3_) 157.3, 140.7 (d, *J*_C–F_ = 2.0 Hz), 136.3, 133.5, 132.5, 130.9 (d, *J*_C–F_ = 1.3 Hz), 130.8, 129.3, 129.1 (q, *J*_C–F_ = 30.8 Hz), 128.9 (d, *J*_C–F_ = 46.9 Hz), 126.5 (q, *J*_C–F_ = 5.5 Hz), 123.6 (q, *J*_C–F_ = 272.5 Hz), 121.7, 112.8, 56.6; HPLC purity: 12.7 min, 99.5%; HRMS (M + H)^+^ (ESI^+^) 391.0047 [M + H]^+^ (calcd for C_16_H_13_F_3_O_3_SeH^+^ 390.9982).

#### Preparation of (*E*)-1-Methoxy-2-((2-(trifluoromethoxy)­styryl)­selenonyl)­benzene (**5g**)

Using Method E, **4g** (0.16 g, 0.41 mmol) and 70–75% mCPBA (0.29 g, 1.24 mmol) gave 0.071 g (43%) of **5g** as a white solid; *R*_*f*_ = 0.84 (100% EtOAc); mp: 143.2–149.6 °C; ^1^H NMR (400 MHz, DMSO-*d*_6_) δ 8.24 (d, *J* = 15.4 Hz, 1H), 8.07 (d, *J* = 7.8 Hz, 1H), 7.95 (dd, *J* = 7.9, 1.5 Hz, 1H), 7.85 (d, *J* = 15.4 Hz, 1H), 7.78 (td, *J* = 7.9, 1.6 Hz, 1H), 7.66 (td, *J* = 7.9, 1.5 Hz, 1H), 7.54–7.51 (m, 2H), 7.37 (d, *J* = 8.2 Hz, 1H), 7.29 (t, *J* = 7.9 Hz, 1H), 3.92 (s, 3H); ^13^C NMR (100 MHz, CDCl_3_) 157.1, 147.9, 138.2, 136.2, 132.7 (d, *J*_C–F_ = 7.2 Hz), 132.3, 129.8, 128.9, 127.3, 125.0, 121.7, 121.6, 120.9, 112.7, 56.5; HPLC purity: 12.9 min, 99.4%; HRMS (M + H)^+^ (ESI^+^) 406.9999 [M + H]^+^ (calcd for C_16_H_13_F_3_O_4_SeH^+^ 406.9931).

#### Preparation of (*E*)-1-Chloro-2-(2-((3-methoxyphenyl)­selenonyl)­vinyl)­benzene (**5h**)

Using Method E, **4h** (0.03 g, 0.09 mmol) and 70–75% mCPBA (0.06 g, 0.27 mmol) gave 0.02 g (68%) of **5h** as a white solid; *R*_*f*_ = 0.32 (*n*-hexane:EtOAc = 1:1); mp: 111.1–112.3 °C; ^1^H NMR (400 MHz, CDCl_3_) δ 8.23 (d, *J* = 15.5 Hz, 1H), 7.60–7.52 (m, 4H), 7.46 (d, *J* = 7.9 Hz, 1H), 7.39 (t, *J* = 7.5 Hz, 1H), 7.34–7.26 (m, 2H), 7.22 (d, *J* = 7.8 Hz, 1H), 3.90 (s, 3H); ^13^C NMR (100 MHz, CDCl_3_) δ 160.8, 142.7, 141.3, 135.4, 132.6, 131.2, 130.6, 130.5, 130.0, 128.8, 127.4, 121.1, 118.9, 111.1, 56.0; HPLC purity: 11.0 min, 97.1%; HRMS (M + H)^+^ (ESI^+^) 356.9785 [M + H]^+^ (calcd for C_15_H_13_ClO_3_SeH^+^ 356.9718).

#### Preparation of (*E*)-1-Chloro-2-(2-((4-methoxyphenyl)­selenonyl)­vinyl)­benzene (**5i**)

Using Method E, **4i** (0.025 g, 0.074 mmol) and 70–75% mCPBA (0.051 g, 0.22 mmol) gave 0.013 g (49%) of **5i** as a white solid; *R*_*f*_ = 0.78 (EtOAc:CH_3_OH = 10:1); mp: 87.1–92.5 °C; ^1^H NMR (400 MHz, DMSO-*d*_6_) δ 8.27 (d, *J* = 15.4 Hz, 1H), 8.02 (d, *J* = 15.4 Hz, 1H), 7.93 (d, *J* = 8.2 Hz, 3H), 7.61 (d, *J* = 8.0 Hz, 1H), 7.52 (t, *J* = 7.8 Hz, 1H), 7.45 (t, *J* = 7.8 Hz, 1H), 7.28 (d, *J* = 8.6 Hz, 2H), 3.88 (s, 3H); ^13^C NMR (100 MHz, CDCl_3_) 163.9, 159.2, 140.9, 133.5, 133.0, 132.0, 129.2, 128.9, 120.9, 120.6, 115.4, 111.3, 55.8, 55.5; HPLC purity: 11.3 min, 95.6%; HRMS (M + H)^+^ (ESI^+^) 353.0282 [M + H]^+^ (calcd for C_15_H_13_ClO_3_SeH^+^ 353.0214).

#### Preparation of (*E*)-1-Fluoro-2-(2-((4-methoxyphenyl)­selenonyl)­vinyl)­benzene (**5j**)

Using Method E, **4j** (0.015 g, 0.046 mmol) and 70–75% mCPBA (0.032 g, 0.14 mmol) gave 0.007 g (45%) of **5j** as a white solid; *R*_*f*_ = 0.74 (EtOAc:CH_3_OH = 10:1); mp: 124.4–125.2 °C; ^1^H NMR (400 MHz, DMSO-*d*_6_) δ 8.15 (d, *J* = 15.6 Hz, 1H), 7.94 (d, *J* = 8.8 Hz, 2H), 7.88 (t, *J* = 8.4 Hz, 1H), 7.82 (d, *J* = 15.6 Hz, 1H), 7.57 (q, *J* = 7.0 Hz, 1H), 7.39–7.26 (m, 4H), 3.87 (s, 3H); ^13^C NMR (100 MHz, CDCl_3_) 164.1, 162.9, 138.1, 133.3 (d, *J*_C–F_ = 9.0 Hz), 132.9, 131.3 (d, *J*_C–F_ = 9.1 Hz), 131.1, 128.9, 124.9 (d, *J*_C–F_ = 3.4 Hz), 120.1, 116.6 (d, *J*_C–F_ = 21.4 Hz), 115.5, 55.8; HPLC purity: 11.2 min, 98.3%; HRMS (M + H)^+^ (ESI^+^) 341.0088 [M + H]^+^ (calcd for C_15_H_13_FO_3_SeH^+^ 341.0014).

#### Preparation of (*E*)-1-Chloro-2-(2-((4-methoxyphenyl)­selenonyl)­vinyl)­benzene (**5k**)

Using Method E, **4k** (0.025 g, 0.074 mmol) and 70–75% mCPBA (0.050 g, 0.22 mmol) gave 0.013 g (49%) of **5k** as a white solid; *R*_*f*_ = 0.78 (EtOAc:CH_3_OH = 10:1); mp: 148.3–149.6 °C; ^1^H NMR (400 MHz, DMSO-*d*_6_) δ 8.27 (d, *J* = 15.4 Hz, 1H), 8.02 (d, *J* = 15.7 Hz, 1H), 7.93 (d, *J* = 8.2 Hz, 3H), 7.61 (d, *J* = 8.0 Hz, 1H), 7.52 (t, *J* = 7.8 Hz, 1H), 7.45 (t, *J* = 7.8 Hz, 1H), 7.28 (d, *J* = 8.6 Hz, 2H), 3.88 (s, 3H); ^13^C NMR (100 MHz, CDCl_3_) 164.2, 140.7, 135.3, 132.7, 132.4, 131.1, 130.6, 130.1, 129.0, 128.7, 127.3, 115.5, 55.8; HPLC purity: 12.5 min, 99.5%; HRMS (M + H)^+^ (ESI^+^) 356.9786 [M + H]^+^ (calcd for C_15_H_13_ClO_3_SeH^+^ 356.9718).

#### Preparation of (*E*)-1-(2-((4-Methoxyphenyl)­selenonyl)­vinyl)-2-(trifluoromethyl)­benzene (**5l**)

Using Method E, **4l** (0.08 g, 0.22 mmol) and 70–75% mCPBA (0.15 g, 0.66 mmol) gave 0.04 g (48%) of **68c** as a white solid; *R*_*f*_ = 0.30 (*n*-hexane:EtOAc = 1:2); mp: 125.4–126.3 °C; ^1^H NMR (400 MHz, CDCl_3_) δ 8.16 (dd, *J* = 15.3, 1.8 Hz, 1H), 7.97–7.90 (m, 2H), 7.74 (d, *J* = 7.6 Hz, 1H), 7.65–7.53 (m, 3H), 7.18–7.08 (m, 3H), 3.90 (s, 3H); ^13^C NMR (100 MHz, CDCl_3_) δ 164.3, 140.8 (q, *J*_C–F_ = 1.9 Hz), 132.9, 132.5, 132.3, 130.9, 130.8 (q, *J*_C–F_ = 1.6 Hz), 129.1 (q, *J*_C–F_ = 30.6 Hz), 129.0, 128.5, 126.5 (q, *J*_C–F_ = 5.3 Hz), 123.6 (q, *J*_C–F_ = 272.5 Hz), 115.6, 55.9; HPLC purity: 11.1 min, 95.0%; HRMS (M + H)^+^ (ESI^+^) 391.0051 [M + H]^+^ (calcd for C_16_H_13_F_3_O_3_SeH^+^ 390.9982).

#### Preparation of (*E*)-5-((2-Chlorostyryl)­selenonyl)-1,2,3-trimethoxybenzene (**5m**)

Using Method E, **4m** (0.08 g, 0.21 mmol) and 70–75% mCPBA (0.15 g, 0.63 mmol) gave 0.054 g (62%) of **5m** as a white solid; *R*_*f*_ = 0.77 (100% EtOAc); mp: 140.1–143.4 °C; ^1^H NMR (400 MHz, DMSO-*d*_6_) δ 8.32 (d, *J* = 15.3 Hz, 1H), 8.03 (d, *J* = 15.3 Hz, 1H), 7.91 (d, *J* = 7.8 Hz, 1H), 7.61 (d, *J* = 8.0 Hz, 1H), 7.53 (t, *J* = 7.3 Hz, 1H), 7.46 (t, *J* = 7.5 Hz, 1H), 7.26 (s, 2H), 3.90 (s, 6H), 3.76 (s, 3H); ^13^C NMR (100 MHz, DMSO-*d*_6_) δ 154.4, 142.5, 139.1, 136.8, 134.6, 133.5, 133.2, 130.7, 130.1, 129.5, 128.4, 104.3, 60.8, 57.1; HPLC purity: 12.9 min, 98.9%; HRMS (M + H)^+^ (ESI^+^) 416.9994 [M + H]^+^ (calcd for C_17_H_17_ClO_5_SeH^+^ 416.9930).

#### Preparation of (*E*)-1-Fluoro-2-(styrylselenonyl)­benzene (**5n**)

Using Method E, **4n** (0.13 g, 0.44 mmol) and 70–75% mCPBA (0.51 g, 2.22 mmol) gave 0.11 g (83%) of **5n** as a white solid; *R*_*f*_ = 0.24 (*n*-hexane:EtOAc = 1:1); mp: 82.3–85.7 °C; ^1^H NMR (400 MHz, DMSO-*d*_6_) δ 8.27 (d, *J* = 15.3 Hz, 1H), 8.04 (t, *J* = 7.4 Hz, 1H), 7.91–7.83 (m, 4H), 7.60 (q, *J* = 8.0 Hz, 2H), 7.52–7.47 (m, 3H); ^13^C NMR (100 MHz, CDCl_3_) δ 159.4 (d, *J*_C–F_ = 252.4 Hz), 146.0, 136.6 (d, *J*_C–F_ = 7.8 Hz), 132.0, 131.5, 129.5, 129.3, 129.0, 128.8, 127.9, 125.5 (d, *J*_C–F_ = 3.2 Hz), 117.5 (d, *J*_C–F_ = 20.0 Hz); HPLC purity: 10.8 min, 98.4%; HRMS (M + H)^+^ (ESI^+^) 310.9975 [M + H]^+^ (calcd for C_14_H_11_FO_2_SeH^+^ 310.9908).

#### Preparation of (*E*)-1-Fluoro-2-((2-methoxystyryl)­selenonyl)­benzene (**5o**)

Using Method E, **4o** (0.12 g, 0.39 mmol) and 70–75% mCPBA (0.29 g, 1.18 mmol) gave 0.01 g (10%) of **5o** as a white solid; *R*_*f*_ = 0.61 (100% EtOAc); mp: 110.4–111.7 °C; ^1^H NMR (400 MHz, CDCl_3_) δ 8.14 (t, *J* = 7.0 Hz, 1H), 8.02 (d, *J* = 15.3 Hz, 1H), 7.71–7.63 (m, 2H), 7.49–7.38 (m, 3H), 7.28 (t, *J* = 8.0 Hz, 1H), 7.01 (t, *J* = 7.5 Hz, 1H), 6.97 (d, *J* = 8.3 Hz, 1H); ^13^C NMR (100 MHz, CDCl_3_) δ 159.5 (d, *J*_C–F_ = 252.7 Hz), 159.3, 158.3, 142.1, 136.3 (d, *J*_C–F_ = 7.7 Hz), 133.3, 132.3, 129.8 (d, *J*_C–F_ = 18.7 Hz), 129.4, 129.1, 125.4 (d, *J*_C–F_ = 3.5 Hz), 121.0, 120.4, 117.4 (d, *J*_C–F_ = 20.2 Hz), 111.4, 55.6; HPLC purity: 10.2 min, 100.0%; HRMS (M + H)^+^ (ESI^+^) 341.0088 [M + H]^+^ (calcd for C_15_H_13_FO_3_SeH^+^ 341.0014).

#### Preparation of (*E*)-1-Fluoro-2-(2-((2-fluorophenyl)­selenonyl)­vinyl)­benzene (**5p**)

Using Method E, **4p** (0.12 g, 0.42 mmol) and 70–75% mCPBA (0.31 g, 1.26 mmol) gave 0.04 g (26%) of **5p** as a white solid; *R*_*f*_ = 0.72 (100% EtOAc); mp: 126.3–128.0 °C; ^1^H NMR (400 MHz, CDCl_3_) δ 8.13 (t, *J* = 7.0 Hz, 1H), 7.98 (d, *J* = 15.6 Hz, 1H), 7.72 (q, *J* = 7.3 Hz, 1H), 7.61–7.41 (m, 4H), 7.31 (t, *J* = 8.7 Hz, 1H), 7.24 (t, *J* = 7.6 Hz, 1H), 7.16 (t, *J* = 9.6 Hz, 1H); ^13^C NMR (100 MHz, CDCl_3_) δ 161.7 (d, *J*_C–F_ = 256.0 Hz), 159.5 (d, *J*_C–F_ = 252.5 Hz), 139.4, 136.7 (d, *J*_C–F_ = 5.4 Hz), 133.7 (d, *J*_C–F_ = 8.9 Hz), 131.2, 129.3 (d, *J*_C–F_ = 16.2 Hz), 129.1, 125.6 (d, *J*_C–F_ = 3.3 Hz), 125.0 (d, *J*_C–F_ = 3.6 Hz), 119.8 (d, *J*_C–F_ = 9.4 Hz), 117.6 (d, *J*_C–F_ = 20.0 Hz), 116.6 (d, *J*_C–F_ = 21.3 Hz); HPLC purity: 10.1 min, 99.4%; HRMS (M + H)^+^ (ESI^+^) 328.9883 [M + H]^+^ (calcd for C_14_H_10_F_2_O_2_SeH^+^ 328.9814).

#### Preparation of (*E*)-1-Chloro-2-(2-((2-fluorophenyl)­selenonyl)­vinyl)­benzene (**5q**)

Using Method E, **4q** (0.27 g, 0.82 mmol) and 70–75% mCPBA (0.95 mmol, 4.12 mmol) gave 0.15 g (58%) of **5q** as a white solid; *R*_*f*_ = 0.24 (*n*-hexane:EtOAc = 1:1); mp: 112.8–118.0 °C; ^1^H NMR (400 MHz, DMSO-*d*_6_) δ 8.41 (d, *J* = 15.2 Hz, 1H), 8.13 (d, *J* = 15.2 Hz, 1H), 8.07–8.03 (m, 1H), 8.00 (d, *J* = 7.7 Hz, 1H), 7.90 (q, *J* = 7.3 Hz, 1H), 7.65–7.60 (m, 3H), 7.55 (t, *J* = 7.5 Hz, 1H), 7.46 (t, *J* = 7.5 Hz, 1H); ^13^C NMR (100 MHz, CDCl_3_) 159.5 (d, *J*_C–F_ = 252.5 Hz), 142.1, 136.6 (d, *J*_C–F_ = 7.9 Hz), 135.5, 132.7, 130.9, 130.6, 129.8, 129.2 (d, *J*_C–F_ = 18.7 Hz), 129.1, 128.8, 127.4, 125.6 (d, *J*_C–F_ = 3.2 Hz), 117.5 (d, *J*_C–F_ = 20.1 Hz); HPLC purity: 11.9 min, 97.5%; HRMS (M + H)^+^ (ESI^+^) 344.9579 [M + H]^+^ (calcd for C_14_H_10_ClFO_2_SeH^+^ 344.9519).

#### Preparation of (*E*)-1-Fluoro-2-((2-(trifluoromethyl)­styryl)­selenonyl)­benzene (**5r**)

Using Method E, **4r** (0.15 g, 0.42 mmol) and 70–75% mCPBA (0.31 g, 1.27 mmol) gave 0.04 g (24%) of **5r** as a white solid; *R*_*f*_ = 0.73 (100% EtOAc); mp: 101.8–103.0 °C; ^1^H NMR (400 MHz, CDCl_3_) δ 8.31 (d, *J* = 15.2 Hz, 1H), 8.15 (t, *J* = 7.0 Hz, 1H), 7.79–7.56 (m, 5H), 7.46 (t, *J* = 7.7 Hz, 1H), 7.37–7.28 (m, 2H); ^13^C NMR (100 MHz, CDCl_3_) δ 159.5 (d, *J*_C–F_ = 253.2 Hz), 142.1, 136.8 (d, *J*_C–F_ = 8.9 Hz), 132.6, 132.5, 132.2, 130.4, 129.2 (d, *J*_C–F_ = 60.2 Hz), 129.1, 128.7, 126.6 (q, *J*_C–F_ = 24.1 Hz), 125.7 (d, *J*_C–F_ = 3.4 Hz), 123.5, (q, *J*_C–F_ = 272.5 Hz), 117.6 (d, *J*_C–F_ = 20.1 Hz); HPLC purity: 11.2 min, 96.1%; HRMS (M + H)^+^ (ESI^+^) 378.9849 [M + H]^+^ (calcd for C_15_H_10_F_4_O_2_SeH^+^ 378.9782).

#### Preparation of (*E*)-1-Fluoro-2-((2-(trifluoromethoxy)­styryl)­selenonyl)­benzene (**5s**)

Using Method E, **4s** (0.15 g, 0.42 mmol) and 70–75% mCPBA (0.31 g, 1.27 mmol) gave 0.04 g (24%) of **5s** as a white solid; *R*_*f*_ = 0.73 (100% EtOAc); mp: 97.3–98.0 °C; ^1^H NMR (400 MHz, CDCl_3_) δ 8.18–8.06 (m, 2H), 7.71 (q, *J* = 7.0 Hz, 1H), 7.63 (d, *J* = 7.6 Hz, 1H), 7.57–7.42 (m, 3H), 7.41–7.28 (m, 3H); ^13^C NMR (100 MHz, CDCl_3_) δ 159.5 (d, *J*_C–F_ = 252.9 Hz), 148.0, 139.5, 136.7 (d, *J*_C–F_ = 7.9 Hz), 133.1, 131.7, 129.9, 129.3 (d, *J*_C–F_ = 18.7 Hz), 129.1, 127.3, 125.6 (d, *J*_C–F_ = 3.3 Hz), 124.7, 121.2, 120.3 (q, *J*_C–F_ = 258.6 Hz), 117.5, (d, *J*_C–F_ = 20.2 Hz); HPLC purity: 11.5 min, 96.9%; HRMS (M + H)^+^ (ESI^+^) 394.9798 [M + H]^+^ (calcd for C_15_H_10_F_4_O_3_SeH^+^ 394.9731).

#### Preparation of (*E*)-1-Chloro-2-(styrylselenonyl)­benzene (**5t**)

Using Method E, **4t** (0.10 g, 0.35 mmol) and 70–75% mCPBA (0.18 g, 1.06 mmol) gave 0.022 g (19%) of **5t** as a white solid; *R*_*f*_ = 0.50 (*n*-hexane:EtOAc = 1:1); mp: 110.6–114.6 °C; ^1^H NMR (400 MHz, DMSO-*d*_6_) δ 8.24 (d, *J* = 15.3 Hz, 1H), 8.21 (dd, *J* = 7.9, 1.4 Hz, 1H), 7.87 (d, *J* = 15.4 Hz, 1H), 7.84–7.79 (m, 4H), 7.76–7.72 (m, 1H), 7.52–7.46 (m, 3H); ^13^C NMR (100 MHz, CDCl_3_) 146.3, 140.4, 135.3, 132.6, 132.0, 131.8, 131.6, 130.0, 129.3, 128.8, 128.1 127.7; HPLC purity: 15.4 min, 100.0%; HRMS (M + H)^+^ (ESI^+^) 326.9681 [M + H]^+^ (calcd for C_14_H_11_ClO_2_SeH^+^ 326.9613).

#### Preparation of (*E*)-1-Chloro-2-((2-methoxystyryl)­selenonyl)­benzene (**5u**)

Using Method E, **4u** (0.15 g, 0.46 mmol) and 70–75% mCPBA (0.33 g, 1.38 mmol) gave 0.051 g (31%) of **5u** as a white solid; *R*_*f*_ = 0.70 (100% EtOAc); mp: 160.6–163.6 °C; ^1^H NMR (400 MHz, CDCl_3_) δ 8.34–8.31 (m, 1H), 8.01 (d, *J* = 15.3 Hz, 1H), 7.73 (d, *J* = 15.3 Hz, 1H), 7.64–7.59 (m, 1H), 7.57–7.53 (m, 2H), 7.46–7.42 (m, 2H), 7.01 (td, *J* = 7.5, 0.8 Hz, 1H), 6.96 (d, *J* = 8.1 Hz, 1H), 3.92 (s, 3H); ^13^C NMR (100 MHz, CDCl_3_) 159.2, 142.4, 140.6, 135.0, 133.2, 132.7, 132.2, 131.7, 130.0, 129.0, 127.9, 121.0, 120.5, 111.3, 55.6; HPLC purity: 12.6 min, 97.8%; HRMS (M + H)^+^ (ESI^+^) 356.9785 [M + H]^+^ (calcd for C_15_H_13_ClO_3_SeH^+^ 356.9718).

#### Preparation of (*E*)-1-Chloro-2-((2-fluorostyryl)­selenonyl)­benzene (**5v**)

Using Method E, **4v** (0.090 g, 0.29 mmol) and 70–75% mCPBA (0.21 g, 0.87 mmol) gave 0.032 g (32%) of **5v** as a white solid; *R*_*f*_ = 0.80 (100% EtOAc); mp: 121.8–124.5 °C; ^1^H NMR (400 MHz, DMSO-*d*_6_) δ 8.27 (d, *J* = 15.4 Hz, 1H), 8.21 (dd, *J* = 7.8, 1.3 Hz, 1H), 7.97–7.93 (m, 1H), 7.91 (d, *J* = 15.3 Hz, 1H), 7.86–7.80 (m, 2H), 7.77–7.73 (m, 1H), 7.62–7.57 (m, 1H), 7.40–7.32 (m, 2H); ^13^C NMR (100 MHz, CDCl_3_) 161.7 (d, *J*_C–F_ = 254.9 Hz), 140.2, 139.7, 135.3, 133.5 (d, *J*_C–F_ = 8.9 Hz), 132.7, 131.8, 131.2 (d, *J*_C–F_ = 1.9 Hz), 131.0 (d, *J*_C–F_ = 10.1 Hz), 130.1, 128.1, 124.9 (d, *J*_C–F_ = 3.5 Hz), 119.9 (d, *J*_C–F_ = 11.1 Hz), 116.6 (d, *J*_C–F_ = 21.4 Hz); HPLC purity: 12.4 min, 99.3%; HRMS (M + H)^+^ (ESI^+^) 344.9582 [M + H]^+^ (calcd for C_14_H_10_ClFO_2_SeH^+^ 344.9519).

#### Preparation of (*E*)-1-Chloro-2-(2-((2-chlorophenyl)­selenonyl)­vinyl)­benzene (**5w**)

Using Method E, **4w** (0.71 g, 2.17 mmol) and 70–75% mCPBA (1.60 g, 6.50 mmol) gave 0.12 g (15%) of **5w** as a white solid; *R*_*f*_ = 0.28 (*n*-hexane:EtOAc = 1:1); mp: 135.7–138.5 °C; ^1^H NMR (400 MHz, DMSO-*d*_6_) δ 8.38 (d, *J* = 15.2 Hz, 1H), 8.23–8.21 (m, 1H), 8.13 (d, *J* = 15.2 Hz, 1H), 7.99 (dd, *J* = 7.8, 1.3 Hz, 1H), 7.87–7.81 (m, 2H), 7.78–7.74 (m, 1H), 7.63 (d, *J* = 8.0 Hz, 1H), 7.55 (td, *J* = 7.4, 1.4 Hz, 1H), 7.47 (t, *J* = 7.4 Hz, 1H); ^13^C NMR (100 MHz, CDCl_3_) 142.5, 140.2, 135.4, 135.4, 132.6, 131.8, 130.7, 130.6, 130.5, 130.1, 130.0, 128.8, 128.1, 127.4; HPLC purity: 13.3 min, 98.6%; HRMS (M + H)^+^ (ESI^+^) 360.9291 [M + H]^+^ (calcd for C_14_H_10_Cl_2_O_2_SeH^+^ 360.9223).

#### Preparation of (*E*)-1-Chloro-2-((2-(trifluoromethyl)­styryl)­selenonyl)­benzene (**5x**)

Using Method E, **4x** (0.13 g, 0.35 mmol) and 70–75% mCPBA (0.43 g, 1.05 mmol) gave 0.016 g (12%) of **5x** as a white solid; *R*_*f*_ = 0.80 (100% EtOAc); mp: 111.6–118.3 °C; ^1^H NMR (400 MHz, CDCl_3_) δ 8.35–8.27 (m, 2H), 7.76 (d, *J* = 7.2 Hz, 1H), 7.69–7.56 (m, 6H), 7.40 (d, *J* = 15.2 Hz, 1H); ^13^C NMR (100 MHz, CDCl_3_) 142.4 (d, *J*_C–F_ = 2.0 Hz), 139.9, 135.5, 132.7, 132.4, 132.2, 131.8, 131.3 (q, *J*_C–F_ = 73.0 Hz), 131.1, 130.5, 130.2, 128.6, 128.2, 126.6 (q, *J*_C–F_ = 5.7 Hz); HPLC purity: 13.6 min, 100.0%; HRMS (M + H)^+^ (ESI^+^) 394.9554 [M + H]^+^ (calcd for C_15_H_10_ClF_3_O_2_SeH^+^ 394.9487).

#### Preparation of (*E*)-1-Chloro-2-((2-(trifluoromethoxy)­styryl)­selenonyl)­benzene (**5y**)

Using Method E, **4y** (0.11 g, 0.29 mmol) and 70–75% mCPBA (0.22 g, 0.88 mmol) gave 0.02 g (20%) of **5y** as a white solid; *R*_*f*_ = 0.69 (100% EtOAc); mp: 111.7–112.8 °C; ^1^H NMR (400 MHz, CDCl_3_) δ 8.33 (d, *J* = 7.6 Hz, 1H), 8.08 (d, *J* = 15.5 Hz, 1H), 7.67–7.48 (m, 6H), 7.41–7.33 (m, 2H); ^13^C NMR (100 MHz, CDCl_3_) δ 148.0, 140.2, 139.8, 135.4, 133.1, 132.7, 131.8, 131.5, 130.1, 130.0, 128.2, 127.3, 124.8, 121.1, 120.3 (q, *J*_C–F_ = 258.4 Hz); HPLC purity: 9.5 min, 100.0%; HRMS (M + H)^+^ (ESI^+^) 410.9502 [M + H]^+^ (calcd for C_15_H_10_ClF_3_O_3_SeH^+^ 410.9436).

#### Preparation of (*E*)-1-Chloro-2-(2-((3-chlorophenyl)­selenonyl)­vinyl)­benzene (**5z**)

Using Method E, **4z** (0.022 g, 0.064 mmol) and 70–75% mCPBA (0.069, 0.32 mmol) gave 0.015 g (65%) of **5z** as a white solid; *R*_*f*_ = 0.80 (EtOAc:CH_3_OH = 10:1); mp: 141.6–142.3 °C; ^1^H NMR (400 MHz, DMSO-*d*_6_) δ 8.34 (d, *J* = 15.4 Hz, 1H), 8.10 (t, *J* = 5.9 Hz, 2H), 8.00 (d, *J* = 8.5 Hz, 1H), 7.92 (d, *J* = 7.6 Hz, 2H), 7.80 (t, *J* = 7.7 Hz, 1H), 7.63 (d, *J* = 7.8 Hz, 1H), 7.54 (t, *J* = 7.0 Hz, 1H), 7.46 (t, *J* = 8.0 Hz, 1H); ^13^C NMR (100 MHz, CDCl_3_) 143.2, 142.0, 136.6, 135.5, 134.4, 132.7, 131.4, 130.7, 130.1, 129.8, 128.8, 127.4, 127.1, 125.0; HPLC purity: 13.9 min, 100.0%; HRMS (M + H)^+^ (ESI^+^) 360.9291 [M + H]^+^ (calcd for C_14_H_10_Cl_2_O_2_SeH^+^ 360.9223).

#### Preparation of (*E*)-1-Chloro-2-(2-((4-chlorophenyl)­selenonyl)­vinyl)­benzene (**5aa**)

Using Method E, **4aa** (0.014 g, 0.041 mmol) and 70–75% mCPBA (0.044 g, 0.20 mmol) gave 0.0040 g (27%) of **5aa** as a white solid; *R*_*f*_ = 0.80 (EtOAc:CH_3_OH = 10:1); mp: 186.6–190.2 °C; ^1^H NMR (400 MHz, DMSO-*d*_6_) δ 8.32 (d, *J* = 15.2 Hz, 1H), 8.09–8.03 (m, 3H), 7.93 (d, *J* = 7.8 Hz, 1H), 7.85 (d, *J* = 8.3 Hz, 2H), 7.62 (d, *J* = 7.9 Hz, 1H), 7.5384 (t, *J* = 7.4 Hz, 1H), 7.46 (t, *J* = 7.7 Hz, 1H); ^13^C NMR (100 MHz, CDCl_3_) 141.8, 141.2, 135.5, 132.7, 131.6, 130.7, 130.6, 130.3, 129.8, 128.8, 128.4, 127.4; HPLC purity: 12.6 min, 97.0%; HRMS (M + H)^+^ (ESI^+^) 360.9289 [M + H]^+^ (calcd for C_14_H_10_Cl_2_O_2_SeH^+^ 360.9223).

#### Preparation of (*E*)-3-Fluoro-2-(2-((2-methoxyphenyl)­selenonyl)­vinyl)­pyridine (**7a**)

Using Method E, **6a** (0.047 g, 0.14 mmol) and 70–75% mCPBA (0.16 g, 0.72 mmol) gave 0.014 g (29%) of **7a** as a white solid; *R*_*f*_ = 0.24 (100% EtOAc); mp: 141.0–143.5 °C; ^1^H NMR (400 MHz, CDCl_3_) δ 8.48 (d, *J* = 4.4 Hz, 1H), 8.17–8.11 (m, 2H), 8.05 (d, *J* = 15.1 Hz, 1H), 7.66–7.62 (m, 1H), 7.52–7.47 (m, 1H), 7.41–7.37 (m, 1H), 7.22–7.18 (m, 1H), 7.09 (d, *J* = 8.3 Hz, 1H), 4.01 (s, 3H); ^13^C NMR (100 MHz, CDCl_3_) 158.4 (d, *J*_C–F_ = 264.3 Hz), 157.2, 146.0 (d, *J*_C–F_ = 5.08 Hz), 139.2 (d, *J*_C–F_ = 11.7 Hz), 136.1, 135.7, 135.4 (d, *J*_C–F_ = 4.6 Hz), 129.8, 129.0, 127.0 (d, *J*_C–F_ = 4.3 Hz), 124.2 (d, *J*_C–F_ = 18.9 Hz), 121.7, 112.8, 56.6; HPLC purity: 9.0 min, 98.4%; HRMS (M + H)^+^ (ESI^+^) 342.0040 [M + H]^+^ (calcd for C_14_H_12_FNO_3_SeH^+^ 341.9966).

#### Preparation of (*E*)-3-Chloro-2-(2-((2-methoxyphenyl)­selenonyl)­vinyl)­pyridine (**7b**)

Using Method E, **6b** (0.05 g, 0.15 mmol) and 70–75% mCPBA (0.45 g, 0.73 mmol) gave 0.02 g (30%) of **7b** as a white solid; *R*_*f*_ = 0.40 (100% EtOAc); mp: 149.5–151.0 °C; ^1^H NMR (400 MHz, CDCl_3_) δ 8.53 (dd, *J* = 4.5, 1.2 Hz, 1H), 8.34 (d, *J* = 14.8 Hz, 1H), 8.14–8.08 (m, 2H), 7.76 (dd, *J* = 8.2, 1.3 Hz, 1H), 7.66–7.62 (m, 1H), 7.31 (dd, *J* = 8.2, 4.5 Hz, 1H), 7.20 (t, *J* = 7.5 Hz, 1H), 7.08 (d, *J* = 8.3 Hz, 1H), 4.02 (s, 3H); ^13^C NMR (100 MHz, CDCl_3_) δ 157.3, 148.0, 147.8, 138.2, 138.1, 136.5, 136.2, 133.2, 129.7, 129.0, 126.2, 121.7, 112.8, 56.7; HPLC purity: 8.7 min, 100.0%; HRMS (M + H)^+^ (ESI^+^) 357.9737 [M + H]^+^ (calcd for C_14_H_12_ClNO_3_SeH^+^ 357.9671).

#### Preparation of (*E*)-2-(2-((2-Methoxyphenyl)­selenonyl)­vinyl)­3-(trifluoromethyl)­pyridine (**7c**)

Using Method E, **6c** (0.04 g, 0.11 mmol) and 70–75% mCPBA (0.26 g, 0.54 mmol) gave 0.01 g (28%) of **7c** as a yellow solid; *R*_*f*_ = 0.30 (100% EtOAc); mp: 104.3–105.6 °C; ^1^H NMR (400 MHz, CDCl_3_) δ 8.81 (d, *J* = 4.2 Hz, 1H), 8.25–8.09 (m, 3H), 8.05 (d, *J* = 7.8 Hz, 1H), 7.65 (t, *J* = 8.0 Hz, 1H), 7.48 (dd, *J* = 7.8, 4.8 Hz, 1H), 7.20 (t, *J* = 7.4 Hz, 1H), 7.09 (d, *J* = 8.3 Hz, 1H), 4.00 (s, 3H); ^13^C NMR (100 MHz, CDCl_3_) δ 157.3, 152.5, 148.5, 138.2, 137.9 (q, *J*_C–F_ = 2.7 Hz), 136.3, 134.5 (q, *J*_C–F_ = 5.1 Hz), 129.4, 129.2, 126.0 (q, *J*_C–F_ = 32.2 Hz), 124.6, 123.1 (q, *J*_C–F_ = 272.4 Hz), 121.7, 112.8, 56.6; HPLC purity: 9.5 min, 100.0%; HRMS (M + H)^+^ (ESI^+^) 392.0001 [M + H]^+^ (calcd for C_15_H_12_F_3_NO_3_SeH^+^ 391.9934).

#### Preparation of (*E*)-3-Fluoro-2-(2-((3-methoxyphenyl)­selenonyl)­vinyl)­pyridine (**7d**)

Using Method E, **6d** (0.089 g, 0.29 mmol) and 70–75% mCPBA (0.33 g, 1.45 mmol) gave 0.023 g (23%) of **7d** as a white solid; *R*_*f*_ = 0.65 (100% EtOAc); mp: 119.3–121.8 °C; ^1^H NMR (400 MHz, CDCl_3_) δ 8.46 (d, *J* = 4.4 Hz, 1H), 8.13 (d, *J* = 15.0 Hz, 1H), 7.83 (d, *J* = 15.0 Hz, 1H), 7.60–7.48 (m, 4H), 7.42–7.38 (m, 1H), 7.22 (d, *J* = 8.2 Hz, 1H), 3.90 (s, 3H); ^13^C NMR (100 MHz, CDCl_3_) δ 160.8, 158.5 (d, *J*_C–F_ = 265.2 Hz), 146.1 (d, *J*_C–F_ = 5.0 Hz), 142.6, 138.9 (d, *J*_C–F_ = 11.3 Hz), 136.1, 134.2 (d, *J*_C–F_ = 4.5 Hz), 131.2, 127.3 (d, *J*_C–F_ = 4.4 Hz), 124.4 (d, *J*_C–F_ = 18.9 Hz), 121.2, 119.0, 111.0, 55.9; HPLC purity: 10.8 min, 96.8%; HRMS (M + H)^+^ (ESI^+^) 342.0040 [M + H]^+^ (calcd for C_14_H_12_FNO_3_SeH^+^ 341.9966).

#### Preparation of (*E*)-3-Chloro-2-(2-((3-methoxyphenyl)­selenonyl)­vinyl)­pyridine (**7e**)

Using Method E, **6e** (0.036 g, 0.11 mmol) and 70–75% mCPBA (0.13 g, 0.55 mmol) gave 0.013 g (33%) of **7e** as a white solid; *R*_*f*_ = 0.63 (100% EtOAc); mp: 96.2–98.5 °C; ^1^H NMR (400 MHz, CDCl_3_) δ 8.51 (d, *J* = 3.2 Hz, 1H), 8.32 (d, *J* = 14.72 Hz, 1H), 7.88 (d, *J* = 14.68 Hz, 1H), 7.78 (dd, *J* = 8.1, 1.1 Hz, 1H), 7.62–7.50 (m, 3H), 7.32 (dd, *J* = 8.1, 4.5 Hz, 1H), 7.22 (d, *J* = 7.4 Hz, 1H), 3.90 (s, 3H); ^13^C NMR (100 MHz, CDCl_3_) δ 160.8, 148.1, 147.5, 142.6, 138.6, 138.1, 135.3, 133.3, 131.2, 126.4, 121.1, 119.0, 111.1, 56.0; HPLC purity: 12.1 min, 98.5%; HRMS (M + H)^+^ (ESI^+^) 357.9737 [M + H]^+^ (calcd for C_14_H_12_ClNO_3_SeH^+^ 357.9671).

#### Preparation of (*E*)-3-Fluoro-2-(2-((4-methoxyphenyl)­selenonyl)­vinyl)­pyridine (**7f**)

Using Method E, **6f** (0.060 g, 0.20 mmol) and 70–75% mCPBA (0.23 g, 1.00 mmol) gave 0.020 g (29%) of **7f** as a white solid; *R*_*f*_ = 0.74 (100% EtOAc); mp: 101.4–105.6 °C; ^1^H NMR (400 MHz, CDCl_3_) δ 8.45 (d, *J* = 4.2 Hz, 1H), 8.10 (d, *J* = 15.0 Hz, 1H), 7.95 (d, *J* = 11.1 Hz, 2H), 7.81 (d, *J* = 15.4 Hz, 1H), 7.50 (t, *J* = 9.0 Hz, 1H), 7.42–7.37 (m, 1H), 7.10 (d, *J* = 9.0 Hz, 2H), 3.90 (s, 3H); ^13^C NMR (100 MHz, CDCl_3_) δ 164.3, 158.4 (d, *J*_C–F_ = 265.0 Hz), 146.1 (d, *J*_C–F_ = 5.0 Hz), 138.0 (d, *J*_C–F_ = 11.3 Hz), 135.6, 134.6 (d, *J*_C–F_ = 4.6 Hz), 132.6, 129.0, 127.2 (d, *J*_C–F_ = 4.4 Hz), 124.4 (d, *J*_C–F_ = 19.0 Hz), 115.6, 55.9; HPLC purity: 10.4 min, 98.4%; HRMS (M + H)^+^ (ESI^+^) 342.0040 [M + H]^+^ (calcd for C_14_H_12_FNO_3_SeH^+^ 341.9966).

#### Preparation of (*E*)-3-Chloro-2-(2-((4-methoxyphenyl)­selenonyl)­vinyl)­pyridine (**7g**)

Using Method E, **6g** (0.06 g, 0.18 mmol) and 70–75% mCPBA (0.22 g, 0.35 mmol) gave 0.02 g (27%) of **7g** as a white solid; *R*_*f*_ = 0.62 (EtOAc:CH_3_OH = 19:1); mp: 158.2–159.5 °C; ^1^H NMR (400 MHz, CDCl_3_) δ 8.51 (dd, *J* = 4.5, 1.4 Hz, 1H), 8.29 (d, *J* = 14.8 Hz, 1H), 7.99–7.92 (m, 2H), 7.86 (d, *J* = 14.8 Hz, 1H), 7.77 (dd, *J* = 8.2, 1.4 Hz, 1H), 7.30 (dd, *J* = 8.2, 4.5 Hz, 1H), 7.13–7.07 (m, 2H), 3.90 (s, 3H); ^13^C NMR (100 MHz, CDCl_3_) δ 164.3, 148.0, 147.6, 138.1, 138.0, 135.7, 133.3, 132.6, 129.0, 126.3, 115.6, 55.9; HPLC purity: 9.4 min, 98.7%; HRMS (M + H)^+^ (ESI^+^) 357.9737 [M + H]^+^ (calcd for C_14_H_12_ClNO_3_SeH^+^ 357.9671).

#### Preparation of (*E*)-3-Fluoro-2-(2-((2-fluorophenyl)­selenonyl)­vinyl)­pyridine (**7h**)

Using Method E, **6h** (0.05 g, 0.16 mmol) and 70–75% mCPBA (0.49 g, 0.79 mmol) gave 0.02 g (34%) of **7h** as a white solid; *R*_*f*_ = 0.54 (100% EtOAc); mp: 137.4–138.5 °C; ^1^H NMR (400 MHz, CDCl_3_) δ 8.48 (d, *J* = 4.4 Hz, 1H), 8.21 (d, *J* = 15.0 Hz, 1H), 8.17–8.11 (m, 1H), 8.02 (dd, *J* = 15.0, 1.2 Hz, 1H), 7.75–7.68 (m, 1H), 7.55–7.40 (m, 3H), 7.34–7.28 (m, 1H); ^13^C NMR (100 MHz, CDCl_3_) δ 160.4 (d, *J*_C–F_ = 92.4 Hz), 157.8 (d, *J*_C–F_ = 104.5 Hz), 146.2 (d, *J*_C–F_ = 5.0 Hz), 138.8 (d, *J*_C–F_ = 11.1 Hz), 137.0, 136.7 (d, *J*_C–F_ = 7.9 Hz), 134.5 (d, *J*_C–F_ = 4.5 Hz), 129.2, 129.1 (d, *J*_C–F_ = 18.7 Hz), 127.5 (d, *J*_C–F_ = 4.5 Hz), 125.6 (d, *J*_C–F_ = 3.5 Hz), 124.4 (d, *J*_C–F_ = 18.8 Hz), 117.6 (d, *J*_C–F_ = 20.1 Hz); HPLC purity: 8.5 min, 100.0%; HRMS (M + H)^+^ (ESI^+^) 329.9837 [M + H]^+^ (calcd for C_13_H_9_F_2_NO_2_SeH^+^ 329.9767).

#### Preparation of (*E*)-3-Chloro-2-(2-((2-fluorophenyl)­selenonyl)­vinyl)­pyridine (**7i**)

Using Method E, **6i** (0.07 g, 0.20 mmol) and 70–75% mCPBA (0.63 g, 1.01 mmol) gave 0.02 g (35%) of **7i** as a white solid; *R*_*f*_ = 0.74 (100% EtOAc); mp: 122.8–124.6 °C; ^1^H NMR (400 MHz, CDCl_3_) δ 8.54 (dd, *J* = 4.5, 1.3 Hz, 1H), 8.39 (d, *J* = 14.7 Hz, 1H), 8.18–8.02 (m, 2H), 7.79 (dd, *J* = 8.2, 1.3 Hz, 1H), 7.75–7.68 (m, 1H), 7.48–7.42 (m, 1H), 7.37–7.27 (m, 2H); ^13^C NMR (100 MHz, CDCl_3_) δ 159.6 (d, *J*_C–F_ = 253.1 Hz), 148.1, 147.3, 139.4, 138.2, 136.7 (d, *J*_C–F_ = 7.9 Hz), 135.6, 133.5, 129.1, 129.0 (d, *J*_C–F_ = 18.6 Hz), 126.6, 125.6 (d, *J*_C–F_ = 3.5 Hz), 117.6 (d, *J*_C–F_ = 20.0 Hz); HPLC purity: 9.5 min, 100.0%; HRMS (M + H)^+^ (ESI^+^) 345.9534 [M + H]^+^ (calcd for C_13_H_9_ClFNO_2_SeH^+^ 345.9471).

#### Preparation of (*E*)-2-(2-((2-Fluorophenyl)­selenonyl)­vinyl)-3-(trifluoromethyl)­pyridine (**7j**)

Using Method E, **6j** (0.06 g, 0.16 mmol) and 70–75% mCPBA (0.08 g, 0.48 mmol) gave 0.03 g (43%) of **7j** as a white solid; *R*_*f*_ = 0.65 (100% EtOAc); mp: 112.8–113.7 °C; ^1^H NMR (400 MHz, CDCl_3_) δ 8.82 (d, *J* = 4.6 Hz, 1H), 8.27–8.11 (m, 3H), 8.07 (d, *J* = 8.0 Hz, 1H), 7.73 (q, *J* = 7.0 Hz, 1H), 7.52 (dd, *J* = 7.9, 4.8 Hz, 1H), 7.46 (t, *J* = 7.7 Hz, 1H), 7.31 (d, *J* = 8.7 Hz, 1H); ^13^C NMR (100 MHz, CDCl_3_) δ 159.6 (d, *J*_C–F_ = 253.1 Hz), 152.6, 147.9, 139.2, 137.2, 136.9 (d, *J*_C–F_ = 7.9 Hz), 134.6 (q, *J*_C–F_ = 20.1 Hz), 129.2, 128.9 (d, *J*_C–F_ = 18.5 Hz), 126.4 (q, *J*_C–F_ = 32.5 Hz), 125.7 (d, *J*_C–F_ = 3.3 Hz), 125.0, 123.1 (q, *J*_C–F_ = 272.4 Hz), 117.6 (d, *J*_C–F_ = 20.0 Hz); HPLC purity: 10.2 min, 100.0%; HRMS (M + H)^+^ (ESI^+^) 379.9800 [M + H]^+^ (calcd for C_14_H_9_F_4_NO_2_SeH^+^ 379.9735).

#### Preparation of (*E*)-3-Fluoro-2-(2-((3-fluorophenyl)­selenonyl)­vinyl)­pyridine (**7k**)

Using Method E, **6k** (0.06 g, 0.21 mmol) and 70–75% mCPBA (0.11 g, 0.64 mmol) gave 0.006 g (9%) of **7k** as a white solid; *R*_*f*_ = 0.61 (100% EtOAc); mp: 97.0–97.8 °C; ^1^H NMR (400 MHz, CDCl_3_) δ 8.47 (d, *J* = 4.4 Hz, 1H), 8.17 (d, *J* = 15.0 Hz, 1H), 7.92–7.73 (m, 3H), 7.70–7.62 (m, 1H), 7.56–7.48 (m, 1H), 7.45–7.38 (m, 2H); ^13^C NMR (100 MHz, CDCl_3_) δ 162.9 (d, *J*_C–F_ = 254.4 Hz), 158.6 (d, *J*_C–F_ = 265.3 Hz), 146.2 (d, *J*_C–F_ = 4.9 Hz), 143.2 (d, *J*_C–F_ = 6.4 Hz), 138.7 (d, *J*_C–F_ = 11.0 Hz), 136.8, 133.7 (d, *J*_C–F_ = 4.4 Hz), 132.0 (d, *J*_C–F_ = 7.3 Hz), 127.6 (d, *J*_C–F_ = 4.6 Hz), 124.5 (d, *J*_C–F_ = 18.9 Hz), 122.9 (d, *J*_C–F_ = 3.6 Hz), 121.7 (d, *J*_C–F_ = 21.1 Hz), 114.7 (d, *J*_C–F_ = 24.7 Hz); HPLC purity: 9.7 min, 95.3%; HRMS (M + H)^+^ (ESI^+^) 329.9838 [M + H]^+^ (calcd for C_13_H_9_F_2_NO_2_SeH^+^ 329.9767).

#### Preparation of (*E*)-3-Chloro-2-(2-((3-fluorophenyl)­selenonyl)­vinyl)­pyridine (**7l**)

Using Method E, **6l** (0.02 g, 0.06 mmol) and 70–75% mCPBA (0.03 g, 0.17 mmol) gave 0.003 g (15%) of **7l** as a white solid; *R*_*f*_ = 0.67 (100% EtOAc); mp: 103.3–104.1 °C; ^1^H NMR (400 MHz, CDCl_3_) δ 8.52 (d, *J* = 4.4 Hz, 1H), 8.35 (d, *J* = 14.7 Hz, 1H), 7.93–7.74 (m, 4H), 7.70–7.62 (m, 1H), 7.45–7.38 (m, 1H), 7.33 (dd, *J* = 8.3, 4.5 Hz, 1H); ^13^C NMR (100 MHz, CDCl_3_) δ 162.9 (d, *J*_C–F_ = 253.6 Hz), 148.1, 147.3, 143.2 (d, *J*_C–F_ = 5.9 Hz), 139.2, 138.2, 134.8, 133.5, 132.0 (d, *J*_C–F_ = 7.3 Hz), 126.6, 122.9 (d, *J*_C–F_ = 3.5 Hz), 121.7 (d, *J*_C–F_ = 21.4 Hz), 114.7 (d, *J*_C–F_ = 24.9 Hz); HPLC purity: 11.2 min, 98.6%; HRMS (M + H)^+^ (ESI^+^) 345.9535 [M + H]^+^ (calcd for C_13_H_9_ClFNO_2_SeH^+^ 345.9471).

#### Preparation of (*E*)-3-Fluoro-2-(2-((4-fluorophenyl)­selenonyl)­vinyl)­pyridine (**7m**)

Using Method E, **6m** (0.09 g, 0.31 mmol) and 70–75% mCPBA (0.16 g, 0.94 mmol) gave 0.002 g (2%) of **7m** as a white solid; *R*_*f*_ = 0.62 (100% EtOAc); mp: 129.4–130.1 °C; ^1^H NMR (400 MHz, CDCl_3_) δ 8.47 (d, *J* = 4.1 Hz, 1H), 8.21–8.00 (m, 3H), 7.82 (d, *J* = 14.8 Hz, 1H), 7.52 (t, *J* = 9.1 Hz, 1H), 7.45–7.38 (m, 1H), 7.34 (t, *J* = 7.8 Hz, 2H); ^13^C NMR (100 MHz, CDCl_3_) δ 166.2 (d, *J*_C–F_ = 255.7 Hz), 158.5 (d, *J*_C–F_ = 264.9 Hz), 146.2 (d, *J*_C–F_ = 5.1 Hz), 138.8 (d, *J*_C–F_ = 10.5 Hz), 137.2 (d, *J*_C–F_ = 3.0 Hz), 136.4, 134.0 (d, *J*_C–F_ = 4.7 Hz), 129.9 (d, *J*_C–F_ = 9.5 Hz), 127.5 (d, *J*_C–F_ = 4.4 Hz), 124.5 (d, *J*_C–F_ = 18.9 Hz), 117.8 (d, *J*_C–F_ = 22.7 Hz); HPLC purity: 9.2 min, 95.3%; HRMS (M + H)^+^ (ESI^+^) 329.9839 [M + H]^+^ (calcd for C_13_H_9_F_2_NO_2_SeH^+^ 329.9767).

#### Preparation of (*E*)-3-Chloro-2-(2-((4-fluorophenyl)­selenonyl)­vinyl)­pyridine (**7n**)

Using Method E, **6n** (0.06 g, 0.19 mmol) and 70–75% mCPBA (0.10 g, 0.56 mmol) gave 0.001 g (2%) of **7n** as a white solid; *R*_*f*_ = 0.70 (100% EtOAc); mp: 135.4–136.0 °C; ^1^H NMR (400 MHz, CDCl_3_) δ 8.47 (dd, *J* = 4.9, 1.2 Hz, 1H), 8.34 (d, *J* = 14.7 Hz, 1H), 8.07 (m, 2H), 7.87 (d, *J* = 14.7 Hz, 1H), 7.78 (dd, *J* = 8.4, 1.4 Hz, 1H), 7.37–7.30 (m, 3H); ^13^C NMR (100 MHz, CDCl_3_) δ 166.1 (d, *J*_C–F_ = 256.2 Hz), 148.1, 147.4, 138.9, 138.2, 137.3 (d, *J*_C–F_ = 2.3 Hz), 135.2, 133.4, 129.9 (d, *J*_C–F_ = 9.6 Hz), 126.5, 117.8 (d, *J*_C–F_ = 22.9 Hz); HPLC purity: 11.2 min, 95.2%; HRMS (M + H)^+^ (ESI^+^) 345.9538 [M + H]^+^ (calcd for C_13_H_9_ClFNO_2_SeH^+^ 345.9471).

#### Preparation of (*E*)-2-(2-((2-Chlorophenyl)­selenonyl)­vinyl)-3-fluoropyridine (**7o**)

Using Method E, **6o** (0.078 g, 0.24 mmol) and 70–75% mCPBA (0.26 g, 1.19 mmol) gave 0.021 g (25%) of **7o** as a white solid; *R*_*f*_ = 0.59 (100% EtOAc); mp: 125.5–129.5 °C; ^1^H NMR (400 MHz, CDCl_3_) δ 8.49 (d, *J* = 4.4 Hz, 1H), 8.34–8.31 (m, 1H), 8.20 (d, *J* = 15.1 Hz, 1H), 8.09 (d, *J* = 15.0 Hz, 1H), 7.67–7.63 (m, 1H), 7.59–7.49 (m, 3H), 7.45–7.40 (m, 1H); ^13^C NMR (100 MHz, CDCl_3_) 158.5 (d, *J*_C–F_ = 265.2 Hz), 146.2 (d, *J*_C–F_ = 5.0 Hz), 140.1, 138.8 (d, *J*_C–F_ = 11.0 Hz), 137.2, 135.4, 134.3 (d, *J*_C–F_ = 4.5 Hz), 132.8, 131.8, 130.1, 128.1, 127.4 (d, *J*_C–F_ = 4.3 Hz), 124.4 (d, *J*_C–F_ = 18.9 Hz); HPLC purity: 10.3 min, 98.3%; HRMS (M + H)^+^ (ESI^+^) 345.9536 [M + H]^+^ (calcd for C_13_H_9_ClFNO_2_SeH^+^ 345.9471).

#### Preparation of (*E*)-3-Chloro-2-(2-((2-chlorophenyl)­selenonyl)­vinyl)­pyridine (**7p**)

Using Method E, **6p** (0.021 g, 0.060 mmol) and 70–75% mCPBA (0.027 g, 0.12 mmol) gave 0.013 g (60%) of **7p** as a white solid; *R*_*f*_ = 0.69 (100% EtOAc); mp: 149.2–152.4 °C; ^1^H NMR (400 MHz, CDCl_3_) δ 8.54 (dd, *J* = 4.5, 1.4 Hz, 1H), 8.39 (d, *J* = 14.7 Hz, 1H), 8.33 (dd, *J* = 8.1, 1.8 Hz, 1H), 8.13 (d, *J* = 14.7 Hz, 1H), 7.77 (dd, *J* = 8.2, 1.5 Hz, 1H), 7.64–7.62 (m, 1H), 7.59–7.55 (m, 2H), 7.32 (dd, *J* = 8.2, 4.5 Hz, 1H); ^13^C NMR (100 MHz, CDCl_3_) 148.1, 147.4, 140.1, 139.6, 138.1, 135.4, 135.4, 133.4, 132.8, 131.8, 130.1, 128.1, 126.4; HPLC purity: 11.4 min, 99.6%; HRMS (M + H)^+^ (ESI^+^) 361.9242 [M + H]^+^ (calcd for C_13_H_9_Cl_2_NO_2_SeH^+^ 361.9176).

#### Preparation of (*E*)-2-(2-((2-Chlorophenyl)­selenonyl)­vinyl)-3-(trifluoromethyl)­pyridine (**7q**)

Using Method E, **6q** (0.095 g, 0.25 mmol) and 70–75% mCPBA (0.25 g, 1.25 mmol) gave 0.020 g (20%) of **7q** as a white solid; *R*_*f*_ = 0.71 (100% EtOAc); mp: 111.7–112.3 °C; ^1^H NMR (400 MHz, CDCl_3_) δ 8.82 (d, *J* = 4.1 Hz, 1H), 8.35–8.32 (m, 1H), 8.23 (s, 2H), 8.06 (d, *J* = 8.0 Hz, 1H), 7.65–7.63 (m, 1H), 7.60–7.56 (m, 2H), 7.50 (dd, *J* = 7.9, 4.8 Hz, 1H); ^13^C NMR (100 MHz, CDCl_3_) 152.5, 139.8, 139.4 (q, *J*_C–F_ = 2.5 Hz), 137.0, 135.5, 134.5 (q, *J*_C–F_ = 5.0 Hz), 132.9, 131.8, 130.2, 128.1, 126.3 (q, *J*_C–F_ = 32.4 Hz), 124.8, 123.0 (q, *J*_C–F_ = 272.2 Hz); HPLC purity: 12.5 min, 97.5%; HRMS (M + H)^+^ (ESI^+^) 395.9505 [M + H]^+^ (calcd for C_14_H_9_ClF_3_NO_2_SeH^+^ 395.9439).

#### Preparation of (*E*)-2-(2-((3-Chlorophenyl)­selenonyl)­vinyl)-3-fluoropyridine (**7r**)

Using Method E, **6r** (0.037 g, 0.11 mmol) and 70–75% mCPBA (0.12 g, 0.56 mmol) gave 0.035 g (92%) of **7r** as a white solid; *R*_*f*_ = 0.75 (EtOAc:CH_3_OH = 10:1); mp: 112.2–116.6 °C; ^1^H NMR (400 MHz, DMSO-*d*_6_) δ 8.56 (d, *J* = 4.2 Hz, 1H), 8.40 (d, *J* = 15.1 Hz, 1H), 8.16 (s, 1H), 8.04 (d, *J* = 7.9 Hz, 1H), 7.97–7.90 (m, 3H), 7.78 (t, *J* = 8.0 Hz, 1H), 7.65 (quint, *J* = 4.2 Hz, 1H); ^13^C NMR (100 MHz, DMSO-*d*_6_) δ 159.9, 157.4, 146.9 (d, *J*_C–F_ = 4.8 Hz), 143.7, 138.6 (d, *J*_C–F_ = 10.8 Hz), 136.6, 135.4, 134.9, 132.8, 128.9 (d, *J*_C–F_ = 4.8 Hz), 127.1, 126.0, 125.6, 125.4; HPLC purity: 11.3 min, 99.3%; HRMS (M + H)^+^ (ESI^+^) 345.9537 [M + H]^+^ (calcd for C_13_H_9_ClFNO_2_SeH^+^ 345.9471).

#### Preparation of (*E*)-3-Chloro-2-(2-((3-chlorophenyl)­selenonyl)­vinyl)­pyridine (**7s**)

Using Method E, **6s** (0.052 g, 0.15 mmol) and 70–75% mCPBA (0.16 g, 0.75 mmol) gave 0.028 g (52%) of **7s** as a white solid; *R*_*f*_ = 0.79 (EtOAc:CH_3_OH = 10:1); mp: 135.1–138.3 °C; ^1^H NMR (400 MHz, DMSO-*d*_6_) δ 8.65 (d, *J* = 4.3 Hz, 1H), 8.46 (d, *J* = 14.8 Hz, 1H), 8.18–8.10 (m, 3H), 8.04 (d, *J* = 7.9 Hz, 1H), 7.91 (d, *J* = 6.0 Hz, 1H), 7.79 (t, *J* = 7.9 Hz, 1H), 7.58 (dd, *J* = 8.1, 4.6 Hz, 1H); ^13^C NMR (100 MHz, DMSO-*d*_6_) δ 149.2, 147.1, 143.6, 139.0, 138.7, 136.2, 135.5, 134.9, 132.9, 132.8, 127.9, 127.1, 126.0; HPLC purity: 12.8 min, 100.0%; HRMS (M + H)^+^ (ESI^+^) 361.9243 [M + H]^+^ (calcd for C_13_H_9_Cl_2_NO_2_SeH^+^ 361.9176).

#### Preparation of (*E*)-2-(2-((4-Chlorophenyl)­selenonyl)­vinyl)-3-fluoropyridine (**7t**)

Using Method E, **6t** (0.042 g, 0.13 mmol) and 70–75% mCPBA (0.15 g, 0.64 mmol) gave 0.050 g (100%) of **7t** as a white solid; *R*_*f*_ = 0.80 (EtOAc:CH_3_OH = 10:1); mp: 134.3–136.8 °C; ^1^H NMR (400 MHz, DMSO-*d*_6_) δ 8.56 (d, *J* = 4.5 Hz, 1H), 8.36 (d, *J* = 15.1 Hz, 1H), 8.09 (d, *J* = 8.6 Hz, 2H), 7.95–7.89 (m, 2H), 7.86–7.83 (m, 2H), 7.65 (quint, *J* = 4.4 Hz, 1H); ^13^C NMR (100 MHz, DMSO-*d*_6_) δ 146.9 (d, *J*_C–F_ = 5.0 Hz), 140.9, 140.0, 139.1 (d, *J*_C–F_ = 10.8 Hz), 136.3, 135.1 (d, *J*_C–F_ = 4.4 Hz), 131.0, 129.3, 128.9 (d, *J*_C–F_ = 3.7 Hz), 125.6, 125.4; HPLC purity: 11.1 min, 100.0%; HRMS (M + H)^+^ (ESI^+^) 345.9532 [M + H]^+^ (calcd for C_13_H_9_ClFNO_2_SeH^+^ 345.9471).

#### Preparation of (*E*)-3-Chloro-2-(2-((4-chlorophenyl)­selenonyl)­vinyl)­pyridine (**7u**)

Using Method E, **6u** (0.072 g, 0.21 mmol) and 70–75% mCPBA (0.23 g, 1.04 mmol) gave 0.041 g (54%) of **7u** as a white solid; *R*_*f*_ = 0.83 (EtOAc:CH_3_OH = 10:1); mp: 146.9–148.8 °C; ^1^H NMR (400 MHz, DMSO-*d*_6_) δ 8.64 (dd, *J* = 4.5, 1.1 Hz, 1H), 8.42 (d, *J* = 14.8 Hz, 1H), 8.16–8.08 (m, 4H), 7.86–7.83 (m, 2H), 7.57 (dd, *J* = 8.2, 4.5 Hz, 1H); ^13^C NMR (100 MHz, DMSO-*d*_6_) δ 146; HPLC purity: 12.2 min, 100.0%; HRMS (M + H)^+^ (ESI^+^) 361.9242 [M + H]^+^ (calcd for C_13_H_9_Cl_2_NO_2_SeH^+^ 361.9176).

### Cell Culture

HaCaT keratinocytes (human keratinocyte) and Raw264.7 macrophages (mouse macrophage) were cultured in Dulbecco’s modified Eagle’s medium (DMEM) (Biowest, Nuaillé, France) supplemented with 10% (v/v) fetal bovine serum (Biowest) and 100 U/mL penicillin-streptomycin (Gibco) at 37 °C in a 5% CO_2_ humidified incubator. To investigate Keap1-Nrf2 nuclear translocation, the PathHunter U2OS Keap1-Nrf2 nuclear translocation cell line (93-0821C3, DiscoverX, Fremont, CA, USA) was cultured in AssayComplete cell culture medium (92-3103G, DiscoverX) at 37 °C in a 5% CO_2_ humidified incubator.

### Microsomal Stability Test

Xenotech Xtreme 200 human liver microsomes (HLM) (66103, XenoTech, Kansas City, KS, USA) were used at a concentration of 0.5 mg/mL and preincubated in 0.1 M phosphate buffer (pH 7.4) at 37 °C for 5 min. The reaction was initiated by the addition of NADPH regeneration buffer (containing NADP^+^, MgCl_2_, glucose-6-phosphate, and glucose-6-phosphate dehydrogenase) and the test compounds (1 μM), and terminated after 30 min at 37 °C by adding carbamazepine in acetonitrile. Precipitated proteins were removed by centrifugation (12,000*g*, 5 min, 4 °C), and the supernatant was analyzed using a Shimadzu LCMS-2020 system. Chromatographic separation was performed on an ACE Excel 2 C18-AR column (100 × 2.1 mm, 2.0 μm particle size; ACE) with a mobile phase consisting of distilled water (A) with 0.1% formic acid and acetonitrile (B) with 0.1% formic acid. Data acquisition and analysis were performed using LabSolutions software (version 5.113). The percentage of compound remaining was determined by comparing peak areas. Data are expressed as the mean of duplicate determinations (*n* = 2).

### Skin-PAMPA Assay

The skin-PAMPA assay for **5w** was conducted by KMEDIhub, Daegu-Gyeongbuk Medical Center (Daegu, Korea). PRISMA HT buffer (pH 7.4) was prepared in distilled water and used as donor and acceptor buffers, and the test compound was dissolved in donor buffer. Acceptor plate membranes were hydrated overnight (15–18 h). On the assay day, donor solutions (200 μL) and acceptor buffer (200 μL) were added to the donor and acceptor plates, respectively, and assembled into a Stirwell PAMPA sandwich. Plates were incubated at 25 °C for 5 h. Following incubation, 150 μL from both donor and acceptor wells was transferred to a UV plate, and absorbance was measured at 250–500 nm (4 nm intervals) using a SYNERGY H1 reader. Permeability coefficients (*P*_e_, cm/s) were calculated using PAMPA Explorer (v3.8, Pion Inc.) with a detection threshold of 0.015. Results are expressed as the mean ± SD of triplicates (*n* = 3).

### Keap1-Nrf2 Nuclear Translocation Assay

The Nrf2 nuclear translocation activity of the synthesized compounds was evaluated using the PathHunter eXpress Keap1-Nrf2 Nuclear Translocation Assay Kit (93-0821E3CP0L, DiscoverX) following the manufacturer’s instructions. PathHunter U2OS cells were genetically engineered to coexpress Nrf2 tagged with an enzyme donor and a nuclear-localized enzyme acceptor. Upon Nrf2 activation, nuclear translocation facilitates the proximity of the two enzyme fragments, resulting in their complementation and the production of functional β-galactosidase, which is detected by a chemi luminescence. For the assay, engineered U2OS cells were seeded into 96-well white plates and treated with various concentrations of the test compound for 6 h at room temperature (20–23 °C). Cells were then incubated for 1 h in the dark with the detection reagent, and chemiluminescent signals were recorded at all wavelengths using a microplate reader (SpectraMax i3, Molecular Devices, San Jose, CA, USA). EC_50_ values were calculated from concentration–response curves using SigmaPlot version 13.0 and expressed as the mean ± SEM from triplicate measurements.

### Cytotoxicity Assay

The cytotoxicity of **5w** was assessed using the EZ-Cytox assay kit (DoGenBio, Seoul, South Korea).^70^ HaCaT keratinocytes were seeded in clear 96-well plates and incubated with various concentrations of **5w** for 24 h at 37 °C under 5% CO_2_. Following an incubation with EZ-Cytox reagent, cell viability was measured by detecting the absorbance of orange formazan dye produced via WST-8 reduction by mitochondrial dehydrogenases in living cells. The absorbance at 450 nm was detected using a SpectraMax i3 microplate reader (Molecular Device).

### Western Blotting

For whole cell lysates, HaCaT keratinocytes or Raw264.7 macrophages were washed with ice-cold phosphate buffered saline (PBS) and lysed in RIPA lysis buffer (Sigma-Aldrich, St. Louis, MO, USA) containing protease inhibitor cocktail (Roche Diagnostics Corp., Indianapolis, IN, USA), for 40 min on ice. Following centrifugation at 15,800*g* for 20 min, the supernatants representing whole cell lysate were collected. Nuclear/cytosol fractionation was conducted according to a protocol previously established.^41^ In parallel, mouse dorsal skin tissue samples were homogenized in RIPA lysis buffer containing protease inhibitors using a bead ruptor (OMNI International, Kennesaw, GA, USA), and the resulting homogenates were centrifuged to extract total protein. Protein concentrations were quantified using the Pierce BCA Protein Assay Kit (Thermo Fisher Scientific), and 10 μg of each sample was separated by polyacrylamide gel eletrophoresis. The proteins were subsequently transferred onto a polyvinylidene fluoride (PVDF) membrane (Millipore, Burlington, MA, USA). The membranes were blocked in 5% skim milk or 4% BSA in TBST (10 mM Tris-HCl, pH 7.5, 150 mM NaCl, and 0.1% Tween 20) for 1 h at room temperature and incubated with primary antibodies at 4 °C overnight. The antibodies used were as follows; Nrf2 (Abcam, Cambridge, UK, 1:500), HO-1 (Enzo Life Science, Ann Arbor, MI, USA, 1:2000), GCLM (Santa Cruz Biotechnology, Santa Cruz, Dallas, TX, USA, 1:1000), IL-1β (R&D systems, McKinley Place, MN, USA, 1:500), iNOS (Abcam, Cambridge, UK, 1:1000), IL-6 (Santa Cruz Biotechnology, 1:1000), Loricrin (Abclonal, Woburn, MA, USA, 1:1000), Lamin B1 (Bioworld Technology, St. Louis Park, MN, USA, 1:3000), or β-Actin (Santa Cruz Biotechnology, 1:5000) in 4% BSA in TBST, with shaking. After washing with TBST, blots were were incubated with antimouse, antirabbit, or antigoat horseradish peroxidase-conjugated IgG (GeneTex, 1:10000) for 1 h at room temperature. The protein bands were developed using SuperLumia ECL HRP Substrate solution (Abbkine, Atlanta, GA, USA) and detected using the Amersham Imager 600 (GE Healthcare, Arlington Heights, IL, USA). All Western blot experiments were independently repeated at least twice. Protein band intensities were quantified with ImageJ software (NIH, Bethesda, MD, USA), and relative values were normalized to Lamin B1 or β-Actin.

### Quantitative Real-time Reverse-transcription PCR (qRT-PCR)

Total RNA was extracted from HaCaT keratinocytes or Raw264.7 macrophages using TRIzol reagent (Invitrogen, Thermo Fisher Scientific, Carlsbad, CA, USA) following the manufacturer’s instructions.^70^ After removing genomic DNA, complementary DNA (cDNA) was synthesized from 1000 ng of total RNA with the iScript gDNA Clear cDNA Synthesis Kit (Bio-Rad, Hercules, CA, USA). Quantitative real-time PCR was carried out with the CFX Connect Real-Time PCR Detection System (Bio-Rad) using iQ SYBR Green Supermix (Bio-Rad). All qRT-PCR experiments were independently repeated twice. Normalization of relative gene expression was performed using ACTB as the reference gene for human samples and Hprt for mouse samples. The following primer sequences were used: *HMOX1* (Forward: 5′-TCTACCGCTCCCGCATGAAC-3′, Reverse: 5′-GGGCTCTGGTCCTTGGTGTC-3′), *GCLM* (Forward: 5′-TGCCTCCTGCTGTGTGATGC-3′, Reverse: 5′-ACTCGTGCGCTTGAATGTCAG-3′), *GCLC* (Forward: 5′-GGTGTTTGTGGTACTGCTCACC-3′, Reverse: 5′-TTCCCTGCAAGACAGCATCTC-3′), *NQO1* (Forward: 5′-AGAAAGGATGGGAGGTGGTGG-3′, Reverse: 5′-AAGCCAGAACAGACTCGGCAG-3′), *IL6* (Forward: 5′-AGTCCTGATCCAGTTCCTGC-3′, Reverse: 5′-AGATGAGTTGTCATGTCCTGC-3′), *TNF* (Forward: 5′-TGCTGCACTTTGGAGTGATC-3′, Reverse: 5′-CAGCTTGAGGGTTTGCTACA-3′), *CCL17* (Forward: 5′-TGCAGCACATCCACGCAG-3′, Reverse: 5′-CCCTGGAGCAGTCCTCAGATG-3′), *CCL22* (Forward: 5′-TGCACTCCTGGTTGTCCTCG-3′, Reverse: 5′-GTAATCACGGCAGCAGACGC-3′), (Forward: 5′-AGACCTCAACAGAGCCCTCA-3′, *ACTB* (Forward: 5′-TGGCACCCAGCACAATGAAG-3′, Reverse: 5′-CTGGAAGGTGGACAGCGAG-3′), *Nos2* Reverse: 5′-TCGAAGGTGAGCTGAACGAG-3′), *Il6* (Forward: 5′-ACAACGATGATGCACTTGCAGA-3′, Reverse: 5′-GGTACTCCAGAAGACCAGAGGAAA-3′), *Il1b* (Forward: 5′-ACTCAACTGTGAAATGCCACCT-3′, Reverse: 5′-ATGTGCTGCTGCGAGATTTG-3′), *Tnf* (Forward: 5′-CTCTTCTCATTCCTGCTTGTGGC-3′, Reverse: 5′-GAGAGGGAGGCCATTTGGGA-3′), *Ccl17* (Forward: 5′-CTGGCTGCTCTGCTTCTGGG-3′, Reverse: 5′-TGGCATCCCTGGAACACTCC-3′), *Ccl22* (Forward: 5′-TGGTGGCTCTCGTCCTTCTTG-3′, Reverse: 5′-ATGGCAGAGGGTGACGGATG-3′), *Tslp* (Forward: 5′-CGAGCAAATCGAGGACTGTGAG-3′, Reverse: 5′-TGAGGGCTTCTCTTGTTCTCCG-3′), and *Hprt* (Forward: 5′-CAGGAGAGAAAGATGTGATTGATA-3′, Reverse: 5′-GCCAACACTGCTGAAACA-3′).

### Enzyme-Linked Immunosorbent Assay (ELISA)

The concentration of pro-inflammatory cytokines secreted from HaCaT keratinocytes or Raw264.7 macrophages and IgE from mouse plasma was detected using ELISA kits for mouse IL-6 (Invitrogen, 88-7064-88), IgE (Invitrogen, 88-50460-88) and human IL-6 (BD Bioscience, San Jose, CA, USA, 555220) according to the manufacturer’s instructions. HaCaT keratinocytes were exposed to various concentrations of **5w** or SFN for 3 h, and subsequently stimulated with 10 ng/mL TNF-α+IFN-γ (Peprotech, Rocky hill, NJ, USA) stimulation for 24 h. Raw264.7 macrophages were pretreated with **5w** or SFN at indicated concentrations for 3 h and then exposed to 0.2 μg/mL LPS (Sigma-Aldrich) for 24 h. The conditioned medium was collected for analysis. Plasma samples were prepared by centrifuging blood collected from the heart at 848 *g* for 15 min at 4 °C. Absorbance at 450 nm was measured using a SpectraMax *i*3 microplate reader (Molecular Devices). All ELISA experiments were independently repeated twice.

### Griess Assay

The concentration of nitric oxide (NO) in the culture medium was measured using the Griess assay.^70^ Raw264.7 macrophages were pretreated with various concentrations of **5w** or SFN for 3 h, followed by 0.2 μg/mL LPS (Sigma-Aldrich) stimulation for 24 h. The conditioned medium was collected and incubated with equal volume of sulfanilamide solution (1% sulfanilamide in 5% phosphoric acid) for 5 min at room temperature (20–23 °C) in the dark. Subsequently, NED solution (0.1% *N*-1-naphthylethylenediamine dihydrochloride in deionized water) was added and incubated for an additional 5 min under the same conditions. Nitrite levels were quantified by measuring the absorbance at 540 nm using a SpectraMax *i*3 microplate reader (Molecular Devices) and comparing the values to a sodium nitrite standard curve. Griess assay experiments were independently repeated twice.

### Intracellular ROS Fluorescence Imaging

Intracellular ROS accumulation was assessed using 2′,7′-dichlorodihydrofluorescein diacetate (DCFH-DA, Sigma-Aldrich), a nonfluorescent probe that permeates cell membranes and is hydrolyzed by intracellular esterases to DCFH.^70^ Upon oxidation by ROS, DCFH is converted to fluorescent 2′,7′-dichlorofluorescein (DCF). Raw264.7 macrophages were incubated with 0.1 μM **5w** or 1 μM SFN for 9 h at at 37 °C. The cells were loaded with 80 μM DCFH-DA for 40 min, followed by treatment with 300 μM H_2_O_2_ to the culture medium for an additional 20 min at 37 °C. After washing with phosphate-buffered saline (PBS), fluorescence images representing intracellular ROS levels were acquired based on DCF fluorescence, along with corresponding differential interference contrast (DIC) images to visualize cell morphology, using an ImageXpress Pico (Molecular Devices). Fluorescence intensity was quantified using ImageJ software (NIH).

### DNCB-Induced Atopic Dermatitis-like Model

6-week-old male BALB/c mice were purchased from Samtako (Osan, South Korea) and housed in a temperature- and humidity-controlled environment (22 ± 1 °C, 12 h light-dark cycle) with *ad libitum* access to food and water. Animal experiments were conducted in accordance with the guidelines of the Animal Care and Use Committee of the Institutional Animal Care and Use Committee of KIST (Seoul, South Korea), (KIST-IACUC, approval number KIST-IACUC-2025-009). After 7 days of acclimation to their surroundings, mice were divided into five groups (*n* = 5); a vehicle group, a DNCB group as a negative control, a **5w** (0.1 or 0.5%) with DNCB group, and a dexamethasone (0.1%) with DNCB group as a positive control. On the day prior to the experiment (Day 0), the dorsal skin of each mouse was shaved to remove hair. In order to establish atopic dermatitis-like skin lesions, mice were sensitized with 200 μL of 1% 2,4-dinitrochlorobenzene (DNCB; dissolved in acetone:olive oil, 3:1 v/v) topically applied twice over the first week (Days 1–7). In the subsequent week (Days 8–14), 100 μL of 0.4% DNCB was topically administered every other day as a challenge phase. On each challenge day, **5w** was administered topically using a vehicle composed of 49% super refined polyethylene glycol 400 (Sigma-aldrich), 19.9% ethanol (Fisher scientific, Waltham, MA, USA), 15% isopropyl alcohol (Sigma-aldrich), 15 or 15.4% diethylene glycol monoethyl ether (Sigma-aldrich), 0.5% cyclomethicone (Sigma-aldrich), and 0.1% butylated hydroxytoluene (Sigma-aldrich). The pH of the vehicle solution is adjusted by anhydrous citric acid (Sigma-aldrich) and sodium hydroxide (Sigma-aldrich). To allow sufficient absorption and minimize interference, DNCB was applied first, followed by the test compound (or its vehicle control) 1 h later on the same site. Control mice received the corresponding vehicles on an identical schedule. Body weights were recorded at each administration to monitor general health status. Scratching behavior was recorded on the day before sacrifice, and dermatitis scores were evaluated immediately prior to sacrifice. Mice were deeply anesthetized with 2% Avertin (500 mg/kg, *i.p*.) and samples including cardiac blood, lymph nodes, spleen and dorsal skin tissues were collected.

### Scratching Behavior

To quantify pruritus symptom, scratching behavior with the hind paws was measured for 30 min on the day before sacrifice using recorded video. Scratching episodes were independently counted by two observers (J.K. and J.P.), and the results were expressed as the average of the two measurements.

### Dermatitis Scoring

The dermatitis severity score was measured according to five criteria—erythema, edema, excoriation, dryness, and lichenification—following established protocols in murine AD models with slight modifications.^7,63–65^ Each parameters was graded from 0 to 3 (0 = none, 1 = mild, 2 = moderate, 3 = severe), resulting in a maximum total score of 15.

### Histopathological Analysis

Collected dorsal skin tissues were fixed in 4% paraformaldehyde and embedded in paraffin. The paraffin-embedded skin tissues were cut at thickness of 3 μm. For hematoxylin-eosin (H&E) staining, tissue sections were deparaffinized in xylene and rehydrated through a descending series of ethyl alcohol concentrations into distilled water. The sections were then incubated in hematoxylin solution (TissuePro technology, Gainesville, FL, USA) for 5 min. After staining, sections were rinsed with running tap water, and subsequently differentiated in 0.3% acid alcohol to remove nonspecific hematoxylin stain from tissue and glass. Sections were counterstained with eosin Y solution (TissuePro technology) for 1 min. Finally, sections were dehydrated through an ascending ethanol series and cleared in xylene. Stained sections were mounted on slides with mounting medium (Dako, Agilent Technologies, Santa clara, CA, USA) and visualized with the ImageXpress Pico (Molecular Devices). The epidermal thickness was assessed by ImageJ software (NIH)^71^ and the eosinophils were counted in defined regions of interests (ROIs) within the skin images, and the results were expressed as the number of cells per site.

For toluidine blue staining, skin tissue sections were dewaxed in xylene and rehydrated in the descending ethanol series. After rehydrating in distilled water, sections were stained with toluidine blue working solution (0.1% toluidine blue O (Sigma-Aldrich) in 70% ethanol, diluted in 1% NaCl, pH 2.3) for 2 min and washed in running water. Stained sections were dehydrated in ascending ethanol series and then cleared in xylene. Following mounting with mounting medium (Dako), images were captured using ImageXpress Pico (Molecular Devices). The number of mast cells were counted manually in predetermined sites of equal area across groups, and the data were presented as cells per site.

### Statistical Analysis

All statistical analyses were conducted with GraphPad Prism 7 software (GraphPad, San Diego, CA, USA). Results are presented as the mean ± SEM. Multiple comparisons were performed with one-way ANOVA followed by either *Dunnett*’s or *Tukey*’s test. The statistical significance was asterisked as follows: *p < 0.05, **p < 0.01, ***p < 0.001, ****p < 0.0001.

## Supplementary Material





## Notes

Synthetic data for the intermediates **1**, **2**, **3**, **4**, and **6**; supplemental methods; ^1^H and ^13^C NMR spectra, HPLC analysis, and HR-MS data of the final compounds **5** and **7** (PDF)
Molecular formula strings (CSV)

## Abbreviations Used

ARE: antioxidant response element
AhR: aryl hydrocarbon receptor
AD: atopic dermatitis
cLogP: calculated partition coefficient
DNCB: 2,4-dinitrochlorobenzene
ELISA: enzyme-linked immunosorbent assay
GCLC: glutamate-cysteine ligase catalytic subunit
GCLM: glutamate-cysteine ligase modifier subunit
H&E: hematoxylin and eosin
HO-1: heme oxygenase-1
TNF-α: tumor necrosis factor-α
IFN-γ: interferon-γ
NF- κB: nuclear factor-κB
STAT1: signal transducer and activator of transcription 1
H_2_O_2_: hydrogen peroxide
IgE: immunoglobulin E
iNOS: inducible nitric oxide synthase
Keap1: Kelch-like ECH-associated protein 1
Nrf2: nuclear factor erythroid 2-related factor 2
NQO1: NAD­(P)­H quinone oxidoreductase 1
RNS: reactive nitrogen species
ROS: reactive oxygen species
Skin-PAMPA: skin parallel artificial membrane permeation assay
sMAF: small Maf proteins
SAR: structure–activity relationships
SFN: sulforaphane
Th1: T helper 1
Th2: T helper 2
TSLP: thymic stromal lymphopoietin
tPSA: topological polar surface area
