# Supplementary material for: Design, Synthesis, and Biological Evaluation of Novel Vinyl Selenone Derivatives as Potent Nrf2 Activators for Atopic Dermatitis
Source: J Med Chem. 2026 Mar 10;69(7):7572–93. doi: 10.1021/acs.jmedchem.5c02838 (PMC13071876; doi:10.1021/acs.jmedchem.5c02838)
Supplement: Supplementary file 1 [file jm5c02838_si_001.pdf]

Supporting Information

Design, Synthesis, and Biological Evaluation of Novel Vinyl Selenone Derivatives as Potent Nrf2 Activators for Atopic Dermatitis

Jushin Kim[1,2,#], Yoowon Kim[1,#], Byungeun Kim[1,#], Elijah Hwejin Lee[1,3], Rium Kim[1,3], Jiwoo Park[1], Jaehwan Kim[1,3], Yonghan Kim[1,3], Sang In Park[1,3], Minsik Kang[4], Jaeick Lee[4], Hyeon Jeong Kim[1], Jong-Hyun Park[1,3,*], Ji Won Choi[1,*], Dong-Woo Lee[2,*], Ki Duk Park[1,3,*]

[1]Center for Brain Disorders, Brain Science Institute, Korea Institute of Science & Technology (KIST), Seoul 02792, Republic of Korea

[2]Department of Biotechnology, College of Life Science and Biotechnology, Yonsei University, Seoul 03722, Republic of Korea

[3]Division of Bio-Medical Science & Technology, KIST School, University of Science and Technology, Seoul 02792, Republic of Korea

[4]Doping Control Center, KIST, Seoul 02792, Republic of Korea

Corresponding Author Information

*E-mail: jhyunprk@kist.re.kr Phone: +82-2-958-6979. Fax: +82-2-958-5189.

*E-mail: jiwon0602@kist.re.kr Phone: +82-2-958-5132. Fax: +82-2-958-5189.

*E-mail: leehicam@yonsei.ac.kr Phone: +82-2-2123-2866. Fax: +82-2-362-7265.

*E-mail: kdpark@kist.re.kr Phone: +82-2-958-5132. Fax: +82-2-958-5189.

Table of Contents

1. Supplemental Results-- S4
1.1 Evaluation of reported vinyl sulfones--- S4

2. Supplemental Experimental Section--- S5
2.1 Synthesis of **1a**–**1e**-- S5
2.2 Synthesis of **2a**–**2e**-- S6
2.3 Synthesis of **3a**–**3k**-- S8
2.4 Synthesis of **4a**–**4aa**-- S12
2.5 Synthesis of **6a**–**6u**-- S20

3. ^{1}H and ^{13}C-NMR Spectra for the Final Compounds-- S27

4. HPLC Trace for the Final Compounds-- S75

5. HRMS Analysis for the Final Compounds-- S81

1. Supplemental Results

1.1 Evaluation of reported vinyl sulfones

Table S1. Reported vinyl sulfone derivatives and their effects on Nrf2 activation

	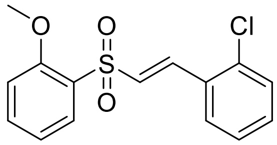 *a*	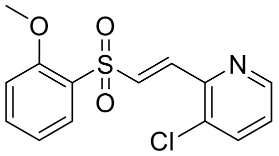 *b*
Nrf2-activating potency (EC_{50}, μM)	0.530 ± 0.025	0.142 ± 0.008

[a]Lead compound in S. Woo et al., *Discovery of Vinyl Sulfones as a Novel Class of Neuroprotective Agents toward Parkinson's Disease Therapy*, *J. Med. Chem.*, 2014, 57, 4, 1473–1487. [b]A vinyl sulfone derivative in J. Choi et al., *Optimization of Vinyl Sulfone Derivatives as Potent Nuclear Factor Erythroid 2-Related Factor 2 (Nrf2) Activators for Parkinson's Disease Therapy*, *J. Med. Chem.*, 2019, 62, 2, 811–830.

2. Supplemental Experimental Section

2.1 Synthesis of 1a–1e

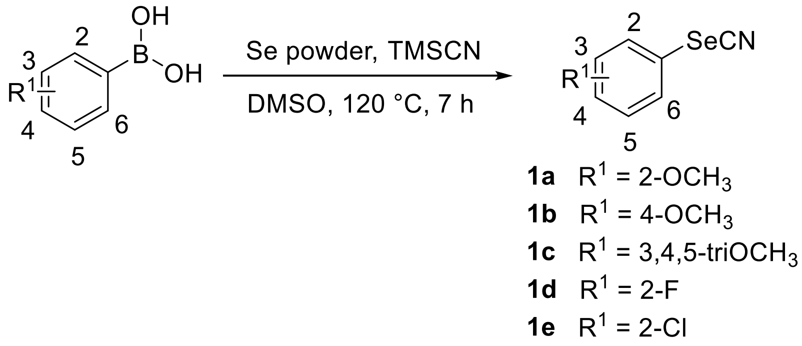

Preparation of 1-methoxy-2-selenocyanatobenzene (1a)

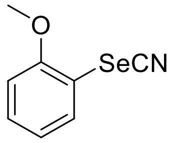

Using Method A, commercially available 2-methoxyphenylboronic acid (2.00 g, 13.16 mmol), selenium powder (3.12 g, 39.48 mmol), and TMSCN (3.29 mL, 26.32 mmol) gave 1.79 g (64%) of **1a** as a yellow oil; R_f = 0.54 (*n*-hexane:EtOAc = 3:1); ^{1}H NMR (400 MHz, $CDCl_3$) δ 7.64 (dd, *J* = 7.8, 1.3 Hz, 1H), 7.37–7.32 (m, 1H), 7.03 (t, *J* = 7.6 Hz, 1H), 6.90 (d, *J* = 8.2 Hz, 1H), 3.90 (s, 3H).

Preparation of 1-methoxy-4-selenocyanatobenzene (1b)

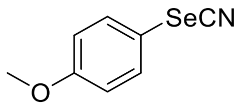

Using Method A, commercially available 4-methoxyphenylboronic acid (2.00 g, 13.16 mmol), selenium powder (3.12 g, 39.48 mmol), and TMSCN (3.29 mL, 26.32 mmol) gave 2.08 g (75%) of **1b** as a yellow oil; R_f = 0.49 (*n*-hexane:EtOAc = 3:1); ^{1}H NMR (400 MHz, $CDCl_3$) δ 7.60 (d, *J* = 8.8 Hz, 2H), 6.92 (d, *J* = 8.8 Hz, 2H), 3.83 (s, 3H).

Preparation of 1,2,3-trimethoxy-5-selenocyanatobenzene (1c)

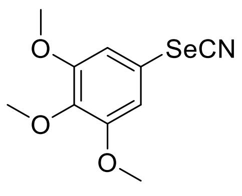

Using Method A, commercially available 3,4,5-trimethoxyphenylboronic acid (0.26 g, 0.93 mmol), selenium powder (0.22 g, 2.79 mmol), and TMSCN (0.23 mL, 1.86 mmol) gave 0.12 g (49%) of **1c** as a yellow oil; R_f = 0.74 (*n*-hexane:EtOAc = 1:1); ^{1}H NMR (400 MHz, $CDCl_3$) δ 6.84 (s, 2H), 3.87 (s, 6H), 3.84 (s, 3H).

Preparation of 1-fluoro-2-selenocyanatobenzene (1d)

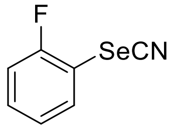

Using Method A, commercially available 2-fluorophenylboronic acid (3.00 g, 21.44 mmol), selenium powder (5.08 g, 64.32 mmol), and TMSCN (5.36 mL, 42.88 mmol) gave 3.78 g (88%) of **1d** as a clear oil; R_f = 0.55 (*n*-hexane:EtOAc = 3:1); ^{1}H NMR (400 MHz, $CDCl_3$) δ 7.71 (t, J = 6.8 Hz, 1H), 7.46–7.41 (m, 1H), 7.25–7.17 (m, 2H).

Preparation of 1-chloro-2-selenocyanatobenzene (1e)

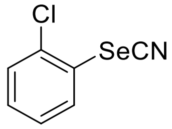

Using Method A, commercially available 2-chlorophenylboronic acid (3.00 g, 19.19 mmol), selenium powder (4.55 g, 57.56 mmol), and TMSCN (4.80 mL, 38.37 mmol) gave 3.97 g (95%) of **1e** as a yellow oil; R_f = 0.65 (*n*-hexane:EtOAc = 3:1); ^{1}H NMR (400 MHz, $CDCl_3$) δ 7.79–7.76 (m, 1H), 7.43–7.31 (m, 3H).

2.2 Synthesis of 2a–2e

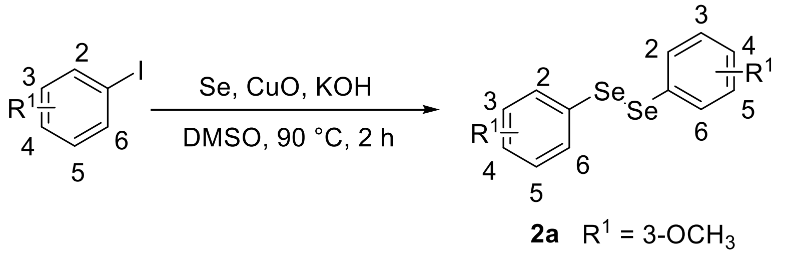

2a R^1 = 3-OCH_3
2b R^1 = 3-F
2c R^1 = 4-F
2d R^1 = 3-Cl
2e R^1 = 4-Cl

Preparation of 1,2-bis(3-methoxyphenyl)diselane (2a)

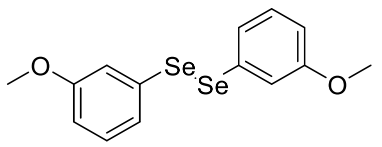

Using Method B, commercially available 1-iodo-3-methoxybenzene (2.00 g, 8.55 mmol), selenium powder (1.35 g, 17.09 mmol), CuO (0.07 g, 0.85 mmol), and 85% KOH (1.13 g, 17.09 mmol) gave 1.37 g (86%) of **2a** as an orange oil; $R_f = 0.39$ (*n*-hexane:EtOAc = 19:1); ^{1}H NMR (400 MHz, $CDCl_3$) δ 7.21–7.15 (m, 6H), 6.78 (d, $J = 7.2$, 2H), 3.77 (s, 6H).

Preparation of 1,2-bis(3-fluorophenyl)diselane (2b)

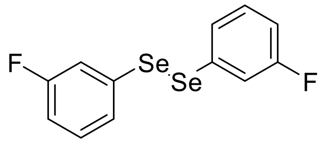

Using Method B, commercially available 1-fluoro-3-iodobenzene (2.00 g, 9.01 mmol), selenium powder (1.42 g, 18.02 mmol), CuO (0.07 g, 0.90 mmol), and 85% KOH (1.19 g, 18.02 mmol) gave 0.94 g (60%) of **2b** as a yellow oil; $R_f = 0.64$ (*n*-hexane:EtOAc = 19:1); ^{1}H NMR (400 MHz, $CDCl_3$) δ 7.39–7.21 (m, 6H), 6.94 (t, $J = 8.3$, 2H).

Preparation of 1,2-bis(4-fluorophenyl)diselane (2c)

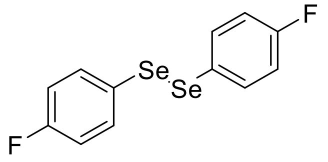

Using Method B, commercially available 1-fluoro-4-iodobenzene (2.00 g, 9.01 mmol), selenium powder (1.42 g, 18.02 mmol), CuO (0.07 g, 0.90 mmol), and 85% KOH (1.19 g, 18.02 mmol) gave 0.90 g (57%) of **2c** as a yellow oil; $R_f = 0.68$ (*n*-hexane:EtOAc = 19:1); ^{1}H NMR (400 MHz, $CDCl_3$) δ 7.55 (t, $J = 6.6$, 4H), 6.97 (t, $J = 8.5$, 4H).

Preparation of 1,2-bis(3-chlorophenyl)diselane (2d)

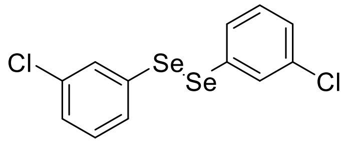

Using Method B, commercially available 1-chloro-3-iodobenzene (0.52 mL, 4.19 mmol), selenium powder (0.66 g, 8.39 mmol), CuO (0.033 g, 0.42 mmol), and 85% KOH (0.55 g, 8.39 mmol) gave 0.65 g (41%) of **2d** as a orange oil; $R_f = 0.65$ (*n*-hexane:EtOAc = 10:1); ^{1}H NMR

(400 MHz, DMSO-d_6) δ 7.67 (s, 2H), 7.60–7.58 (m, 2H), 7.38–7.35 (m, 4H).

Preparation of 1,2-bis(4-chlorophenyl)diselane (2e)

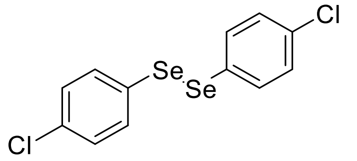

Using Method B, commercially available 1-chloro-4-iodobenzene (1.00 g, 4.19 mmol), selenium powder (0.66 g, 8.39 mmol), CuO (0.033 g, 0.42 mmol), and 85% KOH (0.55 g, 8.39 mmol) gave 0.49 g (31%) of **2e** as a yellow powder; R_f = 0.69 (n-hexane:EtOAc = 10:1); ^{1}H NMR (400 MHz, $CDCl_3$) δ 7.64–7.62 (m, 4H), 7.42–7.39 (m, 4H).

2.3 Synthesis of 3a–3k

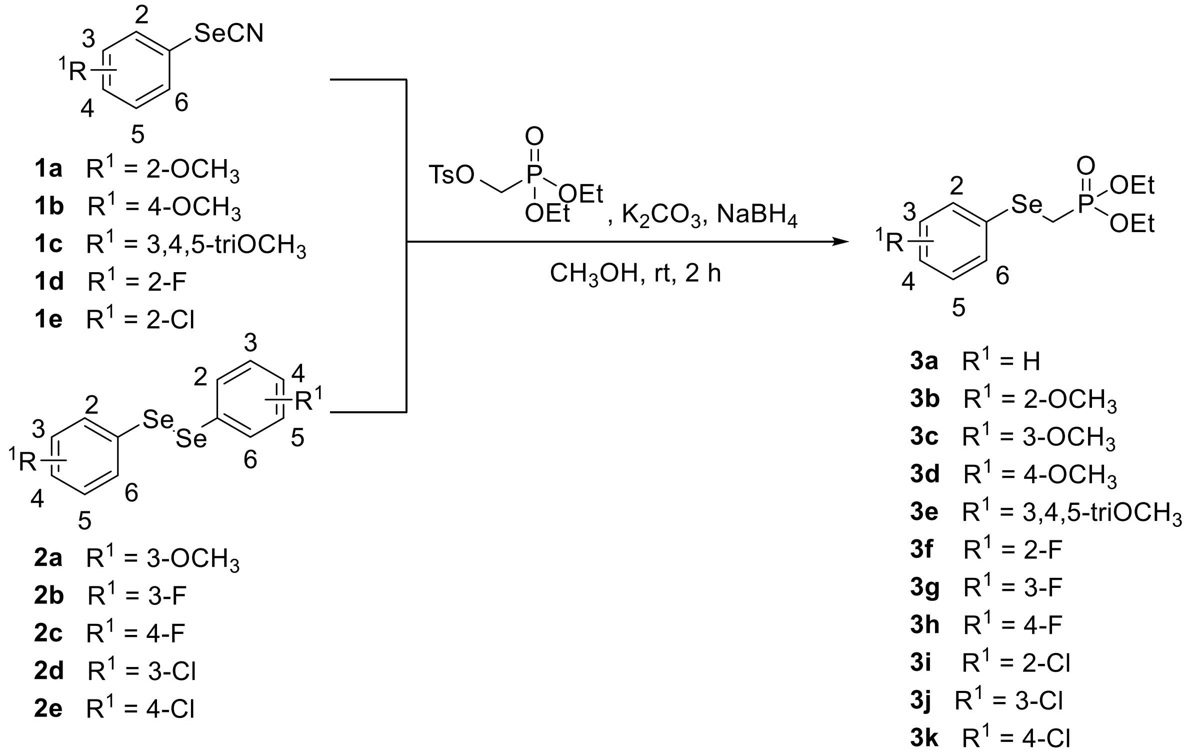

Preparation of diethyl ((phenylselanyl)methyl)phosphonate (3a)

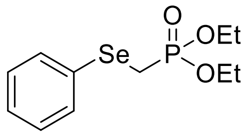

Using Method C, commercially available selenocyanatobenzene (0.20 mL, 1.65 mmol), diethyl

(p-toluenesulfonyloxymethyl)phosphonate (0.51 mL, 1.81 mmol), $NaBH_4$ (0.075 g, 1.98 mmol), and K_2CO_3 (0.45 g, 3.29 mmol) gave 0.31 g (62%) of **3a** as a clear oil; R_f = 0.47 (n-hexane:EtOAc = 1:3); ^{1}H NMR (400MHz, DMSO-d_6) δ 7.57–7.55 (m, 2H), 7.32–7.26 (m, 3H), 4.00 (quint, J = 7.1 Hz, 4H), 3.28 (d, J = 12.5 Hz, 2H), 1.18 (t, J = 7.1 Hz, 6H).

Preparation of diethyl (((2-methoxyphenyl)selanyl)methyl)phosphonate (3b)

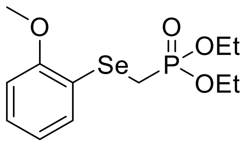

Using Method C, **1a** (1.79 g, 8.44 mmol), diethyl (p-toluenesulfonyloxymethyl)phosphonate (2.17 mL, 8.44 mmol), $NaBH_4$ (0.38 g, 10.13 mmol), and K_2CO_3 (2.33 g, 16.88 mmol) gave 2.35 g (83%) of **3b** as a clear oil; R_f = 0.15 (n-hexane:EtOAc = 1:2); ^{1}H NMR (400MHz, $CDCl_3$) δ 7.46 (dd, J = 7.7, 1.4 Hz, 1H), 7.27–7.23 (m, 1H), 6.92 (t, J = 7.2 Hz, 1H), 6.85 (d, J = 8.1 Hz, 1H), 4.17–4.08 (m, 4H), 3.89 (s, 3H), 3.10–3.06 (m, 2H), 1.30 (t, J = 7.1 Hz, 6H).

Preparation of diethyl (((3-methoxyphenyl)selanyl)methyl)phosphonate (3c)

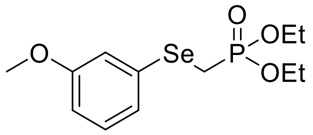

Using Method C, **2a** (1.37 g, 3.68 mmol), diethyl (p-toluenesulfonyloxymethyl)phosphonate (1.90 mL, 7.36 mmol), $NaBH_4$ (0.33 g, 8.83 mmol), and K_2CO_3 (1.20 g, 8.83 mmol) gave 1.07 g (47%) of **3c** as a yellow oil; R_f = 0.25 (n-hexane:EtOAc = 1:2); ^{1}H NMR (400MHz, $CDCl_3$) δ 7.21–7.13 (m, 3H), 6.82–6.79 (m, 1H), 4.18–4.10 (m, 4H), 3.80 (s, 3H), 3.08–3.05 (m, 2H), 1.31 (t, J = 7.1 Hz, 6H).

Preparation of diethyl (((4-methoxyphenyl)selanyl)methyl)phosphonate (3d)

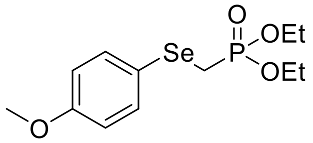

Using Method C, **1b** (2.08 g, 9.81 mmol), diethyl (p-toluenesulfonyloxymethyl)phosphonate (2.53 mL, 9.81 mmol), $NaBH_4$ (0.45 g, 11.77 mmol), and K_2CO_3 (2.71 g, 19.61 mmol) gave 2.24 g (68%) of **3d** as a clear oil; R_f = 0.21 (n-hexane:EtOAc = 1:2); ^{1}H NMR (400MHz, $CDCl_3$) δ 7.59–7.56 (m, 2H), 6.84–6.81 (m, 2H), 4.16–4.08 (m, 4H), 3.80 (s, 3H), 2.98–2.93 (m, 2H), 1.31 (t, J = 7.1 Hz, 6H).

Preparation of diethyl (((3,4,5-trimethoxyphenyl)selanyl)methyl)phosphonate (3e)

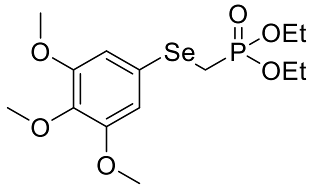

Using Method C, **1c** (0.27 g, 1.00 mmol), diethyl (*p*-toluenesulfonyloxymethyl)phosphonate (0.28 mL, 1.00 mmol), $NaBH_4$ (0.046 g, 1.20 mmol), and K_2CO_3 (0.27 g, 2.01 mmol) gave 0.19 g (48%) of **3e** as a clear oil; R_f= 0.24 (*n*-hexane:EtOAc = 1:1); ^{1}H NMR (400MHz, $CDCl_3$) δ 6.90 (s, 2H), 4.15 (quint, J = 7.2 Hz, 4H), 3.86 (s, 6H), 3.17 (s, 3H), 3.04 (d, J = 12.3 Hz, 2H), 1.32 (t, J = 7.0 Hz, 6H).

Preparation of diethyl (((2-fluorophenyl)selanyl)methyl)phosphonate (3f)

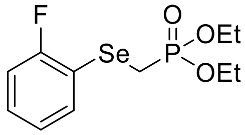

Using Method C, **1d** (3.78 g, 18.89 mmol), diethyl (*p*-toluenesulfonyloxymethyl)phosphonate (4.87 mL, 18.89 mmol), $NaBH_4$ (0.93 g, 24.56 mmol), and K_2CO_3 (5.22 g, 37.78 mmol) gave 5.50 g (90%) of **3f** as a pale yellow oil; R_f= 0.43 (*n*-hexane:EtOAc = 1:3); ^{1}H NMR (400MHz, $CDCl_3$) δ 7.62–7.58 (m, 1H), 7.31–7.26 (m, 1H), 7.10–7.04 (m, 2H), 4.16–4.08 (m, 4H), 3.10–3.04 (m, 2H), 1.29 (t, J = 7.0 Hz, 6H).

Preparation of diethyl (((3-fluorophenyl)selanyl)methyl)phosphonate (3g)

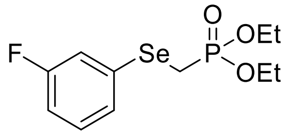

Using Method C, **2b** (0.94 g, 2.71 mmol), diethyl (*p*-toluenesulfonyloxymethyl)phosphonate (1.40 mL, 5.43 mmol), $NaBH_4$ (0.25 g, 6.51 mmol), and K_2CO_3 (0.90 g, 6.51 mmol) gave 1.39 g (79%) of **3g** as a pale yellow oil; R_f= 0.39 (*n*-hexane:EtOAc = 1:3); ^{1}H NMR (400MHz, $CDCl_3$) δ 7.39–7.21 (m, 3H), 6.97 (td, J = 8.5, 2.4 Hz, 1H), 4.18–4.10 (m, 4H), 3.10–3.02 (m, 2H, 1.31 (t, J = 7.1 Hz, 6H).

Preparation of diethyl (((4-fluorophenyl)selanyl)methyl)phosphonate (3h)

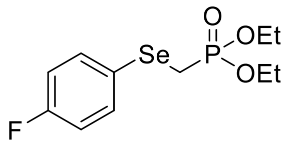

Using Method C, **2c** (0.90 g, 2.58 mmol), diethyl (*p*-toluenesulfonyloxymethyl)phosphonate (1.33 mL, 5.17 mmol), $NaBH_4$ (0.23 g, 6.20 mmol), and K_2CO_3 (0.86 g, 6.20 mmol) gave 1.15 g (69%) of **3h** as a clear oil; R_f= 0.42 (*n*-hexane:EtOAc = 1:2); ^{1}H NMR (400MHz, $CDCl_3$) δ 7.62 (t, J = 6.5 Hz, 2H), 6.99 (t, J = 8.5 Hz, 2H), 4.13 (p, J = 7.4 Hz, 4H), 3.03–2.95 (m, 2H), 1.31 (t, J = 7.1 Hz, 6H).

Preparation of diethyl (((2-chlorophenyl)selanyl)methyl)phosphonate (3i)

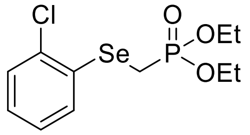

Using Method C, **1e** (3.94 g, 18.20 mmol), diethyl (*p*-toluenesulfonyloxymethyl)phosphonate (4.69 mL, 18.20 mmol), $NaBH_4$ (0.89 g, 23.65 mmol), and K_2CO_3 (5.03 g, 36.39 mmol) gave 5.00 g (80%) of **3i** as a pale yellow oil; R_f= 0.55 (*n*-hexane:EtOAc = 1:2); ^{1}H NMR (400MHz, $CDCl_3$) δ 7.53–7.51 (m, 1H), 7.38–7.35 (m, 1H), 7.23–7.17 (m, 2H), 4.19–4.12 (m, 4H), 3.13–3.07 (m, 2H), 1.32 (t, J = 7.1 Hz, 6H).

Preparation of diethyl (((3-chlorophenyl)selanyl)methyl)phosphonate (3j)

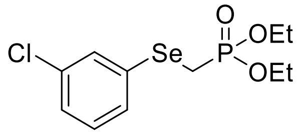

Using Method C, **2d** (0.65 g, 1.73 mmol), diethyl (*p*-toluenesulfonyloxymethyl)phosphonate (1.11 g, 3.45 mmol), $NaBH_4$ (0.15 g, 4.14 mmol), and K_2CO_3 (0.95 g, 6.91 mmol) gave 0.62 g (53%) of **3j** as a clear oil; R_f= 0.38 (*n*-hexane:EtOAc = 1:1); ^{1}H NMR (400MHz, $CDCl_3$) δ 7.65 (s, 1H), 7.52–7.49 (m, 1H), 7.33–7.30 (m, 2H), 4.00 (quint, J = 7.3 Hz, 4H), 3.37 (d, J = 12.3 Hz, 2H), 1.19 (t, J = 7.0 Hz, 6H).

Preparation of diethyl (((4-chlorophenyl)selanyl)methyl)phosphonate (3k)

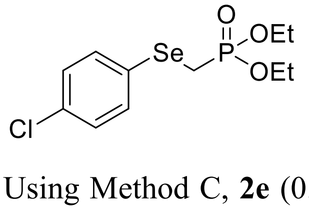

Using Method C, **2e** (0.49 g, 1.29 mmol), diethyl (*p*-toluenesulfonyloxymethyl)phosphonate

(0.83 mg, 2.57 mmol), $NaBH_4$ (0.11 g, 3.09 mmol), and K_2CO_3 (0.71 g, 5.14 mmol) gave 0.44 g (50%) of **3k** as a clear oil; R_f = 0.38 (*n*-hexane:EtOAc = 1:1); ^{1}H NMR (400MHz, DMSO-d_6) δ 7.58 (d, J = 8.6 Hz, 2H), 7.36 (d, J = 8.5 Hz, 2H), 4.00 (quint, J = 7.2 Hz, 4H), 3.31 (d, J = 12.2 Hz, 2H), 1.18 (t, J = 7.04 Hz, 6H).

2.4 Synthesis of 4a–4aa

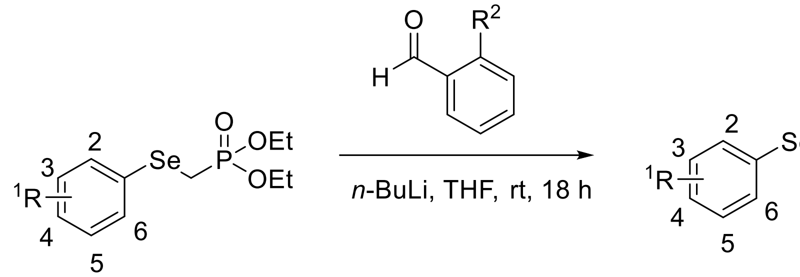

**3a** R^1 = H	**4a** R^1 = H, R^2 = H	**4o** R^1 = 2-F, R^2 = OCH_3
**3b** R^1 = 2-OCH_3	**4b** R^1 = 2-OCH_3, R^2 = H	**4p** R^1 = 2-F, R^2 = F
**3c** R^1 = 3-OCH_3	**4c** R^1 = 2-OCH_3, R^2 = OCH_3	**4q** R^1 = 2-F, R^2 = Cl
**3d** R^1 = 4-OCH_3	**4d** R^1 = 2-OCH_3, R^2 = F	**4r** R^1 = 2-F, R^2 = CF_3
**3e** R^1 = 3,4,5-triOCH_3	**4e** R^1 = 2-OCH_3, R^2 = Cl	**4s** R^1 = 2-F, R^2 = OCF_3
**3f** R^1 = 2-F	**4f** R^1 = 2-OCH_3, R^2 = CF_3	**4t** R^1 = 2-Cl, R^2 = H
**3g** R^1 = 3-F	**4g** R^1 = 2-OCH_3, R^2 = OCF_3	**4u** R^1 = 2-Cl, R^2 = OCH_3
**3h** R^1 = 4-F	**4h** R^1 = 3-OCH_3, R^2 = Cl	**4v** R^1 = 2-Cl, R^2 = F
**3i** R^1 = 2-Cl	**4i** R^1 = 4-OCH_3, R^2 = OCH_3	**4w** R^1 = 2-Cl, R^2 = Cl
**3j** R^1 = 3-Cl	**4j** R^1 = 4-OCH_3, R^2 = F	**4x** R^1 = 2-Cl, R^2 = CF_3
**3k** R^1 = 4-Cl	**4k** R^1 = 4-OCH_3, R^2 = Cl	**4y** R^1 = 2-Cl, R^2 = OCF_3
	**4l** R^1 = 4-OCH_3, R^2 = CF_3	**4z** R^1 = 3-Cl, R^2 = Cl
	**4m** R^1 = 3,4,5-triOCH_3, R^2 = Cl	**4aa** R^1 = 4-Cl, R^2 = Cl
	**4n** R^1 = 2-F, R^2 = H	

Preparation of (*E*)-phenyl(styryl)selane (4a)

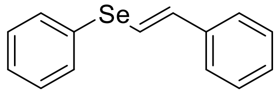

Using Method D, **3a** (1.32 g, 4.30 mmol), 2.0 M *n*-BuLi (3.23 mL, 6.46 mmol) and benzaldehyde (0.65 mL, 6.46 mmol) gave 0.58 g (53%) of **4a** as a pale yellow oil; R_f = 0.36 (*n*-hexane:EtOAc = 1:1); ^{1}H NMR (400MHz, DMSO-d_6) δ 7.55 (d, J = 6.8 Hz, 2H), 7.50–7.46 (m, 3H), 7.40–7.32 (m, 5H), 7.28–7.25 (m, 1H), 6.93 (d, J = 15.8 Hz, 1H).

Preparation of (*E*)-(2-methoxyphenyl)(styryl)selane (4b)

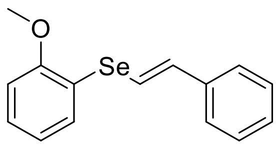

Using Method D, **3b** (0.20 g, 0.59 mmol), 2.0 M *n*-BuLi (0.45 mL, 0.89 mmol) and benzaldehyde (0.07 mL, 0.65 mmol) gave 0.12 g (72%) of **4b** as a yellow oil; R_f= 0.48 (*n*-hexane:EtOAc = 19:1); ^{1}H NMR (400MHz, $CDCl_3$) δ 7.41–7.30 (m, 5H), 7.28–7.22 (m, 2H), 7.18 (d, J= 15.7 Hz, 1H), 7.01 (d, J= 15.8 Hz, 1H), 6.96–6.85 (m, 2H), 3.90 (s, 3H).

Preparation of (*E*)-(2-methoxyphenyl)(2-methoxystyryl)selane (4c)

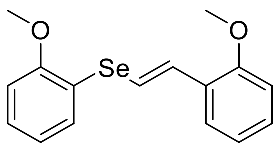

Using Method D, **3b** (0.20 g, 0.60 mmol), 2.0 M *n*-BuLi (0.45 mL, 0.90 mmol) and 2-methoxybenzaldehyde (0.12 mL, 0.90 mmol) gave 0.089 g (46%) of **4c** as a yellow oil; R_f= 0.71 (*n*-hexane:EtOAc = 3:1); ^{1}H NMR (400MHz, DMSO-d_6) δ 7.59 (dd, J= 7.6, 1.6 Hz, 1H), 7.37 (d, J= 15.9 Hz, 1H), 7.33 (dd, J= 7.6, 1.5 Hz, 1H), 7.30–7.25 (m, 2H), 7.22 (d, J= 15.9 Hz, 1H), 3.83 (s, 3H), 3.81 (s, 3H).

Preparation of (*E*)-phenyl(styryl)selane (4d)

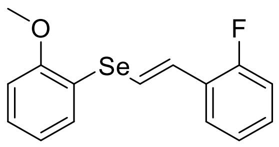

Using Method D, **3b** (0.12 g, 0.38 mmol), 2.0 M *n*-BuLi (0.28 mL, 0.57 mmol) and 2-fluorobenzaldehyde (0.060 mL, 0.57 mmol) gave 0.045 g (39%) of **4d** as a clear oil; R_f= 0.70 (*n*-hexane:EtOAc = 3:1); ^{1}H NMR (400MHz, DMSO-d_6) δ 7.73 (td, J= 7.7, 1.6 Hz, 1H), 7.55 (d, J= 15.9 Hz, 1H), 7.40 (dd, J= 7.6, 1.5 Hz, 1H), 7.33–7.28 (m, 2H), 7.22–7.18 (m, 2H), 7.06–7.01 (m, 2H), 6.97 (td, J= 7.5, 1.1 Hz, 1H), 3.84 (s, 3H).

Preparation of (*E*)-(2-chlorostyryl)(2-methoxyphenyl)selane (4e)

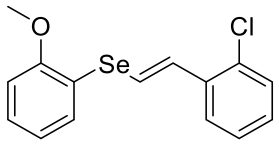

Using Method D, **3b** (2.02 g, 5.99 mmol), 2.0 M *n*-BuLi (4.49 mL, 8.99 mmol) and 2-chlorobenzaldehyde (1.01 mL, 8.99 mmol) gave 0.87 g (45%) of **4e** as a yellow oil; R_f= 0.49

(n-hexane:EtOAc = 9:1); ^{1}H NMR (400MHz, DMSO-d_6) δ 7.83 (dd, J = 7.5, 1.8 Hz, 1H), 7.59 (d, J = 15.7 Hz, 1H), 7.45–7.43 (m, 2H), 7.35–7.27 (m, 3H), 7.15 (d, J = 15.7 Hz, 1H), 7.06 (d, J = 8.2 Hz, 1H), 6.98 (td, J = 7.5, 1.0 Hz, 1H), 3.84 (s, 3H).

Preparation of (*E*)-(2-methoxyphenyl)(2-(trifluoromethyl)styryl)selane (4f)

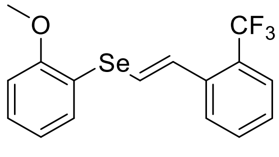

Using Method D, **3b** (0.20 g, 0.60 mmol), 2.0 M n-BuLi (0.45 mL, 0.90 mmol) and 2-trifluomethylbenzaldehyde (0.12 mL, 0.90 mmol) gave 0.10 g (48%) of **4f** as a yellow oil; R_f= 0.69 (n-hexane:EtOAc = 3:1); ^{1}H NMR (400MHz, DMSO-d_6) δ 7.89–7.86 (m, 1H), 7.65 (d, J = 15.8 Hz, 1H), 7.43 (dd, J = 7.6, 1.5 Hz, 1H), 7.40–7.38 (m, 2H), 7.35–7.31 (m, 2H), 7.08 (dd, J = 8.2, 1.0 Hz, 1H), 6.98 (td, J = 7.5, 1.1 Hz, 1H), 6.92 (d, J = 15.8 Hz, 1H), 3.82 (s, 3H).

Preparation of (*E*)-(2-methoxyphenyl)(2-(trifluoromethoxy)styryl)selane (4g)

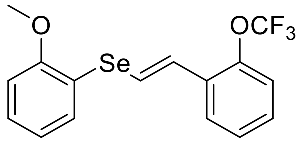

Using Method D, **3b** (0.31 g, 0.93 mmol), 2.0 M n-BuLi (1.0 mL, 2.09 mmol) and 2-trifluoromethoxybenzaldehyde (0.20 mL, 1.39 mmol) gave 0.18 g (54%) of **4g** as a yellow oil; R_f= 0.77 (n-hexane:EtOAc = 3:1); ^{1}H NMR (400MHz, DMSO-d_6) δ 7.89–7.86 (m, 1H), 7.65 (d, J = 15.8 Hz, 1H), 7.44 (dd, J = 7.5, 1.3 Hz, 1H), 7.40–7.37 (m, 2H), 7.35–7.31 (m, 2H), 7.07 (d, J = 8.2 Hz, 1H), 6.98 (t, J = 7.6 Hz, 1H), 6.92 (d, J = 15.8 Hz, 1H), 3.83 (s, 3H).

Preparation of (*E*)-(2-chlorostyryl)(3-methoxyphenyl)selane (4h)

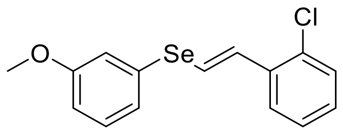

Using Method D, **3c** (0.30 g, 0.89 mmol), 2.0 M n-BuLi (0.66 mL, 1.33 mmol) and 2-chlorobenzaldehyde (0.12 mL, 1.07 mmol) gave 0.26 g (91%) of **4h** as a yellow oil; R_f= 0.34 (100% n-hexane); ^{1}H NMR (400MHz, DMSO-d_6) δ 7.48 (dd, J = 7.8, 1.8 Hz, 1H), 7.34 (dd, J = 7.6, 2.0 Hz, 1H), 7.27–7.10 (m, 7H), 6.85 (dd, J = 8.2, 1.5 Hz, 1H), 3.82 (s, 3H).

Preparation of (*E*)-(4-methoxyphenyl)(2-methoxystyryl)selane (4i)

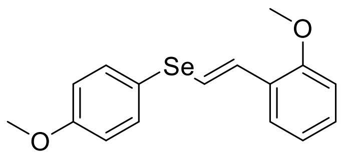

Using Method D, **3d** (0.20 g, 0.60 mmol), 2.0 M *n*-BuLi (0.45 mL, 0.90 mmol) and 2-methoxybenzaldehyde (0.12 mg, 0.90 mmol) gave 0.043 g (22%) of **4i** as a white oil; R_f= 0.35 (*n*-hexane:EtOAc = 9:1); ^{1}H NMR (400MHz, DMSO-d_6) δ 7.46 (t, J = 8.7 Hz, 3H), 7.33 (d, J = 15.8 Hz, 1H), 7.23 (t, J = 7.5 Hz, 1H), 6.99–6.95 (m, 3H), 6.92–6.87 (m, 1H), 3.76 (s, 6H).

Preparation of (*E*)-(2-fluorostyryl)(4-methoxyphenyl)selane (4j)

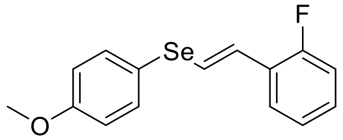

Using Method D, **3d** (0.20 g, 0.60 mmol), 2.0 M *n*-BuLi (0.45 mL, 0.90 mmol) and 2-fluorobenzaldehyde (0.090 mL, 0.90 mmol) gave 0.051 g (28%) of **4j** as a yellow oil; R_f= 0.81 (*n*-hexane:EtOAc = 9:1); ^{1}H NMR (400MHz, DMSO-d_6) δ 7.59 (t, J = 7.8 Hz, 1H), 7.53–7.50 (m, 3H), 7.29–7.24 (m, 1H), 7.14 (t, J = 8.0 Hz, 2H), 6.88 (d, J = 8.2 Hz, 2H), 6.67 (d, J = 15.8 Hz, 1H), 3.78 (s, 3H).

Preparation of (*E*)-(2-chlorostyryl)(4-methoxyphenyl)selane (4k)

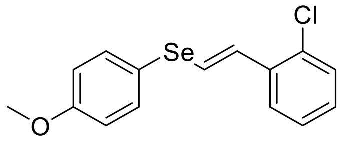

Using Method D, **3d** (0.20 g, 0.61 mmol), 2.0 M *n*-BuLi (0.46 mL, 0.91 mmol) and 2-chlorobenzaldehyde (0.10 mL, 0.91 mmol) gave 0.041 g (21%) of **4k** as a yellow oil; R_f= 0.53 (*n*-hexane:EtOAc = 9:1); ^{1}H NMR (400MHz, DMSO-d_6) δ 7.69 (d, J = 7.6 Hz, 1H), 7.40 (t, J = 8.6 Hz, 3H), 7.29 (t, J = 7.4 Hz, 1H), 7.00 (d, J = 8.5 Hz, 2H), 6.88 (d, J = 8.5 Hz, 2H), 6.83 (d, J = 15.8 Hz, 1H), 3.78 (s, 3H).

Preparation of (*E*)-(4-methoxyphenyl)(2-(trifluoromethyl)styryl)selane (4l)

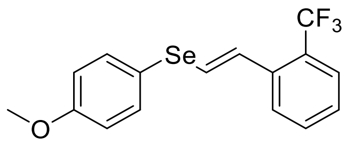

Using Method D, **3d** (0.10 g, 0.30 mmol), 2.0 M *n*-BuLi (0.30 mL, 0.59 mmol) and 2-

trifluoromethylbenzaldehyde (0.05 mL, 0.36 mmol) gave 0.07 g (70%) of **4l** as a yellow oil; R_f = 0.37 (*n*-hexane:EtOAc = 14:1); ^{1}H NMR (400MHz, $CDCl_3$) δ 7.60–7.51 (m, 4H), 7.45 (t, J = 7.6 Hz, 1H), 7.29 (t, J = 7.7 Hz, 1H), 7.18 (d, J = 15.4 Hz, 1H), 6.91–6.85 (m, 3H), 3.83 (s, 3H).

Preparation of (*E*)-(2-chlorostyryl)(3,4,5-trimethoxyphenyl)selane (4m)

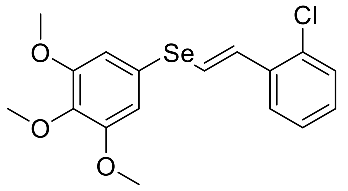

Using Method D, **3e** (0.19 g, 0.48 mmol), 2.0 M *n*-BuLi (0.36 mL, 0.72 mmol) and 2-chlorobenzaldehyde (0.080 mL, 0.72 mmol) gave 0.080 g (43%) of **4m** as a clear oil; R_f= 0.54 (*n*-hexane:EtOAc = 3:1); ^{1}H NMR (400MHz, DMSO-d_6) δ 7.76 (dd, J = 7.6 Hz, 1H), 7.64 (d, J = 15.7 Hz, 1H), 7.42 (dd, J = 7.7, 1.2 Hz, 1H), 7.33–7.26 (m, 2H), 7.02 (d, J = 15.8 Hz, 1H), 6.88 (s, 2H), 3.79 (s, 6H), 3.66 (s, 3H).

Preparation of (*E*)-(2-fluorophenyl)(styryl)selane (4n)

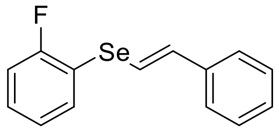

Using Method D, **3f** (0.64 g, 1.97 mmol), 2.0 M *n*-BuLi (1.48 mL, 2.95 mmol) and benzaldehyde (0.29 mL, 2.95 mmol) gave 0.17 g (32%) of **4n** as a clear oil; R_f= 0.39 (*n*-hexane 100%); ^{1}H NMR (400MHz, DMSO-d_6) δ 7.61–7.57 (m, 1H), 7.52–7.22 (m, 8H), 6.99 (d, J = 15.7 Hz, 1H).

Preparation of (*E*)-(2-fluorophenyl)(2-methoxystyryl)selane (4o)

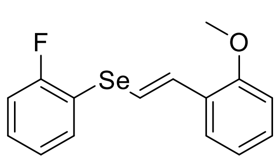

Using Method D, **3f** (0.20 g, 0.62 mmol), 2.0 M *n*-BuLi (0.46 mL, 0.92 mmol) and 2-methoxybenzaldehyde (0.09 g, 0.68 mmol) gave 0.12 g (64%) of **4o** as a clear oil; R_f= 0.60 (*n*-hexane:EtOAc = 19:1); ^{1}H NMR (400MHz, $CDCl_3$) δ 7.49 (t, J = 7.2 Hz, 1H), 7.38 (d, J = 7.6 Hz, 1H), 7.33–7.22 (m, 3H), 7.17 (d, J = 15.8 Hz, 1H), 7.11–7.04 (m, 2H), 6.93 (d, J = 7.5 Hz, 1H), 6.88 (d, J = 8.5 Hz, 1H), 3.85 (s, 3H).

Preparation of (*E*)-(2-fluorophenyl)(2-fluorostyryl)selane (4p)

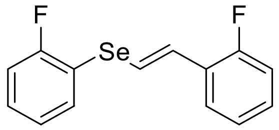

Using Method D, **3f** (0.20 g, 0.62 mmol), 2.0 M *n*-BuLi (0.46 mL, 0.92 mmol) and 2-fluorobenzaldehyde (0.07 mL, 0.68 mmol) gave 0.12 g (68%) of **4p** as a clear oil; R_f= 0.61 (*n*-hexane:EtOAc = 19:1); ^{1}H NMR (400MHz, $CDCl_3$) δ 7.58–7.49 (m, 2H), 7.38 (t, J = 7.8 Hz, 1H), 7.33–7.28 (m, 1H), 7.25 (d, J = 15.8 Hz, 1H), 7.20 (d, J = 7.3 Hz, 1H), 7.14–7.07 (m, 3H), 7.03 (d, J = 15.8 Hz, 1H).

Preparation of (*E*)-(2-chlorostyryl)(2-fluorophenyl)selane (4q)

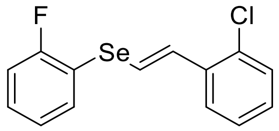

Using Method D, **3f** (1.00 g, 3.08 mmol), 2.0 M *n*-BuLi (2.31 mL, 4.62 mmol) and 2-chlorobenzaldehyde (0.51 mL, 4.61 mmol) gave 0.50 g (53%) of **4q** as a pale yellow oil; R_f= 0.67 (*n*-hexane:EtOAc = 9:1); ^{1}H NMR (400MHz, DMSO-d_6) δ 7.77 (dd, J = 7.5, 1.6 Hz, 1H), 7.67–7.62 (m, 1H), 7.57 (d, J = 15.7 Hz, 1H), 7.50–7.40 (m, 3H), 7.35–7.29 (m, 3H), 7.04 (d, J = 15.7 Hz, 1H).

Preparation of (*E*)-(2-fluorophenyl)(2-(trifluoromethyl)styryl)selane (4r)

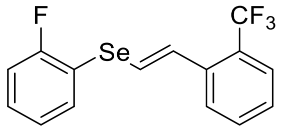

Using Method D, **3f** (0.20 g, 0.62 mmol), 2.0 M *n*-BuLi (0.46 mL, 0.92 mmol) and 2-trifluoromethylbenzaldehyde (0.09 mL, 0.68 mmol) gave 0.15 g (69%) of **4r** as a clear oil; R_f = 0.58 (*n*-hexane:EtOAc = 19:1); ^{1}H NMR (400MHz, $CDCl_3$) δ 7.71 (t, J = 7.9 Hz, 1H), 7.63–7.46 (m, 4H), 7.37–7.27 (m, 2H), 7.17–7.07 (m, 3H).

Preparation of (*E*)-(2-fluorophenyl)(2-(trifluoromethoxy)styryl)selane (4s)

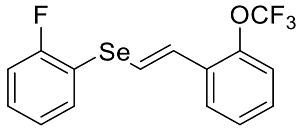

Using Method D, **3f** (0.20 g, 0.62 mmol), 2.0 M *n*-BuLi (0.46 mL, 0.92 mmol) and 2-trifluoromethoxybenzaldehyde (0.10 mL, 0.68 mmol) gave 0.16 g (71%) of **4s** as a clear oil; R_f = 0.63 (*n*-hexane:EtOAc = 19:1); ^{1}H NMR (400MHz, $CDCl_3$) δ 7.62–7.49 (m, 3H), 7.38–7.27 (m, 3H), 7.23–7.10 (m, 3H), 7.04 (d, J = 15.9 Hz, 1H).

Preparation of (*E*)-(2-chlorophenyl)(styryl)selane (4t)

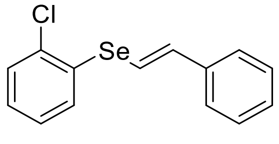

Using Method D, **3i** (0.20 g, 0.59 mmol), 2.0 M *n*-BuLi (0.44 mL, 0.88 mmol) and benzaldehyde (0.09 mL, 0.88 mmol) gave 0.10 g (60%) of **4t** as a light yellow oil; R_f= 0.70 (*n*-hexane:EtOAc = 9:1); ^{1}H NMR (400MHz, DMSO-d_6) δ 7.59–7.57 (m, 2H), 7.52–7.48 (m, 3H), 7.39–7.35 (m, 2H), 7.34–7.27 (m, 3H), 7.15 (d, J = 15.7 Hz, 1H).

Preparation of (*E*)-(2-chlorophenyl)(2-methoxystyryl)selane (4u)

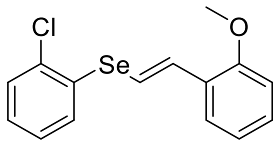

Using Method D, **3i** (0.23 g, 0.68 mmol), 2.0 M *n*-BuLi (0.51 mL, 1.02 mmol) and 2-methoxybenzaldehyde (0.14 mL, 1.02 mmol) gave 0.15 g (68%) of **4u** as a light yellow oil; R_f = 0.80 (*n*-hexane:EtOAc = 3:1); ^{1}H NMR (400MHz, DMSO-d_6) δ 7.63 (dd, J = 7.6, 1.6 Hz, 1H), 7.50–7.45 (m, 2H), 7.43 (d, J = 15.8 Hz, 1H), 7.35–7.27 (m, 4H), 7.04 (d, J = 7.8 Hz, 1H), 6.96 (t, J = 7.4 Hz, 1H), 3.82 (s, 3H).

Preparation of (*E*)-(2-chlorophenyl)(2-fluorostyryl)selane (4v)

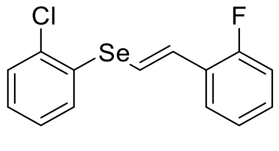

Using Method D, **3i** (0.20 g, 0.60 mmol), 2.0 M *n*-BuLi (0.45 mL, 0.90 mmol) and 2-fluorobenzaldehyde (0.090 mL, 0.90 mmol) gave 0.12 g (67%) of **4v** as a clear oil; R_f= 0.72 (*n*-hexane:EtOAc = 3:1); ^{1}H NMR (400MHz, DMSO-d_6) δ 7.78 (td, J = 7.7, 1.4 Hz, 1H), 7.60 (d, J = 15.9 Hz, 1H), 7.54 (td, J = 7.6, 2.4 Hz, 2H), 7.39–7.31 (m, 3H), 7.25 (m, 2H), 7.14 (d, J = 15.8 Hz, 1H).

Preparation of (*E*)-(2-chlorophenyl)(2-chlorostyryl)selane (4w)

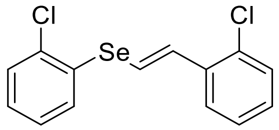

Using Method D, **3i** (1.35 g, 3.95 mmol), 2.0 M *n*-BuLi (2.96 mL, 5.93 mmol) and 2-chlorobenzaldehyde (0.67 mL, 5.93 mmol) gave 0.93 g (72%) of **4w** as a light yellow oil; R_f= 0.51 (*n*-hexane:EtOAc = 9:1); ^{1}H NMR (400MHz, DMSO-d_6) δ 7.87 (dd, *J* = 7.3 Hz, 1H), 7.65 (d, *J* = 15.6 Hz, 1H), 7.60–7.57 (m, 1H), 7.55–7.53 (m, 1H), 7.47 (dd, *J* = 7.4, 1.6 Hz, 1H), 7.38–7.32 (m, 4H), 7.26 (d, *J* = 15.7 Hz, 1H).

Preparation of (*E*)-(2-chlorophenyl)(2-(trifluoromethyl)styryl)selane (4x)

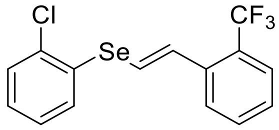

Using Method D, **3i** (0.30 g, 0.88 mmol), 2.0 M *n*-BuLi (0.66 mL, 1.32 mmol) and 2-trifluomethylbenzaldehyde (0.18 mL, 1.32 mmol) gave 0.20 g (64%) of **4x** as a clear oil; R_f= 0.75 (*n*-hexane:EtOAc = 3:1); ^{1}H NMR (400MHz, $CDCl_3$) δ 7.65 (dd, *J* = 7.6, 3.5 Hz, 2H), 7.52 (t, *J* = 7.4 Hz, 1H), 7.47–7.45 (m, 1H), 7.42–7.40 (m, 1H), 7.39–7.37 (m, 1H), 7.36–7.31 (m, 1H), 7.24–7.16 (m, 3H). .

Preparation of (*E*)-(2-chlorophenyl)(2-(trifluoromethoxy)styryl)selane (4y)

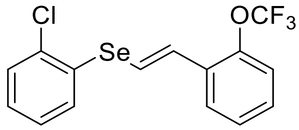

Using Method D, **3i** (0.20 g, 0.59 mmol), 2.0 M *n*-BuLi (0.44 mL, 0.88 mmol) and 2-trifluoromethoxybenzaldehyde (0.09 mL, 0.64 mmol) gave 0.16 g (71%) of **4y** as a clear oil; R_f= 0.35 (*n*-hexane:EtOAc = 19:1); ^{1}H NMR (400MHz, $CDCl_3$) δ 7.57 (dd, *J* = 7.2, 2.0 Hz, 1H), 7.47–7.43 (m, 1H), 7.42–7.38 (m, 1H), 7.32–7.27 (m, 3H), 7.25–7.18 (m, 4H).

Preparation of (*E*)-(3-chlorophenyl)(2-chlorostyryl)selane (4z)

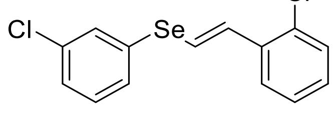

Using Method D, **3j** (0.15 g, 0.47 mmol), 2.0 M *n*-BuLi (0.35 mL, 0.70 mmol) and 2-

chlorobenzaldehyde (0.080 mL, 0.70 mmol) gave 0.056 g (36%) of **4z** as a clear oil; R_f= 0.79 (*n*-hexane:EtOAc = 9:1); ^{1}H NMR (400MHz, DMSO-d_6) δ 7.81 (d, J = 7.4 Hz, 1H), 7.66 (d, J = 15.8 Hz, 1H), 7.54–7.52 (m, 1H), 7.45–7.41 (m, 4H), 7.32 (t, J = 7.4 Hz, 2H), 7.14 (d, J = 15.7 Hz, 1H).

Preparation of (*E*)-(4-chlorophenyl)(2-chlorostyryl)selane (4aa)

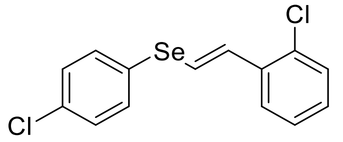

Using Method D, **3k** (0.10 g, 0.31 mmol), 2.0 M *n*-BuLi (0.23 mL, 0.47 mmol) and 2-chlorobenzaldehyde (0.050 mL, 0.47 mmol) gave 0.047 g (46%) of **4aa** as a clear oil; R_f= 0.72 (*n*-hexane:EtOAc = 9:1); ^{1}H NMR (400MHz, $CDCl_3$) δ 7.78 (d, J = 7.4 Hz, 1H), 7.63–7.59 (m, 3H), 7.46–7.42 (m, 3H), 7.30 (quint, J = 8.2 Hz, 2H), 7.08 (d, J = 15.7 Hz, 1H).

2.5 Synthesis of 6a–6u

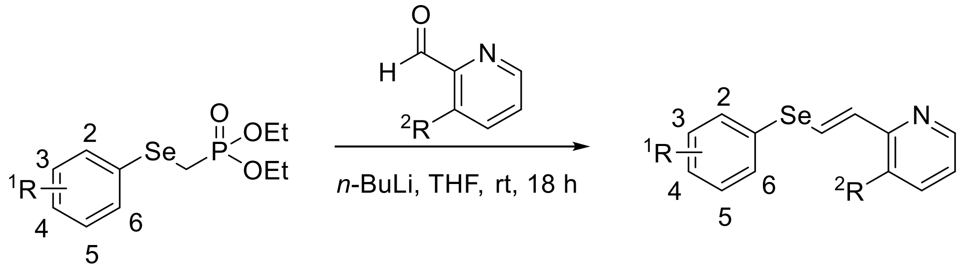

**3a** R^1 = H	**6a** R^1 = 2-OCH_3, R^2 = F	**6l** R^1 = 3-F, R^2 = Cl
**3b** R^1 = 2-OCH_3	**6b** R^1 = 2-OCH_3, R^2 = Cl	**6m** R^1 = 4-F, R^2 = F
**3c** R^1 = 3-OCH_3	**6c** R^1 = 2-OCH_3, R^2 = CF_3	**6n** R^1 = 4-F, R^2 = Cl
**3d** R^1 = 4-OCH_3	**6d** R^1 = 3-OCH_3, R^2 = F	**6o** R^1 = 2-Cl, R^2 = F
**3e** R^1 = 3,4,5-triOCH_3	**6e** R^1 = 3-OCH_3, R^2 = Cl	**6p** R^1 = 2-Cl, R^2 = Cl
**3f** R^1 = 2-F	**6f** R^1 = 4-OCH_3, R^2 = F	**6q** R^1 = 2-Cl, R^2 = CF_3
**3g** R^1 = 3-F	**6g** R^1 = 4-OCH_3, R^2 = Cl	**6r** R^1 = 3-Cl, R^2 = F
**3h** R^1 = 4-F	**6h** R^1 = 2-F, R^2 = F	**6s** R^1 = 3-Cl, R^2 = Cl
**3i** R^1 = 2-Cl	**6i** R^1 = 2-F, R^2 = Cl	**6t** R^1 = 4-Cl, R^2 = F
**3j** R^1 = 3-Cl	**6j** R^1 = 2-F, R^2 = CF_3	**6u** R^1 = 4-Cl, R^2 = Cl
**3k** R^1 = 4-Cl	**6k** R^1 = 3-F, R^2 = F	

Preparation of (*E*)-3-fluoro-2-(2-((2-methoxyphenyl)selanyl)vinyl)pyridine (6a)

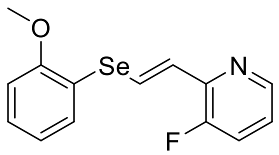

Using Method D, **3b** (0.12 g, 0.38 mmol), 2.0 M *n*-BuLi (0.29 mL, 0.57 mmol) and 3-fluoroplicolinaldehyde (0.072 g, 0.57 mmol) gave 0.048 g (41%) of **6a** as a clear oil; R_f= 0.49 (*n*-hexane:EtOAc = 3:1); ^{1}H NMR (400MHz, DMSO-d_6) δ 8.38 (dt, J = 4.5, 1.4 Hz, 1H), 7.98 (d, J = 15.5 Hz, 1H), 7.71–7.66 (m, 1H), 7.54 (dd, J = 7.6, 1.5 Hz, 1H), 7.39–7.32 (m, 2H), 7.10 (dd, J = 8.2, 1.0 Hz, 1H), 7.03–6.97 (m, 2H), 3.84 (s, 3H).

Preparation of (*E*)-3-chloro-2-(2-((2-methoxyphenyl)selanyl)vinyl)pyridine (6b)

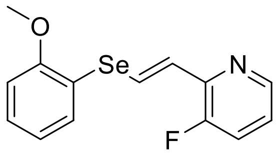

Using Method D, **3b** (0.30 g, 0.89 mmol), 2.0 M *n*-BuLi (0.53 mL, 1.07 mmol) and 3-chloroplicolinaldehyde (0.15 g, 1.07 mmol) gave 0.11 g (39%) of **6b** as a yellow oil; R_f= 0.35 (*n*-hexane:EtOAc = 5:1); ^{1}H NMR (400MHz, $CDCl_3$) δ 8.40 (dd, J = 4.6, 1.3 Hz, 1H), 8.14 (d, J = 15.3 Hz, 1H), 7.59 (dd, J = 8.1, 1.5 Hz, 1H), 7.53 (dd, J = 7.6, 1.5 Hz, 1H), 7.33–7.26 (m, 2H), 7.03 (dd, J = 8.1, 4.6 Hz, 1H), 6.95 (t, J = 7.8 Hz, 1H), 6.90 (d, J = 8.2 Hz, 1H), 3.88 (s, 3H).

Preparation of (*E*)-2-(2-((2-methoxyphenyl)selanyl)vinyl)-3-(trifluoromethyl)pyridine (6c)

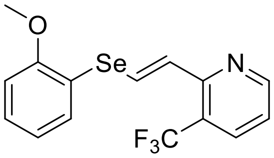

Using Method D, **3b** (0.20 g, 0.59 mmol), 2.0 M *n*-BuLi (0.45 mL, 0.89 mmol) and 3-trifluoromethylplicolinaldehyde (0.11 g, 0.65 mmol) gave 0.05 g (25%) of **6c** as a clear oil; R_f = 0.75 (*n*-hexane:EtOAc = 11:1); ^{1}H NMR (400MHz, $CDCl_3$) δ 8.66 (d, J = 4.3 Hz, 1H), 8.30 (d, J = 14.9 Hz, 1H), 7.85 (d, J = 8.4 Hz, 1H), 7.56 (d, J = 7.7 Hz, 1H), 7.34 (t, J = 7.4 Hz, 1H), 7.56 (dd, J = 7.8, 4.8 Hz, 1H) 7.05–6.91 (m, 3H), 3.89 (s, 3H).

Preparation of (*E*)-3-fluoro-2-(2-((3-methoxyphenyl)selanyl)vinyl)pyridine (6d)

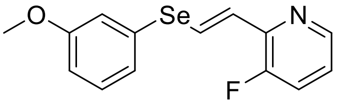

Using Method D, **3c** (0.46 g, 1.36 mmol), 2.0 M *n*-BuLi (1.03 mL, 2.05 mmol) and 3-fluoroplicolinaldehyde (0.26 g, 2.05 mmol) gave 0.09 g (21%) of **6d** as a transparent oil; R_f= 0.58 (*n*-hexane:EtOAc = 4:1); ^{1}H NMR (400MHz, $CDCl_3$) δ 8.32 (d, J = 4.6 Hz, 1H), 8.04 (d,

J = 15.64 Hz, 1H), 7.35–7.23 (m, 2H), 7.20–7.09 (m, 3H), 7.00 (d, J = 15.64 Hz, 1H), 6.88 (dd, J = 8.0 Hz, 2.4 Hz, 1H), 3.82 (s, 3H).

Preparation of (*E*)-3-chloro-2-(2-((3-methoxyphenyl)selanyl)vinyl)pyridine (6e)

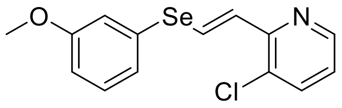

Using Method D, **3c** (0.29 g, 0.88 mmol), 2.0 M *n*-BuLi (0.66 mL, 1.32 mmol) and 3-chloroplicolinaldehyde (0.17 g, 1.32 mmol) gave 0.04 g (15%) of **6e** as a transparent oil; R_f= 0.49 (*n*-hexane:EtOAc = 4:1); ^{1}H NMR (400MHz, $CDCl_3$) δ 8.40 (dd, J = 4.5 Hz, 1.3 Hz, 1H), 8.12 (d, J = 15.28 Hz, 1H), 7.60 (dd, J = 8.06 Hz, 1.48 Hz, 1H), 7.26 (d, J = 1.6 Hz, 1H), 7.25 (d, J = 2.32 Hz, 1H), 7.22–7.17 (m, 2H), 7.05 (t, J = 8.1 Hz, 1H), 6.88 (dd, J = 8.12 Hz, 2.6 Hz, 1H), 3.82 (s, 3H).

Preparation of (*E*)-3-fluoro-2-(2-((4-methoxyphenyl)selanyl)vinyl)pyridine (6f)

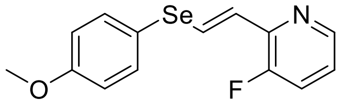

Using Method D, **3d** (0.31 g, 0.91 mmol), 2.0 M *n*-BuLi (0.68 mL, 1.36 mmol) and 3-fluoroplicolinaldehyde (0.17 g, 1.36 mmol) gave 0.06 g (21%) of **6f** as a yellow oil; R_f= 0.58 (*n*-hexane:EtOAc = 4:1); ^{1}H NMR (400MHz, $CDCl_3$) δ 8.29 (d, J = 4.6 Hz, 1H), 8.0 (d, J = 15.56 Hz, 1H), 7.56 (d, J = 8.72 Hz, 2H), 7.30 (m, 1H), 7.09 (m, 1H), 6.90 (d, J = 8.72 Hz, 2H), 6.81 (d, J = 15.6 Hz, 1H), 3.83 (s, 3H).

Preparation of (*E*)-3-chloro-2-(2-((4-methoxyphenyl)selanyl)vinyl)pyridine (6g)

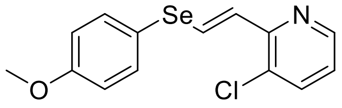

Using Method D, **3d** (0.23 g, 0.68 mmol), 2.0 M *n*-BuLi (0.41 mL, 0.81 mmol) and 3-chloroplicolinaldehyde (0.12 g, 0.81 mmol) gave 0.06 g (26%) of **6g** as a yellow oil; R_f= 0.37 (*n*-hexane:EtOAc = 5:1); ^{1}H NMR (400MHz, $CDCl_3$) δ 8.38 (dd, J = 4.6, 1.4 Hz, 1H), 8.09 (d, J = 15.2 Hz, 1H), 7.61–7.55 (m, 3H), 7.07–7.01 (m, 2H), 6.92–6.88 (m, 3H), 3.83 (s, 3H).

Preparation of (*E*)-3-fluoro-2-(2-((2-fluorophenyl)selanyl)vinyl)pyridine (6h)

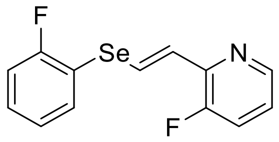

Using Method D, **3f** (0.30 g, 0.92 mmol), 2.0 M *n*-BuLi (0.55 mL, 1.10 mmol) and 3-fluoroplicolinaldehyde (0.14 g, 1.10 mmol) gave 0.09 g (32%) of **6h** as a yellow oil; R_f= 0.30 (*n*-hexane:EtOAc = 4:1); ^{1}H NMR (400MHz, $CDCl_3$) δ 8.33 (d, J = 4.5 Hz, 1H), 7.96 (d, J = 15.6 Hz, 1H), 7.61 (t, J = 7.8 Hz, 1H), 7.38–7.30 (m, 2H), 7.17–7.10 (m, 3H), 7.03 (d, J = 15.5 Hz, 1H).

Preparation of (*E*)-3-chloro-2-(2-((2-fluorophenyl)selanyl)vinyl)pyridine (6i)

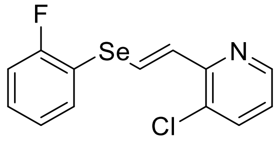

Using Method D, **3f** (0.30 g, 0.92 mmol), 2.0 M *n*-BuLi (0.55 mL, 1.10 mmol) and 3-chloroplicolinaldehyde (0.16 g, 1.10 mmol) gave 0.10 g (34%) of **6i** as a yellow oil; R_f= 0.37 (*n*-hexane:EtOAc = 4:1); ^{1}H NMR (400MHz, $CDCl_3$) δ 8.41 (d, J = 4.5 Hz, 1H), 8.04 (d, J = 15.2 Hz, 1H), 7.64–7.58 (m, 2H), 7.35 (q, J = 6.8 Hz, 1H), 7.25 (d, J = 15.2 Hz, 1H), 7.17–7.11 (m, 2H), 7.06 (dd, J = 8.0, 4.6 Hz, 1H).

Preparation of (*E*)-2-(2-((2-fluorophenyl)selanyl)vinyl)-3-(trifluoromethyl)pyridine (6j)

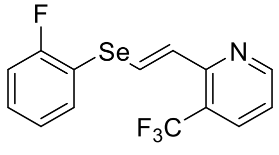

Using Method D, **3f** (0.30 g, 0.92 mmol), 2.0 M *n*-BuLi (0.55 mL, 1.10 mmol) and 3-trifluoromethylplicolinaldehyde (0.19 g, 1.10 mmol) gave 0.09 g (28%) of **6j** as a yellow oil; R_f= 0.36 (*n*-hexane:EtOAc = 4:1); ^{1}H NMR (400MHz, $CDCl_3$) δ 8.65 (d, J = 4.1 Hz, 1H), 8.20 (d, J = 15.0 Hz, 1H), 7.86 (d, J = 7.9 Hz, 1H), 7.62 (t, J = 6.8 Hz, 1H), 7.38 (q, J = 6.9 Hz, 1H), 7.22–7.12 (m, 3H), 6.98 (d, J = 15.0 Hz, 1H).

Preparation of (*E*)-3-fluoro-2-(2-((3-fluorophenyl)selanyl)vinyl)pyridine (6k)

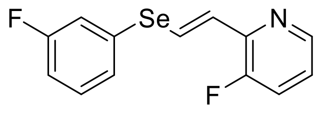

Using Method D, **3g** (0.30 g, 0.92 mmol), 2.0 M *n*-BuLi (0.55 mL, 1.10 mmol) and 3-

fluoroplicolinaldehyde (0.14 g, 1.10 mmol) gave 0.06 g (23%) of **6k** as a yellow oil; R_f= 0.59 (*n*-hexane:EtOAc = 5:1); ^{1}H NMR (400MHz, $CDCl_3$) δ 8.34 (d, J = 4.3 Hz, 1H), 8.00 (d, J = 15.6 Hz, 1H), 7.43–6.99 (m, 7H).

Preparation of (*E*)-3-chloro-2-(2-((3-fluorophenyl)selanyl)vinyl)pyridine (6l)

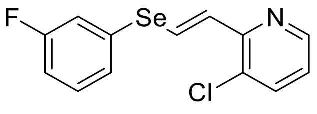

Using Method D, **3g** (0.30 g, 0.92 mmol), 2.0 M *n*-BuLi (0.55 mL, 1.10 mmol) and 3-chloroplicolinaldehyde (0.16 g, 1.10 mmol) gave 0.02 g (6%) of **6l** as a yellow oil; R_f= 0.61 (*n*-hexane:EtOAc = 5:1); ^{1}H NMR (400MHz, $CDCl_3$) δ 8.33 (dd, J = 4.5, 1.2 Hz, 1H), 8.08 (d, J = 15.3 Hz, 1H), 7.62 (dd, J = 8.2, 1.4 Hz, 1H), 7.40–7.21 (m, 4H), 7.10–7.00 (m, 2H).

Preparation of (*E*)-3-fluoro-2-(2-((4-fluorophenyl)selanyl)vinyl)pyridine (6m)

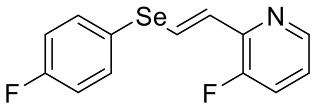

Using Method D, **3h** (0.30 g, 0.92 mmol), 2.0 M *n*-BuLi (0.55 mL, 1.10 mmol) and 3-fluoroplicolinaldehyde (0.14 g, 1.10 mmol) gave 0.09 g (34%) of **6m** as a yellow oil; R_f= 0.63 (*n*-hexane:EtOAc = 5:1); ^{1}H NMR (400MHz, $CDCl_3$) δ 8.33 (dd, J = 4.5, 1.2 Hz, 1H), 8.08 (d, J = 15.3 Hz, 1H), 7.62 (dd, J = 8.2, 1.4 Hz, 1H), 7.40–7.21 (m, 4H), 7.10–7.00 (m, 2H).

Preparation of (*E*)-3-chloro-2-(2-((4-fluorophenyl)selanyl)vinyl)pyridine (6n)

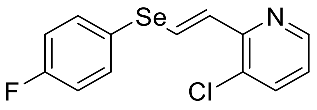

Using Method D, **3h** (0.30 g, 0.92 mmol), 2.0 M *n*-BuLi (0.55 mL, 1.10 mmol) and 3-chloroplicolinaldehyde (0.16 g, 1.10 mmol) gave 0.09 g (28%) of **6n** as a yellow oil; R_f= 0.64 (*n*-hexane:EtOAc = 5:1); ^{1}H NMR (400MHz, $CDCl_3$) δ 8.39 (d, J = 4.7 Hz, 1H), 7.96 (d, J = 15.1 Hz, 1H), 7.65–7.50 (m, 3H), 7.10–6.97 (m, 4H).

Preparation of (*E*)-2-(2-((2-chlorophenyl)selanyl)vinyl)-3-fluoropyridine (6o)

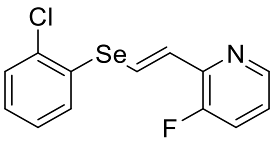

Using Method D, **3i** (0.26 g, 0.77 mmol), 2.0 M *n*-BuLi (0.58 mL, 1.15 mmol) and 3-fluoroplicolinaldehyde (0.14 g, 1.15 mmol) gave 0.13 g (52%) of **6o** as a yellow oil; R_f= 0.56 (*n*-hexane:EtOAc = 3:1); ^{1}H NMR (400MHz, DMSO-d_6) δ 8.41 (d, J = 4.5 Hz, 1H), 7.98 (d, J = 15.4 Hz, 1H), 7.73–7.68 (m, 1H), 7.63–7.58 (m, 2H), 7.42–7.35 (m, 3H), 7.07 (dd, J = 15.4, 1.0 Hz, 1H).

Preparation of (*E*)-3-chloro-2-(2-((2-chlorophenyl)selanyl)vinyl)pyridine (6p)

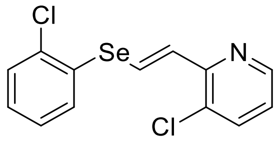

Using Method D, **3i** (0.30 g, 0.88 mmol), 2.0 M *n*-BuLi (0.66 mL, 1.32 mmol) and 3-chloroplicolinaldehyde (0.19 g, 1.32 mmol) gave 0.075 g (26%) of **6p** as an ivory solid; R_f= 0.56 (*n*-hexane:EtOAc = 3:1); ^{1}H NMR (400MHz, DMSO-d_6) δ 8.51 (dd, J = 4.5, 1.4 Hz, 1H), 8.07 (d, J = 15.2 Hz, 1H), 7.91 (dd, J = 8.1, 1.4 Hz, 1H), 7.64–7.62 (m, 1H), 7.61–7.59 (m, 1H), 7.43–7.39 (m, 2H), 7.32 (dd, J = 8.1, 4.5 Hz, 1H), 7.23 (d, J = 15.2 Hz, 1H).

Preparation of (*E*)-2-(2-((2-chlorophenyl)selanyl)vinyl)-3-(trifluoromethyl)pyridine (6q)

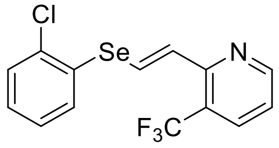

Using Method D, **3i** (0.15 g, 0.44 mmol), 2.0 M *n*-BuLi (0.33 mL, 0.67 mmol) and 3-trifluoromethylplicolinaldehyde (0.11 g, 0.67 mmol) gave 0.097 g (51%) of **6q** as a yellow oil; R_f= 0.55 (*n*-hexane:EtOAc = 3:1); ^{1}H NMR (400MHz, DMSO-d_6) δ 8.79 (d, J = 4.5 Hz, 1H), 8.28 (d, J = 14.8 Hz, 1H), 8.10 (dd, J = 8.0 Hz, 1H), 7.69 (dd, J = 7.5, 1.7 Hz, 1H), 7.63 (dd, J = 7.8, 1.5 Hz, 1H), 7.48–7.39 (m, 3H), 6.84 (dq, J = 14.8, 1.9 Hz, 1H).

Preparation of (*E*)-2-(2-((3-chlorophenyl)selanyl)vinyl)-3-fluoropyridine (6r)

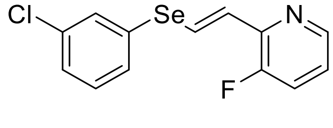

Using Method D, **3j** (0.24 g, 0.71 mmol), 2.0 M *n*-BuLi (0.71 mL, 1.43 mmol) and 3-fluoroplicolinaldehyde (0.13 g, 1.07 mmol) gave 0.061 g (27%) of **6r** as a clear oil; R_f= 0.29 (*n*-hexane:EtOAc = 10:1); ^{1}H NMR (400MHz, DMSO-d_6) δ 8.38 (dd, J = 3.1, 1.1 Hz, 1H), 8.00 (d, J = 15.4 Hz, 1H), 7.72–7.68 (m, 2H), 7.58 (dt, J = 7.0, 1.7 Hz, 1H), 7.50–7.43 (m, 2H), 7.35 (q, J = 4.4 Hz, 1H), 6.97 (dd, J = 15.4, 0.9 Hz, 1H).

Preparation of (*E*)-3-chloro-2-(2-((3-chlorophenyl)selanyl)vinyl)pyridine (6s)

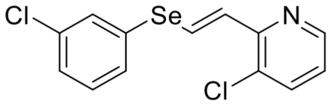

Using Method D, **3j** (0.37 g, 1.09 mmol), 2.0 M *n*-BuLi (0.82 mL, 1.64 mmol) and 3-chloroplicolinaldehyde (0.23 g, 1.64 mmol) gave 0.071 g (20%) of **6s** as a clear oil; R_f= 0.36 (*n*-hexane:EtOAc = 10:1); ^{1}H NMR (400MHz, DMSO-d_6) δ 8.49 (dd, J = 4.3, 1.1 Hz, 1H), 8.08 (d, J = 15.1 Hz, 1H), 7.89 (dd, J = 8.1, 1.3 Hz, 1H), 7.70–7.69 (m, 1H), 7.59 (dt, J = 7.0, 1.7 Hz, 1H), 7.50–7.44 (m, 2H), 7.29 (dd, J = 8.1, 4.5 Hz, 1H), 7.15 (d, J = 15.2 Hz, 1H).

Preparation of (*E*)-2-(2-((4-chlorophenyl)selanyl)vinyl)-3-fluoropyridine (6t)

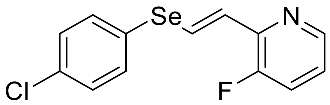

Using Method D, **3k** (0.20 g, 0.61 mmol), 2.0 M *n*-BuLi (0.45 mL, 0.91 mmol) and 3-fluoroplicolinaldehyde (0.11 g, 0.91 mmol) gave 0.084 g (44%) of **6t** as a light yellow oil; R_f= 0.30 (*n*-hexane:EtOAc = 9:1); ^{1}H NMR (400MHz, DMSO-d_6) δ 8.37 (d, J = 4.4 Hz, 1H), 7.98 (d, J = 15.5 Hz, 1H), 7.70–7.62 (m, 3H), 7.49 (d, J = 8.5 Hz, 2H), 7.33 (q, J = 4.4 Hz, 1H), 6.90 (dd, J = 15.5, 0.8 Hz, 1H).

Preparation of (*E*)-3-chloro-2-(2-((4-chlorophenyl)selanyl)vinyl)pyridine (6u)

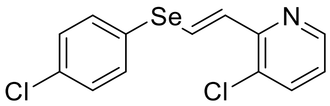

Using Method D, **3k** (0.22 g, 0.67 mmol), 2.0 M *n*-BuLi (0.50 mL, 1.00 mmol) and 3-chloroplicolinaldehyde (0.14 g, 1.00 mmol) gave 0.087 g (39%) of **6u** as a clear oil; R_f= 0.38 (*n*-hexane:EtOAc = 9:1); ^{1}H NMR (400MHz, DMSO-d_6) δ 8.48 (dd, J = 4.6, 0.8 Hz, 1H), 8.07 (d, J = 15.2 Hz, 1H), 7.88 (dd, J = 8.1, 1.1 Hz, 1H), 7.64 (d, J = 8.4 Hz, 2H), 7.50 (d, J = 8.4 Hz, 2H), 7.28 (dd, J = 8.1, 4.5 Hz, 1H), 7.10 (d, J = 15.6 Hz, 1H).

3. ^{1}H and ^{13}C-NMR Spectra for the Final Compounds

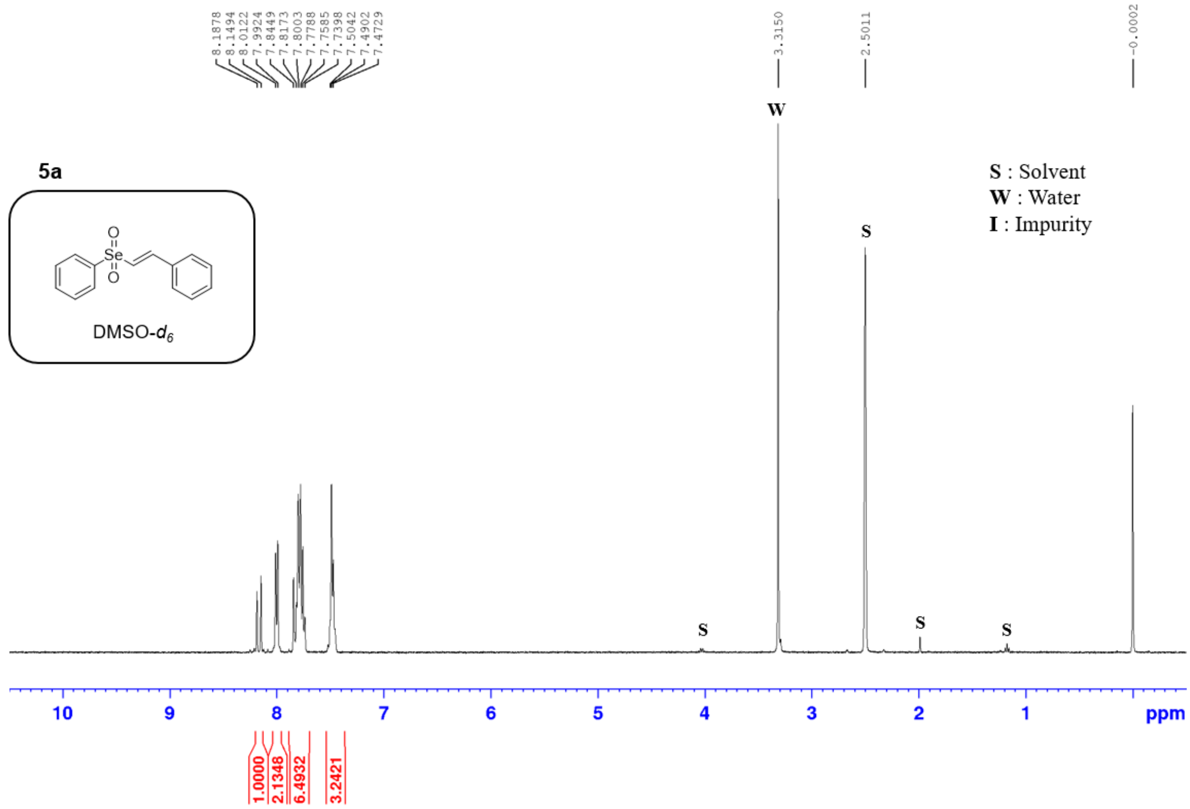

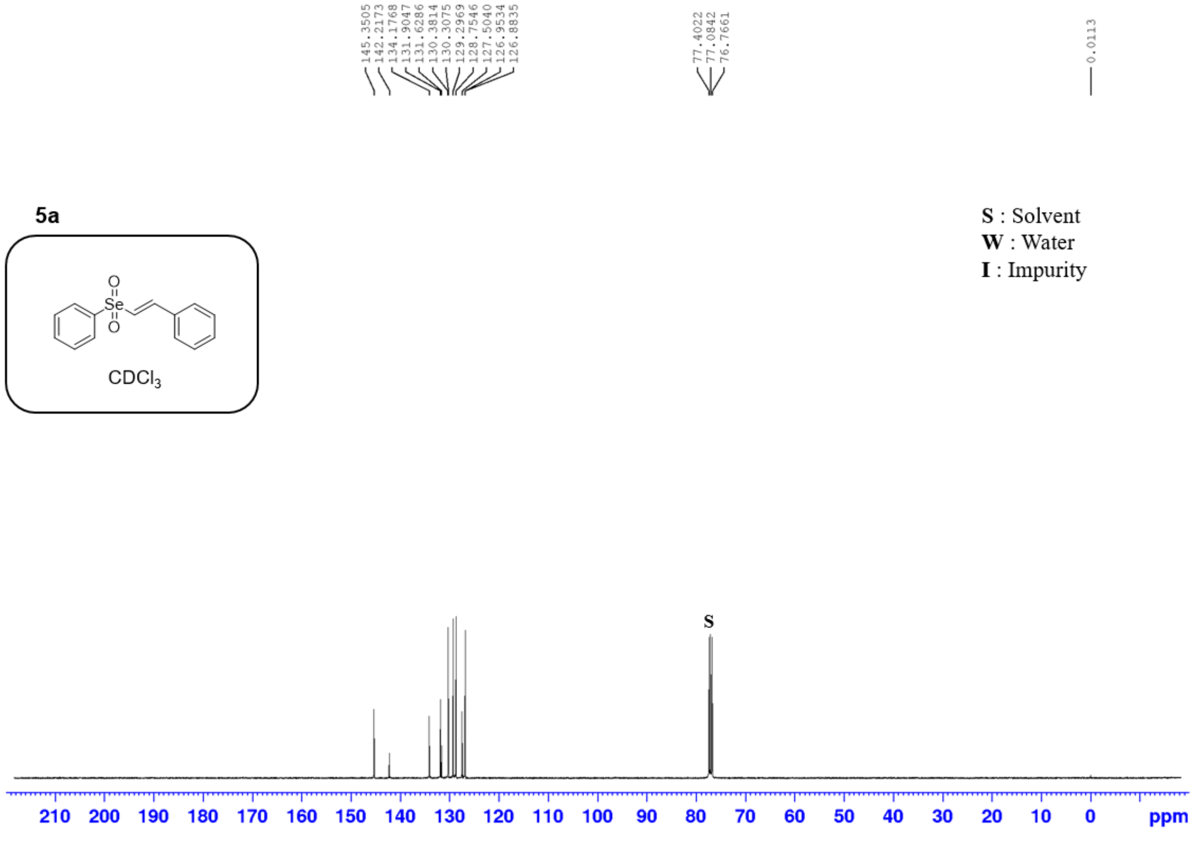

5b

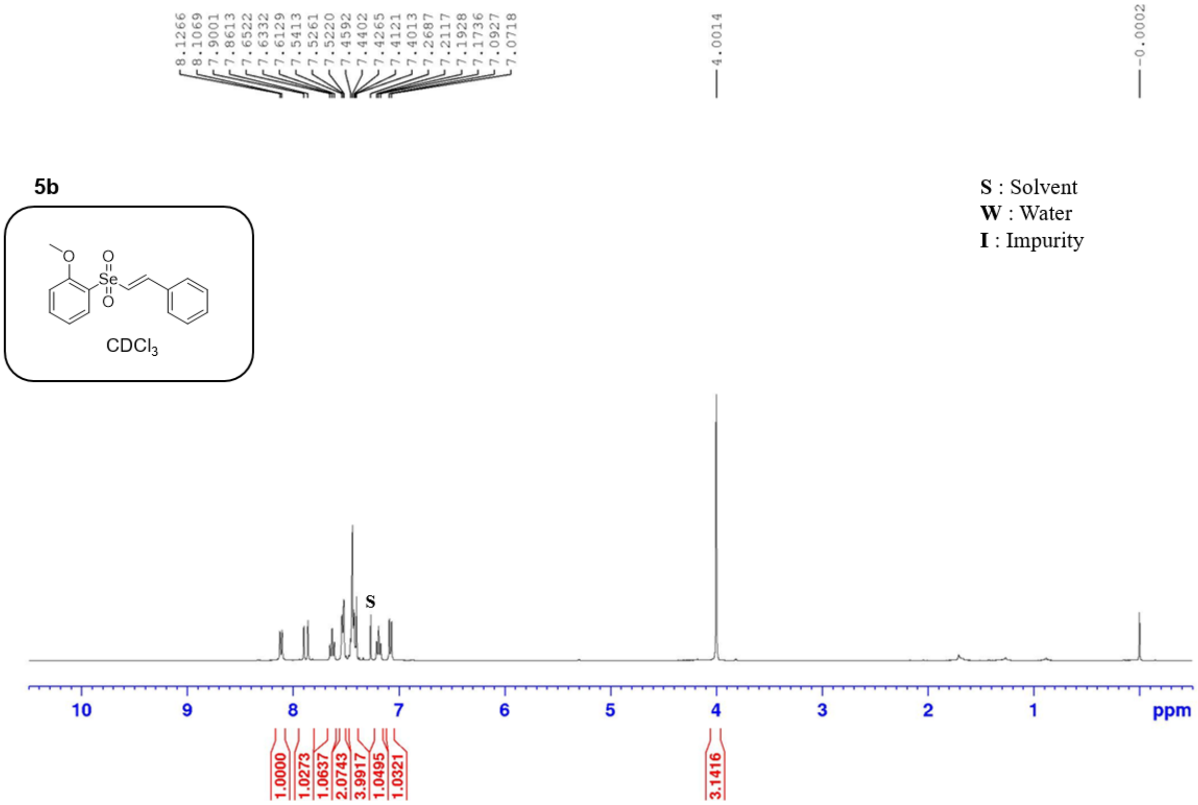
8.1266
8.1069
7.9001
7.8613
7.6522
7.6332
7.6129
7.5413
7.5261
7.5220
7.4592
7.4402
7.4265
7.4121
7.4013
7.2687
7.2117
7.1928
7.1736
7.0927
7.0718
4.0014
-0.0002
S : Solvent
W : Water
I : Impurity
Se
O
CDCl3
S
10
9
8
7
6
5
4
3
2
1
ppm
1.0000
1.0273
1.0637
2.0743
3.9917
1.0495
1.0321
3.1416

5b

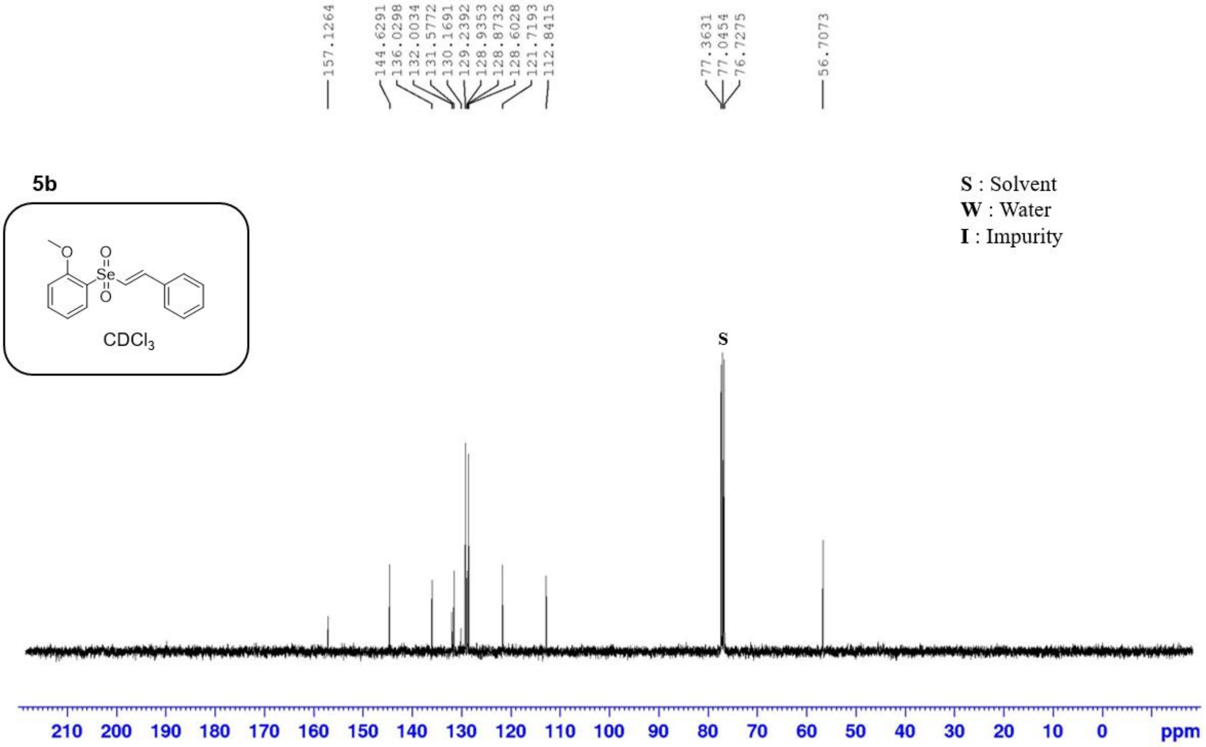
157.1264
144.6291
136.0298
132.0034
131.5772
130.1691
129.2392
128.9353
128.8732
128.6028
121.7193
112.8415
77.3631
77.0454
76.7275
56.7073
S : Solvent
W : Water
I : Impurity
Se
O
CDCl3
S
210
200
190
180
170
160
150
140
130
120
110
100
90
80
70
60
50
40
30
20
10
0
ppm

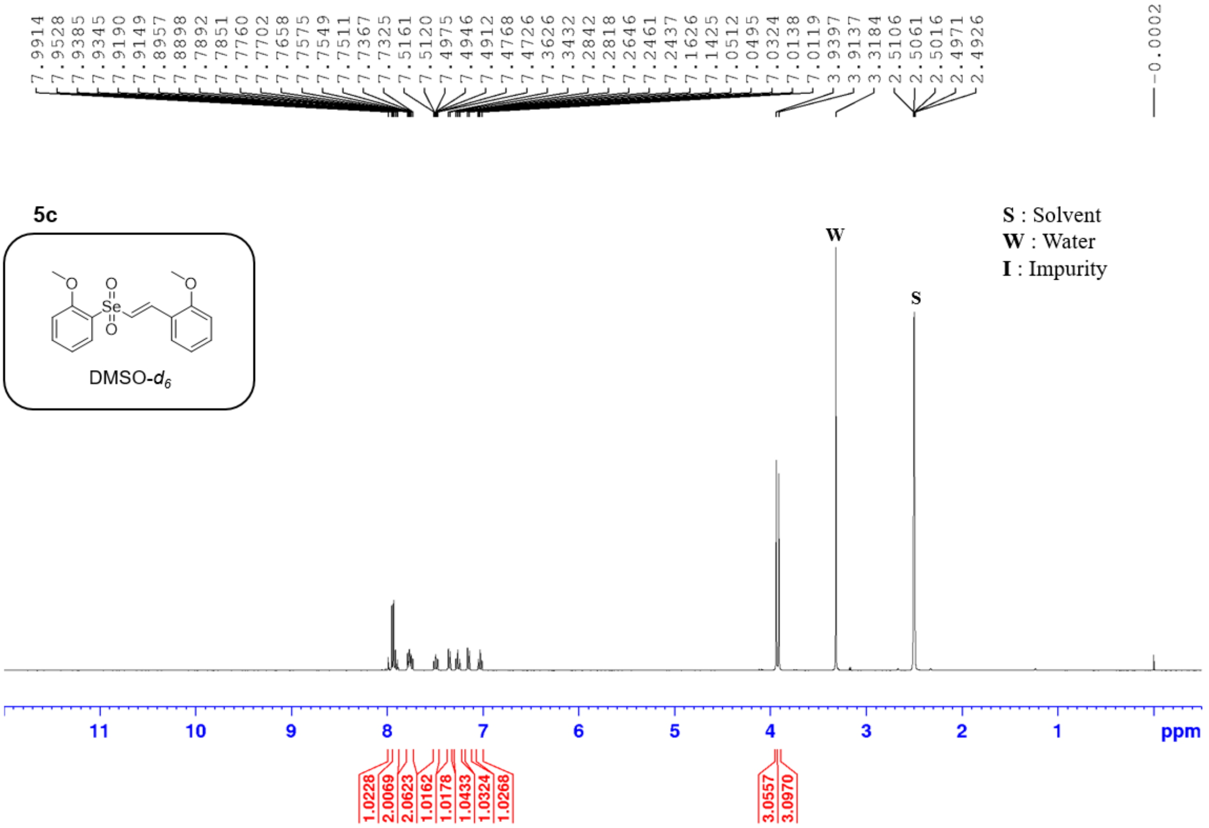

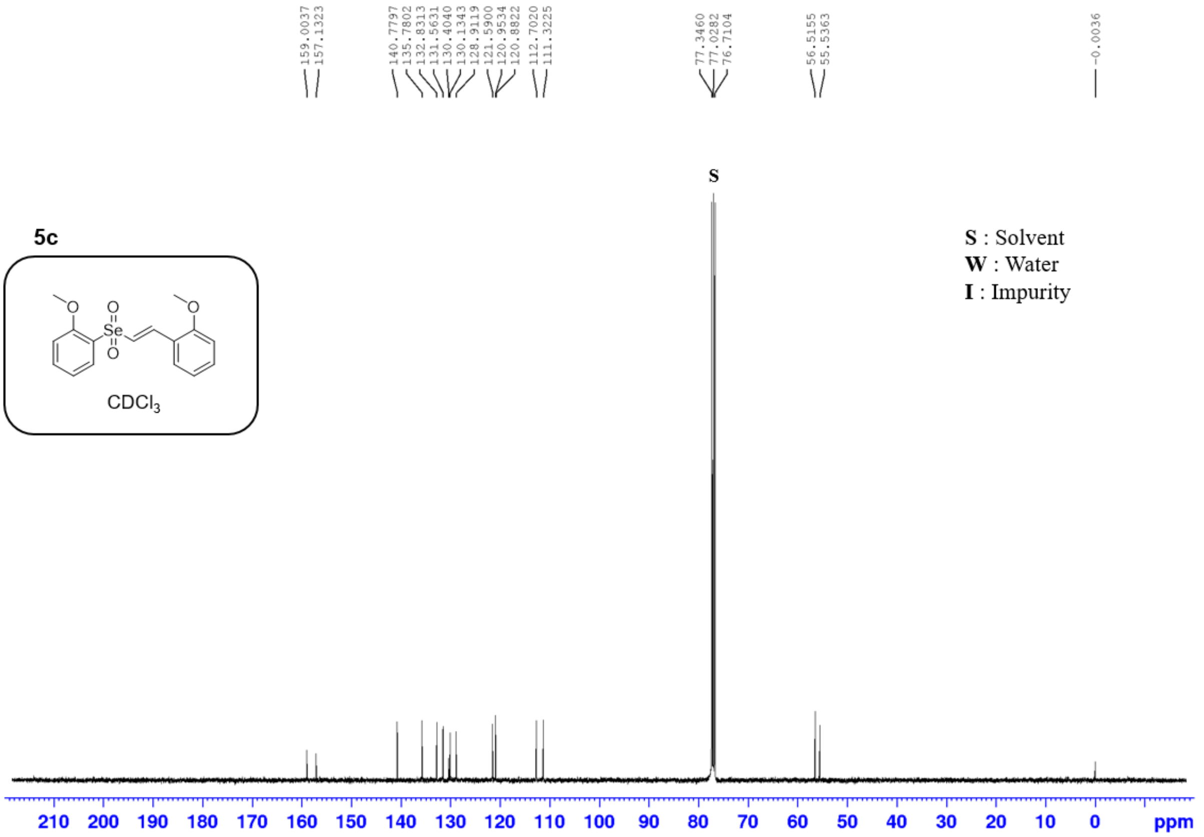

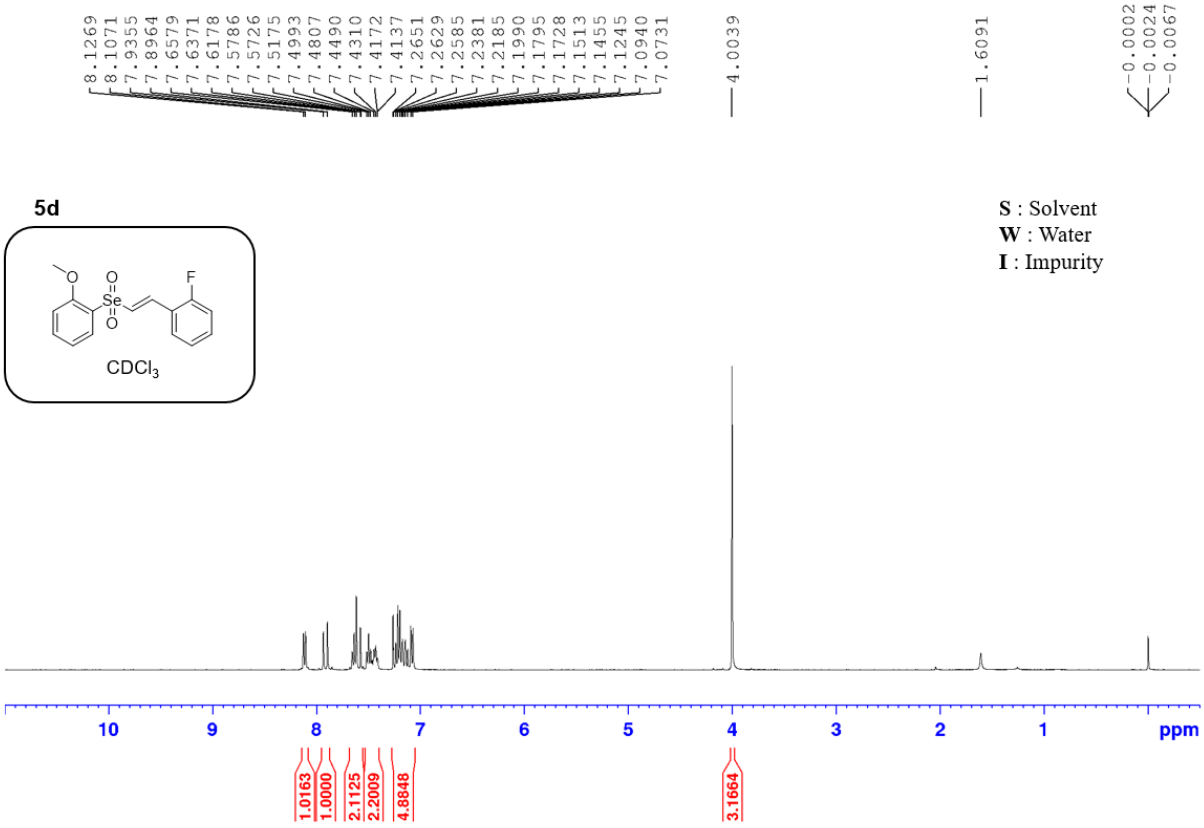
8.1269
8.1071
7.9355
7.8964
7.6579
7.6371
7.6178
7.5786
7.5726
7.5175
7.4993
7.4807
7.4490
7.4310
7.4172
7.4137
7.2651
7.2629
7.2585
7.2381
7.2185
7.1990
7.1795
7.1728
7.1513
7.1455
7.1245
7.0940
7.0731
4.0039
1.6091
-0.0002
-0.0024
-0.0067
5d
Se
O
O
O
F
CDCl3
S : Solvent
W : Water
I : Impurity
1.0163
1.0000
2.1125
2.2009
4.8848
3.1664
ppm

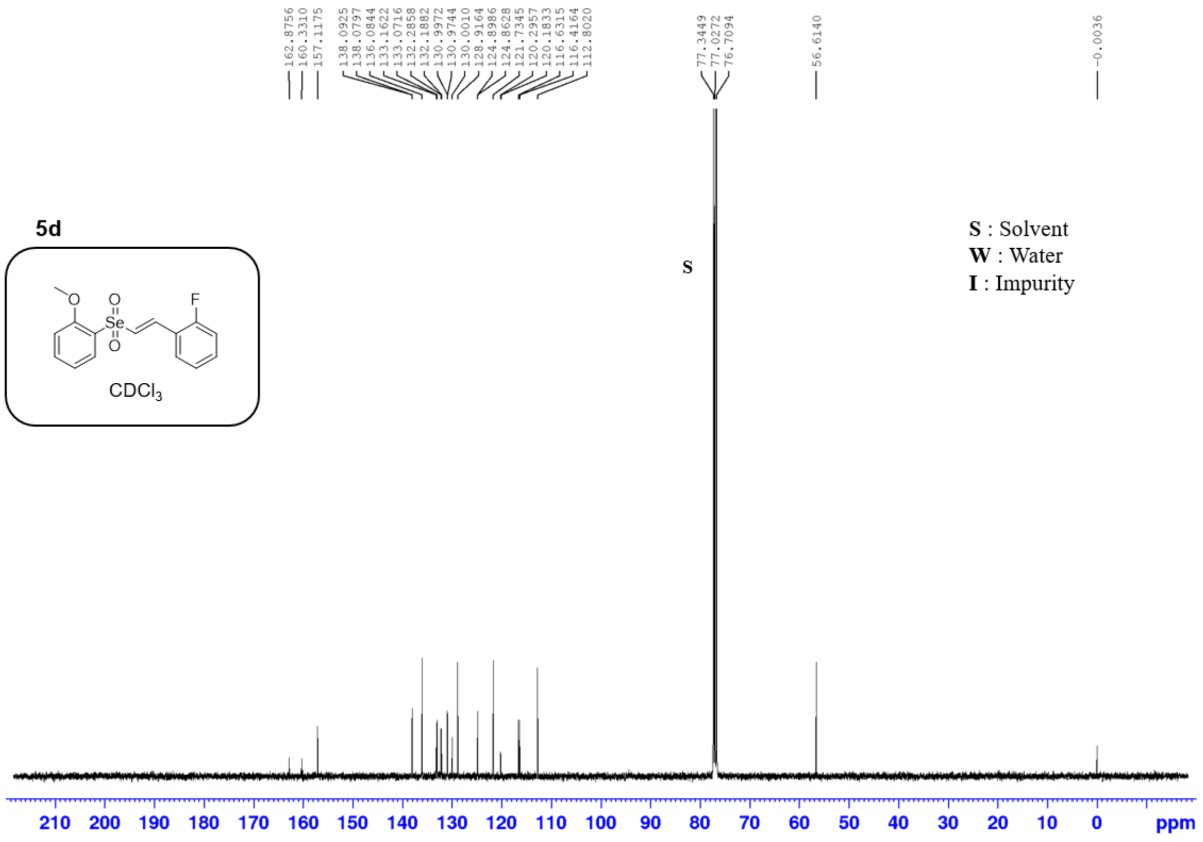
162.8756
160.3310
157.1175
138.0925
138.0797
136.0844
133.1622
133.0716
132.2858
132.1882
130.9972
130.9744
130.0010
128.9164
124.8986
124.8628
121.7345
120.2957
120.1833
116.6315
116.4164
112.8020
77.3449
77.0272
76.7094
56.6140
-0.0036
5d
Se
O
O
O
F
CDCl3
S
S : Solvent
W : Water
I : Impurity
ppm

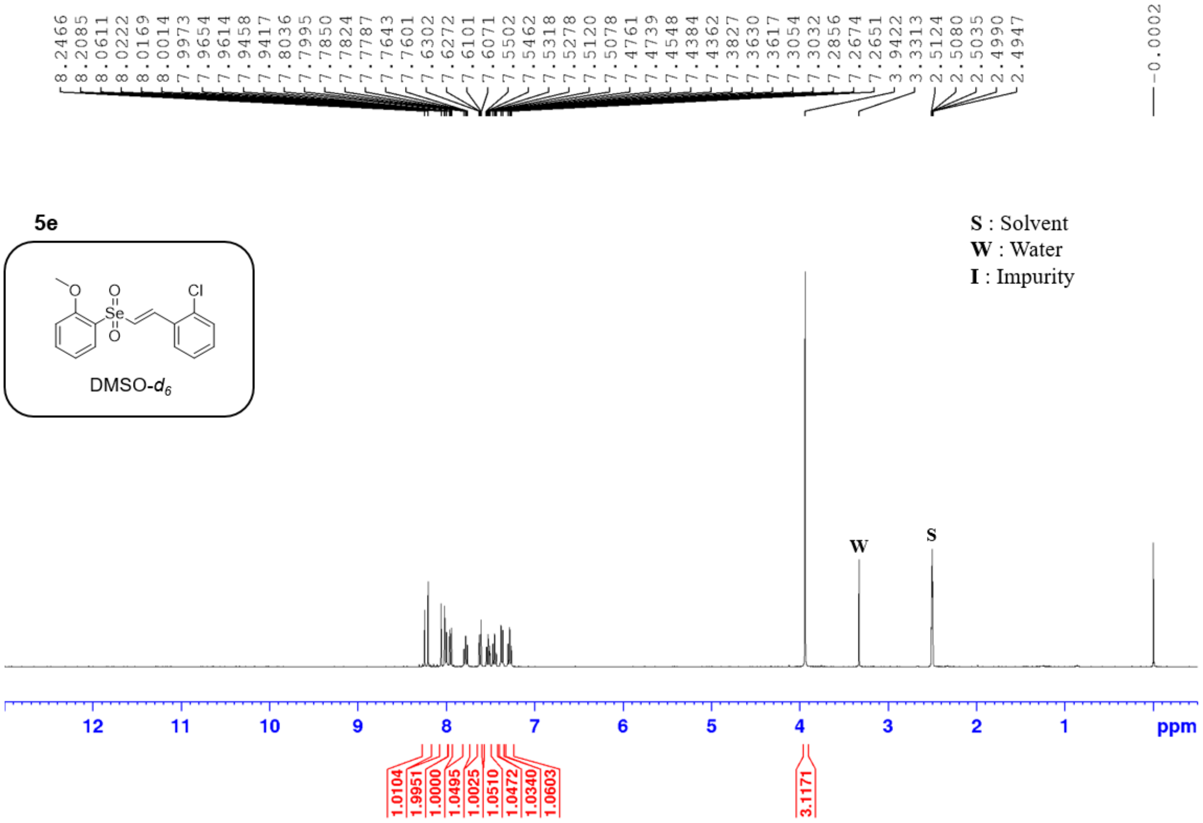
5e
DMSO-d_6
S : Solvent
W : Water
I : Impurity
W
S
ppm

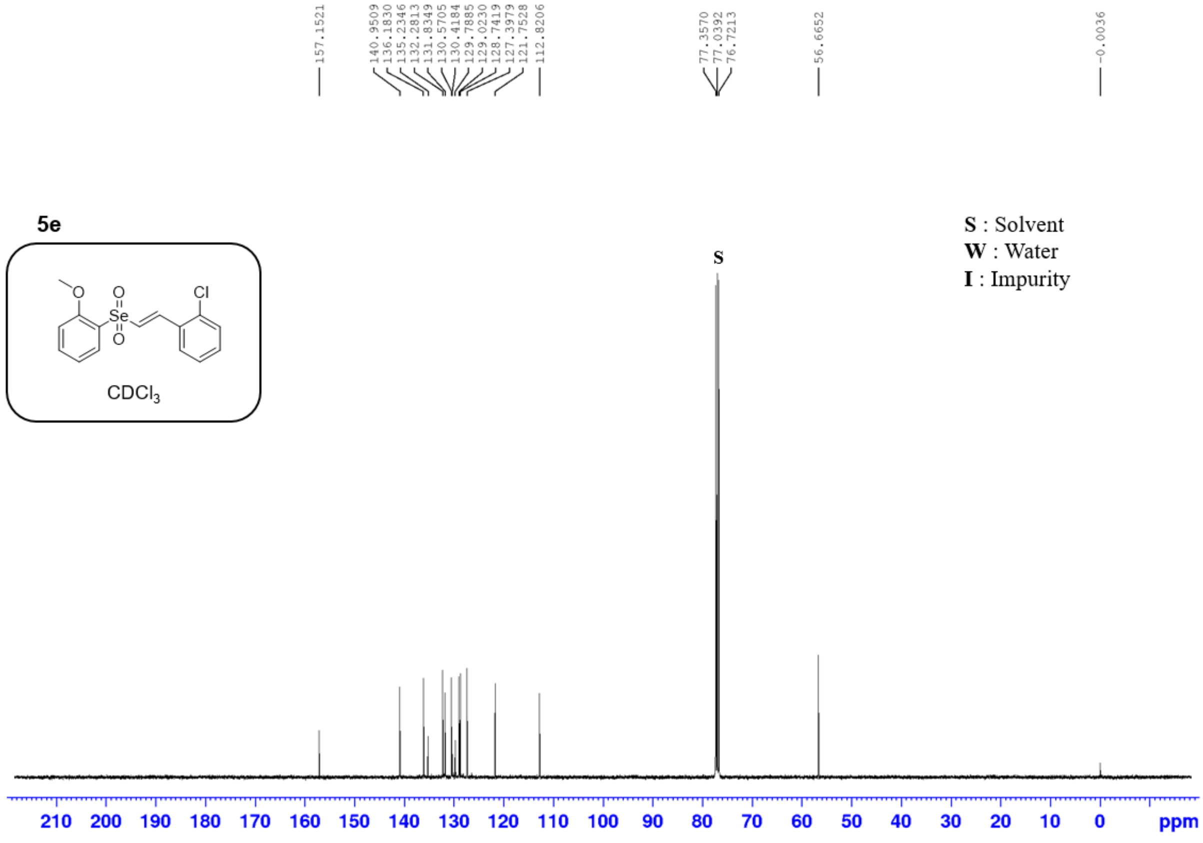
5e
CDCl$_3$
S : Solvent
W : Water
I : Impurity
S
ppm

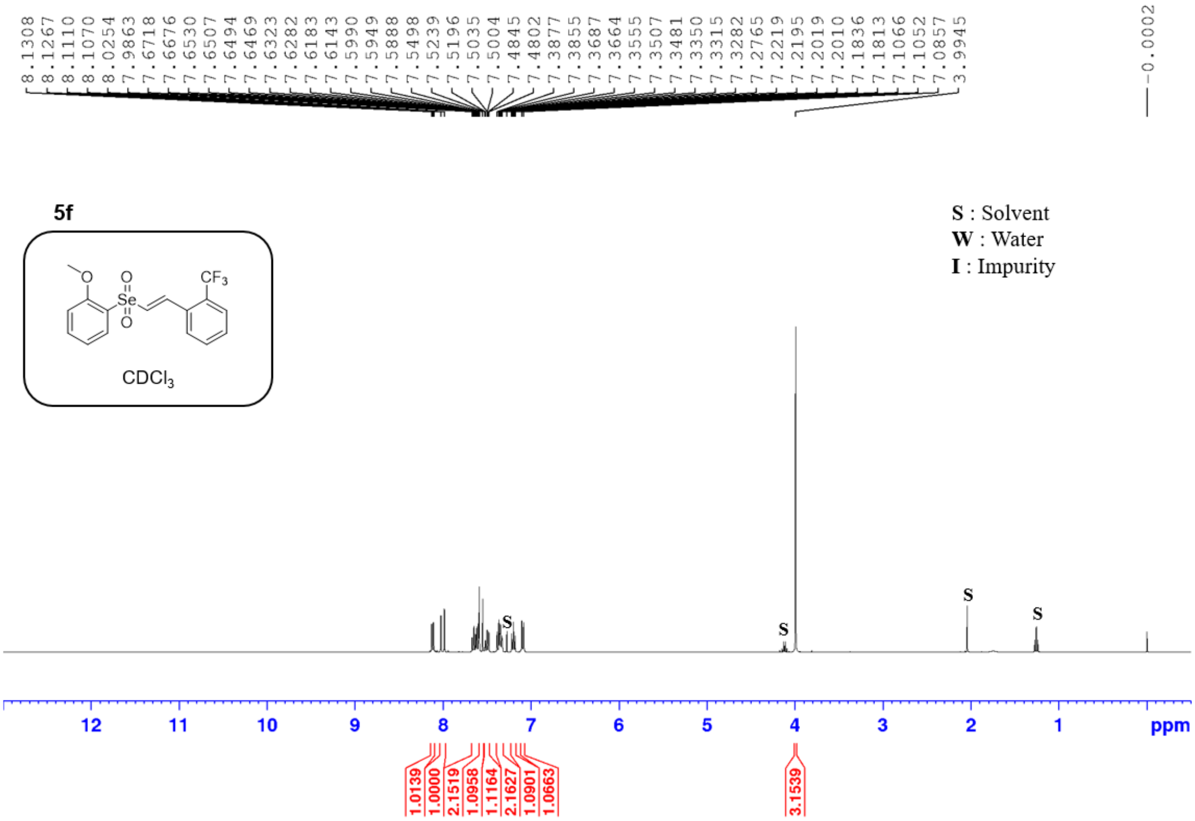
8.1308
8.1267
8.1110
8.1070
8.0254
7.9863
7.6718
7.6676
7.6530
7.6507
7.6494
7.6469
7.6323
7.6282
7.6183
7.6143
7.5990
7.5949
7.5888
7.5498
7.5239
7.5196
7.5035
7.5004
7.4845
7.4802
7.3877
7.3855
7.3687
7.3664
7.3555
7.3507
7.3481
7.3350
7.3315
7.3282
7.2765
7.2219
7.2195
7.2019
7.2010
7.1836
7.1813
7.1066
7.1052
7.0857
3.9945
-0.0002
5f
CDCl3
S : Solvent
W : Water
I : Impurity
S
S
S
S
12
11
10
9
8
7
6
5
4
3
2
1
ppm
1.0139
1.0000
2.1519
1.0958
1.1164
2.1627
1.0901
1.0663
3.1539

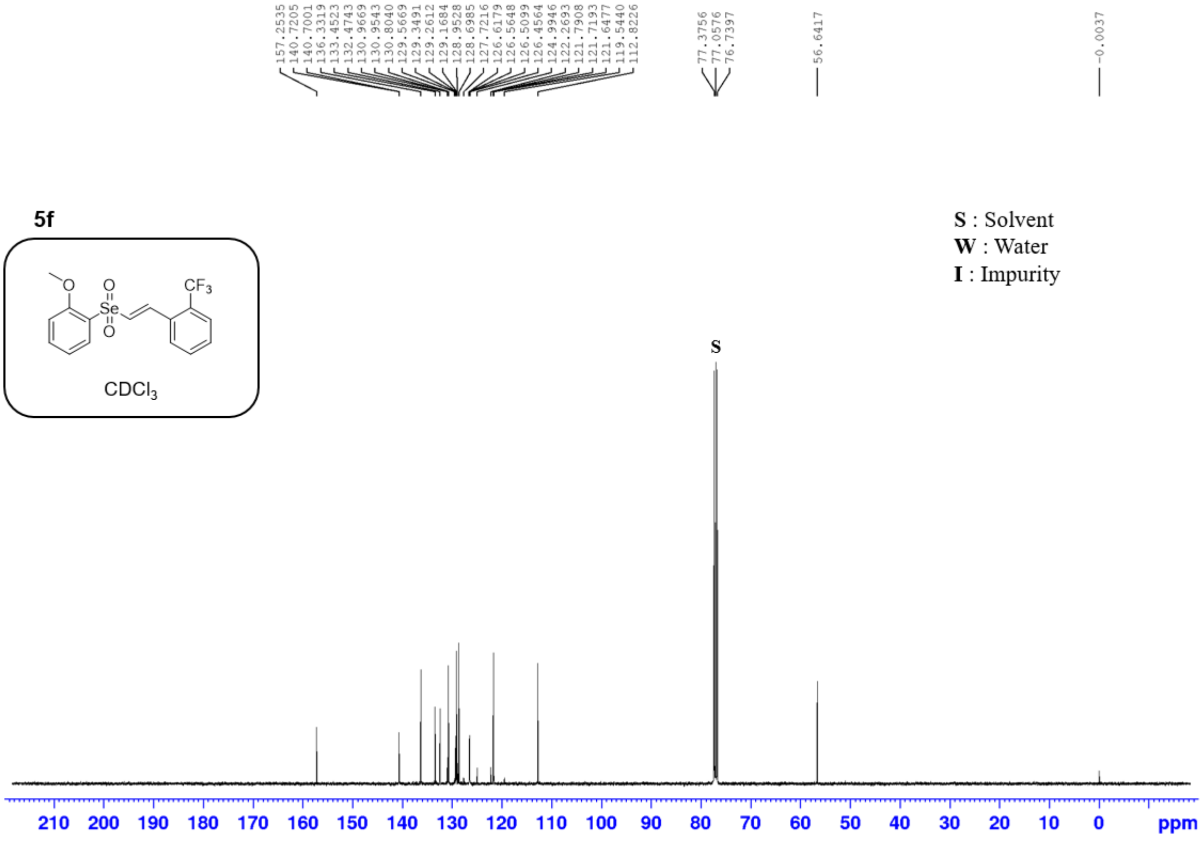
157.2535
140.7205
140.7001
136.3319
133.4523
132.4743
130.9669
130.9543
130.8040
129.5669
129.3491
129.2612
129.1684
128.9528
128.6985
127.7216
126.6179
126.5648
126.5099
126.4564
124.9946
122.2693
121.7908
121.7193
121.6477
119.5440
112.8226
77.3756
77.0576
76.7397
56.6417
-0.0037
5f
CDCl3
S : Solvent
W : Water
I : Impurity
S
210
200
190
180
170
160
150
140
130
120
110
100
90
80
70
60
50
40
30
20
10
0
ppm

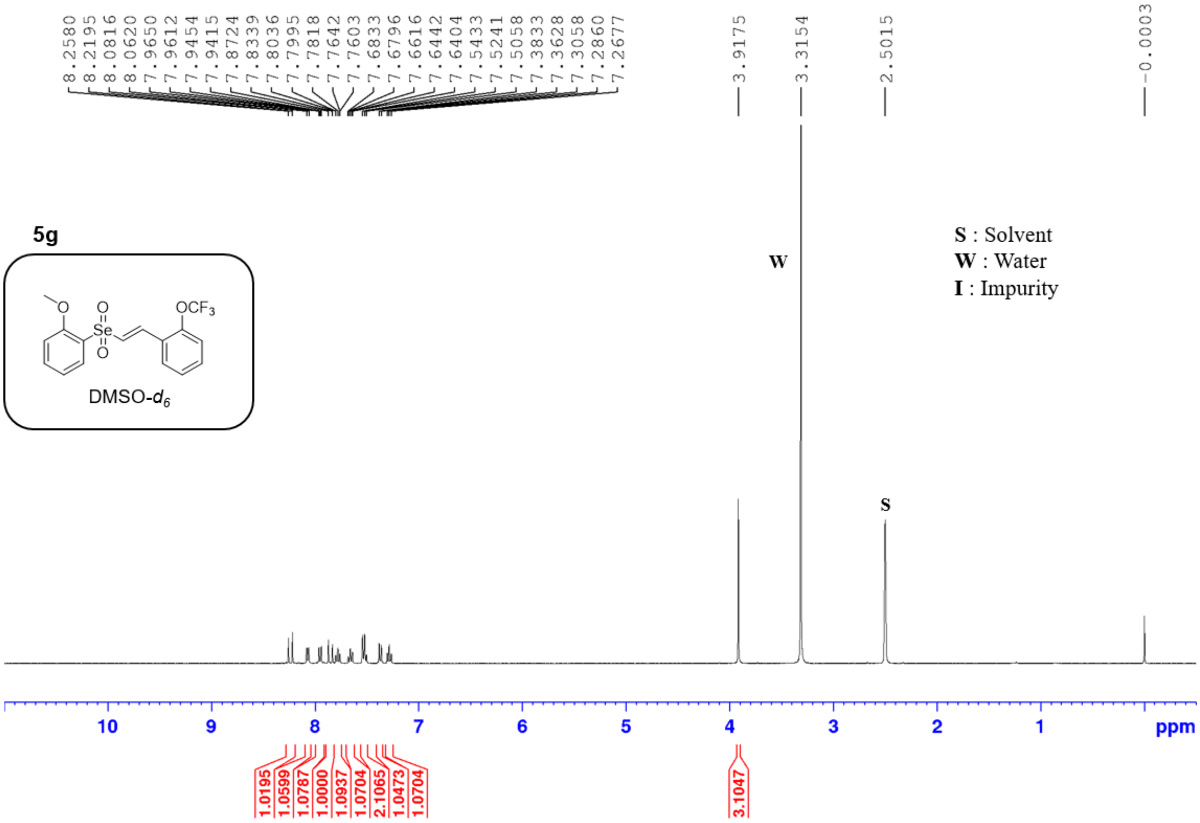
8.2580
8.2195
8.0816
8.0620
7.9650
7.9612
7.9454
7.9415
7.8724
7.8339
7.8036
7.7995
7.7818
7.7642
7.7603
7.6833
7.6796
7.6616
7.6442
7.6404
7.5433
7.5241
7.5058
7.3833
7.3628
7.3058
7.2860
7.2677
3.9175
3.3154
2.5015
-0.0003
5g
OCF3
Se
O
DMSO-d6
W
S
S : Solvent
W : Water
I : Impurity
10
9
8
7
6
5
4
3
2
1
ppm
1.0195
1.0599
1.0787
1.0000
1.0937
1.0704
2.1065
1.0473
1.0704
3.1047

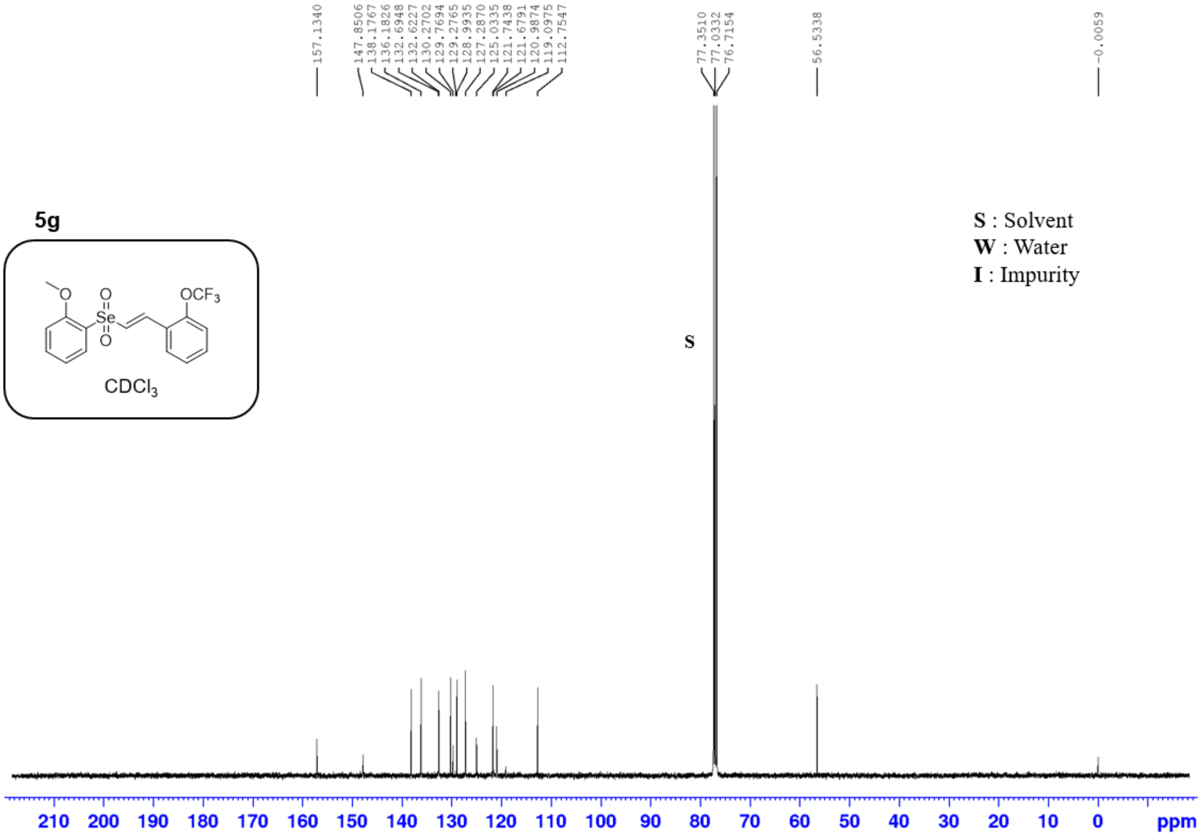
157.1340
147.8506
138.1767
136.1826
132.6948
132.6227
130.2702
129.7694
129.2765
128.9935
127.2870
125.0335
121.7438
121.6791
120.9874
119.0975
112.7547
77.3510
77.0332
76.7154
56.5338
-0.0059
5g
OCF3
Se
O
CDCl3
S
S : Solvent
W : Water
I : Impurity
210
200
190
180
170
160
150
140
130
120
110
100
90
80
70
60
50
40
30
20
10
0
ppm

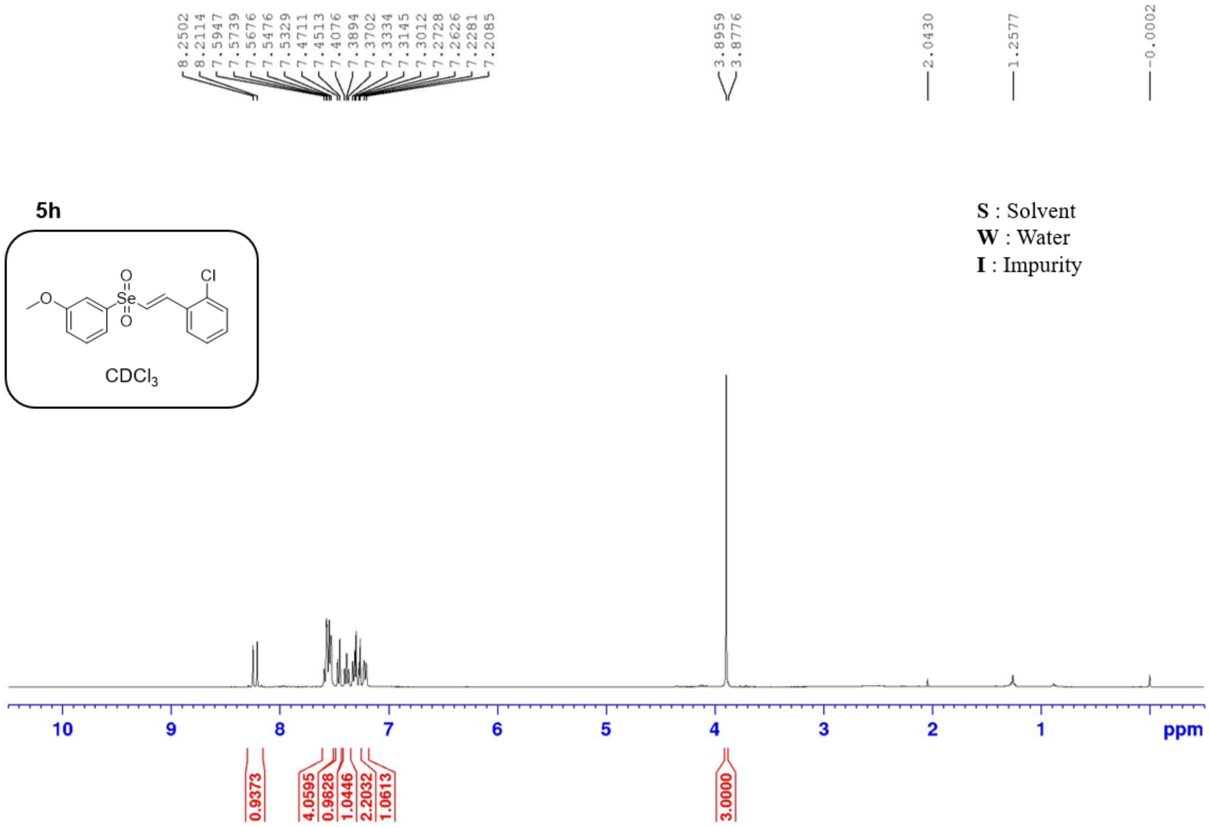
8.2502
8.2114
7.5947
7.5739
7.5676
7.5476
7.5329
7.4711
7.4513
7.4076
7.3894
7.3702
7.3334
7.3145
7.3012
7.2728
7.2626
7.2281
7.2085
3.8959
3.8776
2.0430
1.2577
-0.0002
5h
CDCl3
S : Solvent
W : Water
I : Impurity
10 9 8 7 6 5 4 3 2 1 ppm
0.9373
4.0595
0.9828
1.0446
2.2032
1.0613
3.0000

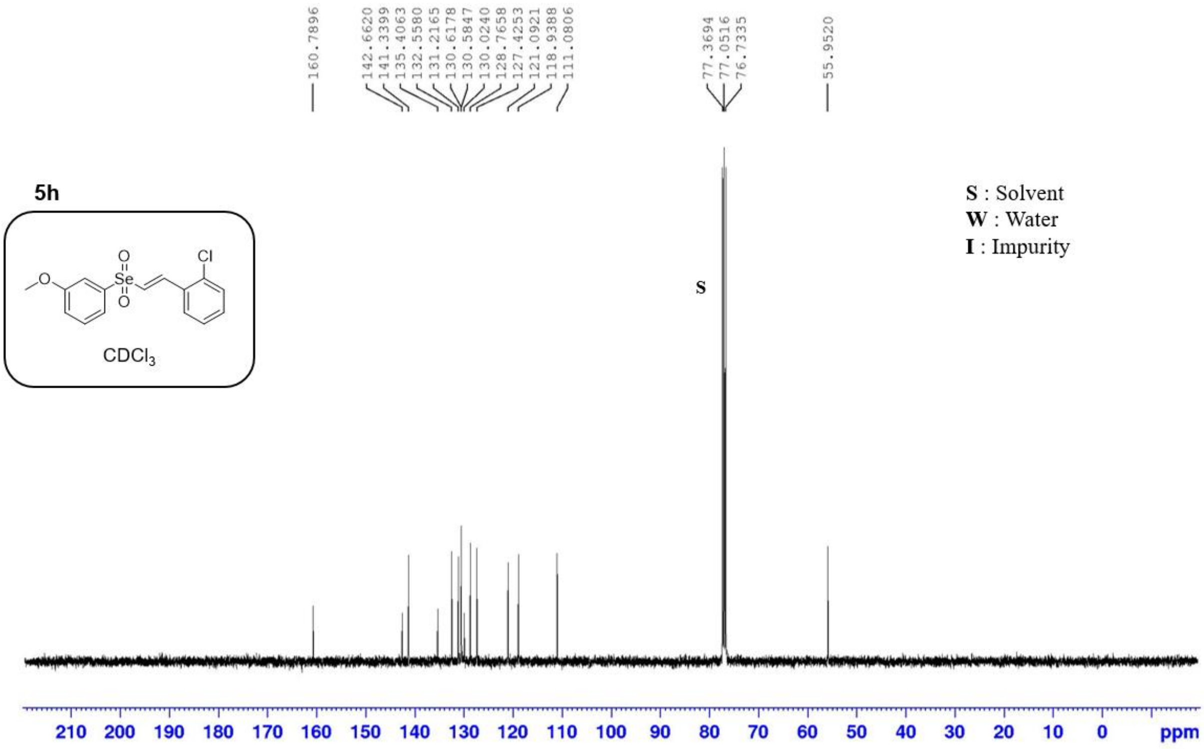
160.7896
142.6620
141.3399
135.4063
132.5580
131.2165
130.6178
130.5847
130.0240
128.7658
127.4253
121.0921
118.9388
111.0806
77.3694
77.0516
76.7335
55.9520
5h
CDCl3
S : Solvent
W : Water
I : Impurity
S
210 200 190 180 170 160 150 140 130 120 110 100 90 80 70 60 50 40 30 20 10 0 ppm

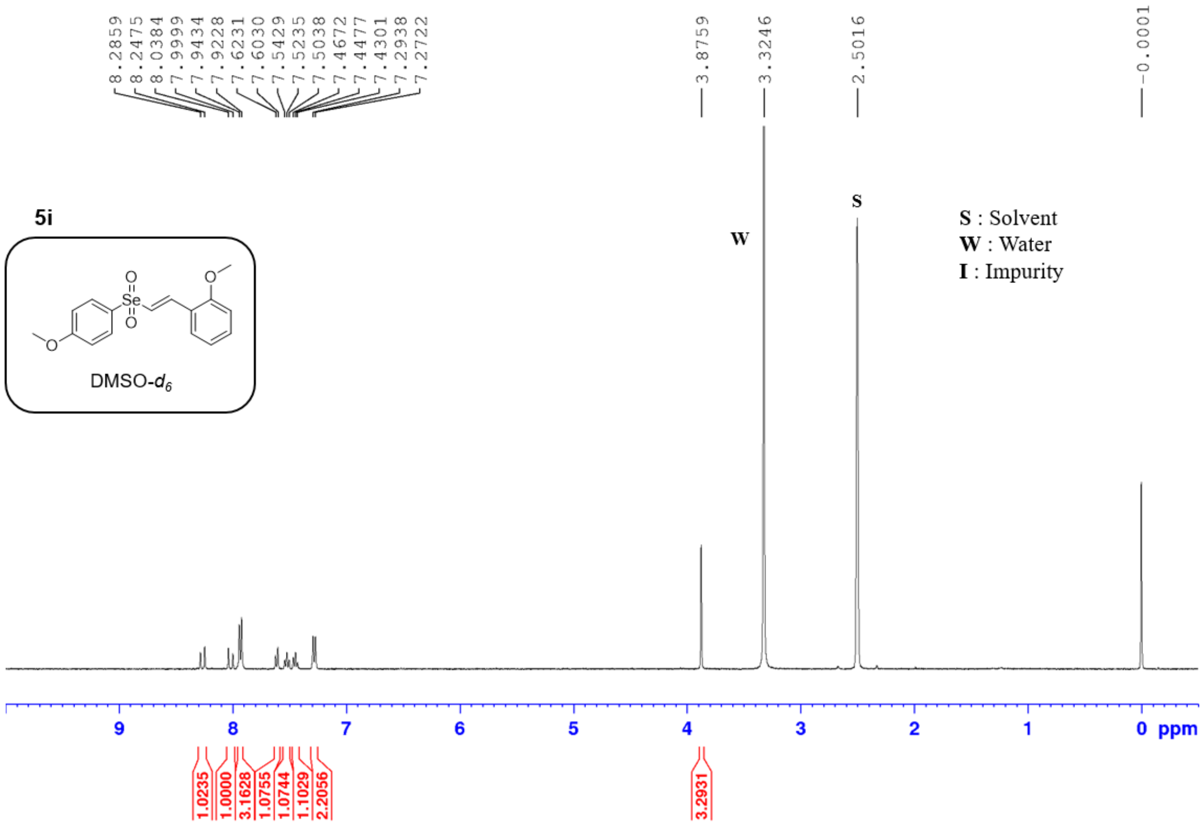
8.2859
8.2475
8.0384
7.9999
7.9434
7.9228
7.6231
7.6030
7.5429
7.5235
7.5038
7.4672
7.4477
7.4301
7.2938
7.2722
3.8759
3.3246
2.5016
-0.0001
5i
DMSO-d6
W
S
S : Solvent
W : Water
I : Impurity
ppm
1.0235
1.0000
3.1628
1.0755
1.0744
1.1029
2.2056
3.2931

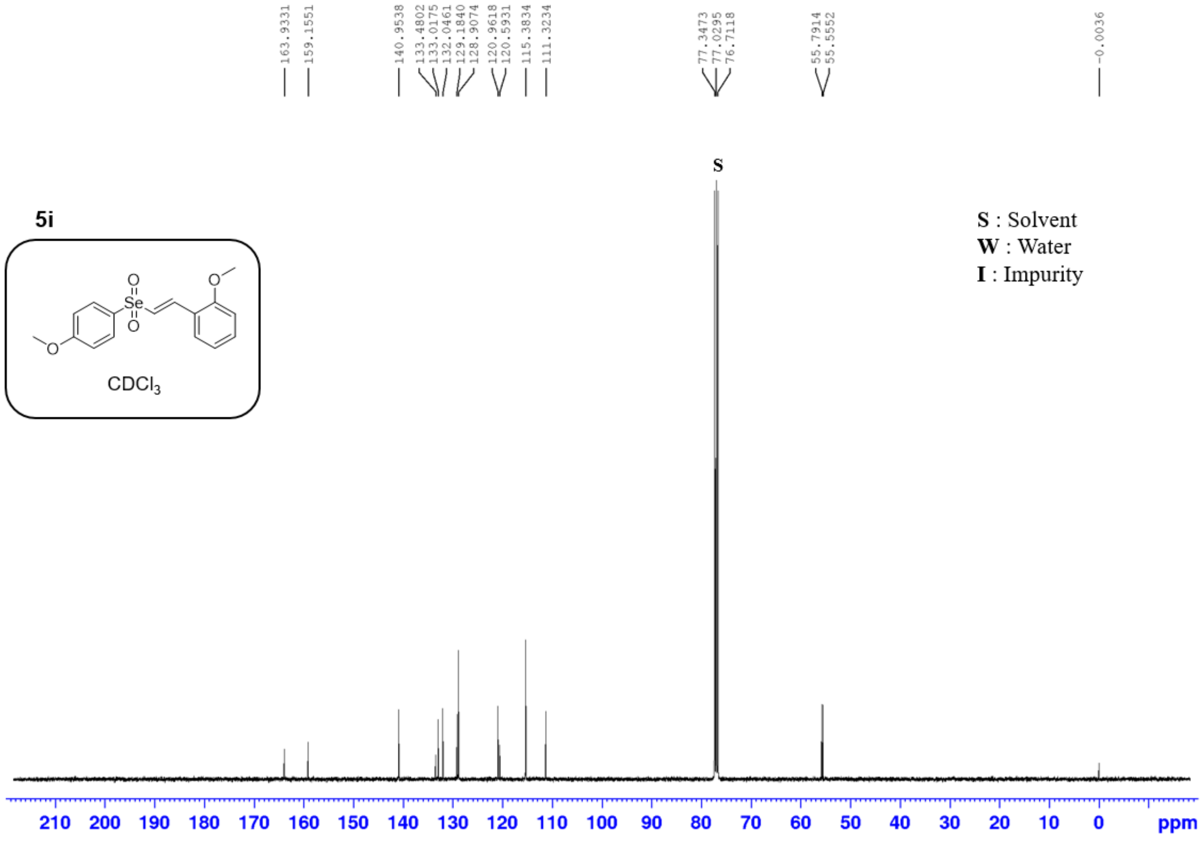
163.9331
159.1551
140.9538
133.4802
133.0175
132.0461
129.1840
128.9074
120.9618
120.5931
115.3834
111.3234
77.3473
77.0295
76.7118
55.7914
55.5552
-0.0036
5i
CDCl3
S
S : Solvent
W : Water
I : Impurity
ppm

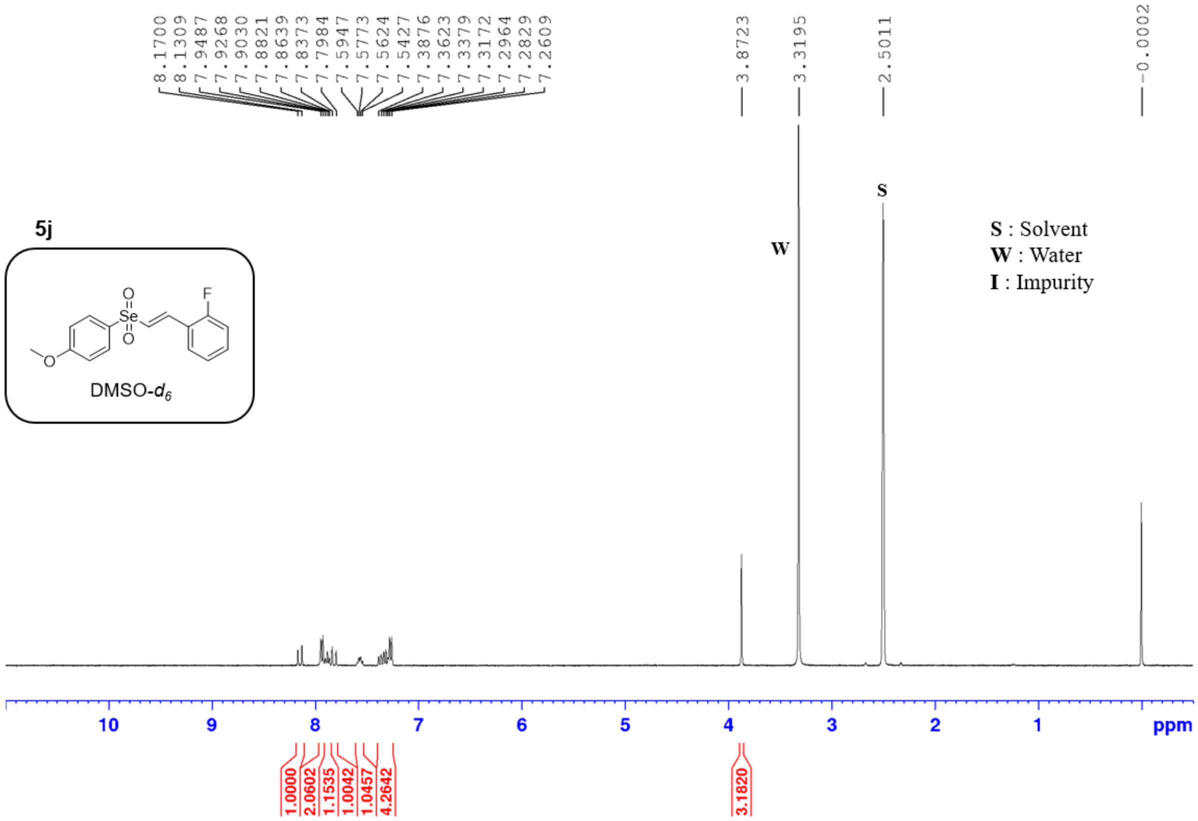
8.1700
8.1309
7.9487
7.9268
7.9030
7.8821
7.8639
7.8373
7.7984
7.5947
7.5773
7.5624
7.5427
7.3876
7.3623
7.3379
7.3172
7.2964
7.2829
7.2609
3.8723
3.3195
2.5011
-0.0002
5j
DMSO-d6
W
S
S : Solvent
W : Water
I : Impurity
10
9
8
7
6
5
4
3
2
1
ppm
1.0000
2.0602
1.1535
1.0042
1.0457
4.2642
3.1820

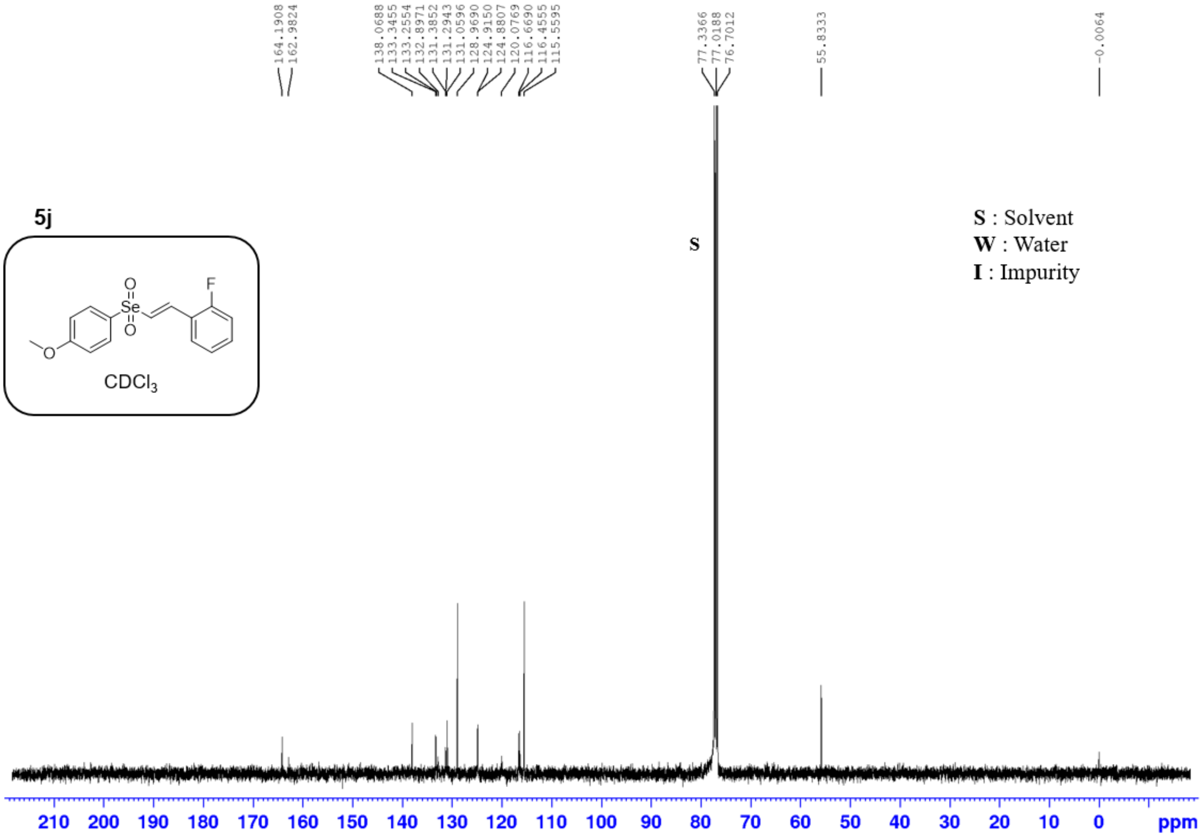
164.1908
162.9824
138.0688
133.3455
133.2554
132.8971
131.3852
131.2943
131.0596
128.9690
124.9150
124.8807
120.0769
116.6690
116.4555
115.5595
77.3366
77.0188
76.7012
55.8333
-0.0064
5j
CDCl3
S
S : Solvent
W : Water
I : Impurity
210
200
190
180
170
160
150
140
130
120
110
100
90
80
70
60
50
40
30
20
10
0
ppm

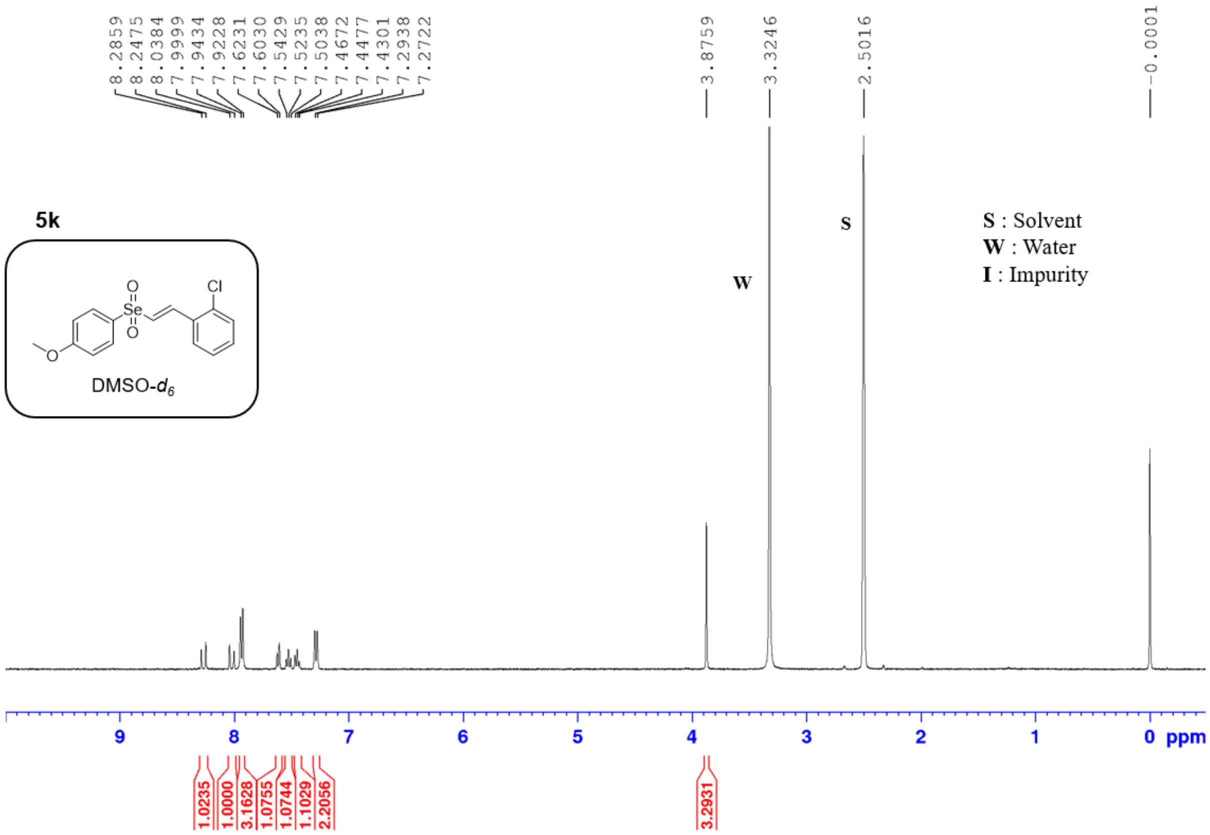
8.2859
8.2475
8.0384
7.9999
7.9434
7.9228
7.6231
7.6030
7.5429
7.5235
7.5038
7.4672
7.4477
7.4301
7.2938
7.2722
3.8759
3.3246
2.5016
-0.0001
5k
DMSO-d_6
W
S
S : Solvent
W : Water
I : Impurity
9
8
7
6
5
4
3
2
1
0 ppm
1.0235
1.0000
3.1628
1.0755
1.0744
1.1029
2.2056
3.2931

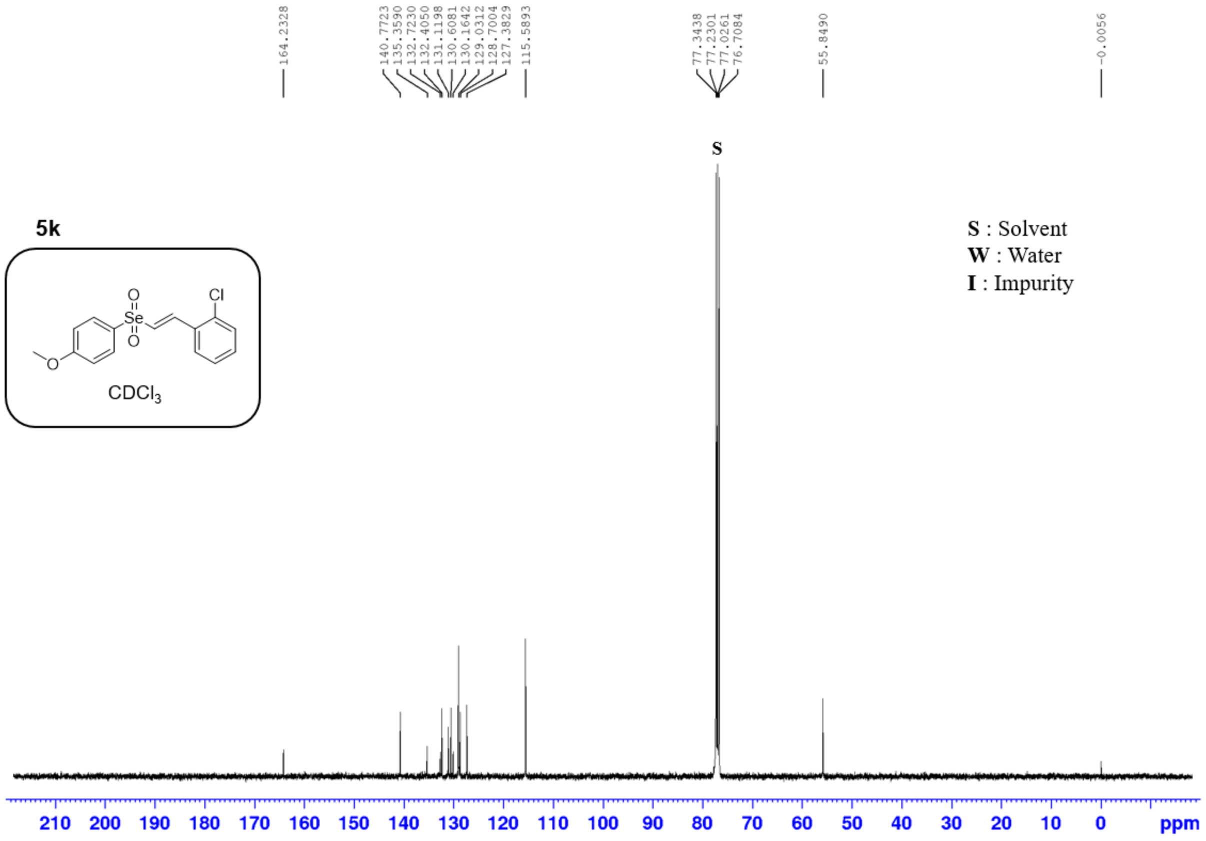
164.2328
140.7723
135.3590
132.7230
132.4050
131.1198
130.6081
130.1642
129.0312
128.7004
127.3829
115.5893
77.3438
77.2301
77.0261
76.7084
55.8490
-0.0056
S
5k
CDCl$_3$
S : Solvent
W : Water
I : Impurity
210
200
190
180
170
160
150
140
130
120
110
100
90
80
70
60
50
40
30
20
10
0
ppm

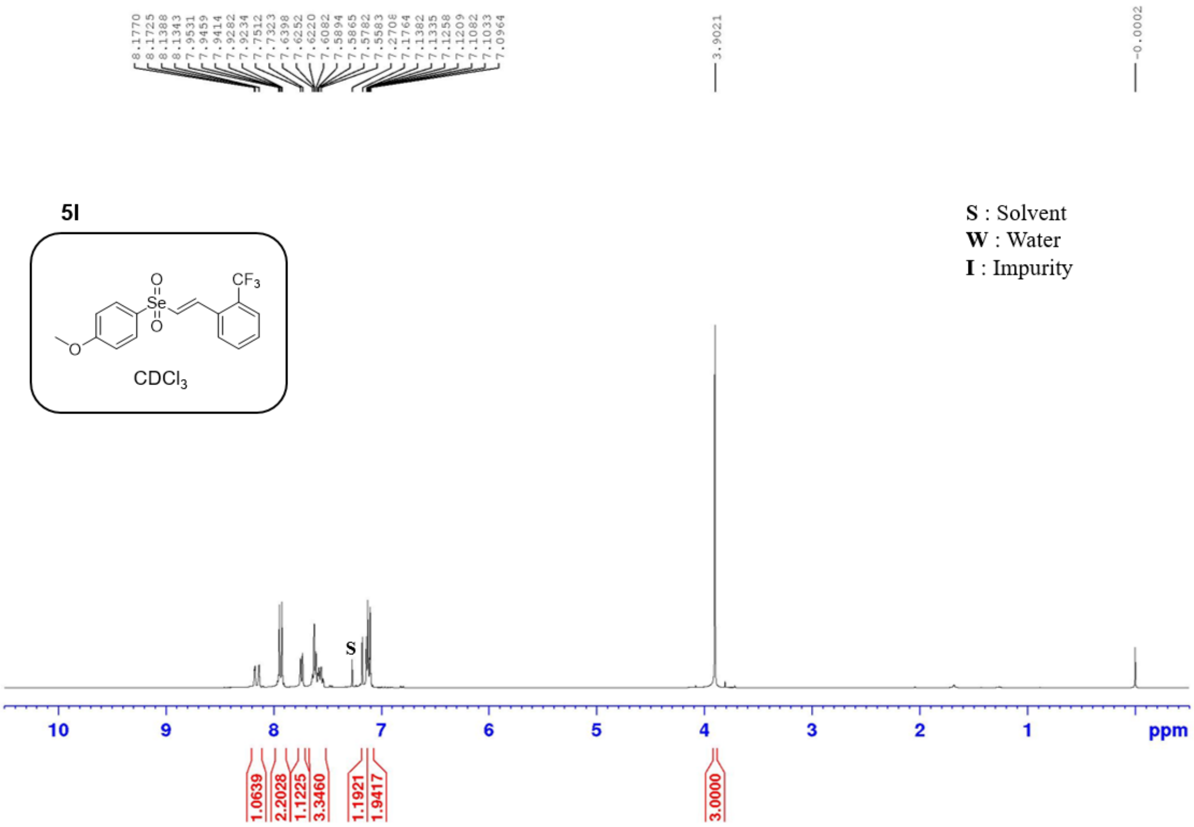
8.1770
8.1725
8.1388
8.1343
7.9531
7.9459
7.9414
7.9282
7.9234
7.7512
7.7323
7.6398
7.6252
7.6220
7.6082
7.5894
7.5865
7.5782
7.5583
7.2708
7.1764
7.1382
7.1335
7.1258
7.1209
7.1082
7.1033
7.0964
3.9021
-0.0002
5l
CF3
Se
O
CDCl3
S : Solvent
W : Water
I : Impurity
S
10 9 8 7 6 5 4 3 2 1 ppm
1.0639
2.2028
1.1225
3.3460
1.1921
1.9417
3.0000

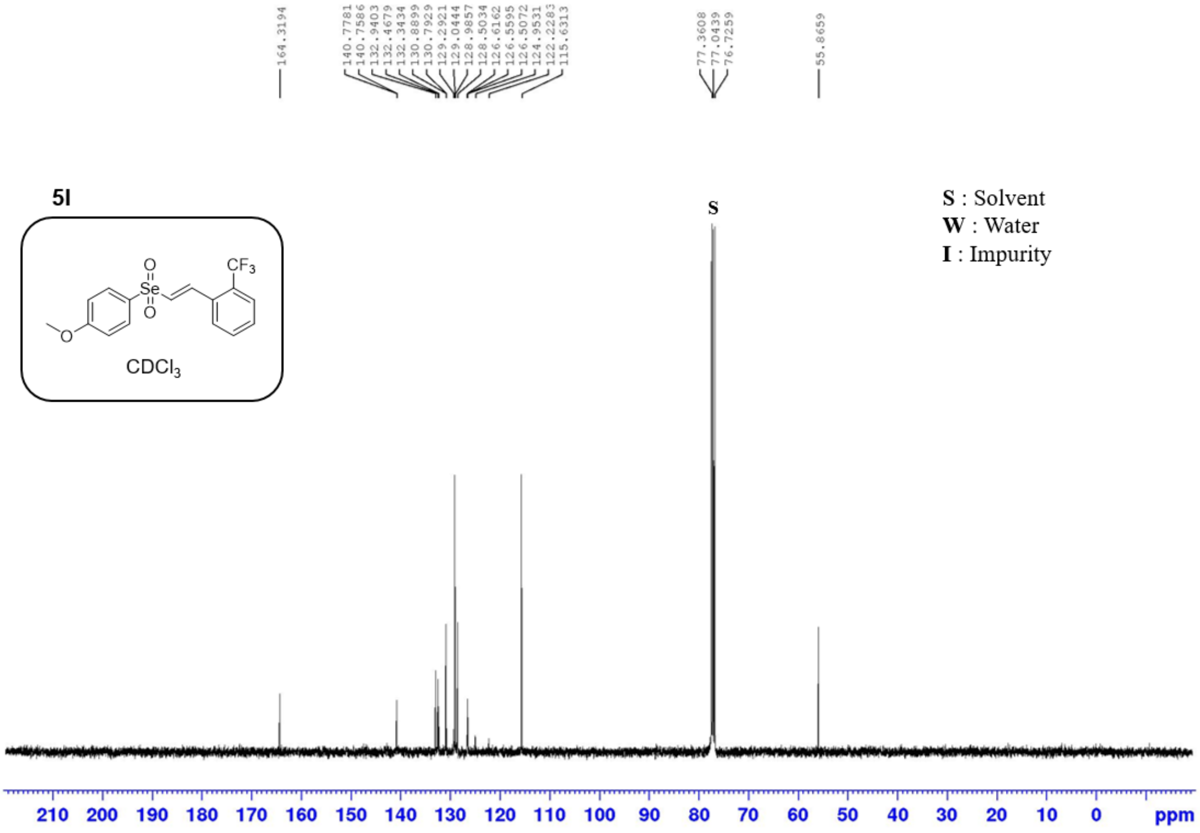
164.3194
140.7781
140.7586
132.9403
132.4679
132.3434
130.8899
130.7929
129.2921
129.0444
128.9857
128.5034
126.6162
126.5595
126.5072
124.9531
122.2283
115.6313
77.3608
77.0439
76.7259
55.8659
5l
CF3
Se
O
CDCl3
S : Solvent
W : Water
I : Impurity
S
210 200 190 180 170 160 150 140 130 120 110 100 90 80 70 60 50 40 30 20 10 0 ppm

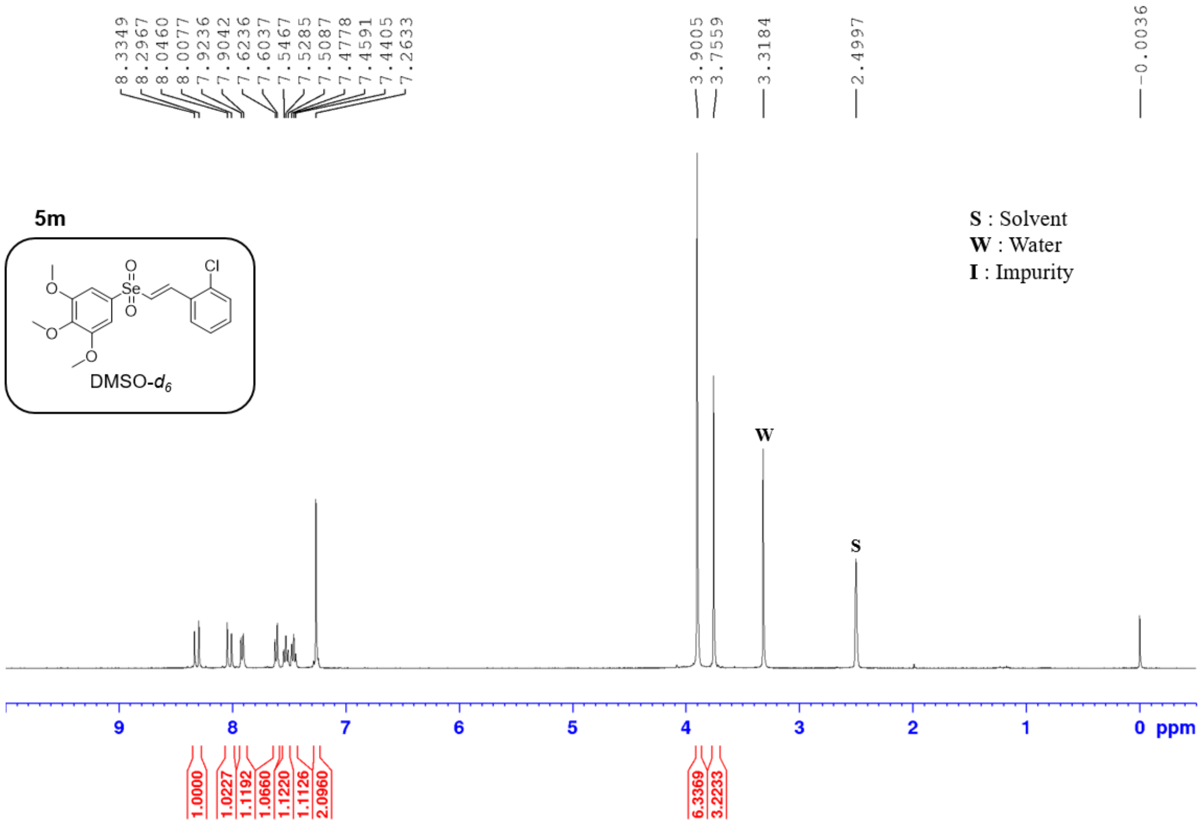
8.3349
8.2967
8.0460
8.0077
7.9236
7.9042
7.6236
7.6037
7.5467
7.5285
7.5087
7.4778
7.4591
7.4405
7.2633
3.9005
3.7559
3.3184
2.4997
-0.0036
5m
Cl
Se
O
DMSO-d_6
S : Solvent
W : Water
I : Impurity
W
S
9
8
7
6
5
4
3
2
1
0 ppm
1.0000
1.0227
1.1192
1.0660
1.1220
1.1126
2.0960
6.3369
3.2233

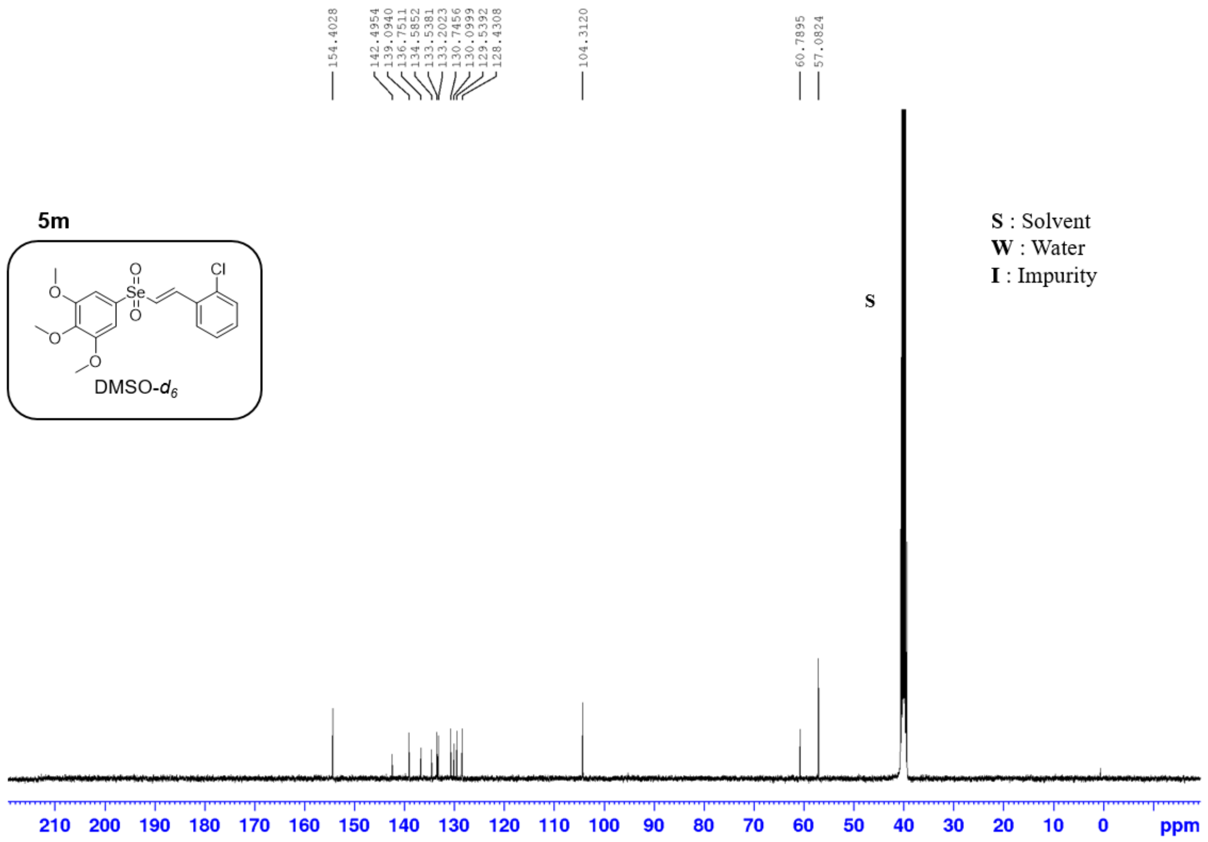
154.4028
142.4954
139.0940
136.7511
134.5852
133.5381
133.2023
130.7456
130.0999
129.5392
128.4308
104.3120
60.7895
57.0824
5m
Cl
Se
O
DMSO-d_6
S : Solvent
W : Water
I : Impurity
S
210 200 190 180 170 160 150 140 130 120 110 100 90 80 70 60 50 40 30 20 10 0 ppm

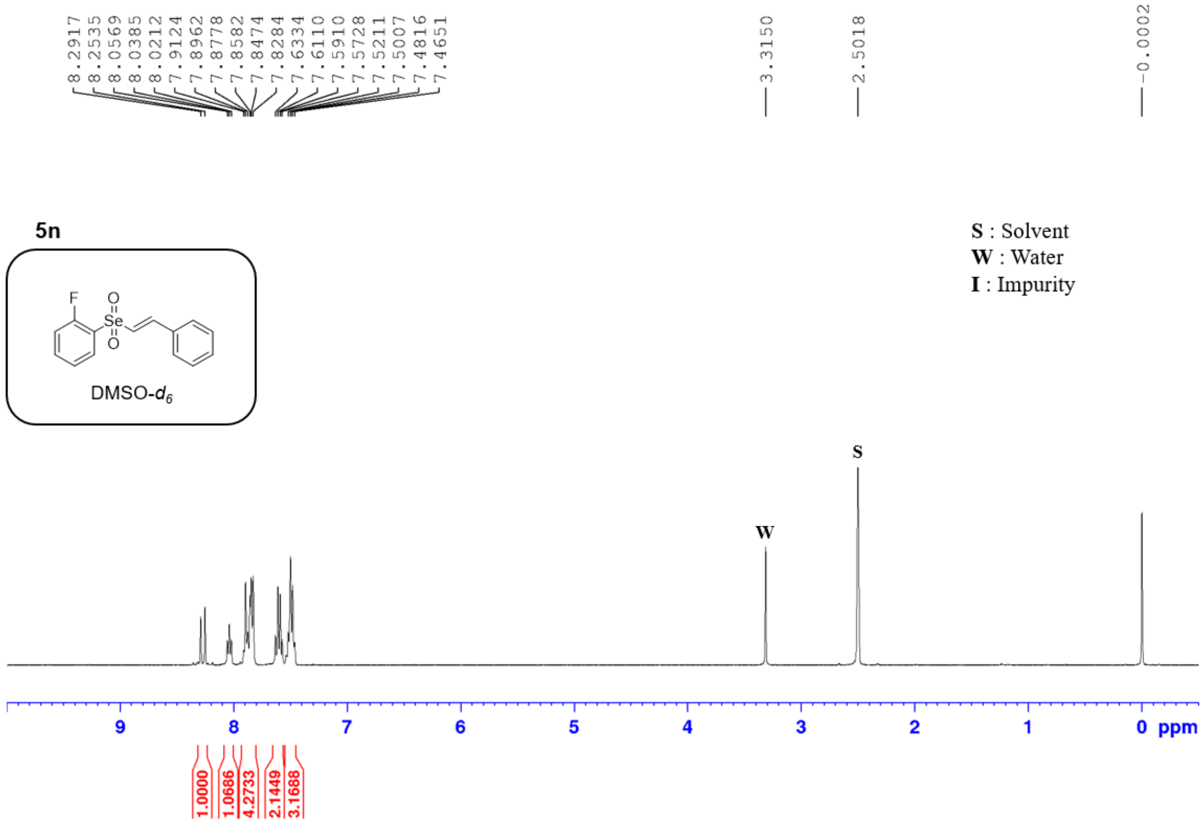
8.2917
8.2535
8.0569
8.0385
8.0212
7.9124
7.8962
7.8778
7.8582
7.8474
7.8284
7.6334
7.6110
7.5910
7.5728
7.5211
7.5007
7.4816
7.4651
3.3150
2.5018
-0.0002
5n
F
Se
O
O
DMSO-d_6
S : Solvent
W : Water
I : Impurity
W
S
9
8
7
6
5
4
3
2
1
0 ppm
1.0000
1.0686
4.2733
2.1449
3.1688

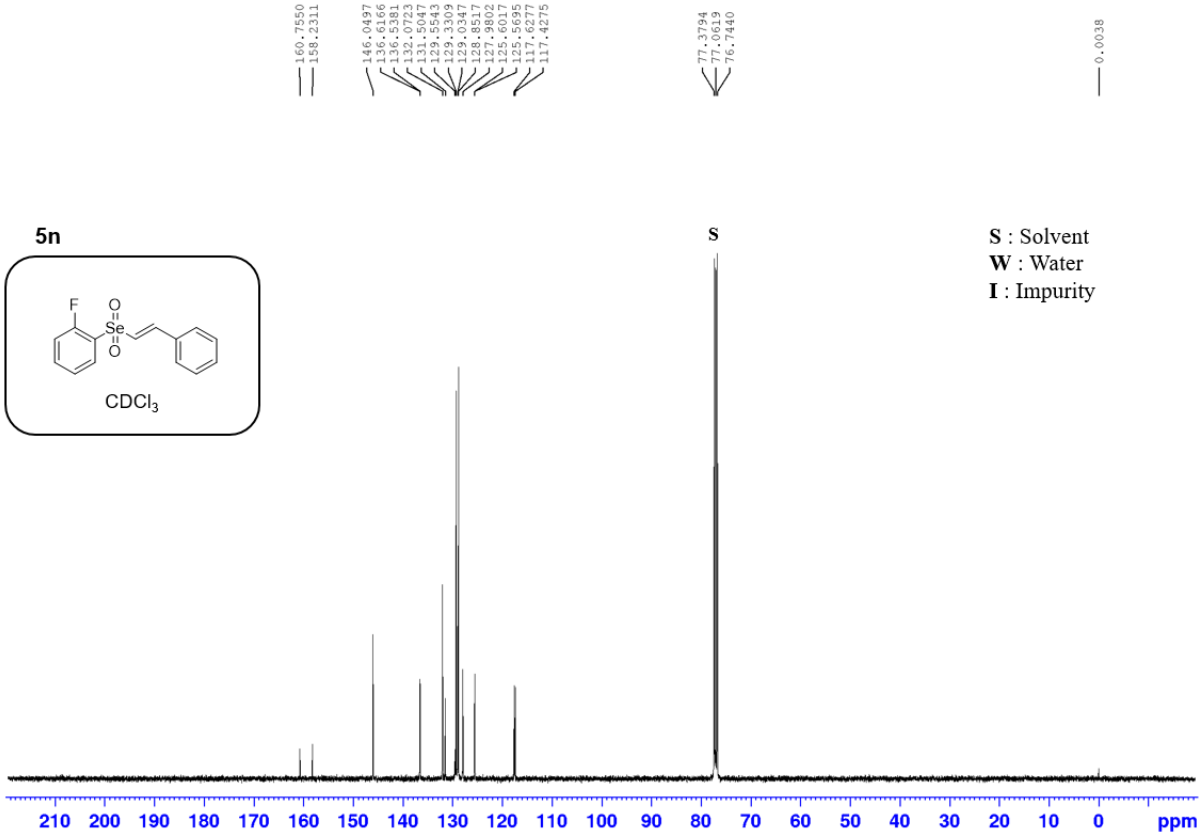
160.7550
158.2311
146.0497
136.6166
136.5381
132.0723
131.5047
129.5543
129.3309
129.0347
128.8517
127.9802
125.6017
125.5695
117.6277
117.4275
77.3794
77.0619
76.7440
0.0038
5n
F
Se
O
O
$CDCl_3$
S : Solvent
W : Water
I : Impurity
S
210
200
190
180
170
160
150
140
130
120
110
100
90
80
70
60
50
40
30
20
10
0
ppm

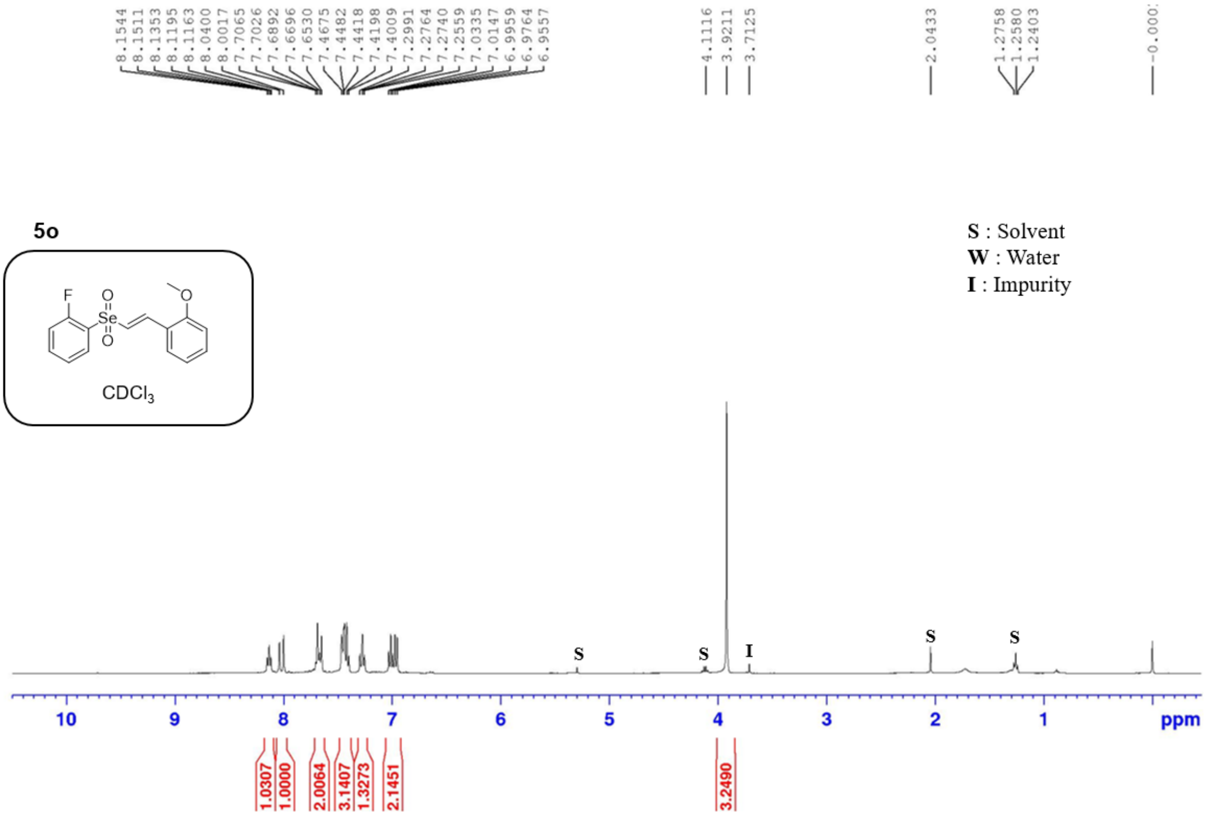
8.1544
8.1511
8.1353
8.1195
8.1163
8.0400
8.0017
7.7065
7.7026
7.6892
7.6696
7.6530
7.4675
7.4482
7.4418
7.4198
7.4009
7.2991
7.2764
7.2740
7.2559
7.0335
7.0147
6.9959
6.9764
6.9557
4.1116
3.9211
3.7125
2.0433
1.2758
1.2580
1.2403
5o
CDCl3
S : Solvent
W : Water
I : Impurity
1.0307
1.0000
2.0064
3.1407
1.3273
2.1451
3.2490
ppm

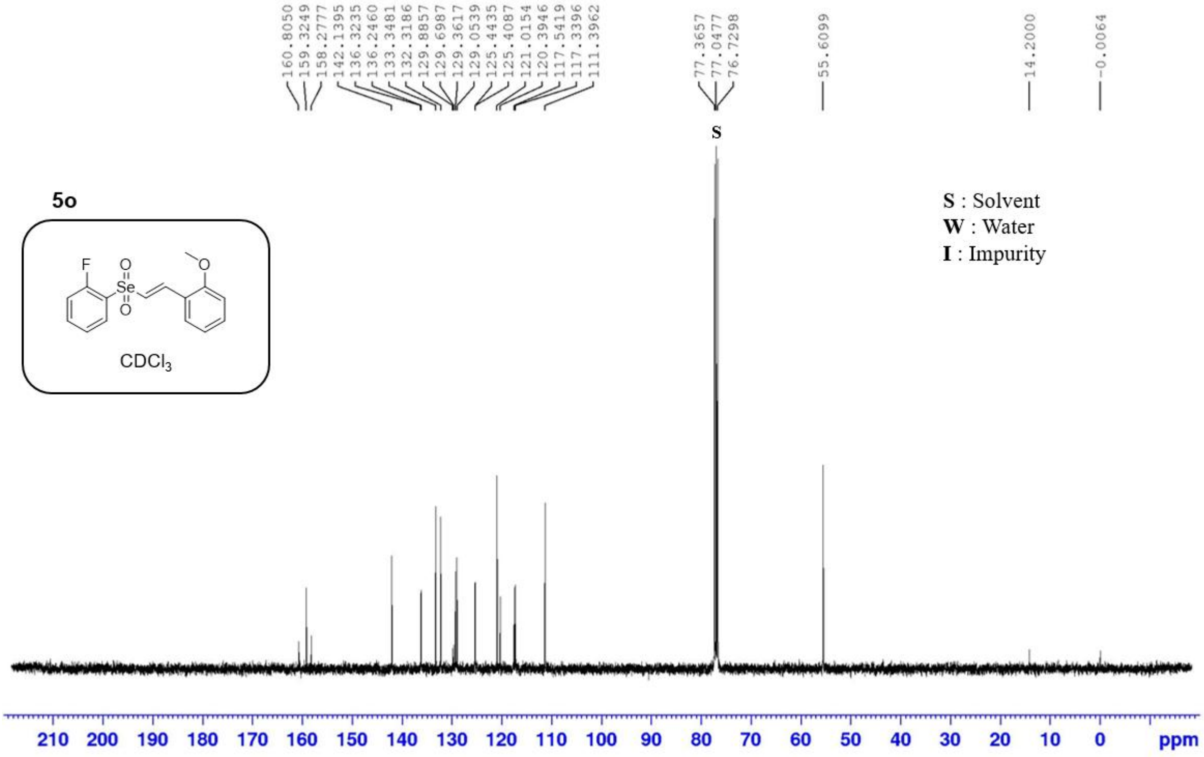
160.8050
159.3249
158.2777
142.1395
136.3235
136.2460
133.3481
132.3186
129.8857
129.6987
129.3617
129.0539
125.4435
125.4087
121.0154
120.3946
117.5419
117.3396
111.3962
77.3657
77.0477
76.7298
55.6099
14.2000
-0.0064
5o
CDCl3
S : Solvent
W : Water
I : Impurity
ppm

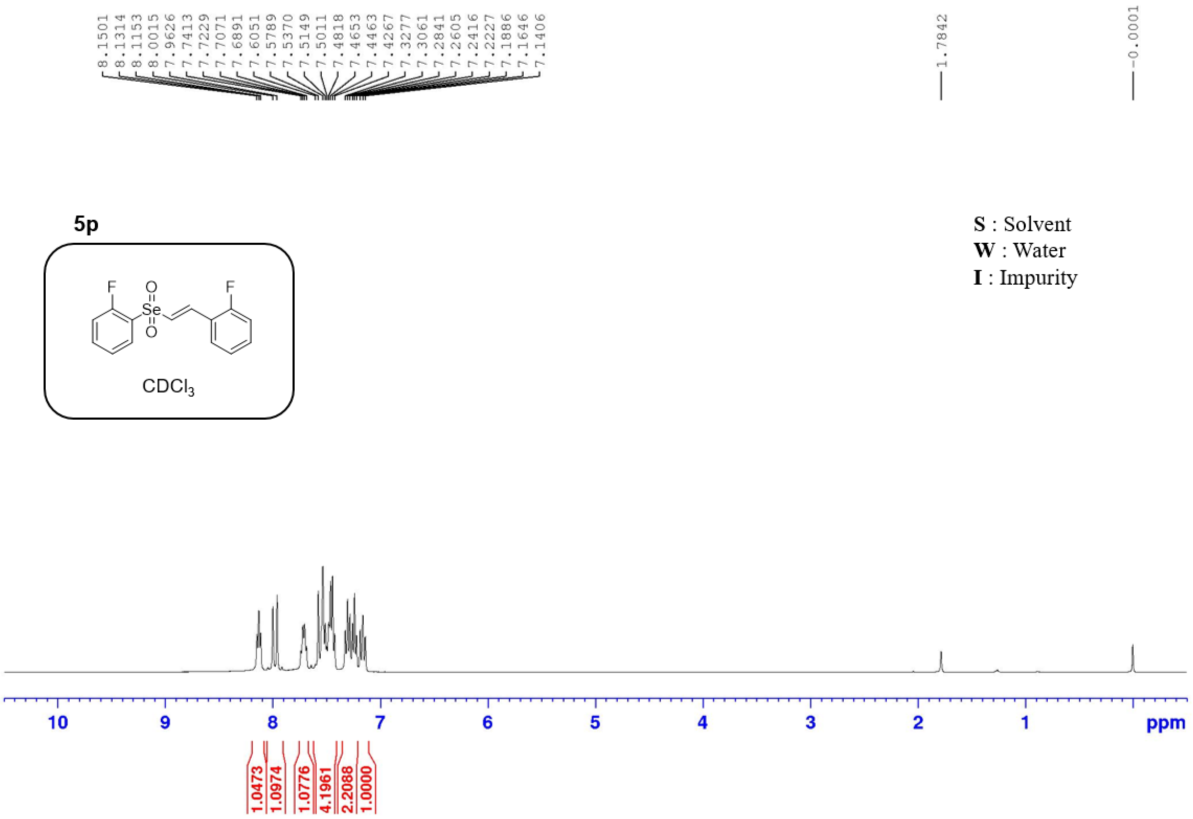
8.1501
8.1314
8.1153
8.0015
7.9626
7.7413
7.7229
7.7071
7.6891
7.6051
7.5789
7.5370
7.5149
7.5011
7.4818
7.4653
7.4463
7.4267
7.3277
7.3061
7.2841
7.2605
7.2416
7.2227
7.1886
7.1646
7.1406
1.7842
-0.0001
5p
CDCl3
S : Solvent
W : Water
I : Impurity
1.0473
1.0974
1.0776
4.1961
2.2088
1.0000
ppm

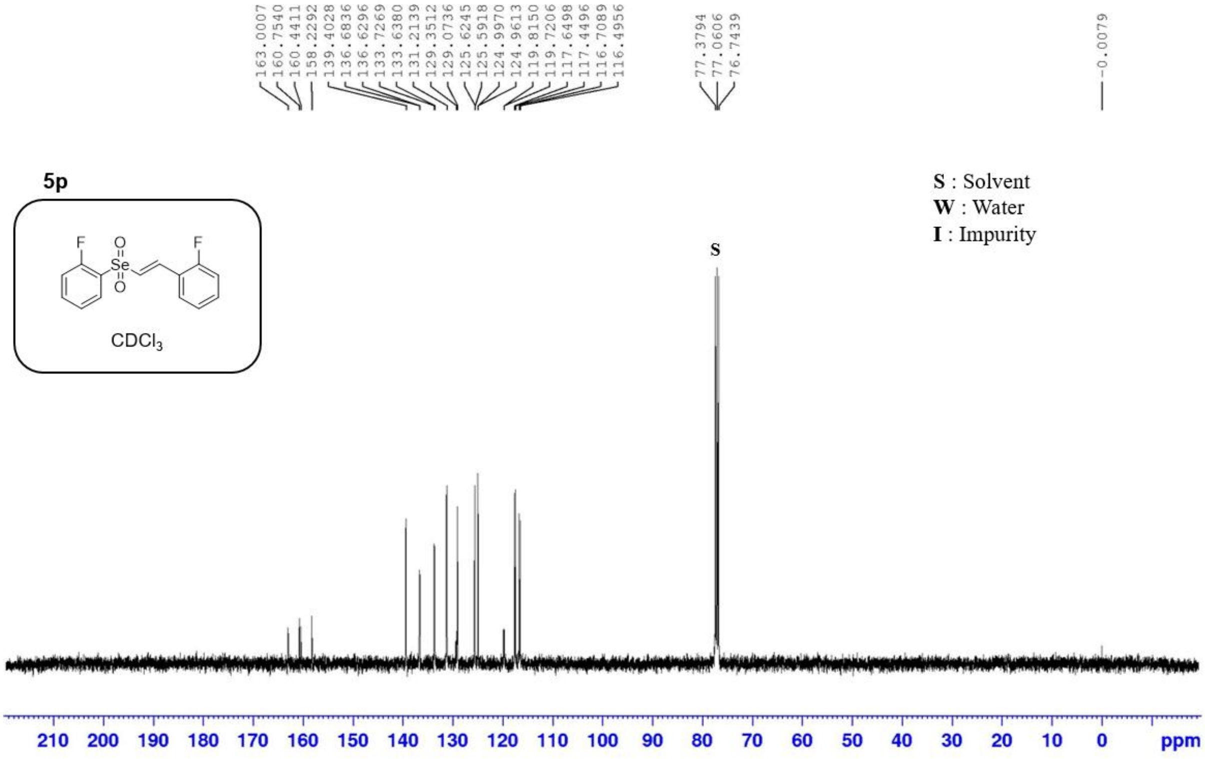
163.0007
160.7540
160.4411
158.2292
139.4028
136.6836
136.6296
133.7269
133.6380
131.2139
129.3512
129.0736
125.6245
125.5918
124.9970
124.9613
119.8150
119.7206
117.6498
117.4496
116.7089
116.4956
77.3794
77.0606
76.7439
-0.0079
5p
CDCl3
S : Solvent
W : Water
I : Impurity
S
ppm

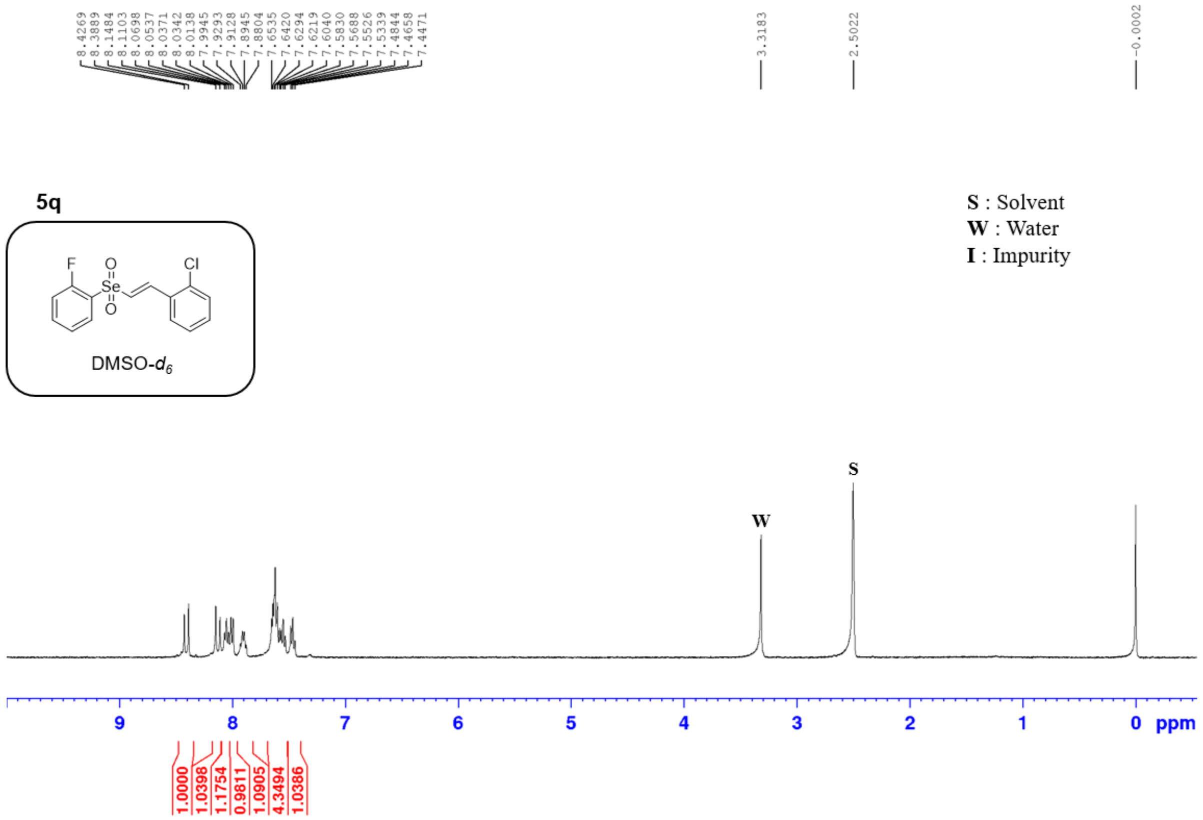
8.4269
8.3889
8.1484
8.1103
8.0698
8.0537
8.0371
8.0342
8.0138
7.9945
7.9293
7.9128
7.8945
7.8804
7.6535
7.6420
7.6294
7.6219
7.6040
7.5830
7.5688
7.5526
7.5339
7.4844
7.4658
7.4471
3.3183
2.5022
-0.0002
5q
F
O
Se
O
Cl
DMSO-d_6
S : Solvent
W : Water
I : Impurity
W
S
9
8
7
6
5
4
3
2
1
0
ppm
1.0000
1.0398
1.1754
0.9811
1.0905
4.3494
1.0386

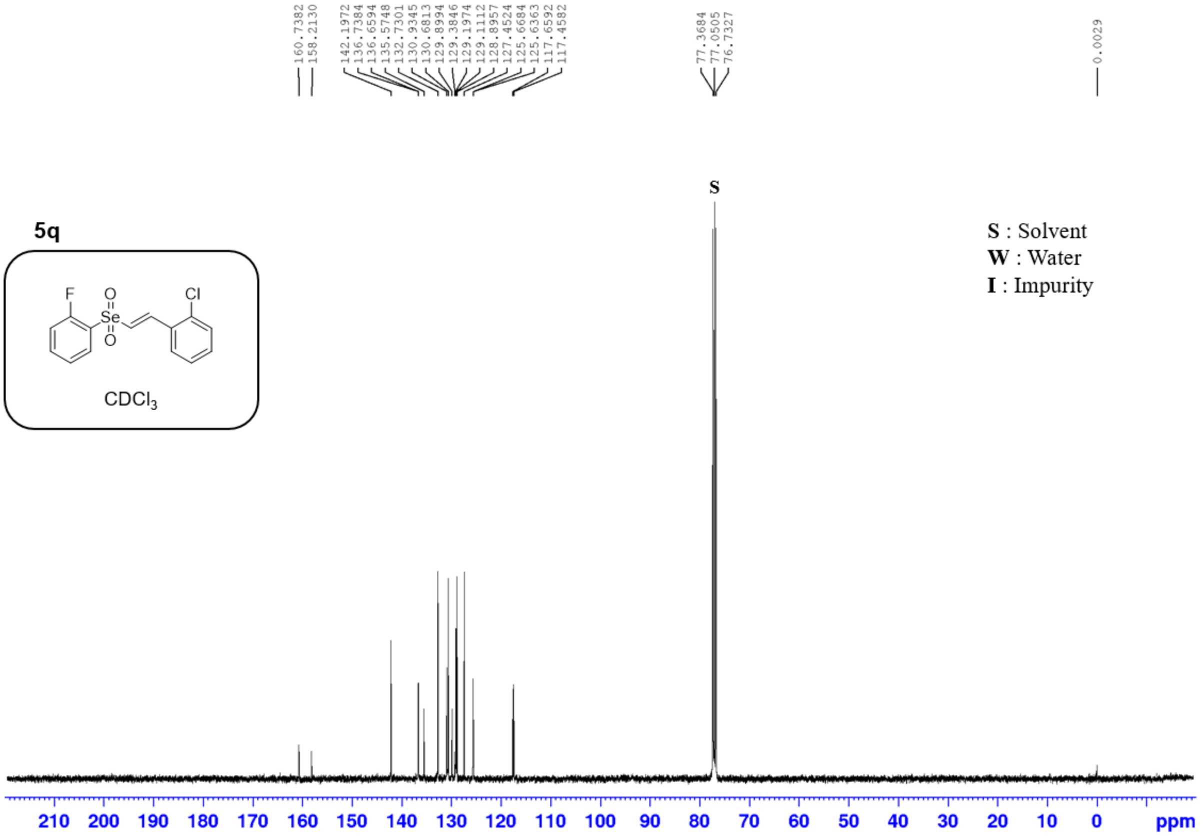
160.7382
158.2130
142.1972
136.7384
136.6594
135.5748
132.7301
130.9345
130.6813
129.8994
129.3846
129.1974
129.1112
128.8957
127.4524
125.6684
125.6363
117.6592
117.4582
77.3684
77.0505
76.7327
0.0029
5q
F
O
Se
O
Cl
CDCl$_3$
S : Solvent
W : Water
I : Impurity
S
210
200
190
180
170
160
150
140
130
120
110
100
90
80
70
60
50
40
30
20
10
0
ppm

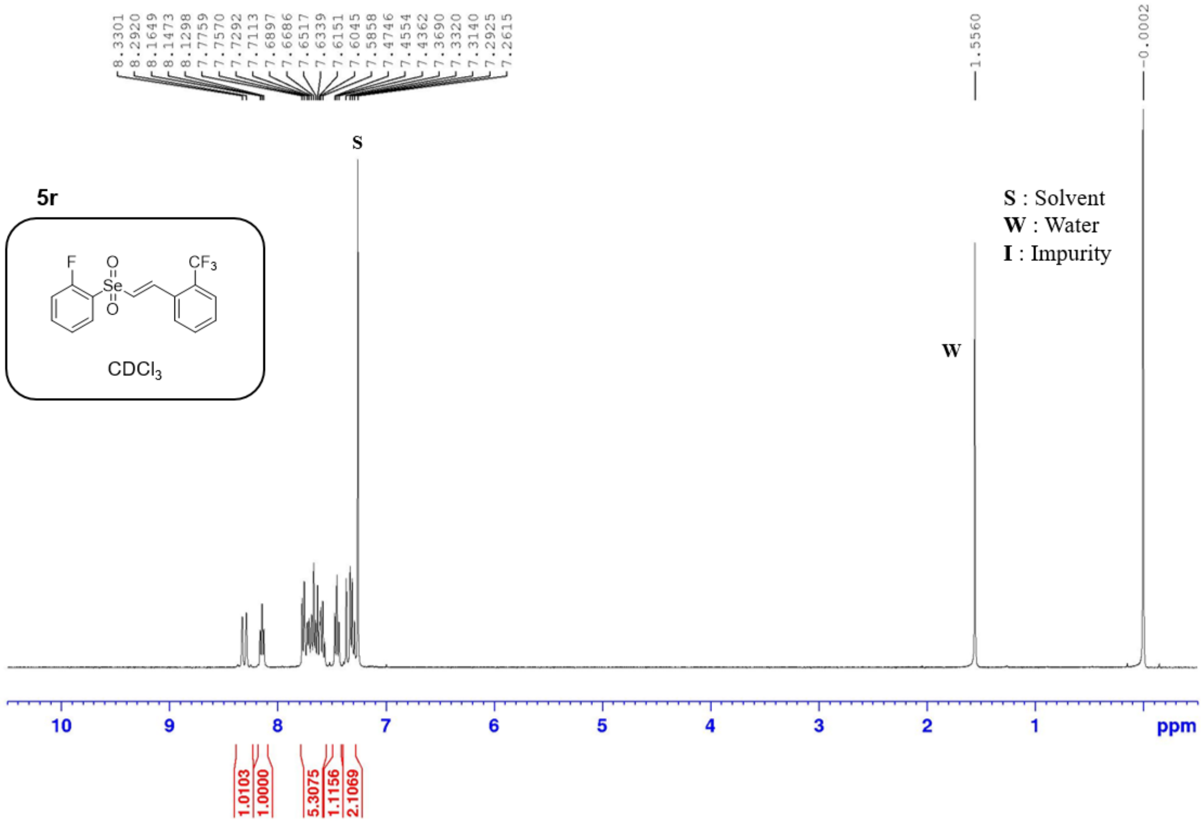
8.3301
8.2920
8.1649
8.1473
8.1298
7.7759
7.7570
7.7292
7.7113
7.6897
7.6686
7.6517
7.6339
7.6151
7.6045
7.5858
7.4746
7.4554
7.4362
7.3690
7.3320
7.3140
7.2925
7.2615
1.5560
-0.0002
S
5r
F
O
Se
O
CF3
CDCl3
S : Solvent
W : Water
I : Impurity
W
10
9
8
7
6
5
4
3
2
1
ppm
1.0103
1.0000
5.3075
1.1156
2.1069

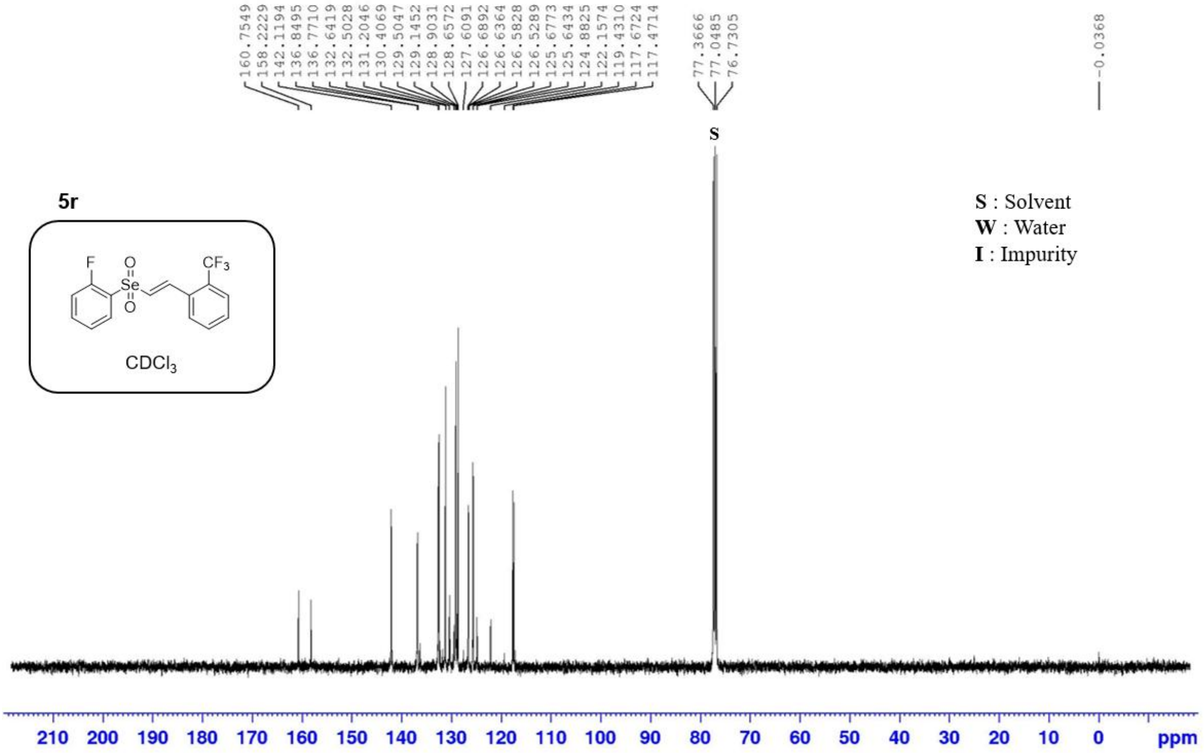
160.7549
158.2229
142.1194
136.8495
136.7710
132.6419
132.5028
131.2046
130.4069
129.5047
129.1452
128.9031
128.6572
127.6091
126.6892
126.6364
126.5828
126.5289
125.6773
125.6434
124.8825
122.1574
119.4310
117.6724
117.4714
77.3666
77.0485
76.7305
-0.0368
S
5r
F
O
Se
O
CF3
CDCl3
S : Solvent
W : Water
I : Impurity
210
200
190
180
170
160
150
140
130
120
110
100
90
80
70
60
50
40
30
20
10
0
ppm

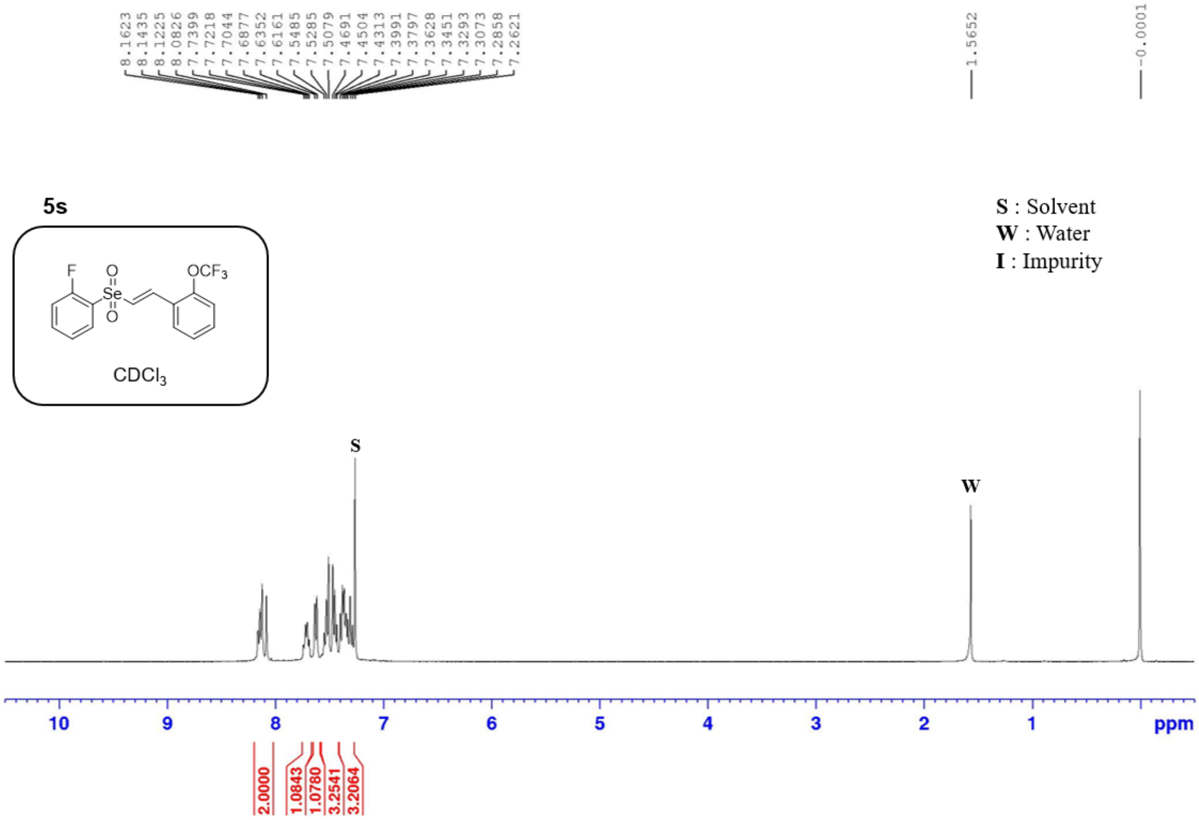
8.1623
8.1435
8.1225
8.0826
7.7399
7.7218
7.7044
7.6877
7.6352
7.6161
7.5485
7.5285
7.5079
7.4691
7.4504
7.4313
7.3991
7.3797
7.3628
7.3451
7.3293
7.3073
7.2858
7.2621
1.5652
-0.0001
5s
F
O
Se
O
OCF3
CDCl3
S : Solvent
W : Water
I : Impurity
S
W
10 9 8 7 6 5 4 3 2 1 ppm
2.0000
1.0843
1.0780
3.2541
3.2064

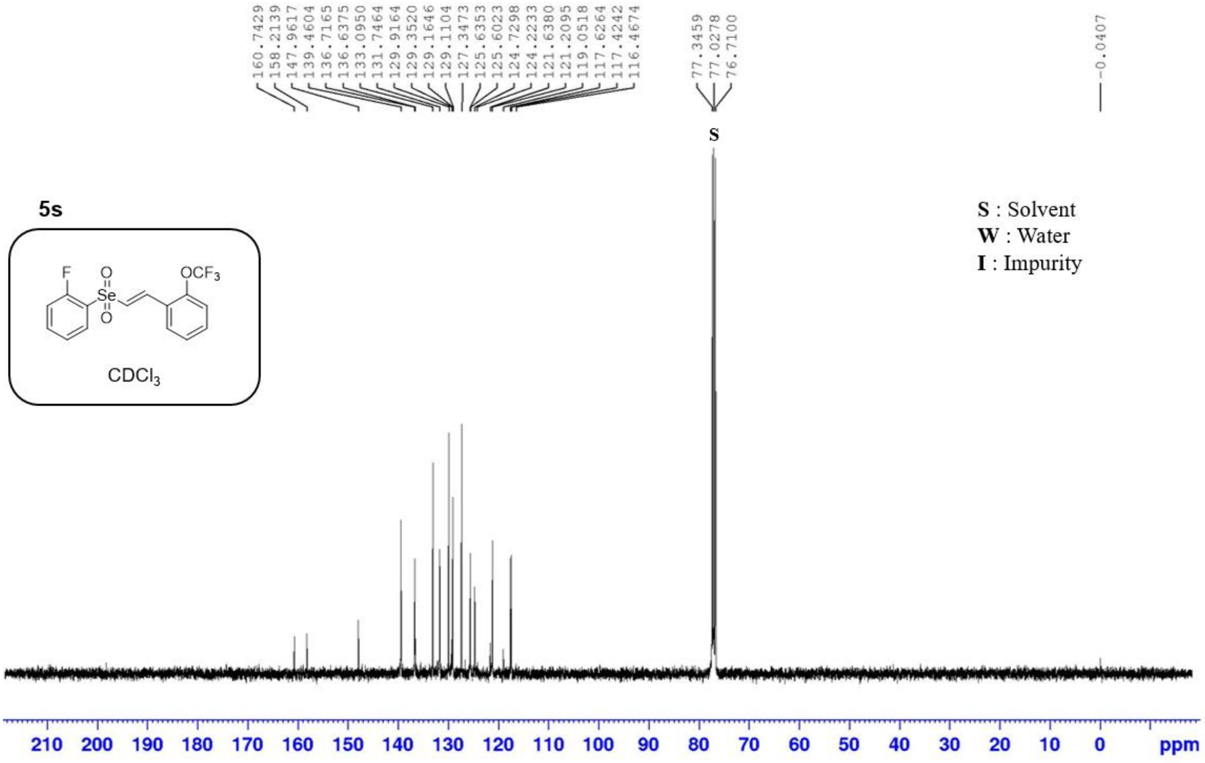
160.7429
158.2139
147.9617
139.4604
136.7165
136.6375
133.0950
131.7464
129.9164
129.3520
129.1646
129.1104
127.3473
125.6353
125.6023
124.7298
124.2233
121.6380
121.2095
119.0518
117.6264
117.4242
116.4674
77.3459
77.0278
76.7100
-0.0407
S
5s
F
O
Se
O
OCF3
CDCl3
S : Solvent
W : Water
I : Impurity
210 200 190 180 170 160 150 140 130 120 110 100 90 80 70 60 50 40 30 20 10 0 ppm

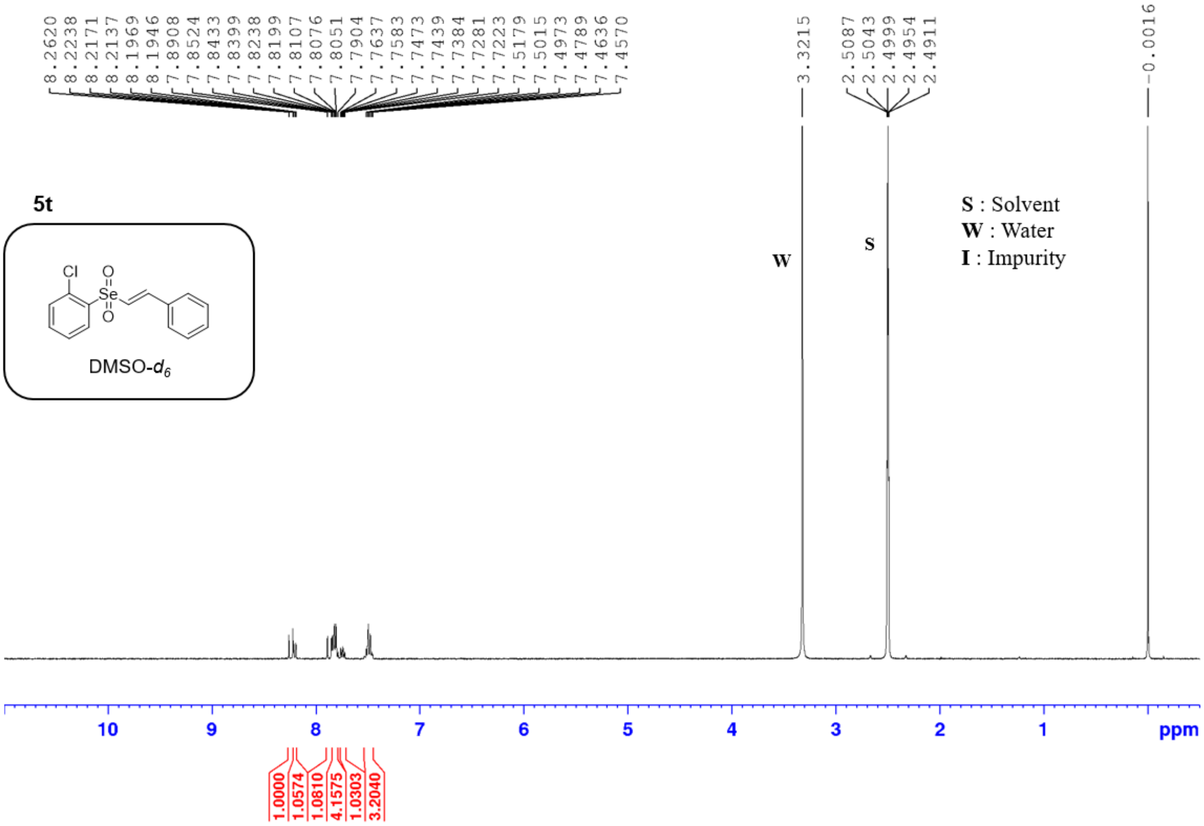
8.2620
8.2238
8.2171
8.2137
8.1969
8.1946
7.8908
7.8524
7.8433
7.8399
7.8238
7.8199
7.8107
7.8076
7.8051
7.7904
7.7637
7.7583
7.7473
7.7439
7.7384
7.7281
7.7223
7.5179
7.5015
7.4973
7.4789
7.4636
7.4570
3.3215
2.5087
2.5043
2.4999
2.4954
2.4911
-0.0016
5t
Cl
O
Se
O
DMSO-d_6
S : Solvent
W : Water
I : Impurity
W
S
10
9
8
7
6
5
4
3
2
1
ppm
1.0000
1.0574
1.0810
4.1575
1.0303
3.2040

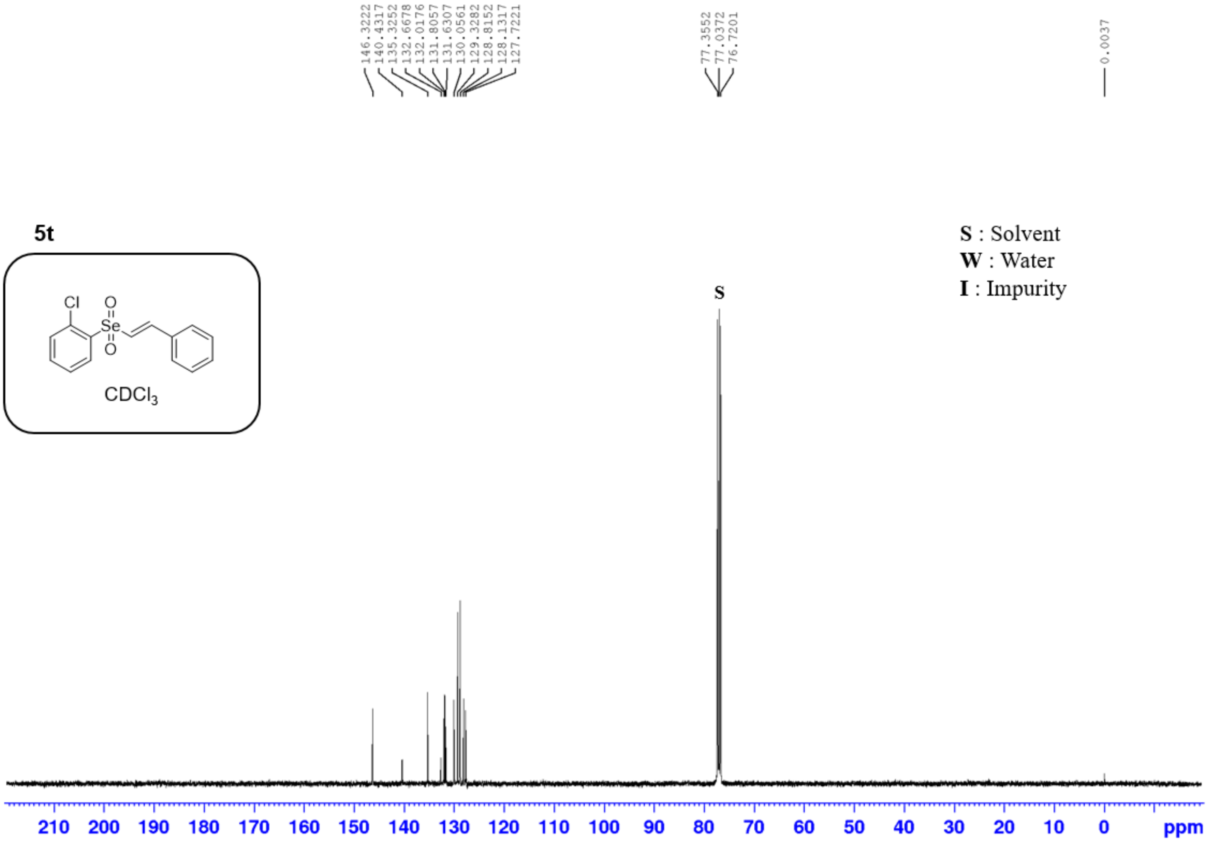
146.3222
140.4317
135.3252
132.6678
132.0176
131.8057
131.6307
130.0561
129.3282
128.8152
128.1317
127.7221
77.3552
77.0372
76.7201
0.0037
5t
Cl
O
Se
O
$CDCl_3$
S : Solvent
W : Water
I : Impurity
S
210
200
190
180
170
160
150
140
130
120
110
100
90
80
70
60
50
40
30
20
10
0
ppm

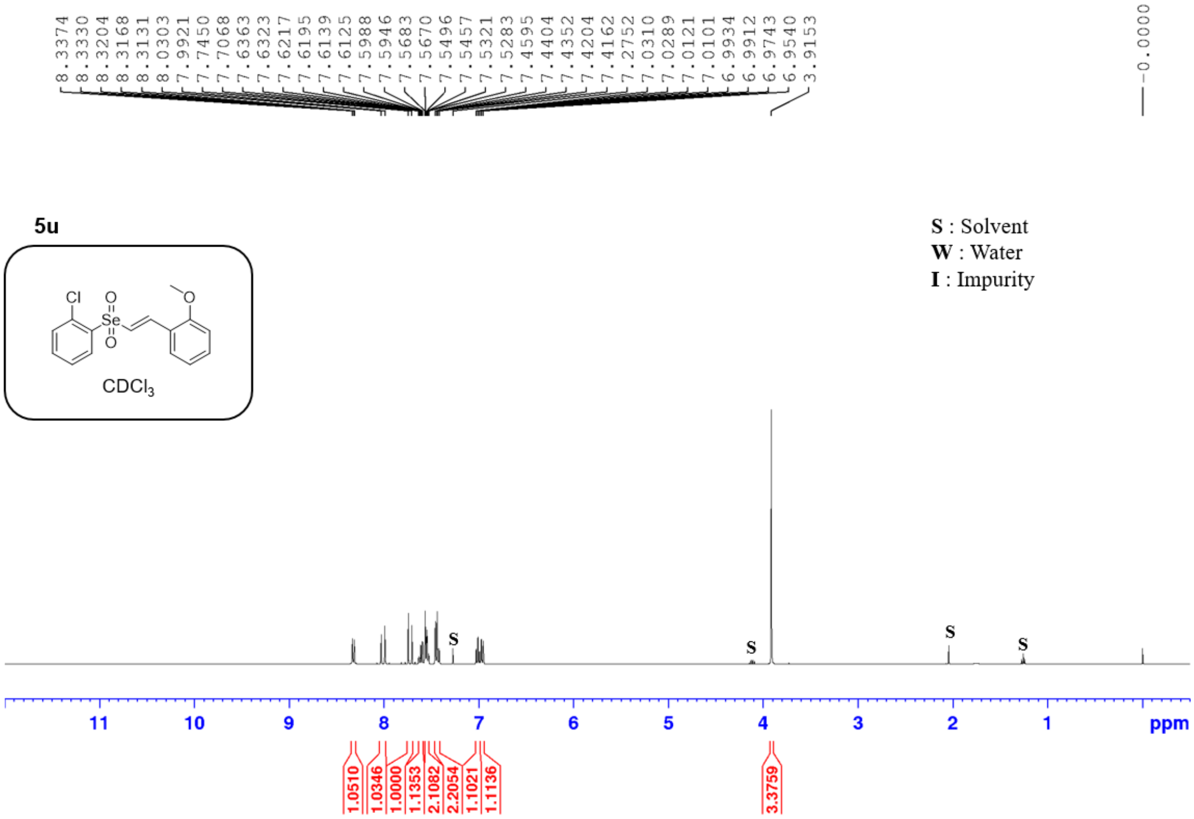
8.3374
8.3330
8.3204
8.3168
8.3131
8.0303
7.9921
7.7450
7.7068
7.6363
7.6323
7.6217
7.6195
7.6139
7.6125
7.5988
7.5946
7.5683
7.5670
7.5496
7.5457
7.5321
7.5283
7.4595
7.4404
7.4352
7.4204
7.4162
7.2752
7.0310
7.0289
7.0121
7.0101
6.9934
6.9912
6.9743
6.9540
3.9153
0.0000
5u
Cl
O
Se
O
O
CDCl3
S : Solvent
W : Water
I : Impurity
S
S
S
S
11
10
9
8
7
6
5
4
3
2
1
ppm
1.0510
1.0346
1.0000
1.1353
2.1082
2.2054
1.1021
1.1136
3.3759

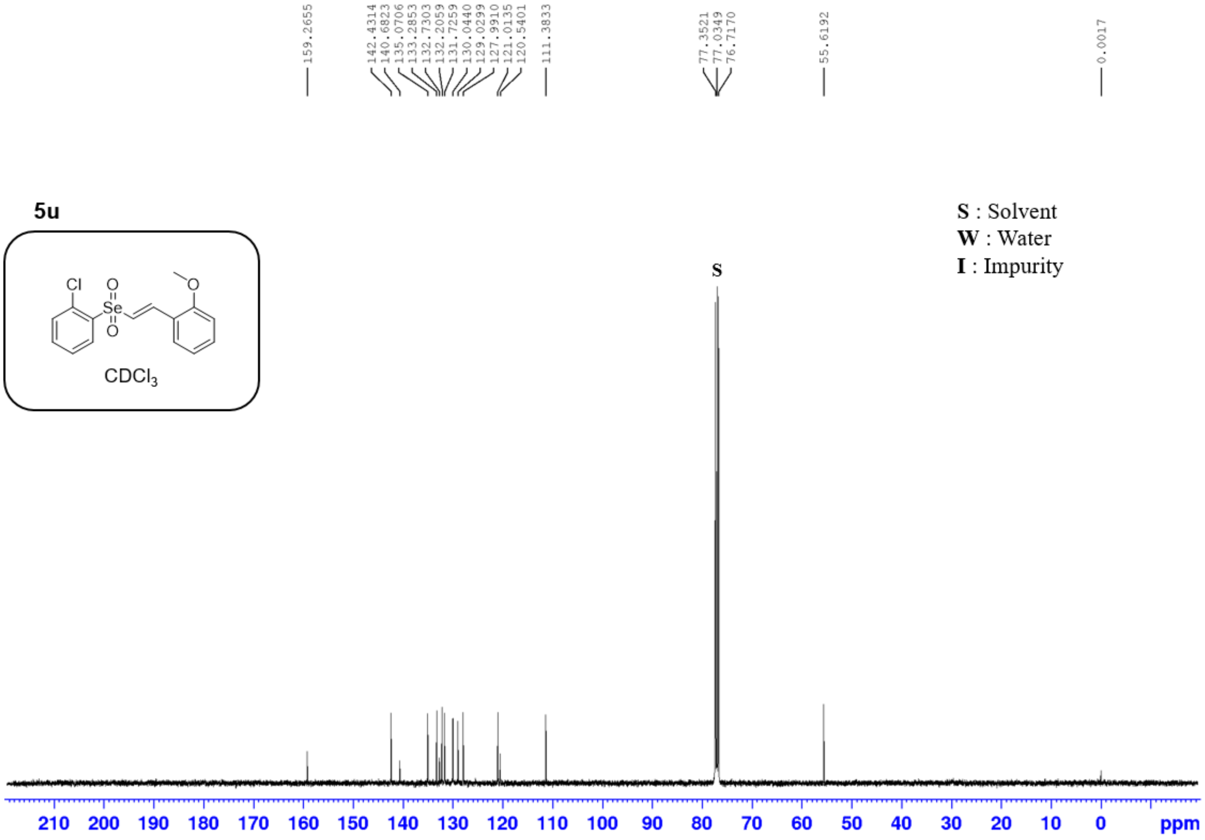
159.2655
142.4314
140.6823
135.0706
133.2853
132.7303
132.2059
131.7259
130.0440
129.0299
127.9910
121.0135
120.5401
111.3833
77.3521
77.0349
76.7170
55.6192
0.0017
5u
Cl
O
Se
O
O
CDCl3
S : Solvent
W : Water
I : Impurity
S
210
200
190
180
170
160
150
140
130
120
110
100
90
80
70
60
50
40
30
20
10
0
ppm

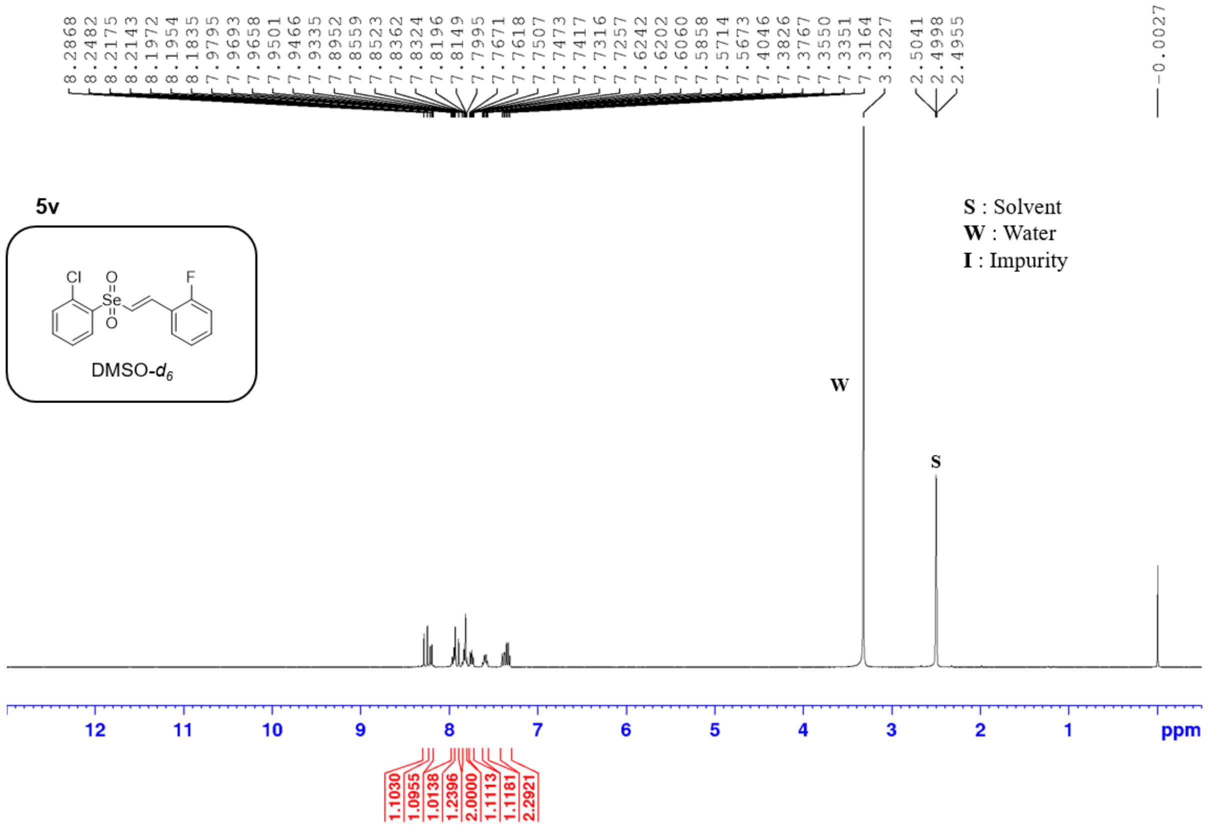
5v
DMSO-d_6
S : Solvent
W : Water
I : Impurity
W
S
ppm

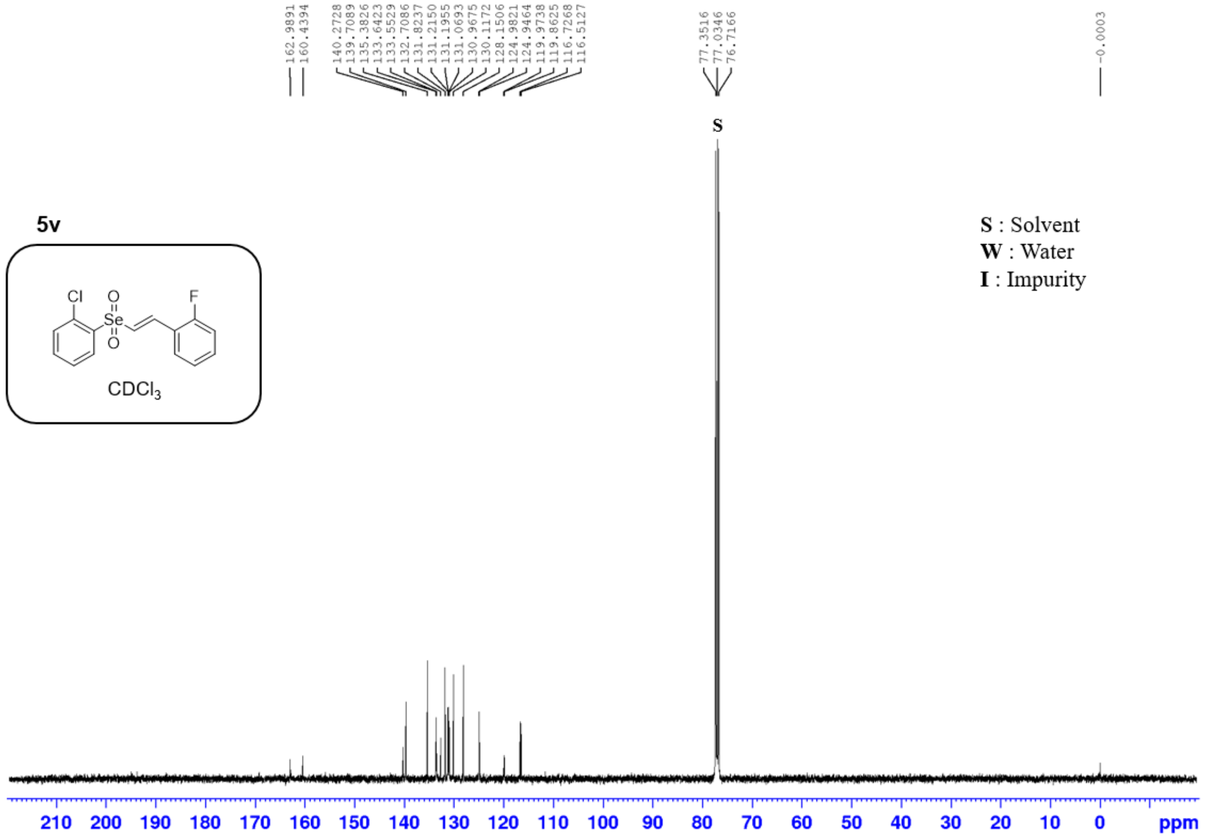
5v
CDCl$_3$
S : Solvent
W : Water
I : Impurity
S
ppm

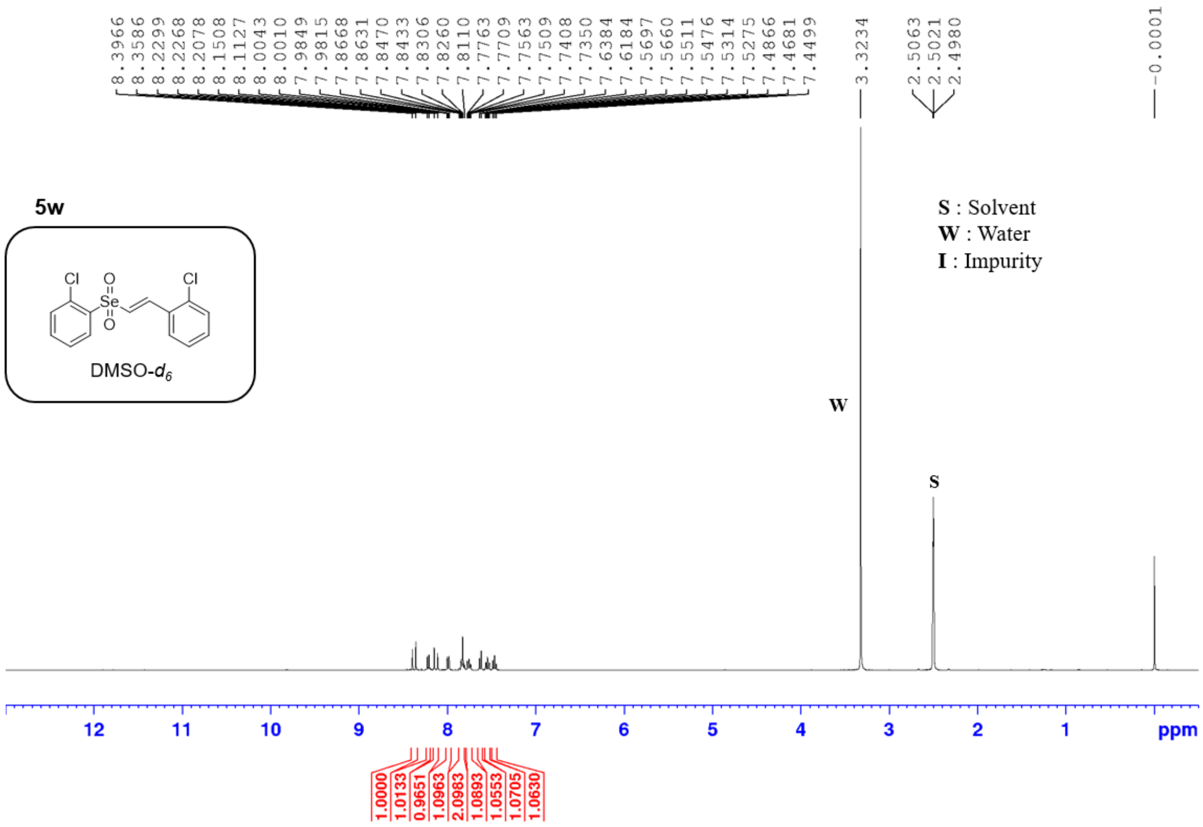
8.3966
8.3586
8.2299
8.2268
8.2078
8.1508
8.1127
8.0043
8.0010
7.9849
7.9815
7.8668
7.8631
7.8470
7.8433
7.8306
7.8260
7.8110
7.7763
7.7709
7.7563
7.7509
7.7408
7.7350
7.6384
7.6184
7.5697
7.5660
7.5511
7.5476
7.5314
7.5275
7.4866
7.4681
7.4499
3.3234
2.5063
2.5021
2.4980
-0.0001
5w
DMSO-d_6
S : Solvent
W : Water
I : Impurity
W
S
12
11
10
9
8
7
6
5
4
3
2
1
ppm
1.0000
1.0133
0.9651
1.0963
2.0983
1.0893
1.0553
1.0705
1.0630

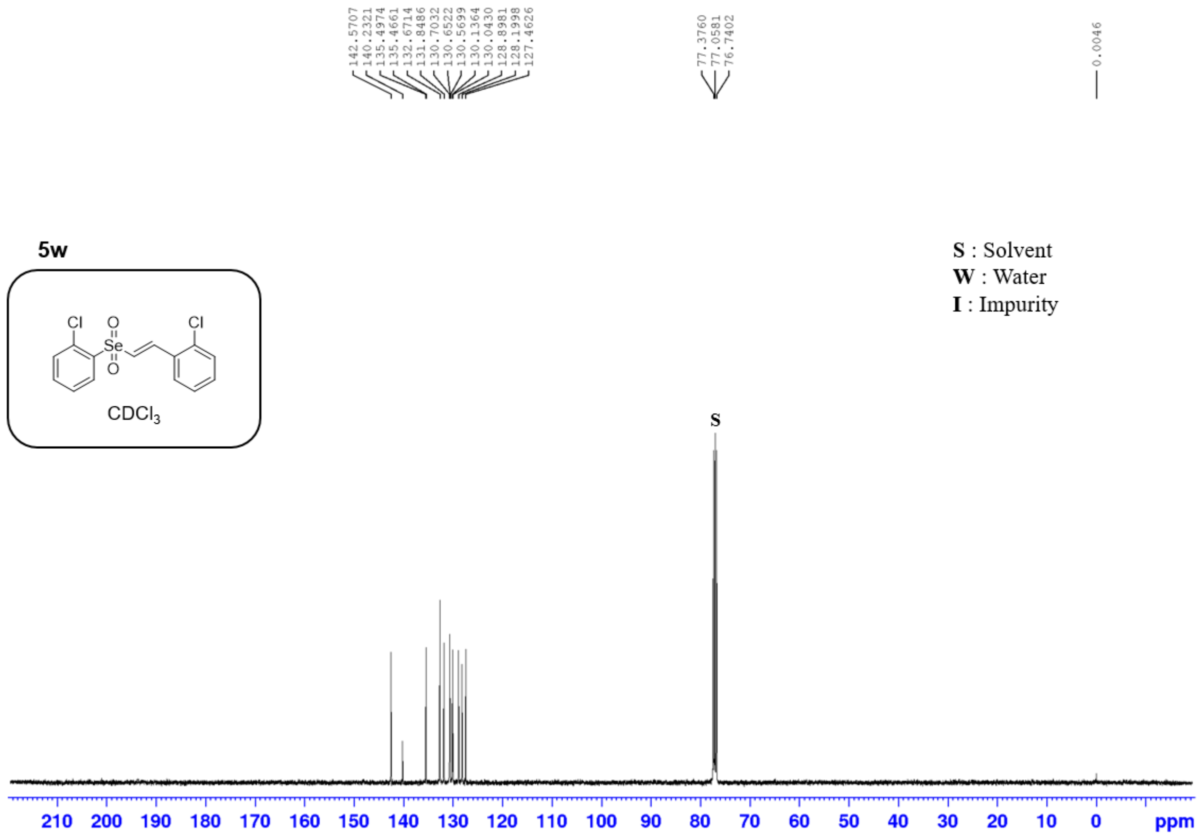
142.5707
140.2321
135.4974
135.4661
132.6714
131.8486
130.7032
130.6522
130.5699
130.1364
130.0430
128.8981
128.1998
127.4626
77.3760
77.0581
76.7402
0.0046
5w
CDCl$_3$
S : Solvent
W : Water
I : Impurity
S
210
200
190
180
170
160
150
140
130
120
110
100
90
80
70
60
50
40
30
20
10
0
ppm

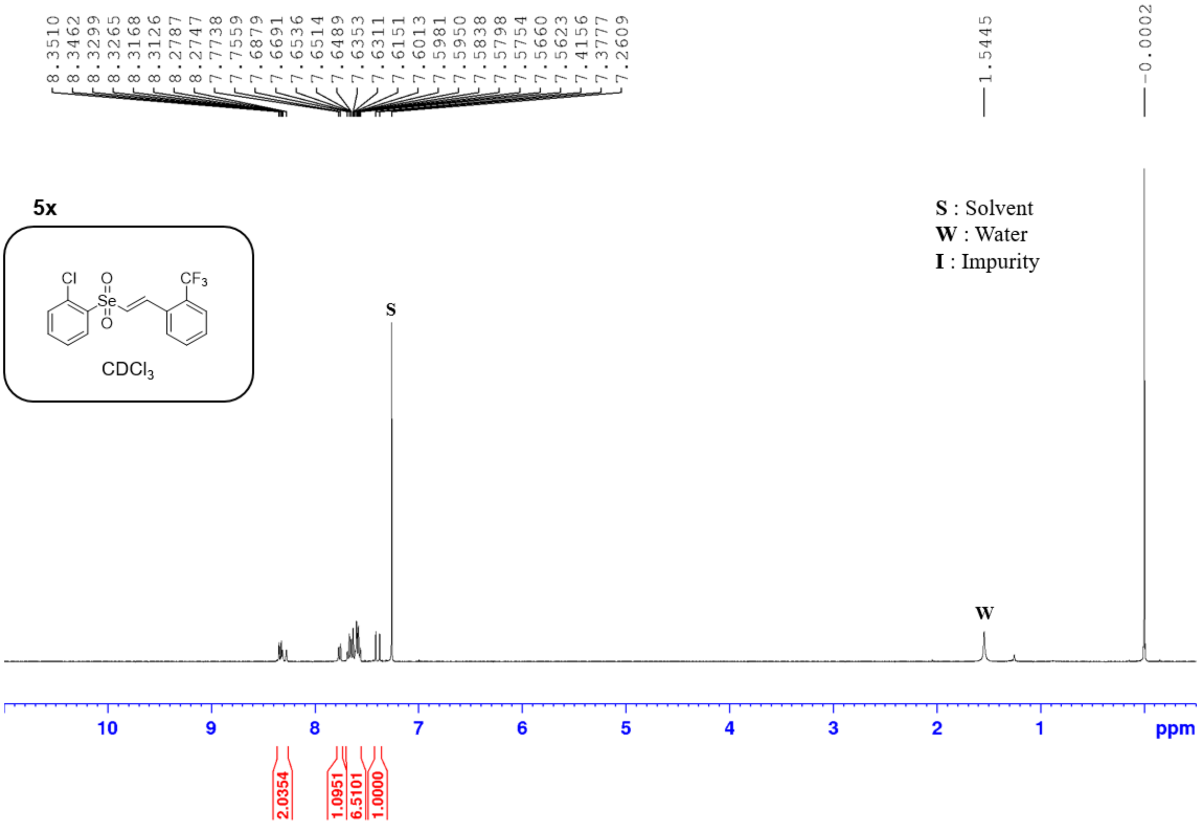
8.3510
8.3462
8.3299
8.3265
8.3168
8.3126
8.2787
8.2747
7.7738
7.7559
7.6879
7.6691
7.6536
7.6514
7.6489
7.6353
7.6311
7.6151
7.6013
7.5981
7.5950
7.5838
7.5798
7.5754
7.5660
7.5623
7.4156
7.3777
7.2609
1.5445
-0.0002
5x
Cl
O
CF3
Se
O
CDCl3
S : Solvent
W : Water
I : Impurity
S
W
10
9
8
7
6
5
4
3
2
1
ppm
2.0354
1.0951
6.5101
1.0000

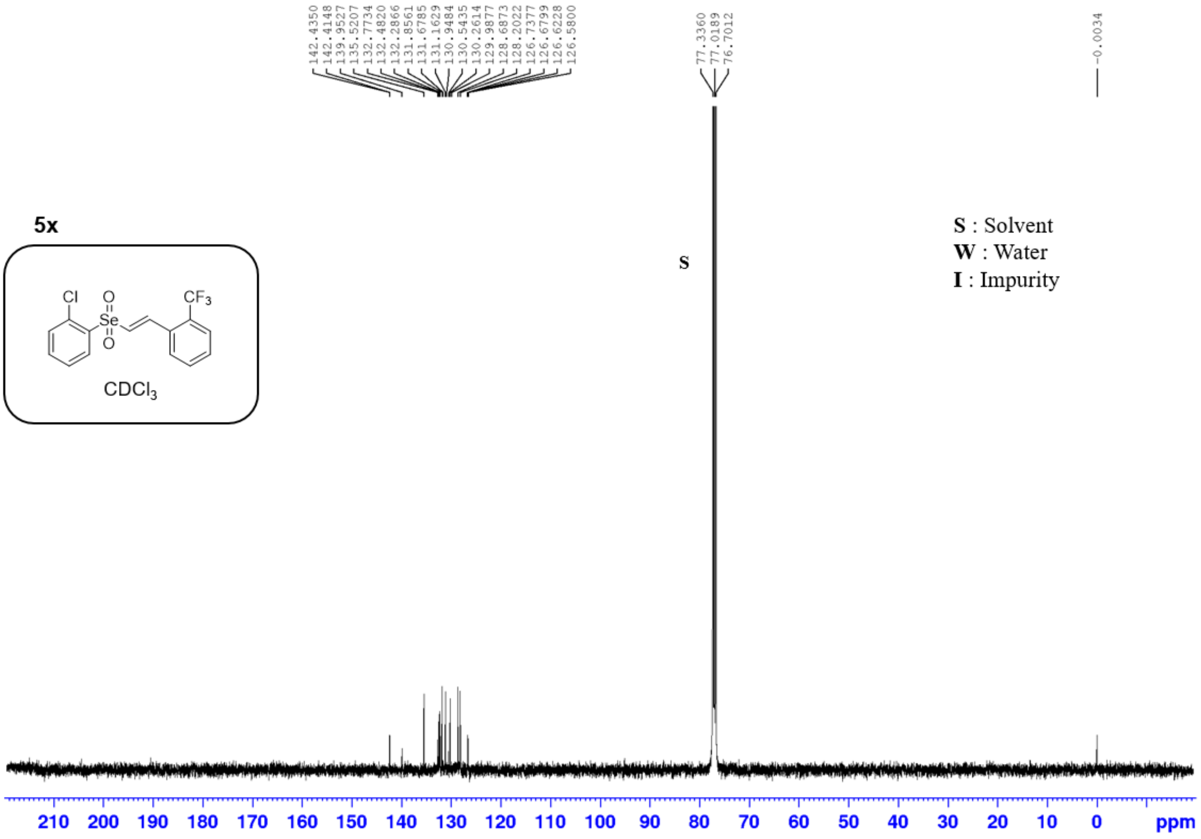
142.4350
142.4148
139.9527
135.5207
132.7734
132.4820
132.2866
131.8561
131.6785
131.1629
130.9484
130.5435
130.2614
129.9877
128.6873
128.2022
126.7377
126.6799
126.6228
126.5800
77.3360
77.0189
76.7012
-0.0034
5x
Cl
O
CF3
Se
O
CDCl3
S : Solvent
W : Water
I : Impurity
S
210
200
190
180
170
160
150
140
130
120
110
100
90
80
70
60
50
40
30
20
10
0
ppm

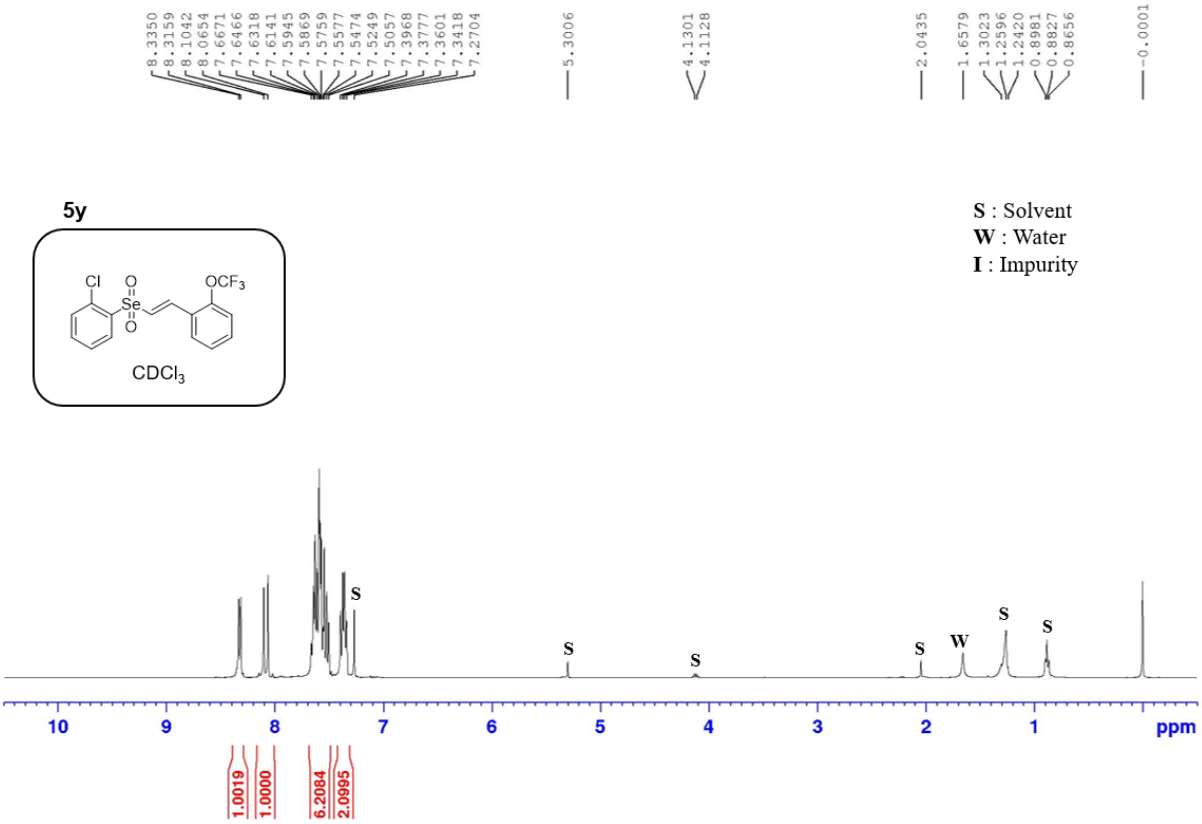
8.3350
8.3159
8.1042
8.0654
7.6671
7.6466
7.6318
7.6141
7.5945
7.5869
7.5759
7.5577
7.5474
7.5249
7.5057
7.3968
7.3777
7.3601
7.3418
7.2704
5.3006
4.1301
4.1128
2.0435
1.6579
1.3023
1.2596
1.2420
0.8981
0.8827
0.8656
-0.0001
5y
Cl
O
Se
O
OCF3
CDCl3
S : Solvent
W : Water
I : Impurity
S
S
S
S
W
S
S
10
9
8
7
6
5
4
3
2
1
ppm
1.0019
1.0000
6.2084
2.0995

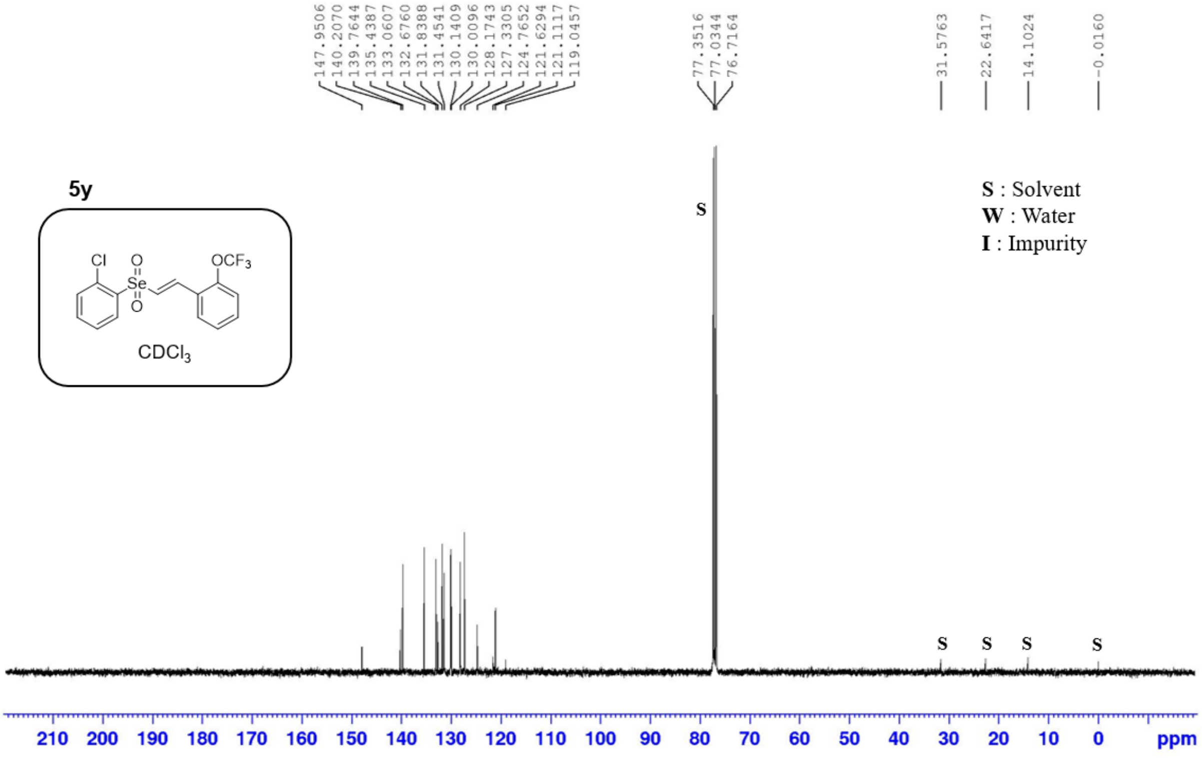
147.9506
140.2070
139.7644
135.4387
133.0607
132.6760
131.8388
131.4541
130.1409
130.0096
128.1743
127.3305
124.7652
121.6294
121.1117
119.0457
77.3516
77.0344
76.7164
31.5763
22.6417
14.1024
-0.0160
5y
Cl
O
Se
O
OCF3
CDCl3
S : Solvent
W : Water
I : Impurity
S
S
S
S
S
210
200
190
180
170
160
150
140
130
120
110
100
90
80
70
60
50
40
30
20
10
0
ppm

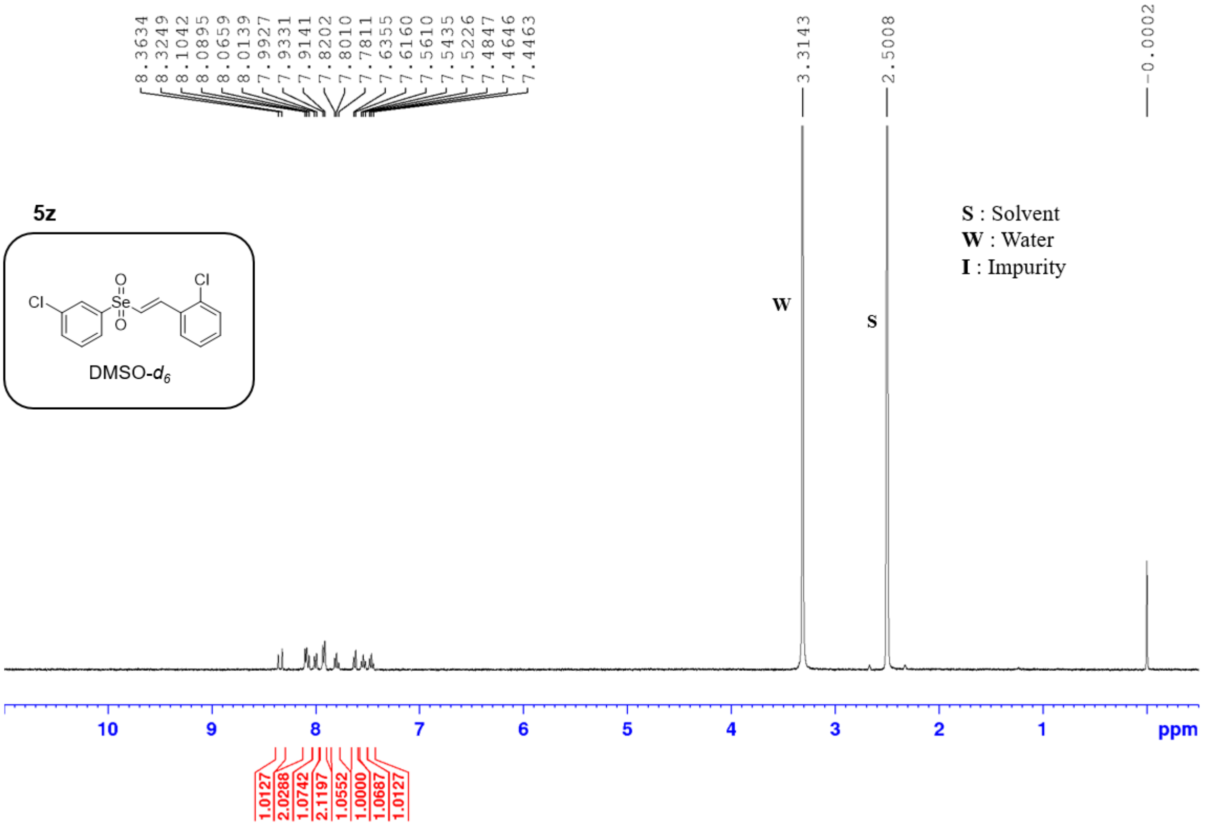
8.3634
8.3249
8.1042
8.0895
8.0659
8.0139
7.9927
7.9331
7.9141
7.8202
7.8010
7.7811
7.6355
7.6160
7.5610
7.5435
7.5226
7.4847
7.4646
7.4463
3.3143
2.5008
-0.0002
5z
Cl
O
Se
O
Cl
DMSO-d_6
W
S
S : Solvent
W : Water
I : Impurity
10
9
8
7
6
5
4
3
2
1
ppm
1.0127
2.0288
1.0742
2.1197
1.0552
1.0000
1.0687
1.0127

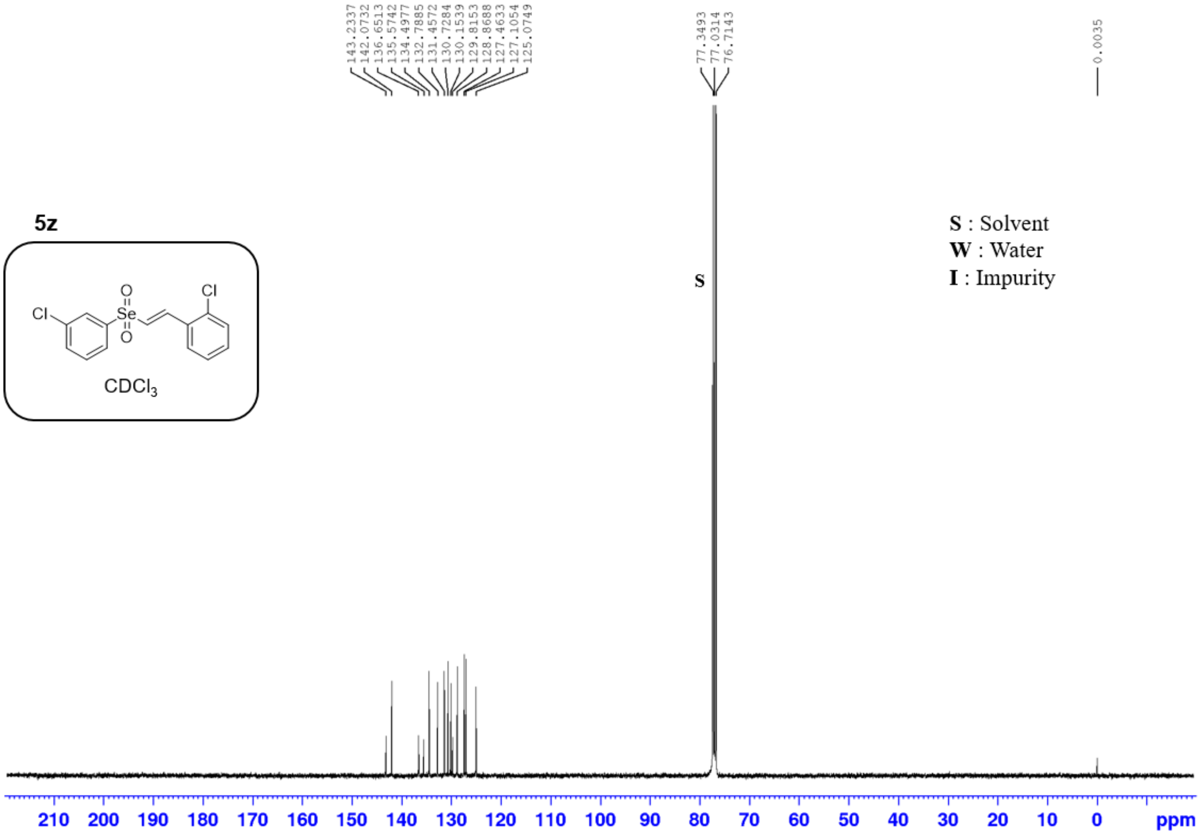
143.2337
142.0732
136.6513
135.5742
134.4977
132.7885
131.4572
130.7284
130.1539
129.8153
128.8688
127.4633
127.1054
125.0749
77.3493
77.0314
76.7143
0.0035
5z
Cl
O
Se
O
Cl
$CDCl_3$
S
S : Solvent
W : Water
I : Impurity
210
200
190
180
170
160
150
140
130
120
110
100
90
80
70
60
50
40
30
20
10
0
ppm

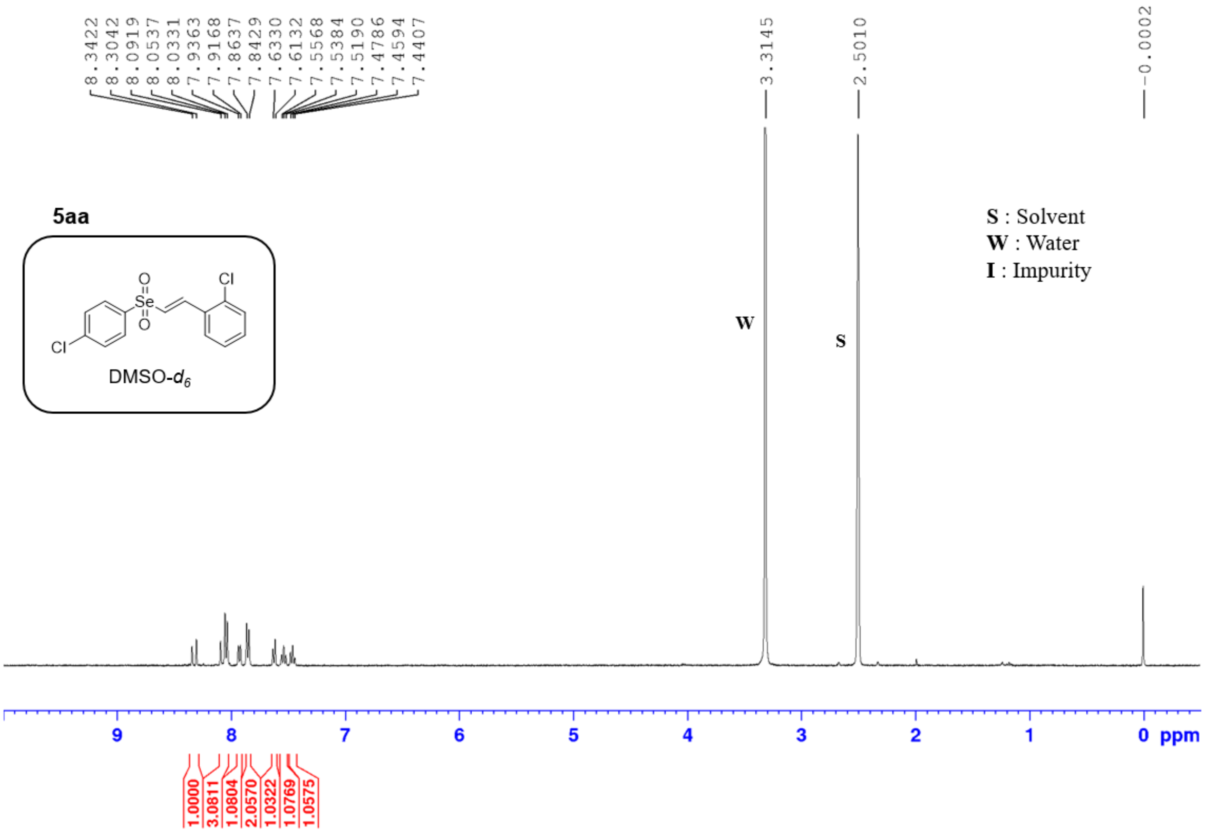

8.3422
8.3042
8.0919
8.0537
8.0331
7.9363
7.9168
7.8637
7.8429
7.6330
7.6132
7.5568
7.5384
7.5190
7.4786
7.4594
7.4407
3.3145
2.5010
-0.0002
5aa
DMSO-d_6
W
S
S : Solvent
W : Water
I : Impurity
1.0000
3.0811
1.0804
2.0570
1.0322
1.0769
1.0575
9
8
7
6
5
4
3
2
1
0 ppm

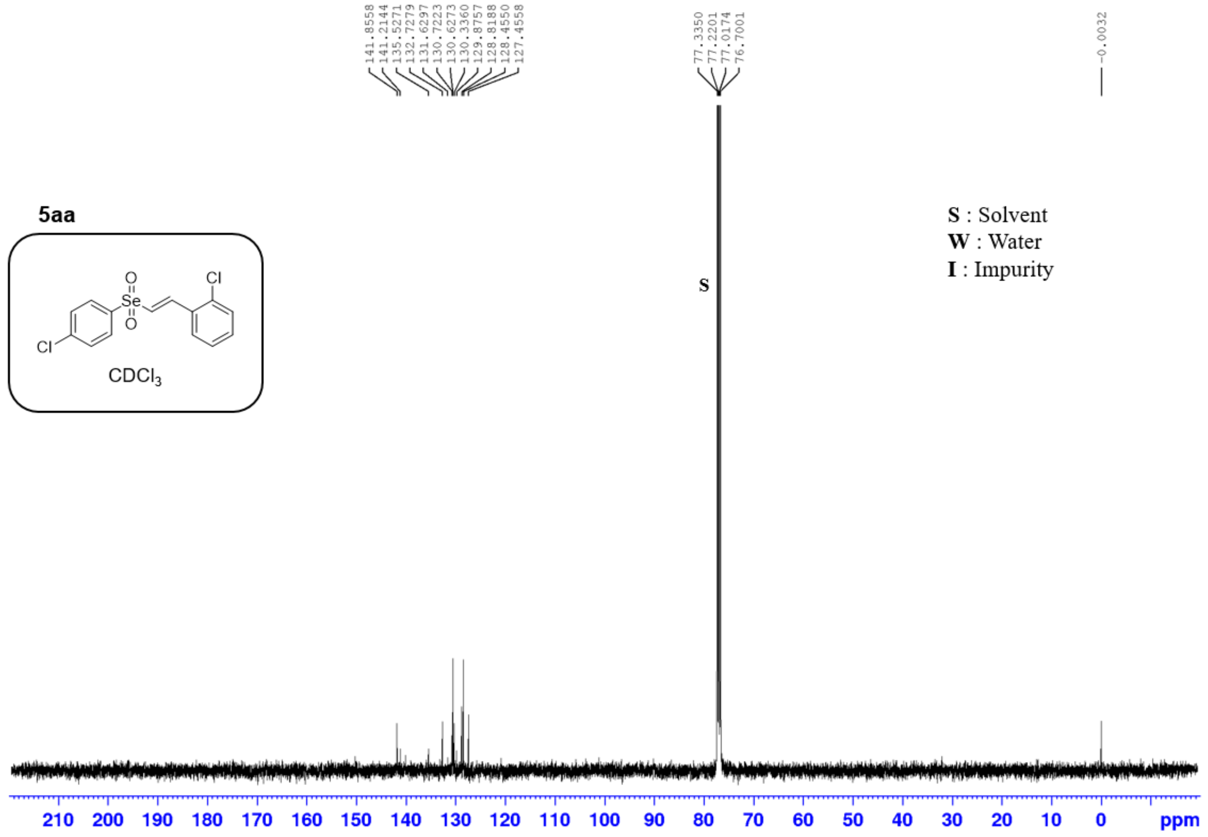

141.8558
141.2144
135.5271
132.7279
131.6297
130.7223
130.6273
130.3360
129.8757
128.8188
128.4550
127.4558
77.3350
77.2201
77.0174
76.7001
-0.0032
5aa
$CDCl_3$
S
S : Solvent
W : Water
I : Impurity
210
200
190
180
170
160
150
140
130
120
110
100
90
80
70
60
50
40
30
20
10
0
ppm

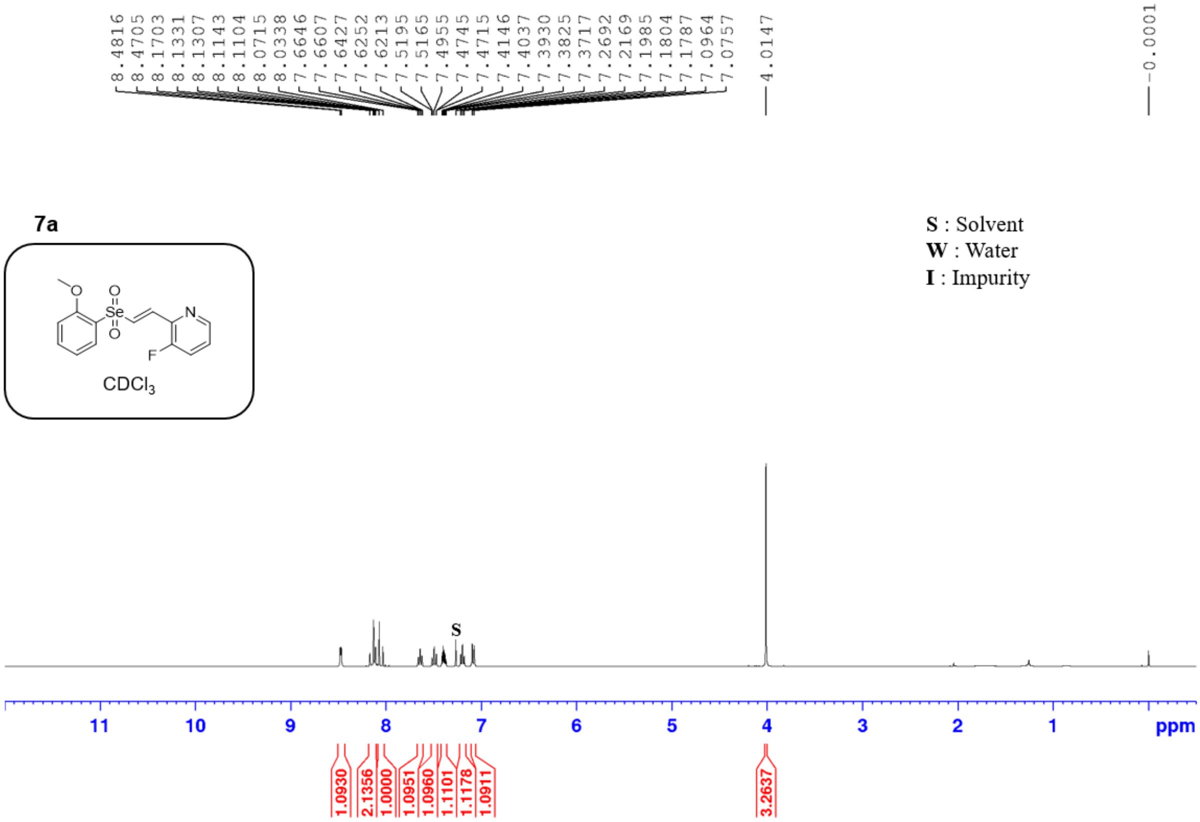

7a
CDCl3
S : Solvent
W : Water
I : Impurity
S
ppm

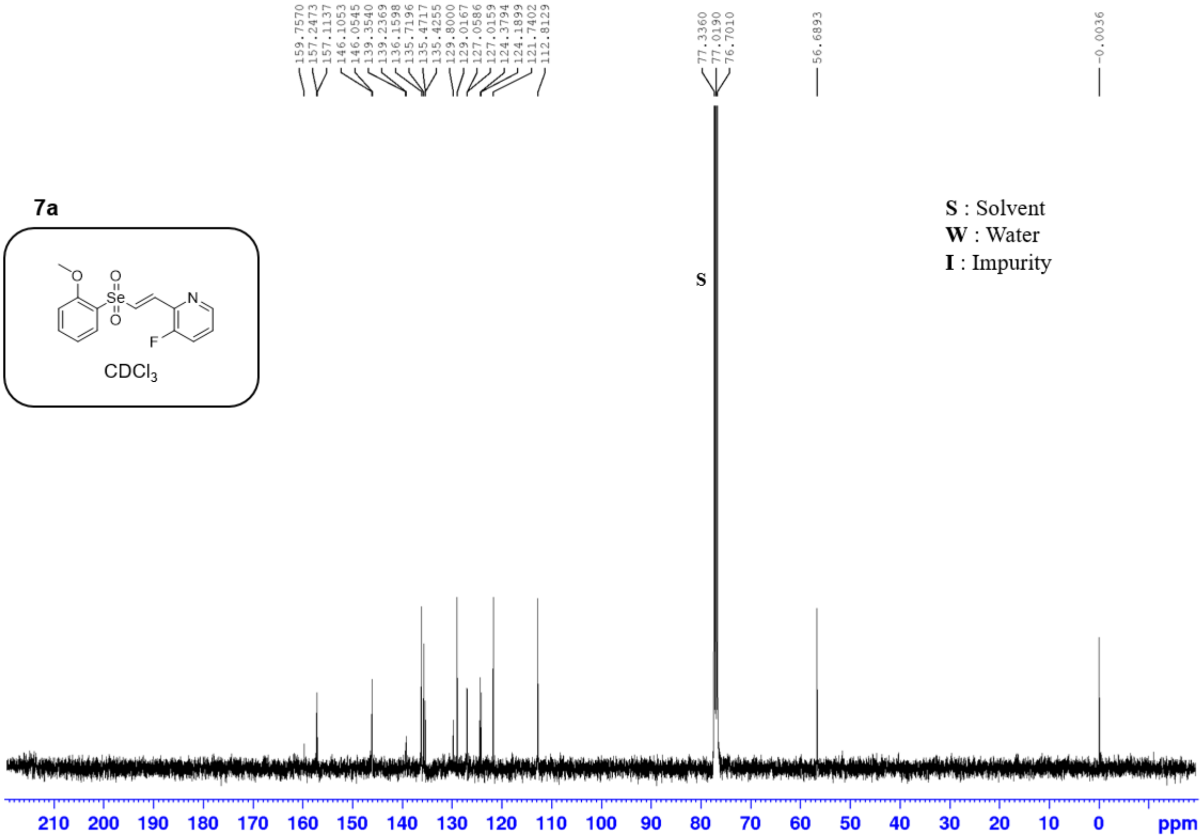

7a
CDCl3
S : Solvent
W : Water
I : Impurity
S
ppm

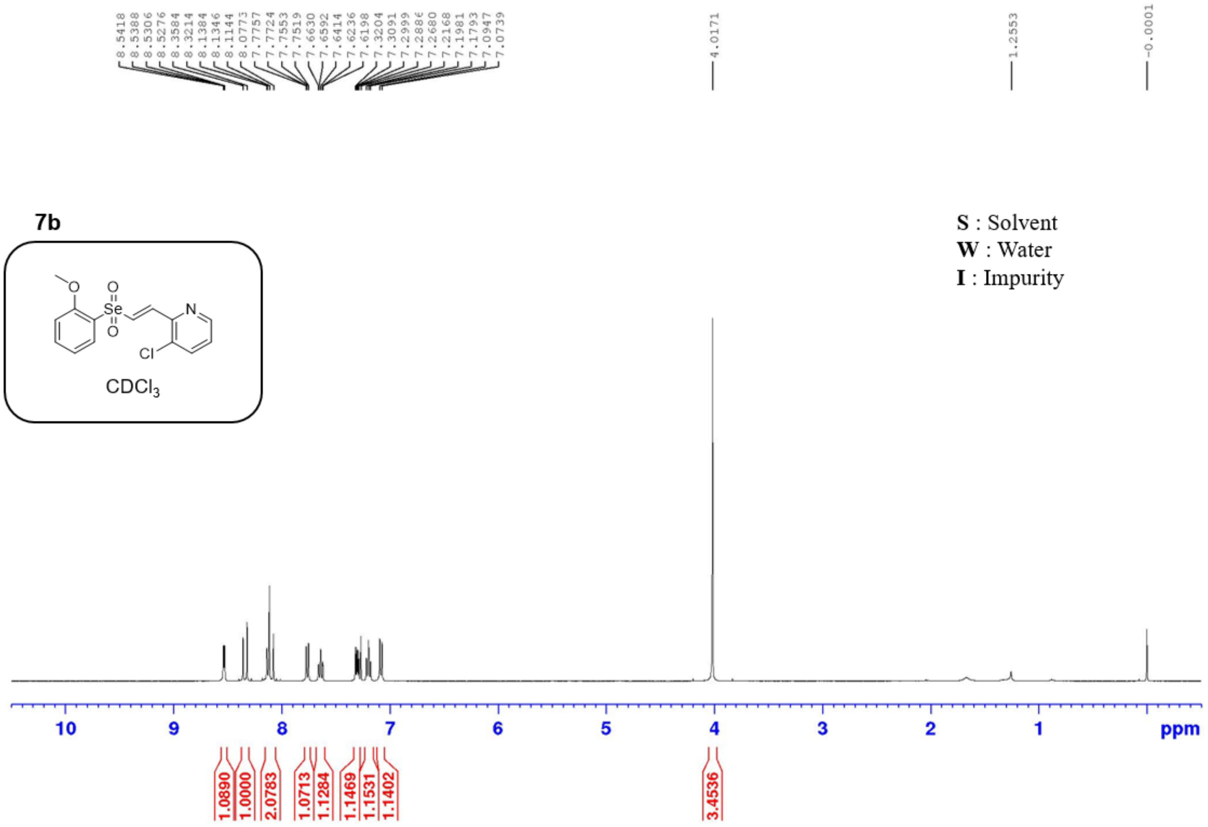
8.5418
8.5388
8.5306
8.5276
8.3584
8.3214
8.1384
8.1346
8.1144
8.0773
7.7757
7.7724
7.7553
7.7519
7.6630
7.6592
7.6414
7.6236
7.6198
7.3204
7.3091
7.2999
7.2886
7.2680
7.2168
7.1981
7.1793
7.0947
7.0739
4.0171
1.2553
-0.0001
7b
CDCl$_3$
S : Solvent
W : Water
I : Impurity
10
9
8
7
6
5
4
3
2
1
ppm
1.0890
1.0000
2.0783
1.0713
1.1284
1.1469
1.1531
1.1402
3.4536

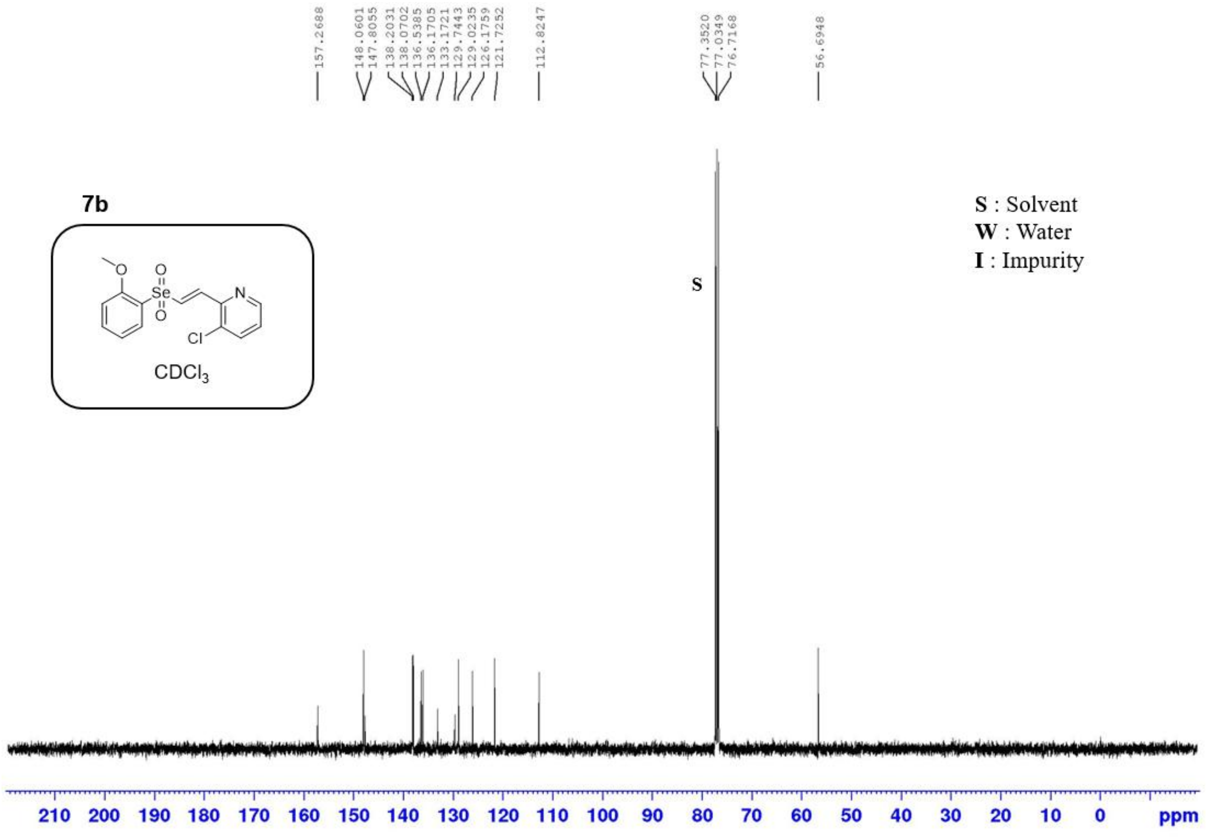
157.2688
148.0601
147.8055
138.2031
138.0702
136.5385
136.1705
133.1721
129.7443
129.0235
126.1759
121.7252
112.8247
77.3520
77.0349
76.7168
56.6948
7b
CDCl$_3$
S : Solvent
W : Water
I : Impurity
S
210
200
190
180
170
160
150
140
130
120
110
100
90
80
70
60
50
40
30
20
10
0
ppm

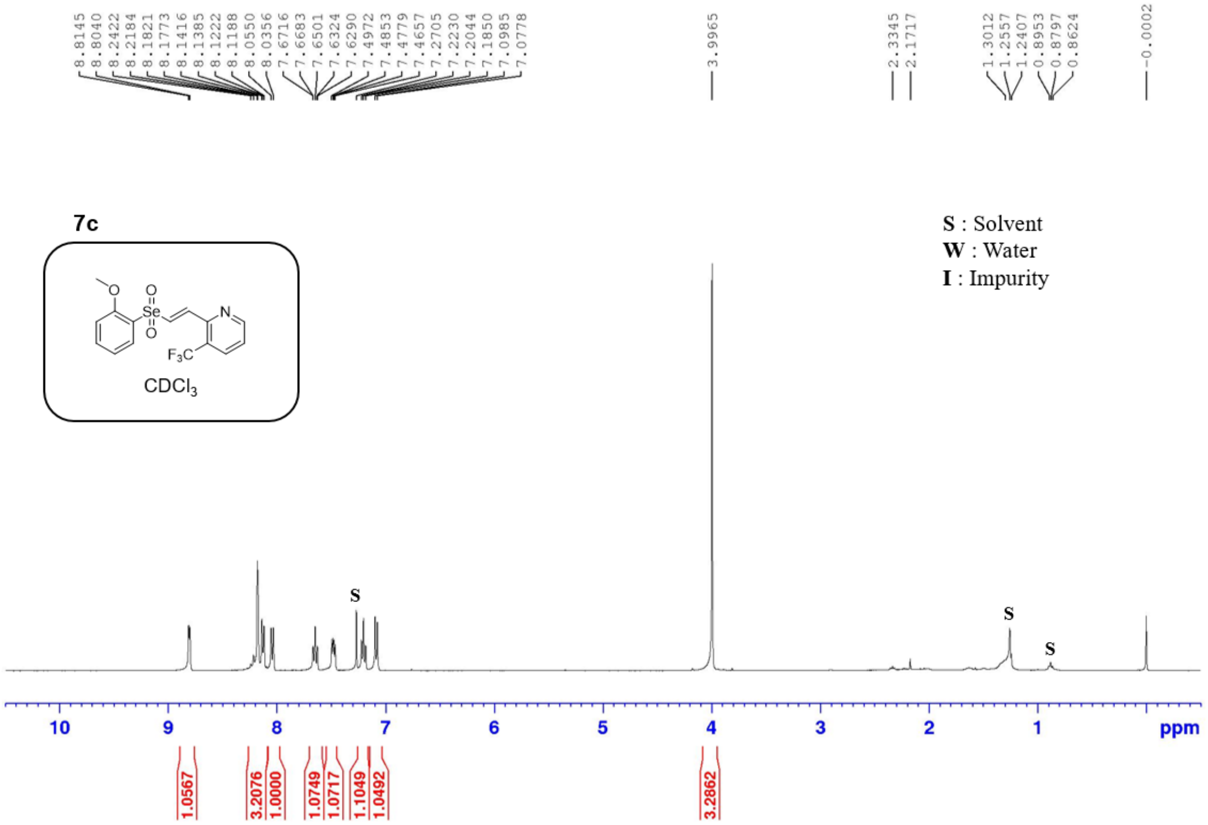

8.8145
8.8040
8.2422
8.2184
8.1821
8.1773
8.1416
8.1385
8.1222
8.1188
8.0550
8.0356
7.6716
7.6683
7.6501
7.6324
7.6290
7.4972
7.4853
7.4779
7.4657
7.2705
7.2230
7.2044
7.1850
7.0985
7.0778
3.9965
2.3345
2.1717
1.3012
1.2557
1.2407
0.8953
0.8797
0.8624
-0.0002
7c
CDCl3
S : Solvent
W : Water
I : Impurity
S
S
S
1.0567
3.2076
1.0000
1.0749
1.0717
1.1049
1.0492
3.2862
ppm

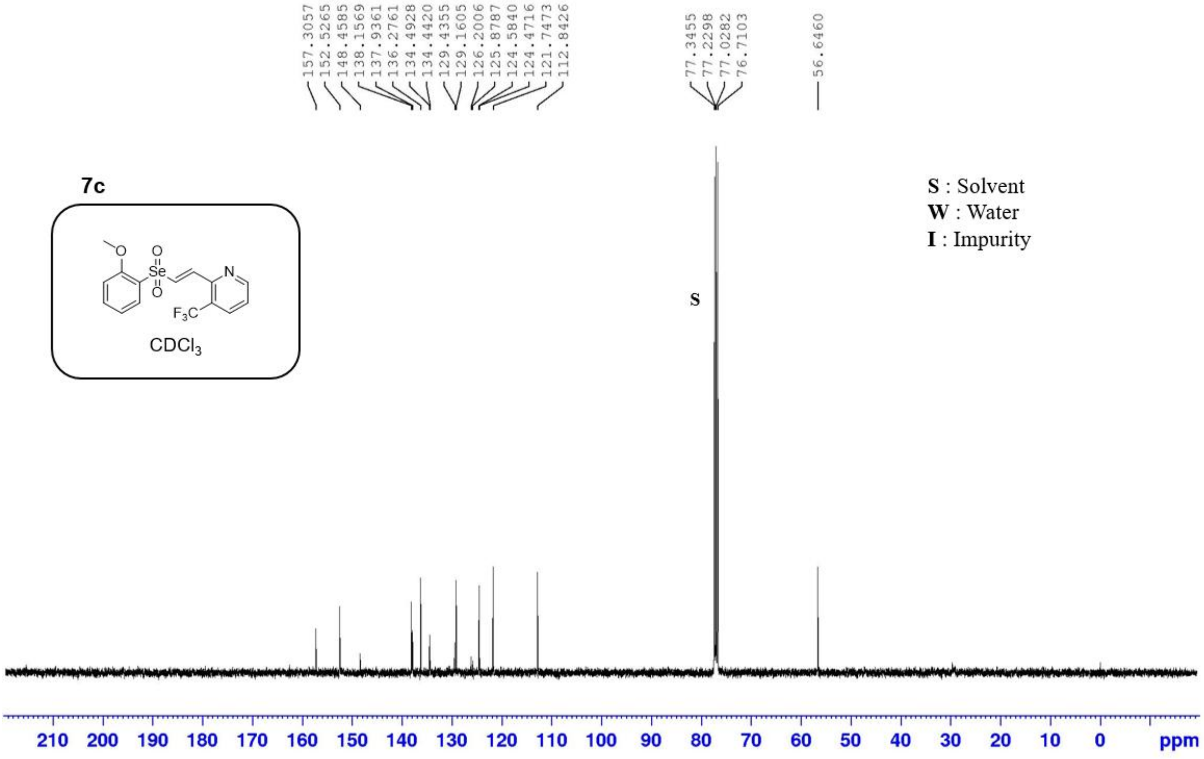

157.3057
152.5265
148.4585
138.1569
137.9361
136.2761
134.4928
134.4420
129.4355
129.1605
126.2006
125.8787
124.5840
124.4716
121.7473
112.8426
77.3455
77.2298
77.0282
76.7103
56.6460
7c
CDCl3
S : Solvent
W : Water
I : Impurity
S
ppm

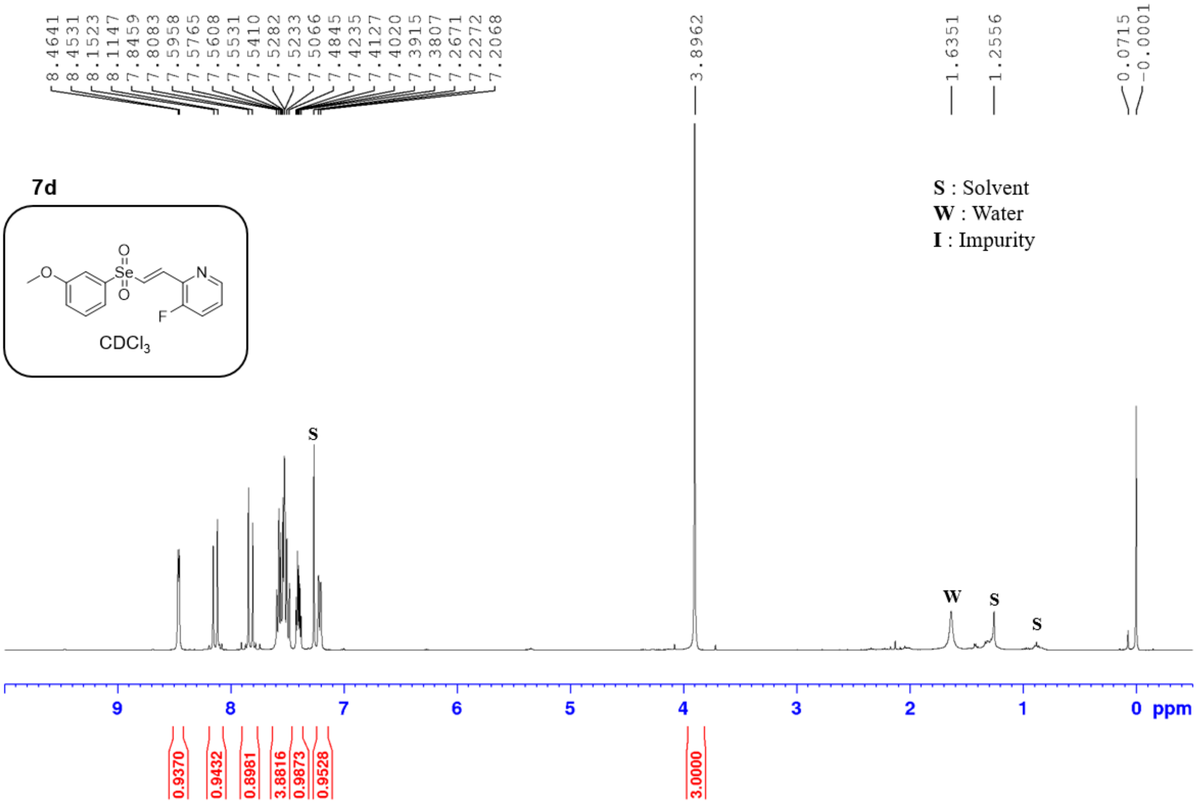
8.4641
8.4531
8.1523
8.1147
7.8459
7.8083
7.5958
7.5765
7.5608
7.5531
7.5410
7.5282
7.5233
7.5066
7.4845
7.4235
7.4127
7.4020
7.3915
7.3807
7.2671
7.2272
7.2068
3.8962
1.6351
1.2556
0.0715
-0.0001
7d
CDCl3
S : Solvent
W : Water
I : Impurity
ppm
0.9370
0.9432
0.8981
3.8816
0.9873
0.9528
3.0000

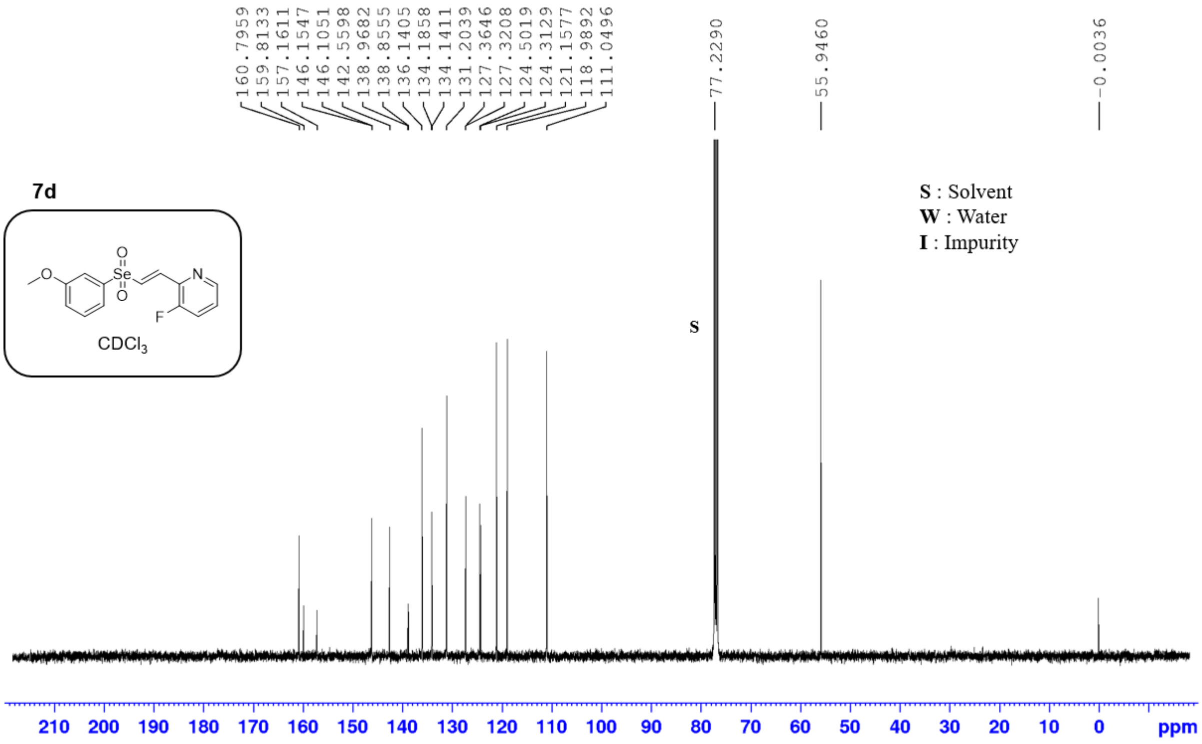
160.7959
159.8133
157.1611
146.1547
146.1051
142.5598
138.9682
138.8555
136.1405
134.1858
134.1411
131.2039
127.3646
127.3208
124.5019
124.3129
121.1577
118.9892
111.0496
77.2290
55.9460
-0.0036
7d
CDCl3
S : Solvent
W : Water
I : Impurity
ppm

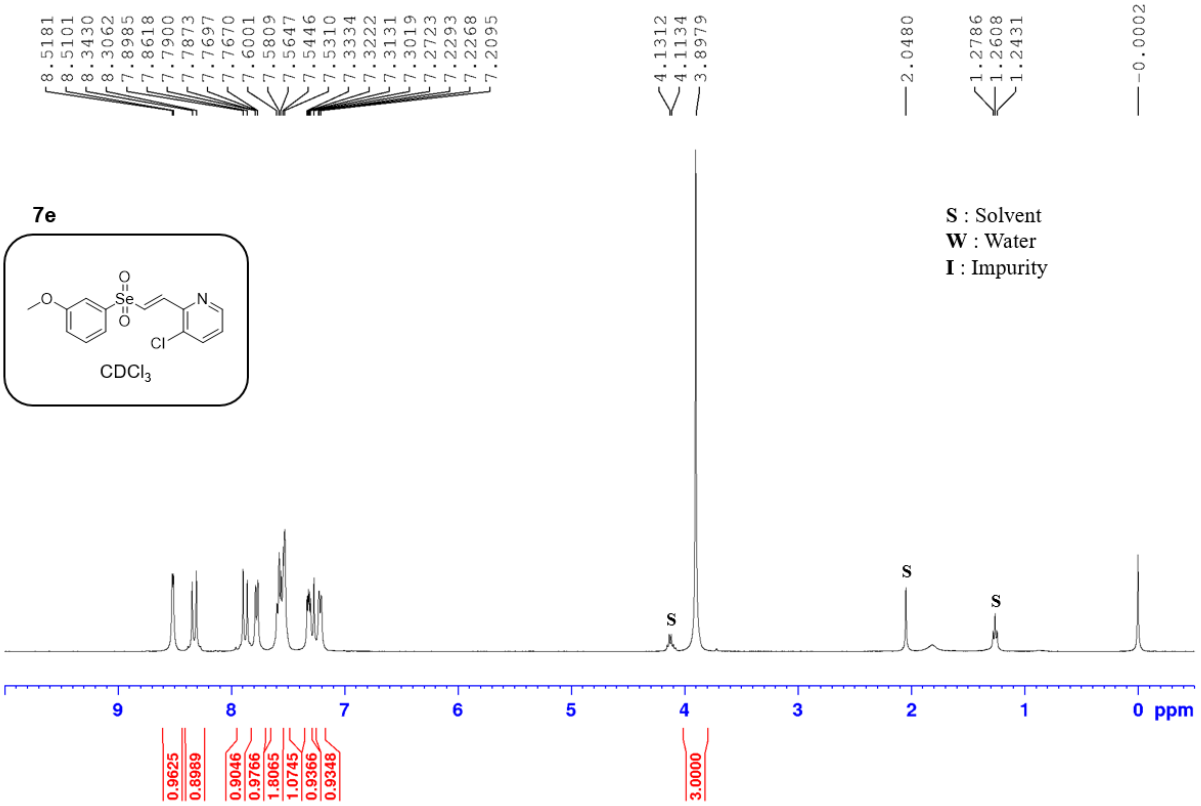
8.5181
8.5101
8.3430
8.3062
7.8985
7.8618
7.7900
7.7873
7.7697
7.7670
7.6001
7.5809
7.5647
7.5446
7.5310
7.3334
7.3222
7.3131
7.3019
7.2723
7.2293
7.2268
7.2095
4.1312
4.1134
3.8979
2.0480
1.2786
1.2608
1.2431
-0.0002
7e
O
Se
O
N
Cl
CDCl3
S : Solvent
W : Water
I : Impurity
S
S
S
9
8
7
6
5
4
3
2
1
0 ppm
0.9625
0.8989
0.9046
0.9766
1.8065
1.0745
0.9366
0.9348
3.0000

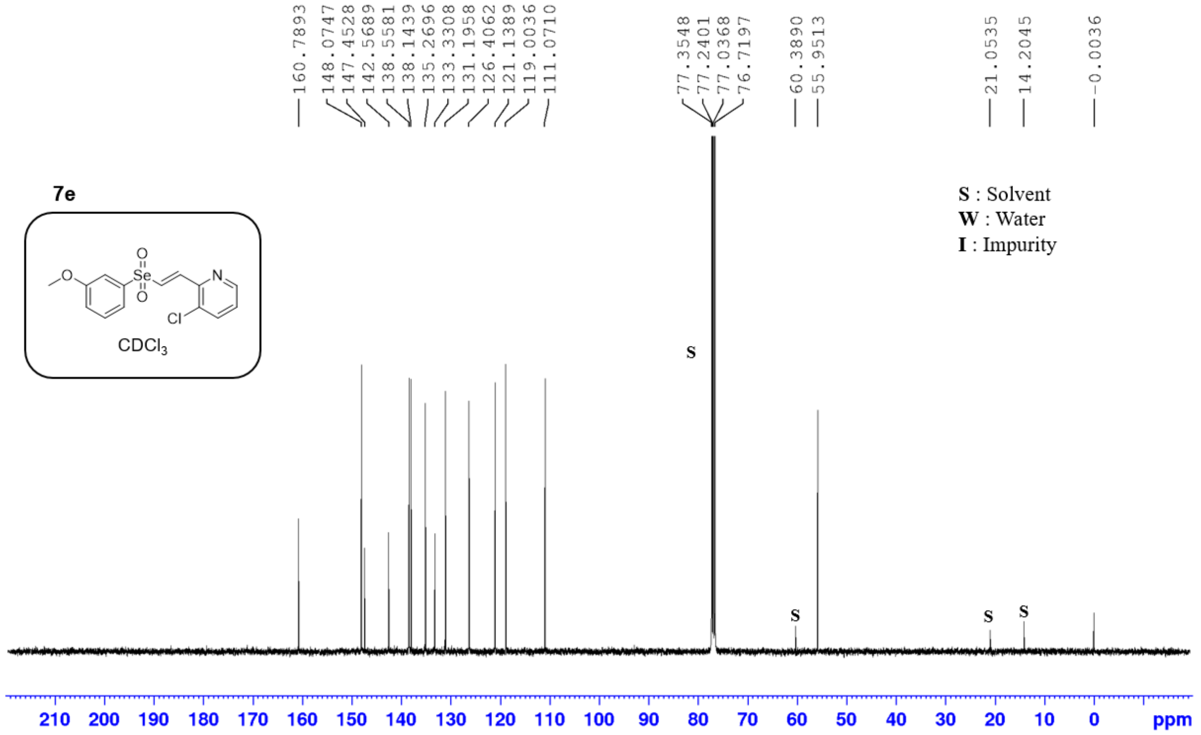
160.7893
148.0747
147.4528
142.5689
138.5581
138.1439
135.2696
133.3308
131.1958
126.4062
121.1389
119.0036
111.0710
77.3548
77.2401
77.0368
76.7197
60.3890
55.9513
21.0535
14.2045
-0.0036
7e
O
Se
O
N
Cl
CDCl3
S : Solvent
W : Water
I : Impurity
S
S
S
S
210
200
190
180
170
160
150
140
130
120
110
100
90
80
70
60
50
40
30
20
10
0
ppm

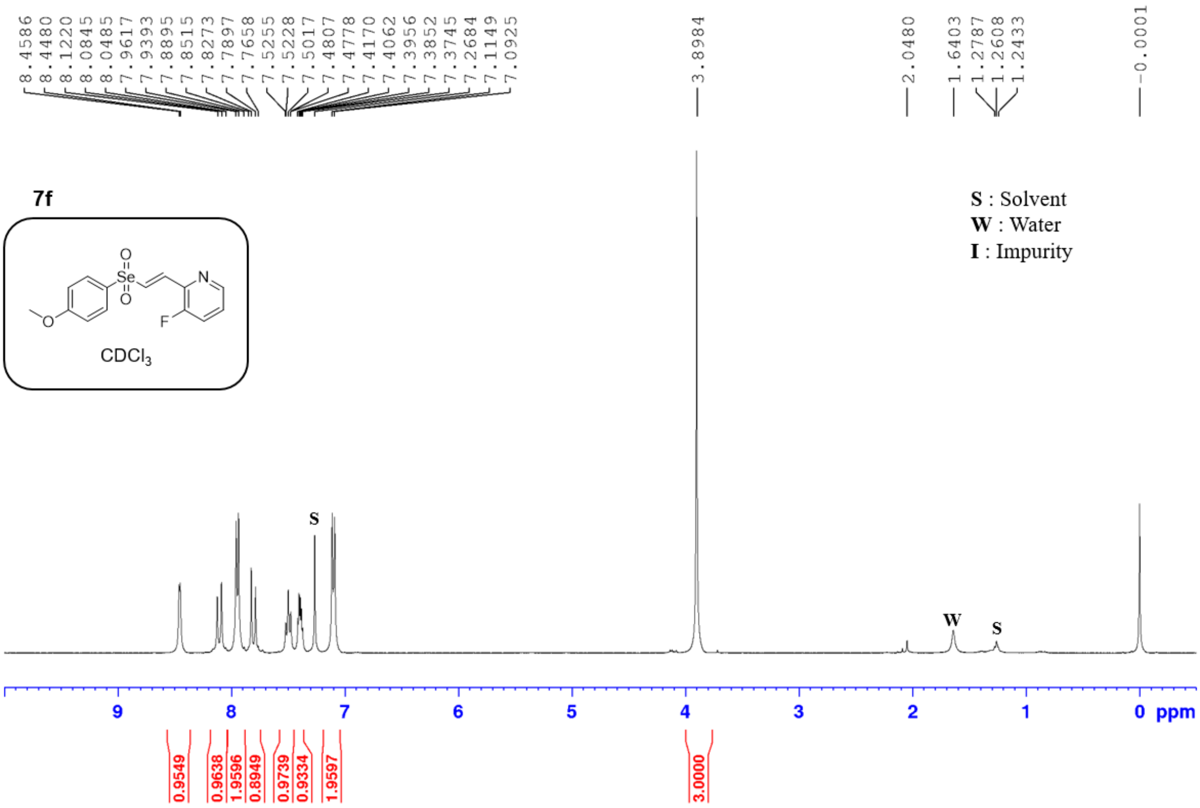
8.4586
8.4480
8.1220
8.0845
8.0485
7.9617
7.9393
7.8895
7.8515
7.8273
7.7897
7.7658
7.5255
7.5228
7.5017
7.4807
7.4778
7.4170
7.4062
7.3956
7.3852
7.3745
7.2684
7.1149
7.0925
3.8984
2.0480
1.6403
1.2787
1.2608
1.2433
-0.0001
7f
Se
N
O
F
CDCl3
S : Solvent
W : Water
I : Impurity
S
W
S
9
8
7
6
5
4
3
2
1
0 ppm
0.9549
0.9638
1.9596
0.8949
0.9739
0.9334
1.9597
3.0000

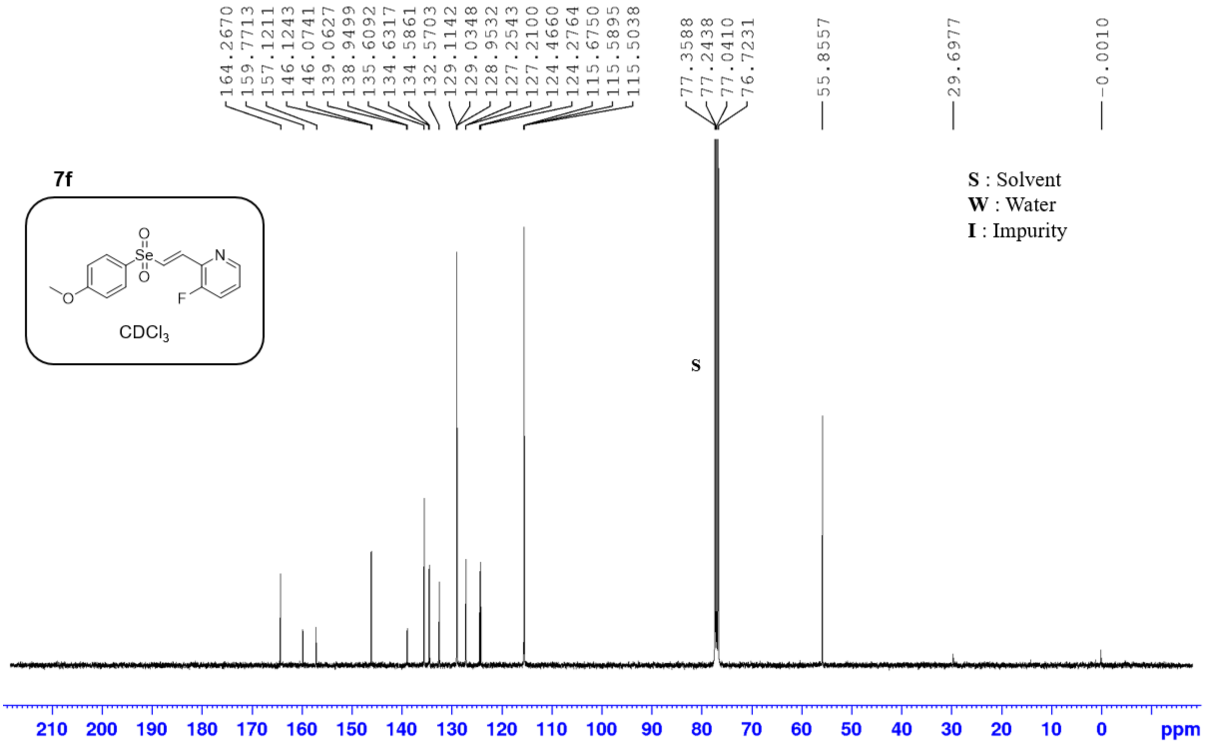
164.2670
159.7713
157.1211
146.1243
146.0741
139.0627
138.9499
135.6092
134.6317
134.5861
132.5703
129.1142
129.0348
128.9532
127.2543
127.2100
124.4660
124.2764
115.6750
115.5895
115.5038
77.3588
77.2438
77.0410
76.7231
55.8557
29.6977
-0.0010
7f
Se
N
O
F
CDCl3
S : Solvent
W : Water
I : Impurity
S
210
200
190
180
170
160
150
140
130
120
110
100
90
80
70
60
50
40
30
20
10
0
ppm

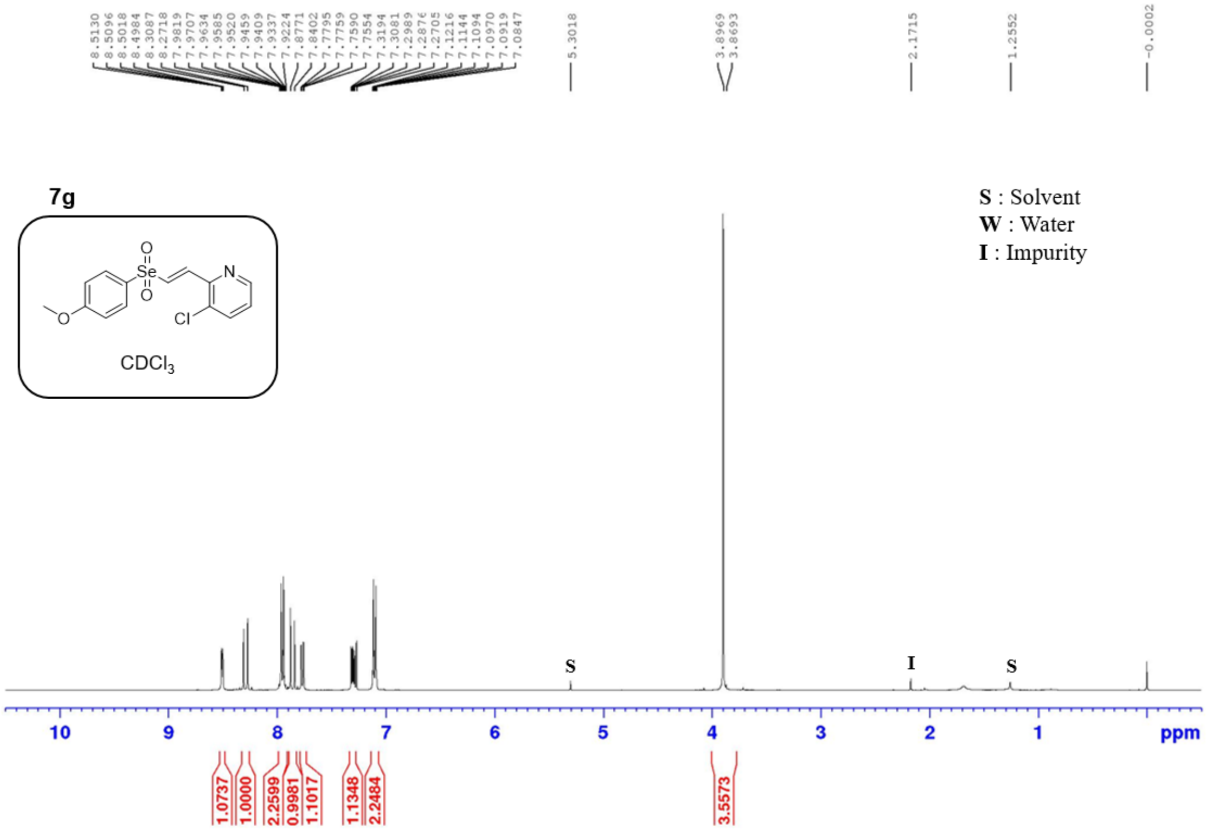
8.5130
8.5096
8.5018
8.4984
8.3087
8.2718
7.9819
7.9707
7.9634
7.9585
7.9520
7.9459
7.9409
7.9337
7.9224
7.8771
7.8402
7.7795
7.7759
7.7590
7.7554
7.3194
7.3081
7.2989
7.2876
7.2705
7.1216
7.1144
7.1094
7.0970
7.0919
7.0847
5.3018
3.8969
3.8693
2.1715
1.2552
-0.0002
7g
CDCl3
S : Solvent
W : Water
I : Impurity
S
I
S
10
9
8
7
6
5
4
3
2
1
ppm
1.0737
1.0000
2.2599
0.9981
1.1017
1.1348
2.2484
3.5573

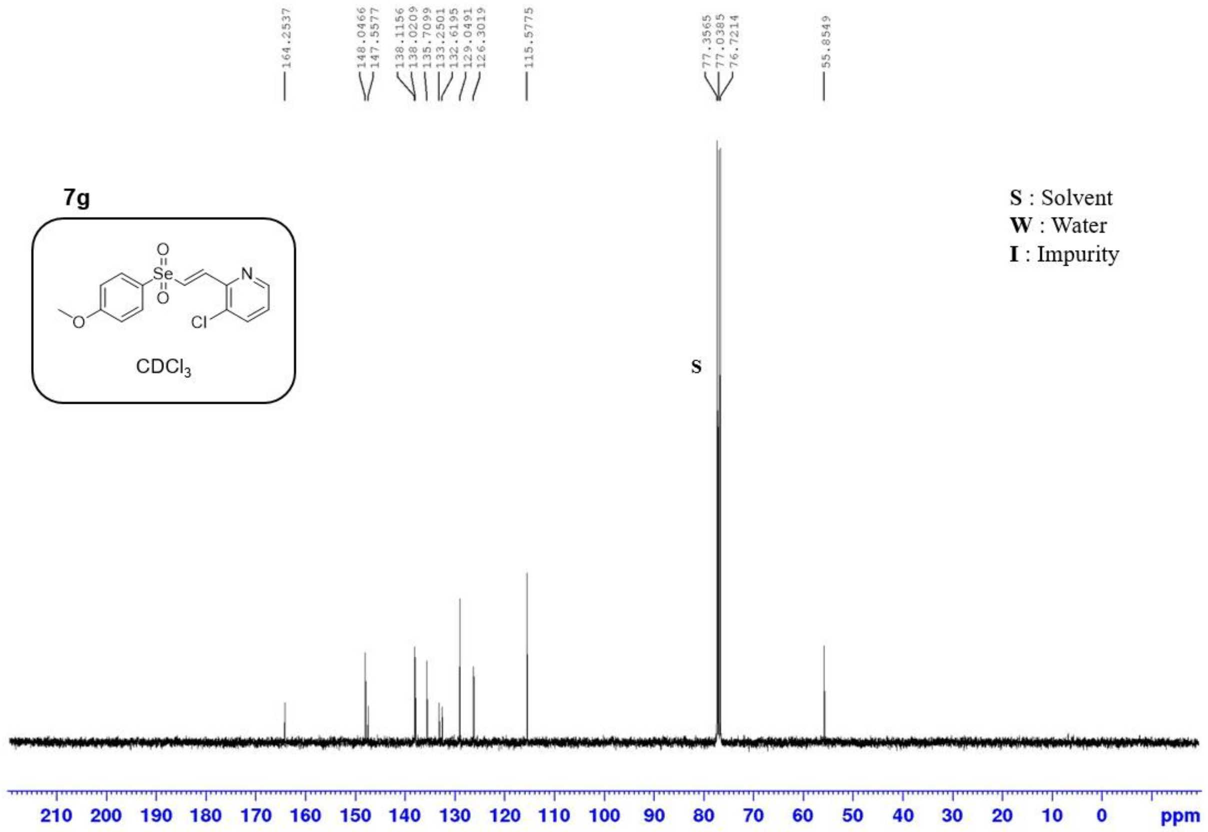
164.2537
148.0466
147.5577
138.1156
138.0209
135.7099
133.2501
132.6195
129.0491
126.3019
115.5775
77.3565
77.0385
76.7214
55.8549
7g
CDCl3
S : Solvent
W : Water
I : Impurity
S
210
200
190
180
170
160
150
140
130
120
110
100
90
80
70
60
50
40
30
20
10
0
ppm

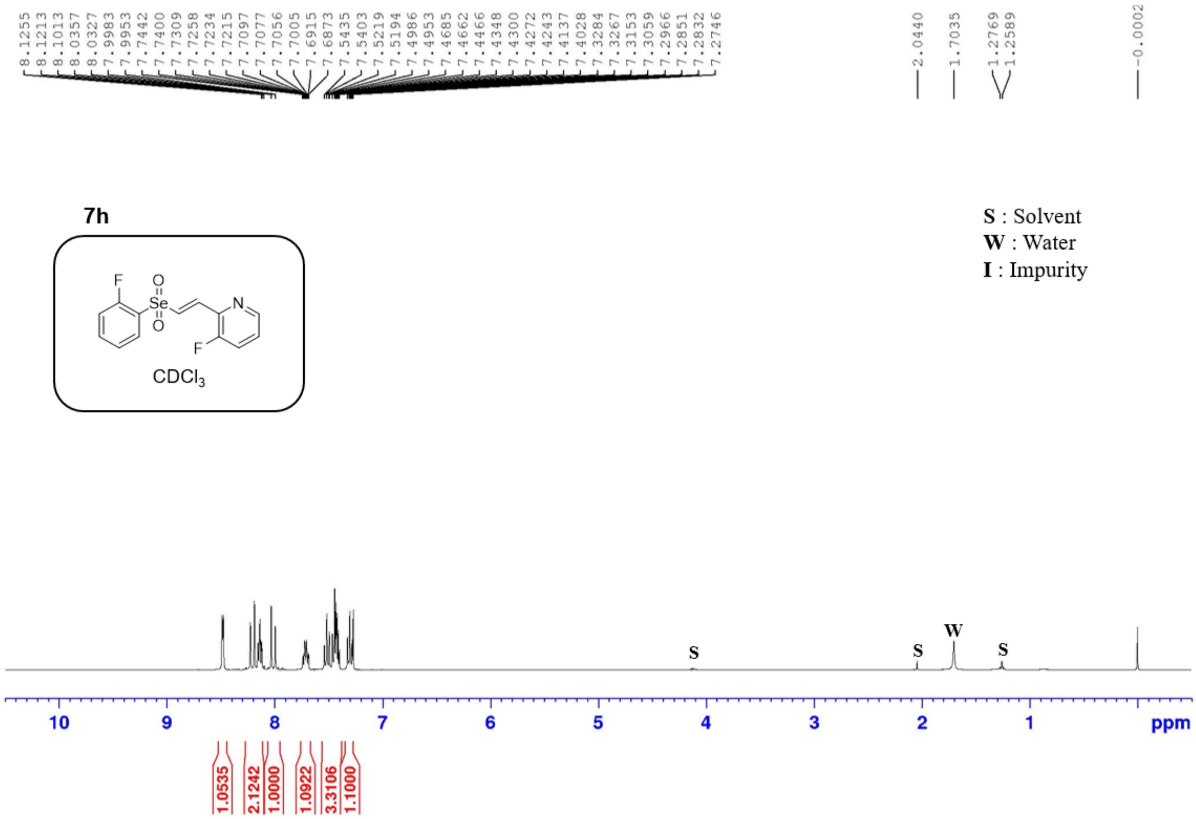
8.1255
8.1213
8.1013
8.0357
8.0327
7.9983
7.9953
7.7442
7.7400
7.7309
7.7258
7.7234
7.7215
7.7097
7.7077
7.7056
7.7005
7.6915
7.6873
7.5435
7.5403
7.5219
7.5194
7.4986
7.4953
7.4685
7.4662
7.4466
7.4348
7.4300
7.4272
7.4243
7.4137
7.4028
7.3284
7.3267
7.3153
7.3059
7.2966
7.2851
7.2832
7.2746
2.0440
1.7035
1.2769
1.2589
-0.0002
7h
CDCl3
S : Solvent
W : Water
I : Impurity
1.0535
2.1242
1.0000
1.0922
3.3106
1.1000
ppm

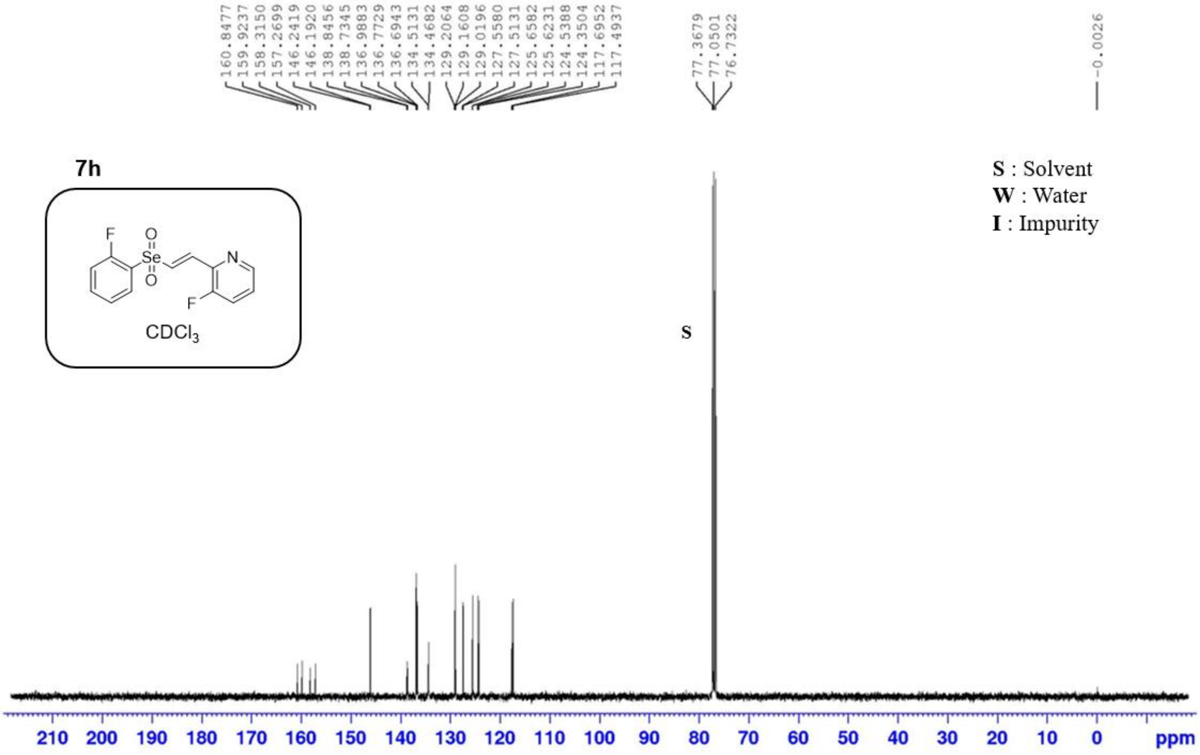
160.8477
159.9237
158.3150
157.2699
146.2419
146.1920
138.8456
138.7345
136.9883
136.7729
136.6943
134.5131
134.4682
129.2064
129.1608
129.0196
127.5580
127.5131
125.6582
125.6231
124.5388
124.3504
117.6952
117.4937
77.3679
77.0501
76.7322
-0.0026
7h
CDCl3
S : Solvent
W : Water
I : Impurity
ppm

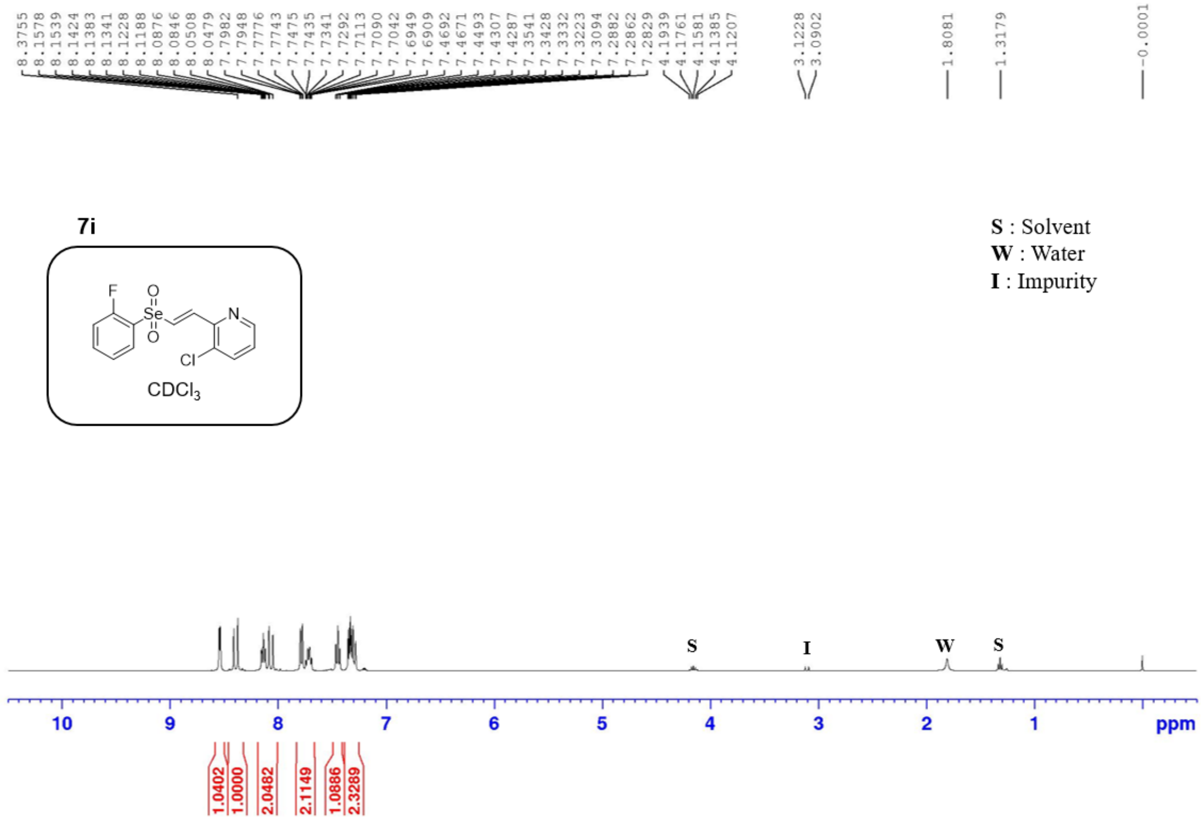
8.3755 8.1578 8.1539 8.1424 8.1383 8.1341 8.1228 8.1188 8.0876 8.0846 8.0508 8.0479 7.7982 7.7948 7.7776 7.7743 7.7475 7.7435 7.7341 7.7292 7.7113 7.7090 7.7042 7.6949 7.6909 7.4692 7.4671 7.4493 7.4307 7.4287 7.3541 7.3428 7.3332 7.3223 7.3094 7.2882 7.2862 7.2829 4.1939 4.1761 4.1581 4.1385 4.1207
3.1228 3.0902
1.8081
1.3179
-0.0001
7i
F
O
Se
O
N
Cl
$CDCl_3$
S : Solvent
W : Water
I : Impurity
S
I
W
S
10 9 8 7 6 5 4 3 2 1 ppm
1.0402
1.0000
2.0482
2.1149
1.0886
2.3289

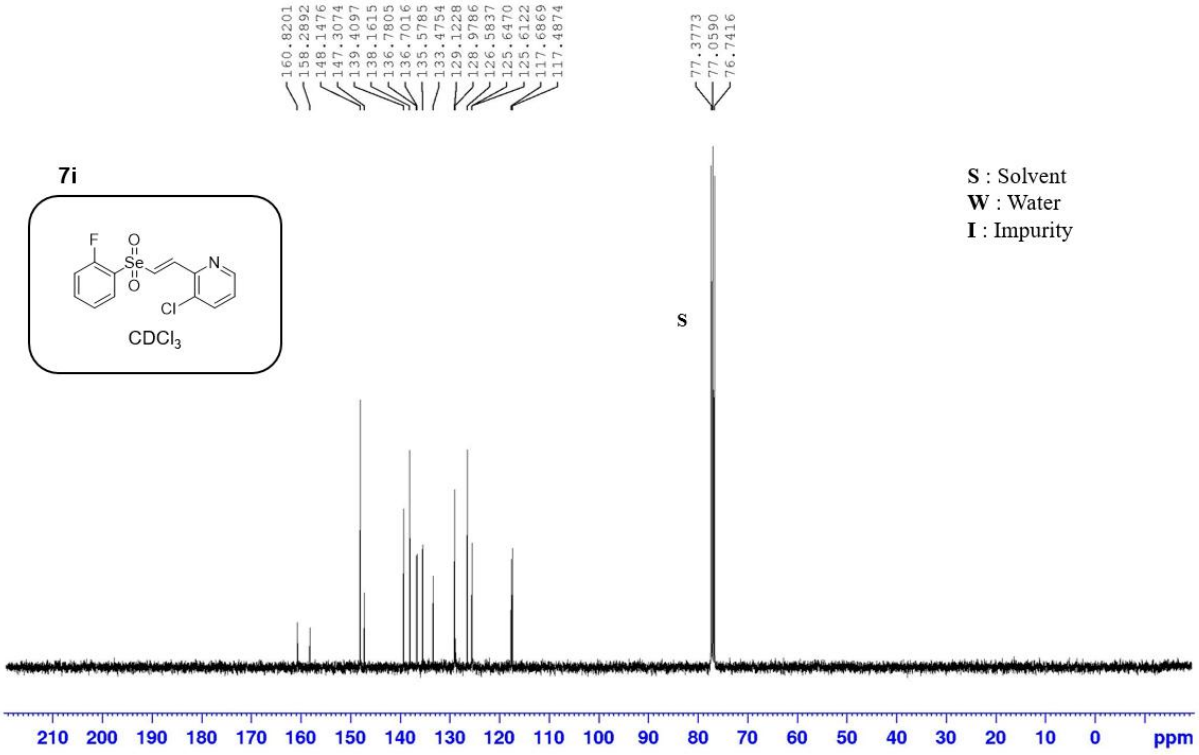
160.8201 158.2892 148.1476 147.3074 139.4097 138.1615 136.7805 136.7016 135.5785 133.4754 129.1228 128.9786 126.5837 125.6470 125.6122 117.6869 117.4874
77.3773 77.0590 76.7416
7i
F
O
Se
O
N
Cl
$CDCl_3$
S : Solvent
W : Water
I : Impurity
S
210 200 190 180 170 160 150 140 130 120 110 100 90 80 70 60 50 40 30 20 10 0 ppm

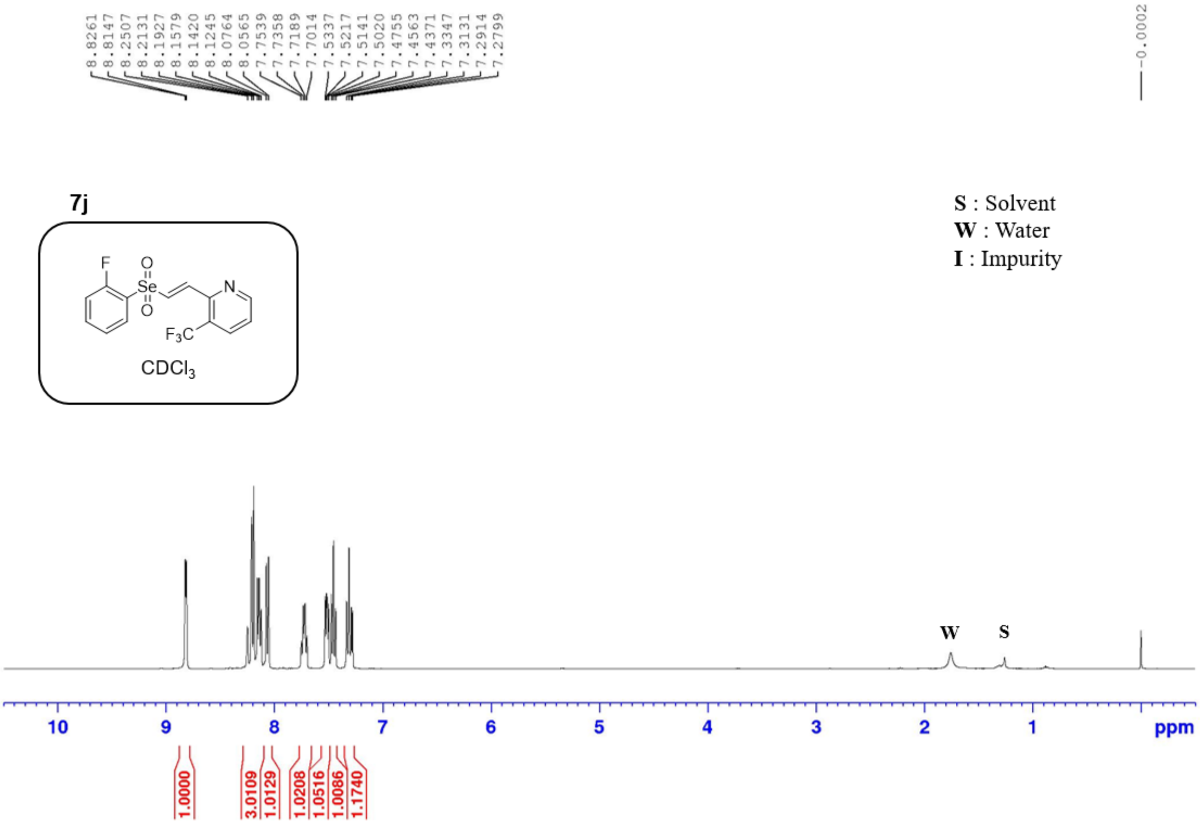
8.8261
8.8147
8.2507
8.2131
8.1927
8.1579
8.1420
8.1245
8.0764
8.0565
7.7539
7.7358
7.7189
7.7014
7.5337
7.5217
7.5141
7.5020
7.4755
7.4563
7.4371
7.3347
7.3131
7.2914
7.2799
-0.0002
7j
F
Se
O
O
N
F_3C
$CDCl_3$
S : Solvent
W : Water
I : Impurity
W
S
10 9 8 7 6 5 4 3 2 1 ppm
1.0000
3.0109
1.0129
1.0208
1.0516
1.0086
1.1740

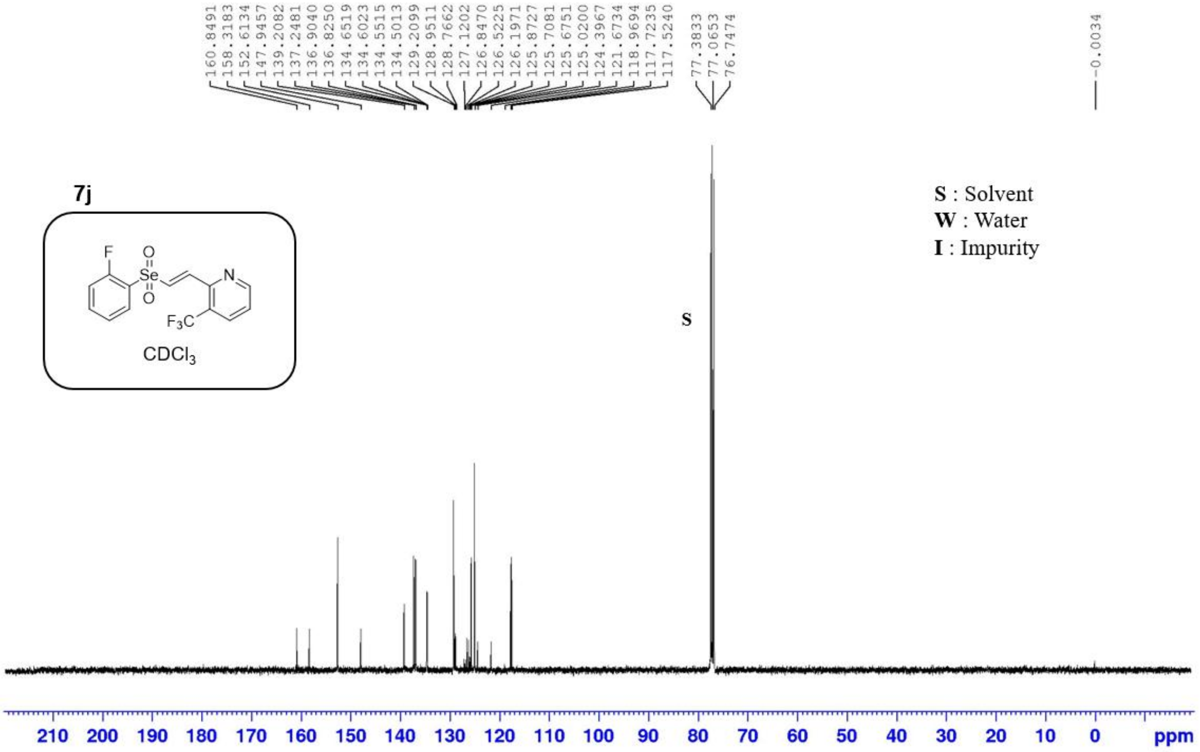
160.8491
158.3183
152.6134
147.9457
139.2082
137.2481
136.9040
136.8250
134.6519
134.6023
134.5515
134.5013
129.2099
128.9511
128.7662
127.1202
126.8470
126.5225
126.1971
125.8727
125.7081
125.6751
125.0200
124.3967
121.6734
118.9694
117.7235
117.5240
77.3833
77.0653
76.7474
-0.0034
7j
F
Se
O
O
N
F_3C
$CDCl_3$
S : Solvent
W : Water
I : Impurity
S
210 200 190 180 170 160 150 140 130 120 110 100 90 80 70 60 50 40 30 20 10 0 ppm

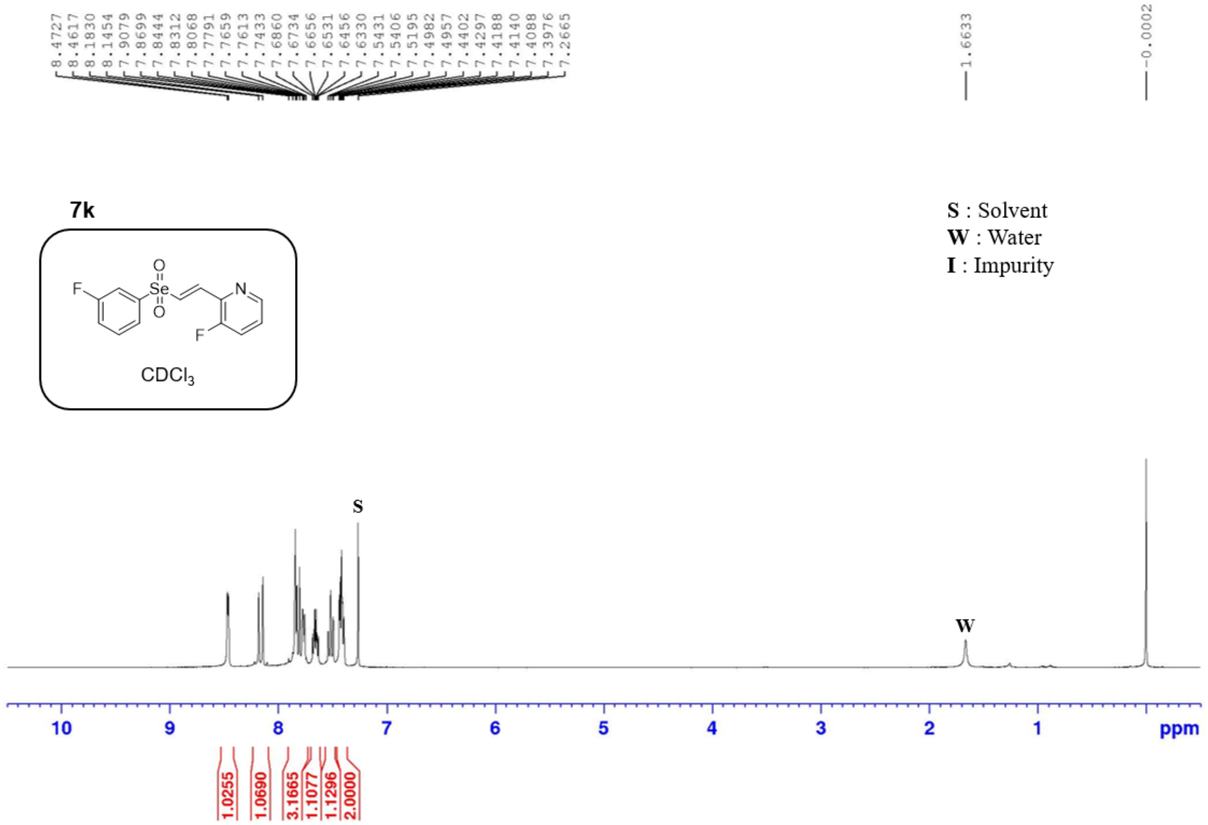
8.4727
8.4617
8.1830
8.1454
7.9079
7.8699
7.8444
7.8312
7.8068
7.7791
7.7659
7.7613
7.7433
7.6860
7.6734
7.6656
7.6531
7.6456
7.6330
7.5431
7.5406
7.5195
7.4982
7.4957
7.4402
7.4297
7.4188
7.4140
7.4088
7.3976
7.2665
1.6633
-0.0002
7k
F
Se
O
O
N
F
CDCl$_3$
S : Solvent
W : Water
I : Impurity
S
W
10
9
8
7
6
5
4
3
2
1
ppm
1.0255
1.0690
3.1665
1.1077
1.1296
2.0000

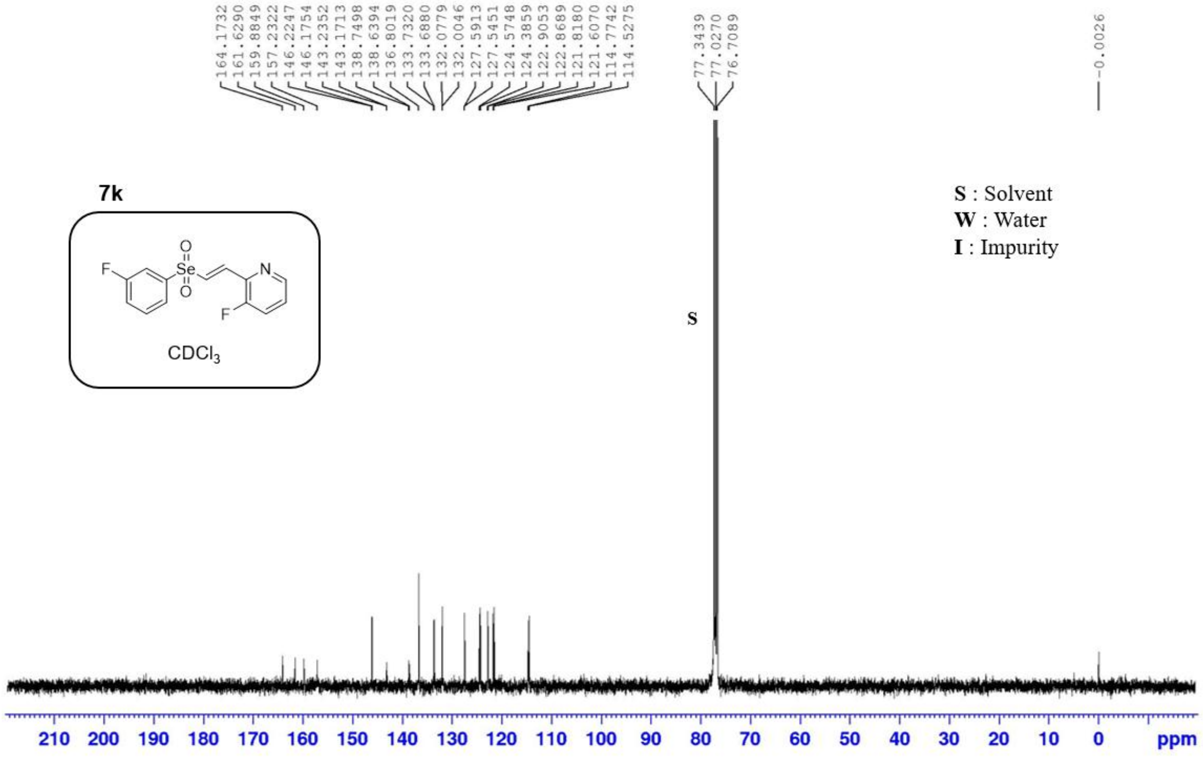
164.1732
161.6290
159.8849
157.2322
146.2247
146.1754
143.2352
143.1713
138.7498
138.6394
136.8019
133.7320
133.6880
132.0779
132.0046
127.5913
127.5451
124.5748
124.3859
122.9053
122.8689
121.8180
121.6070
114.7742
114.5275
77.3439
77.0270
76.7089
-0.0026
7k
F
Se
O
O
N
F
CDCl$_3$
S : Solvent
W : Water
I : Impurity
S
210
200
190
180
170
160
150
140
130
120
110
100
90
80
70
60
50
40
30
20
10
0
ppm

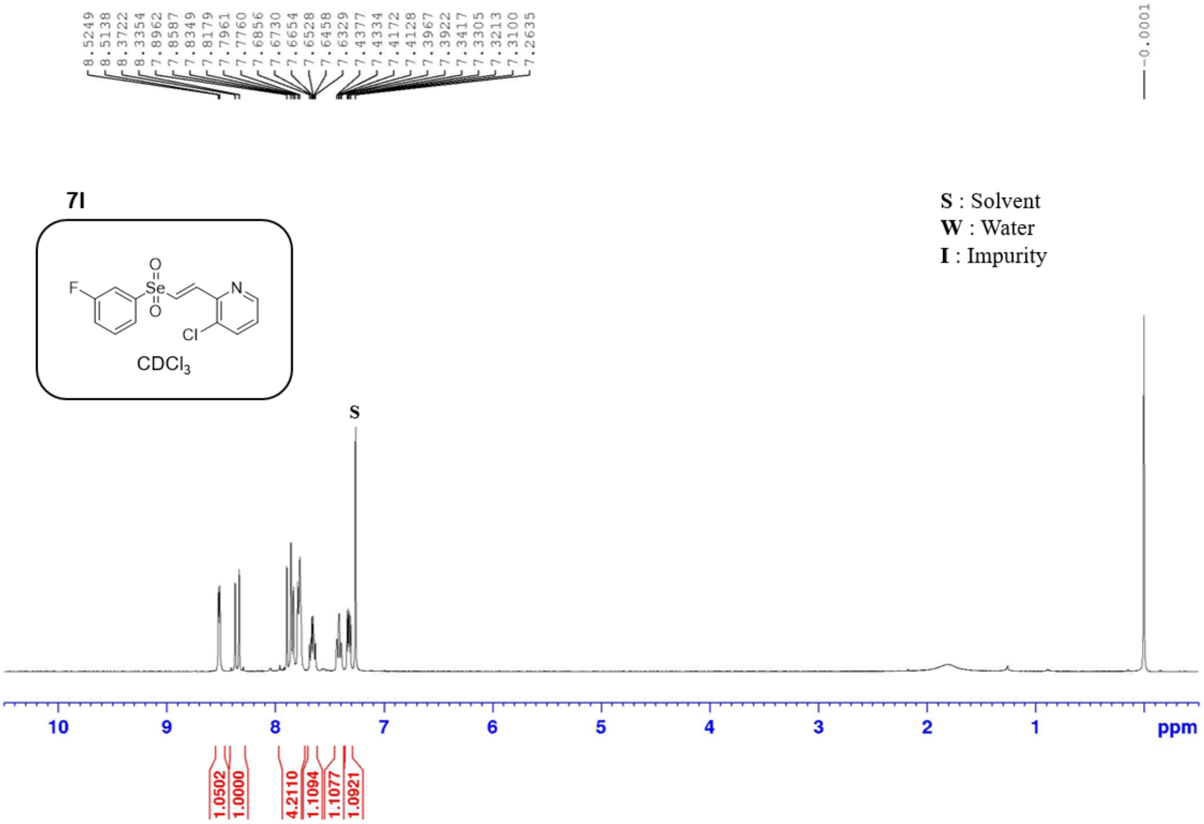
8.5249
8.5138
8.3722
8.3354
7.8962
7.8587
7.8349
7.8179
7.7961
7.7760
7.6856
7.6730
7.6654
7.6528
7.6458
7.6329
7.4377
7.4334
7.4172
7.4128
7.3967
7.3922
7.3417
7.3305
7.3213
7.3100
7.2635
-0.0001
7l
CDCl3
S : Solvent
W : Water
I : Impurity
S
1.0502
1.0000
4.2110
1.1094
1.1077
1.0921
10
9
8
7
6
5
4
3
2
1
ppm

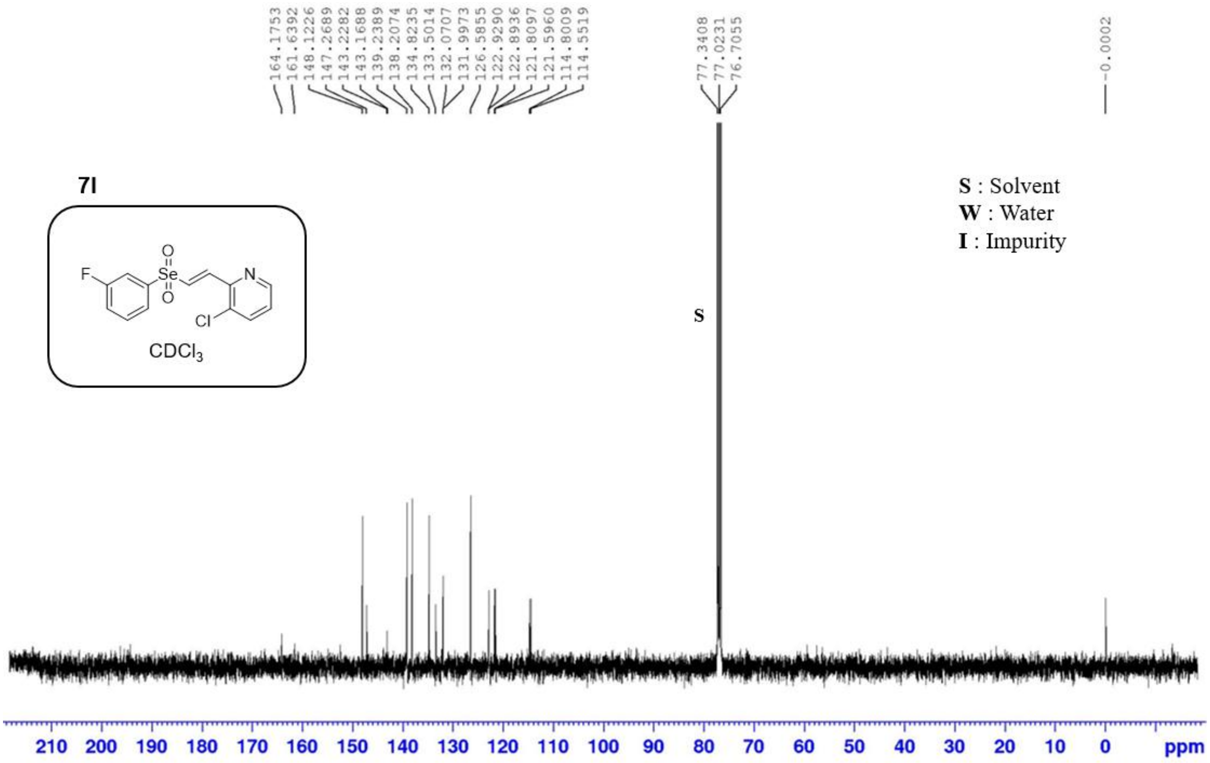
164.1753
161.6392
148.1226
147.2689
143.2282
143.1688
139.2389
138.2074
134.8235
133.5014
132.0707
131.9973
126.5855
122.9290
122.8936
121.8097
121.5960
114.8009
114.5519
77.3408
77.0231
76.7055
-0.0002
7l
CDCl3
S : Solvent
W : Water
I : Impurity
S
210
200
190
180
170
160
150
140
130
120
110
100
90
80
70
60
50
40
30
20
10
0
ppm

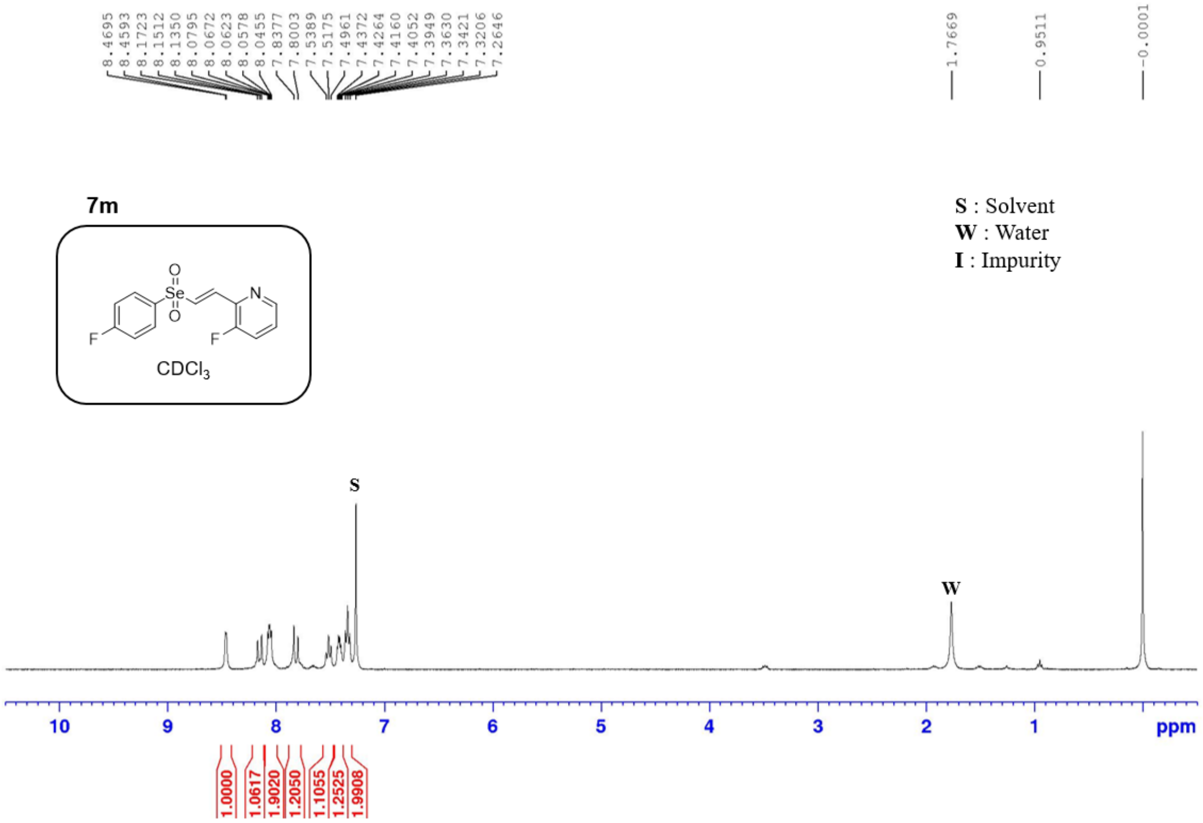
8.4695 8.4593 8.1723 8.1512 8.1350 8.0795 8.0672 8.0623 8.0578 8.0455 7.8377 7.8003 7.5389 7.5175 7.4961 7.4372 7.4264 7.4160 7.4052 7.3949 7.3630 7.3421 7.3206 7.2646
1.7669
0.9511
-0.0001
7m
CDCl3
S : Solvent
W : Water
I : Impurity
S
W
1.0000 1.0617 1.9020 1.2050 1.1055 1.2525 1.9908
ppm

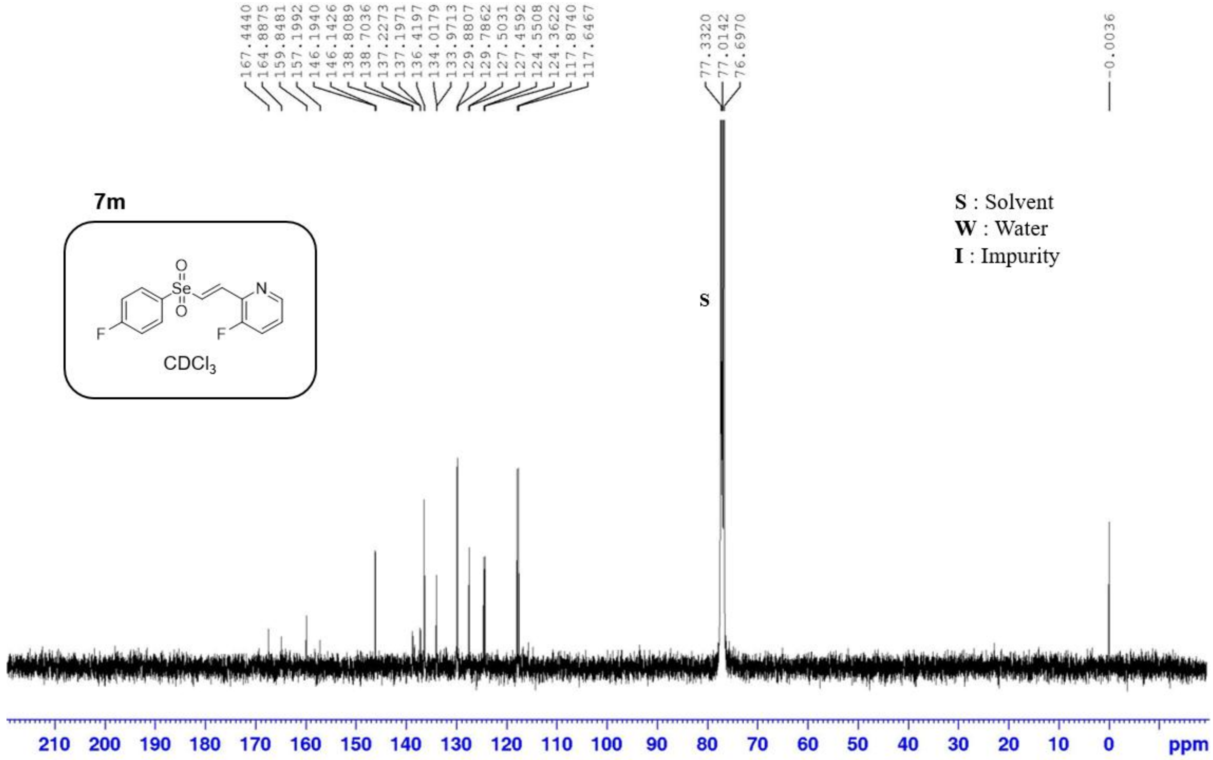
167.4440 164.8875 159.8481 157.1992 146.1940 146.1426 138.8089 138.7036 137.2273 137.1971 136.4197 134.0179 133.9713 129.8807 129.7862 127.5031 127.4592 124.5508 124.3622 117.8740 117.6467
77.3320 77.0142 76.6970
-0.0036
7m
CDCl3
S : Solvent
W : Water
I : Impurity
S
ppm

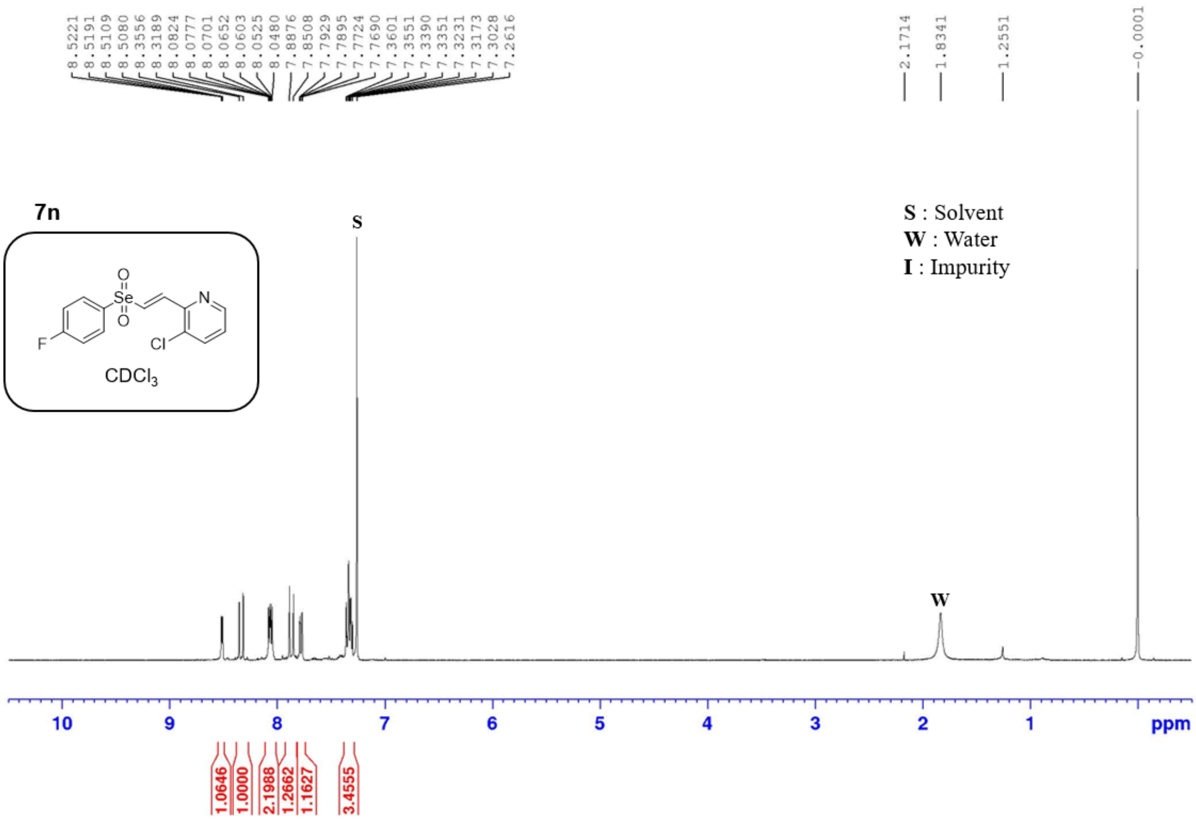
8.5221
8.5191
8.5109
8.5080
8.3556
8.3189
8.0824
8.0777
8.0701
8.0652
8.0603
8.0525
8.0480
7.8876
7.8508
7.7929
7.7895
7.7724
7.7690
7.3601
7.3551
7.3390
7.3351
7.3231
7.3173
7.3028
7.2616
2.1714
1.8341
1.2551
-0.0001
7n
CDCl3
S
S : Solvent
W : Water
I : Impurity
W
10
9
8
7
6
5
4
3
2
1
ppm
1.0646
1.0000
2.1988
1.2662
1.1627
3.4555

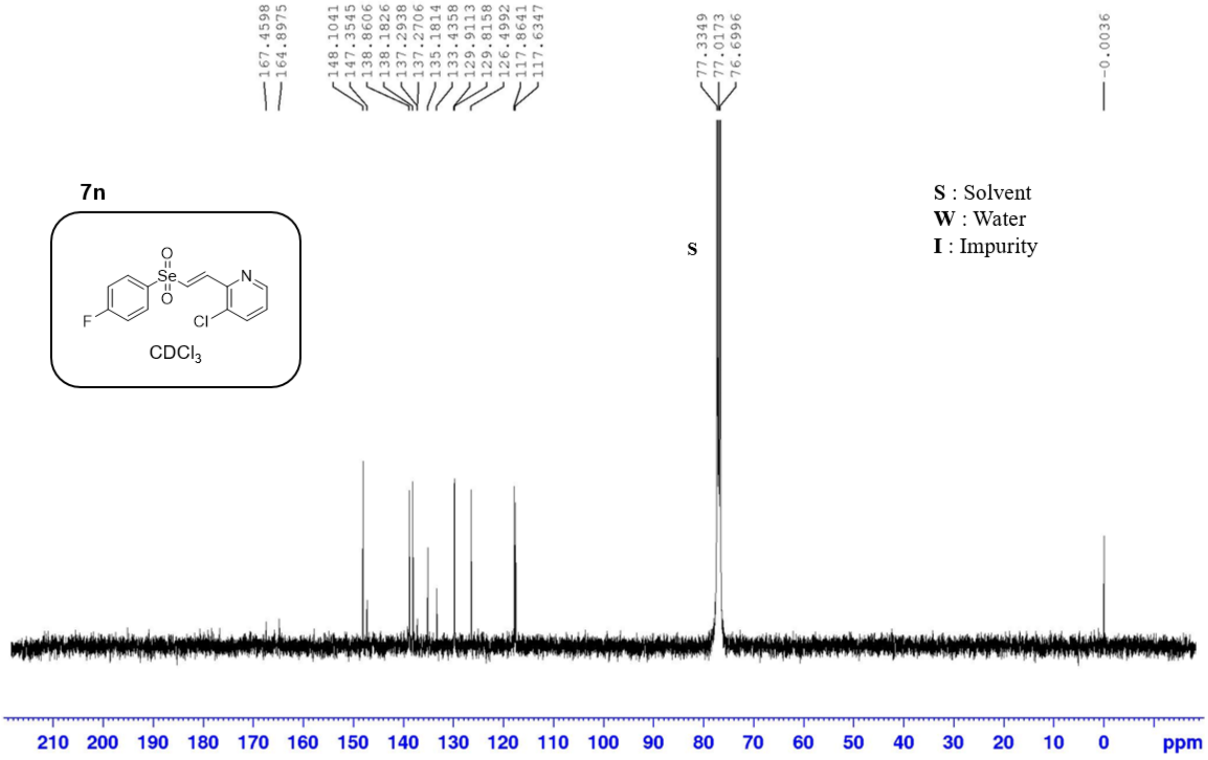
167.4598
164.8975
148.1041
147.3545
138.8606
138.1826
137.2938
137.2706
135.1814
133.4358
129.9113
129.8158
126.4992
117.8641
117.6347
77.3349
77.0173
76.6996
-0.0036
7n
CDCl3
S
S : Solvent
W : Water
I : Impurity
210
200
190
180
170
160
150
140
130
120
110
100
90
80
70
60
50
40
30
20
10
0
ppm

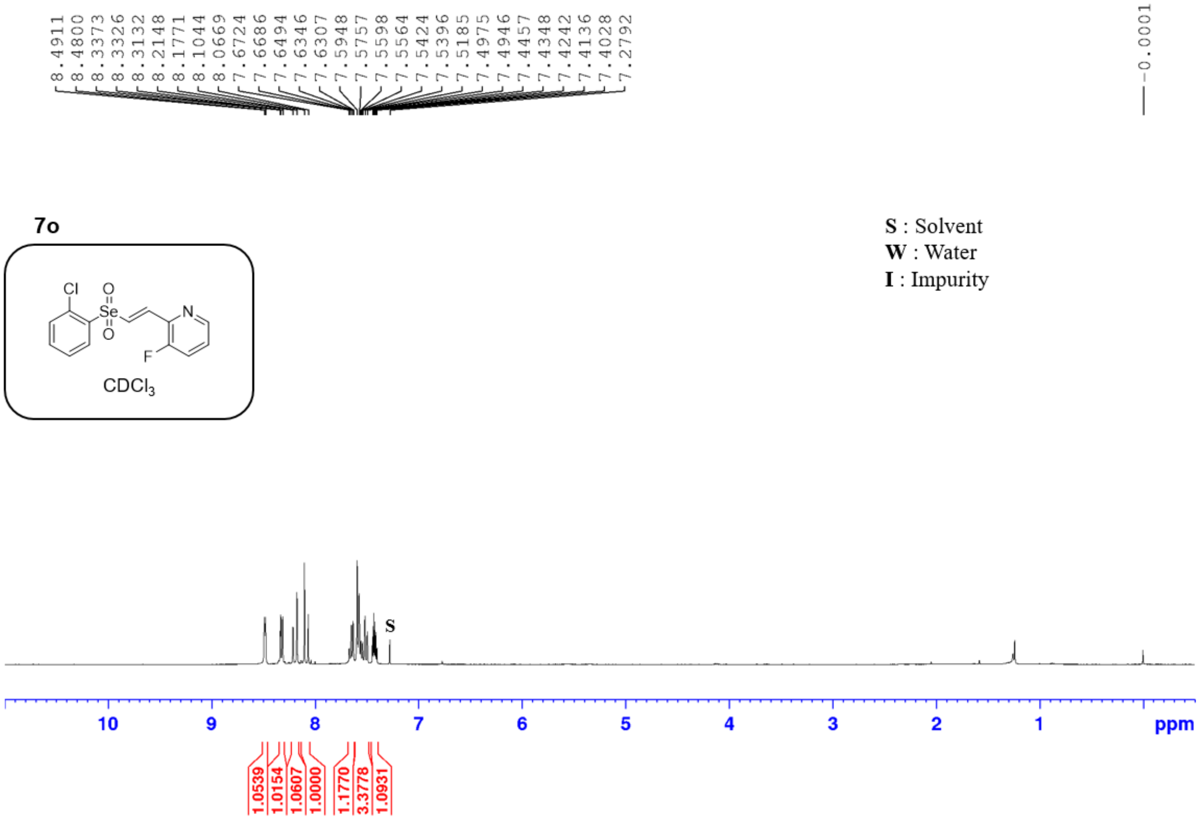
8.4911 8.4800 8.3373 8.3326 8.3132 8.2148 8.1771 8.1044 8.0669 7.6724 7.6686 7.6494 7.6346 7.6307 7.5948 7.5757 7.5598 7.5564 7.5424 7.5396 7.5185 7.4975 7.4946 7.4457 7.4348 7.4242 7.4136 7.4028 7.2792
-0.0001
7o
Cl
O
Se
O
N
F
CDCl3
S : Solvent
W : Water
I : Impurity
S
10 9 8 7 6 5 4 3 2 1 ppm
1.0539 1.0154 1.0607 1.0000 1.1770 3.3778 1.0931

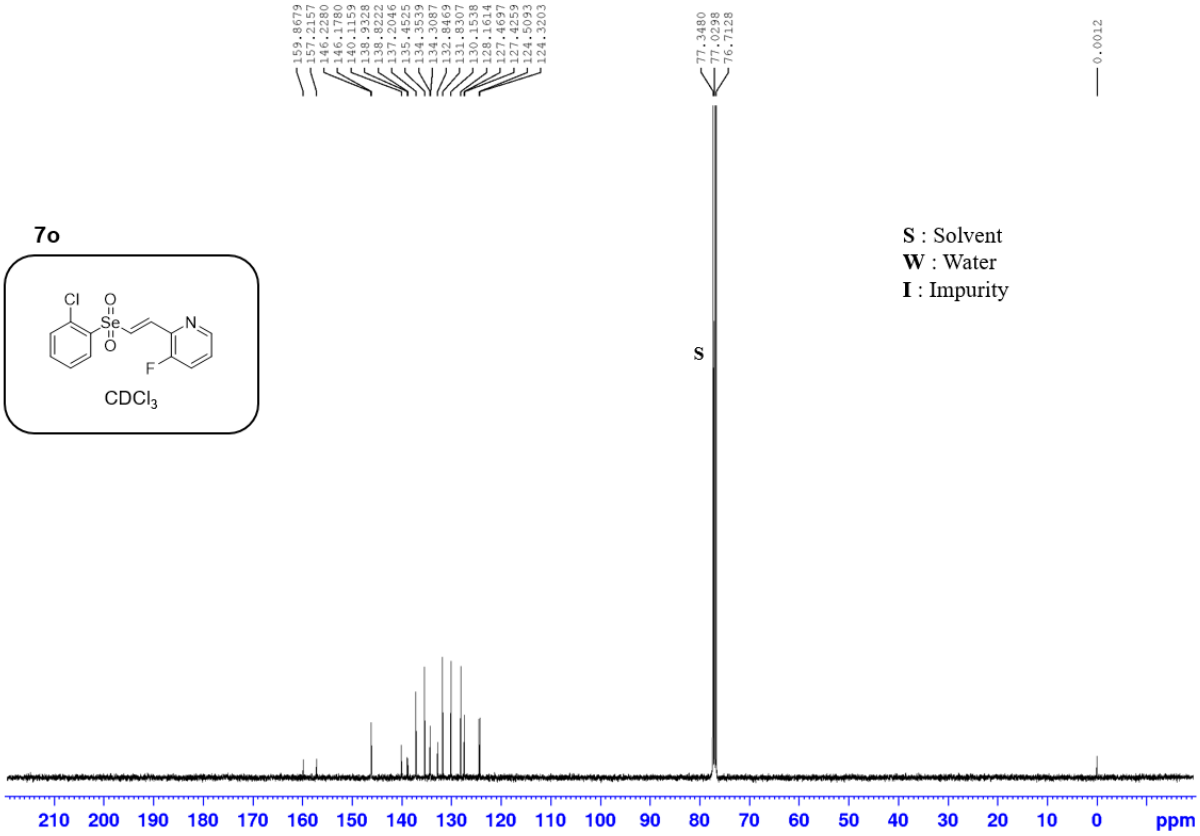
159.8679 157.2157 146.2280 146.1780 140.1159 138.9328 138.8222 137.2046 135.4525 134.3539 134.3087 132.8469 131.8307 130.1538 128.1614 127.4697 127.4259 124.5093 124.3203
77.3480 77.0298 76.7128
0.0012
7o
Cl
O
Se
O
N
F
CDCl3
S : Solvent
W : Water
I : Impurity
S
210 200 190 180 170 160 150 140 130 120 110 100 90 80 70 60 50 40 30 20 10 0 ppm

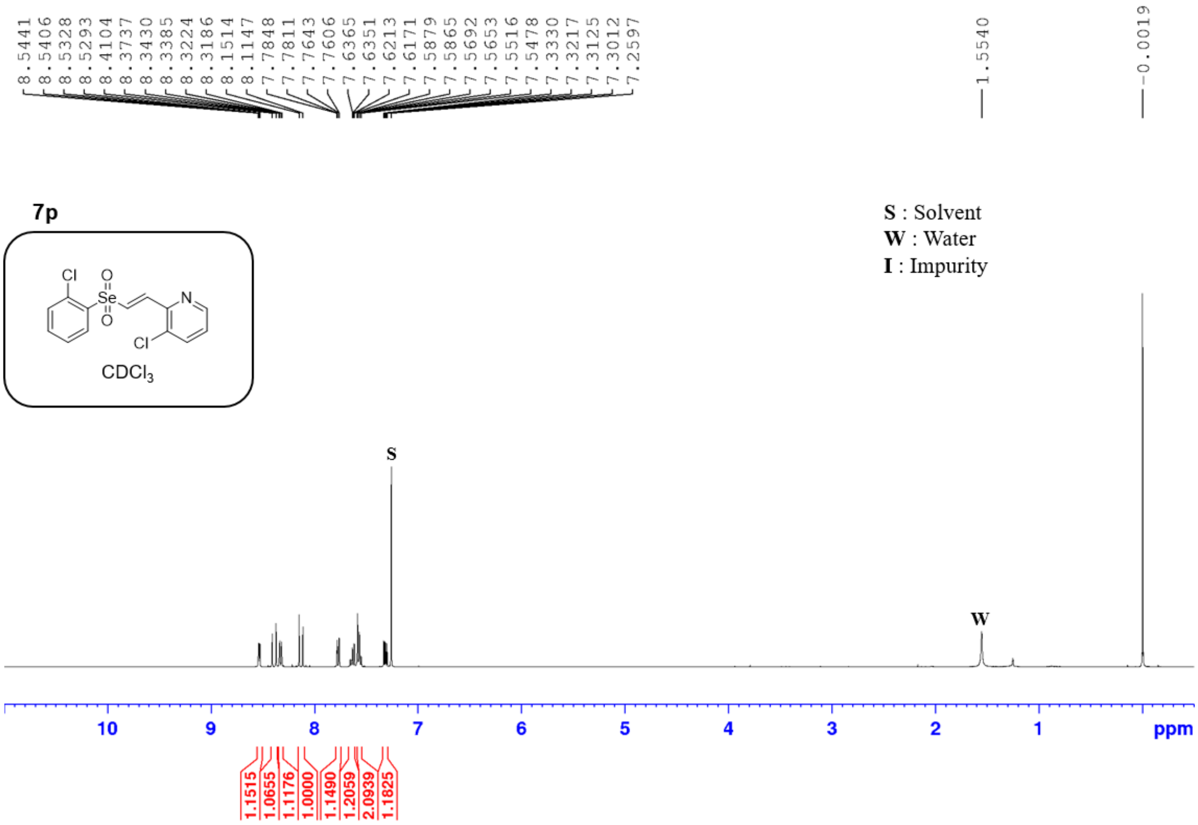
8.5441
8.5406
8.5328
8.5293
8.4104
8.3737
8.3430
8.3385
8.3224
8.3186
8.1514
8.1147
7.7848
7.7811
7.7643
7.7606
7.6365
7.6351
7.6213
7.6171
7.5879
7.5865
7.5692
7.5653
7.5516
7.5478
7.3330
7.3217
7.3125
7.3012
7.2597
1.5540
-0.0019
7p
CDCl3
S : Solvent
W : Water
I : Impurity
S
W
10
9
8
7
6
5
4
3
2
1
ppm
1.1515
1.0655
1.1176
1.0000
1.1490
1.2059
2.0939
1.1825

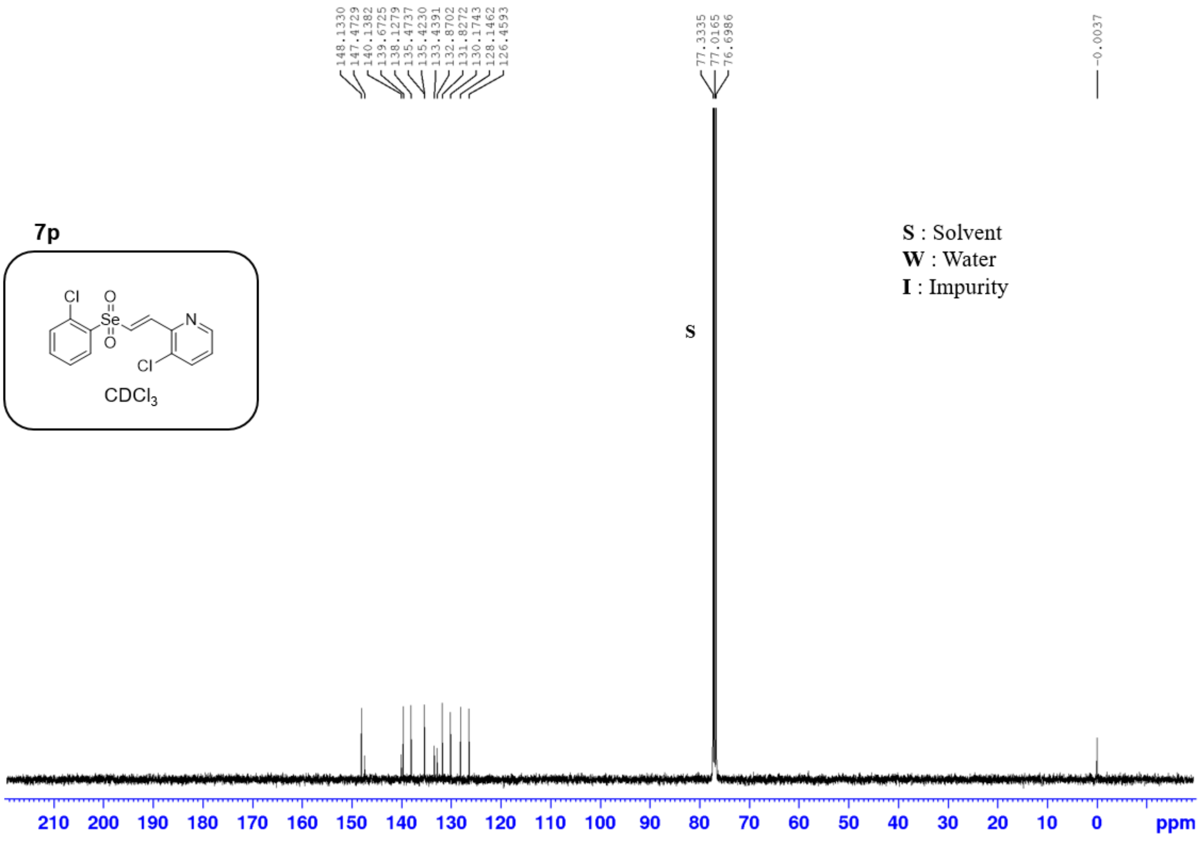
148.1330
147.4729
140.1382
139.6725
138.1279
135.4737
135.4230
133.4391
132.8702
131.8272
130.1743
128.1462
126.4593
77.3335
77.0165
76.6986
-0.0037
7p
CDCl3
S : Solvent
W : Water
I : Impurity
S
210
200
190
180
170
160
150
140
130
120
110
100
90
80
70
60
50
40
30
20
10
0
ppm

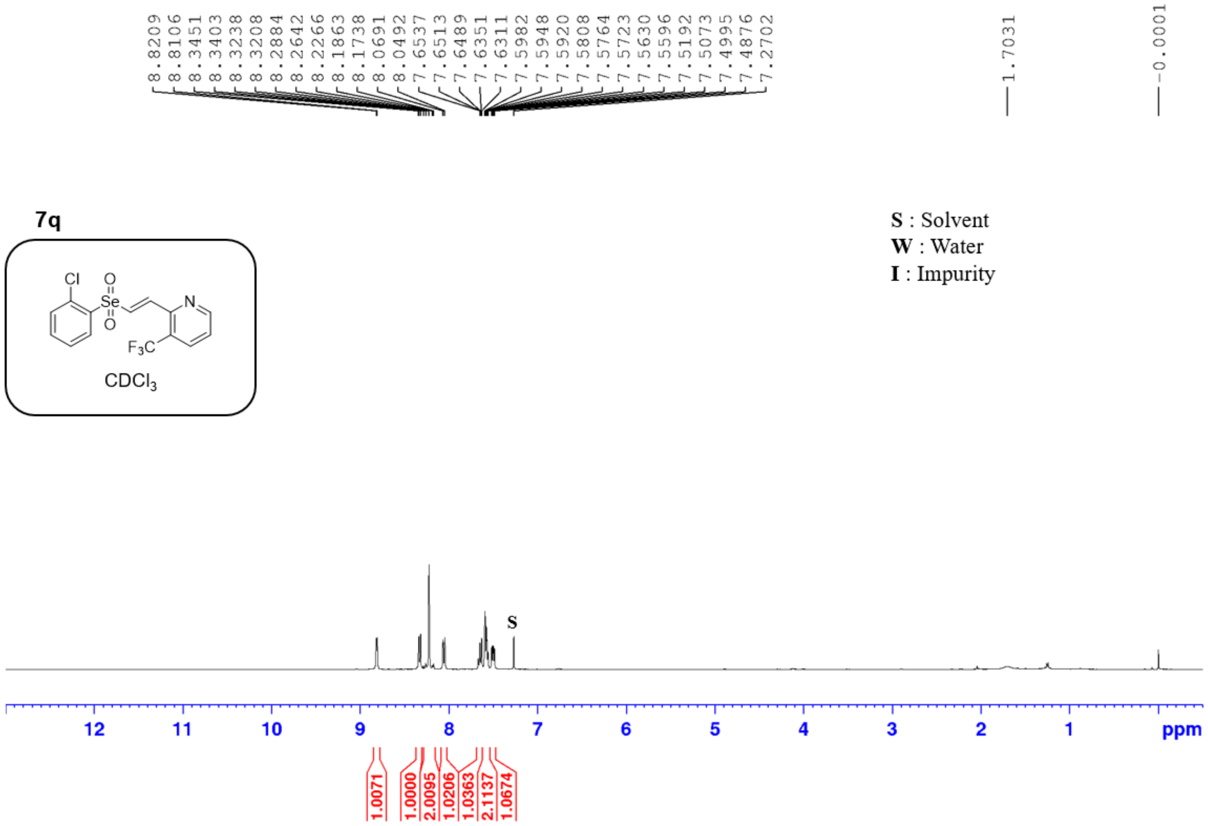
8.8209
8.8106
8.3451
8.3403
8.3238
8.3208
8.2884
8.2642
8.2266
8.1863
8.1738
8.0691
8.0492
7.6537
7.6513
7.6489
7.6351
7.6311
7.5982
7.5948
7.5920
7.5808
7.5764
7.5723
7.5630
7.5596
7.5192
7.5073
7.4995
7.4876
7.2702
1.7031
-0.0001
7q
Cl
O
Se
O
N
F3C
CDCl3
S : Solvent
W : Water
I : Impurity
S
12
11
10
9
8
7
6
5
4
3
2
1
ppm
1.0071
1.0000
2.0095
1.0206
1.0363
2.1137
1.0674

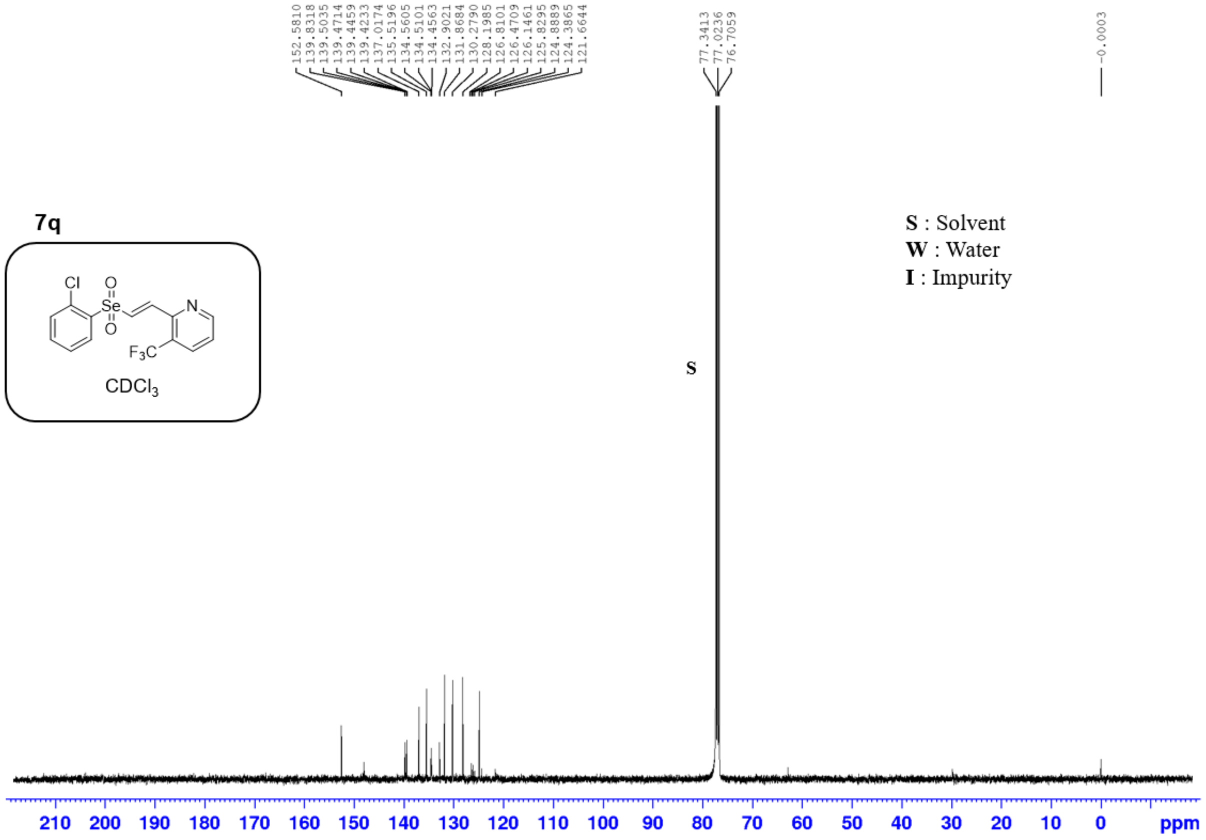
152.5810
139.8318
139.5035
139.4714
139.4459
139.4233
137.0174
135.5196
134.5605
134.5101
134.4563
132.9021
131.8684
130.2790
128.1985
126.8101
126.4709
126.1461
125.8295
124.8889
124.3865
121.6644
77.3413
77.0236
76.7059
-0.0003
7q
Cl
O
Se
O
N
F3C
CDCl3
S : Solvent
W : Water
I : Impurity
S
210
200
190
180
170
160
150
140
130
120
110
100
90
80
70
60
50
40
30
20
10
0
ppm

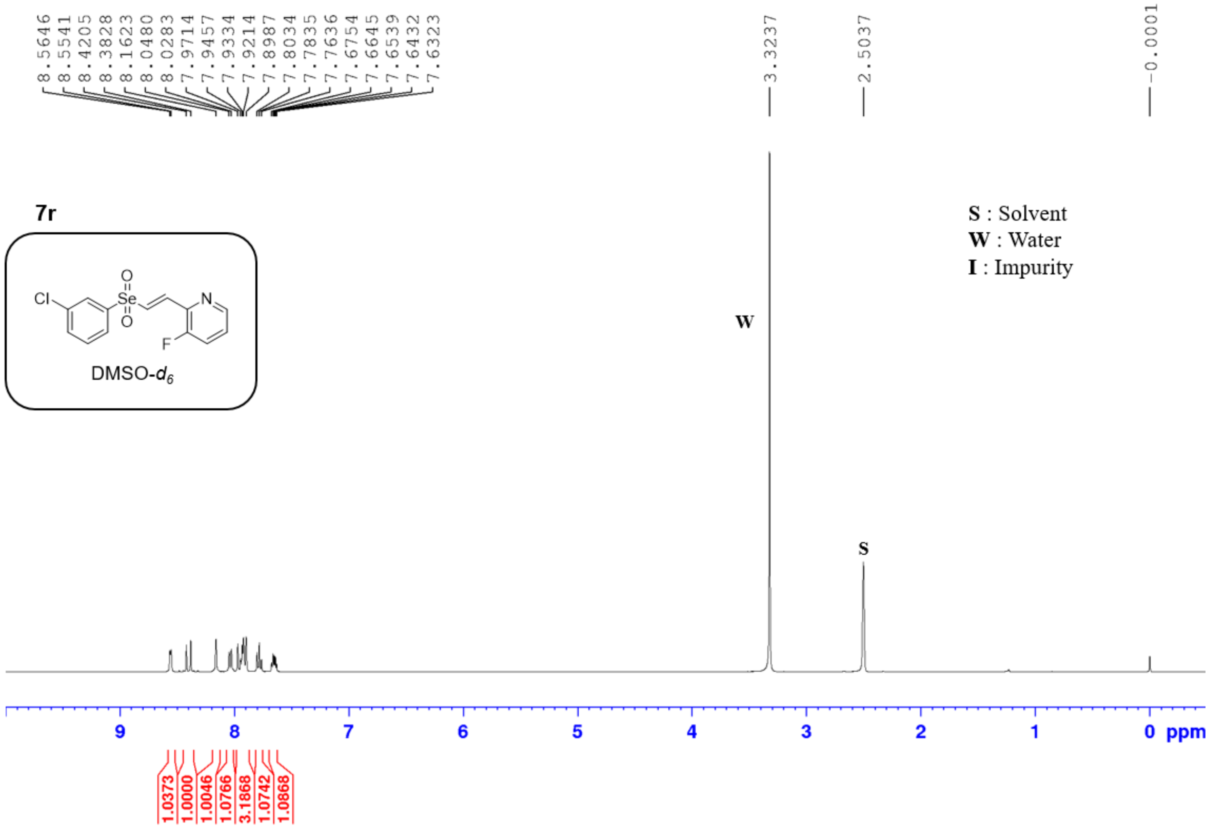
8.5646
8.5541
8.4205
8.3828
8.1623
8.0480
8.0283
7.9714
7.9457
7.9334
7.9214
7.8987
7.8034
7.7835
7.7636
7.6754
7.6645
7.6539
7.6432
7.6323
3.3237
2.5037
-0.0001
7r
Cl
Se
O
O
N
F
DMSO-d6
W
S
S : Solvent
W : Water
I : Impurity
9
8
7
6
5
4
3
2
1
0 ppm
1.0373
1.0000
1.0046
1.0766
3.1868
1.0742
1.0868

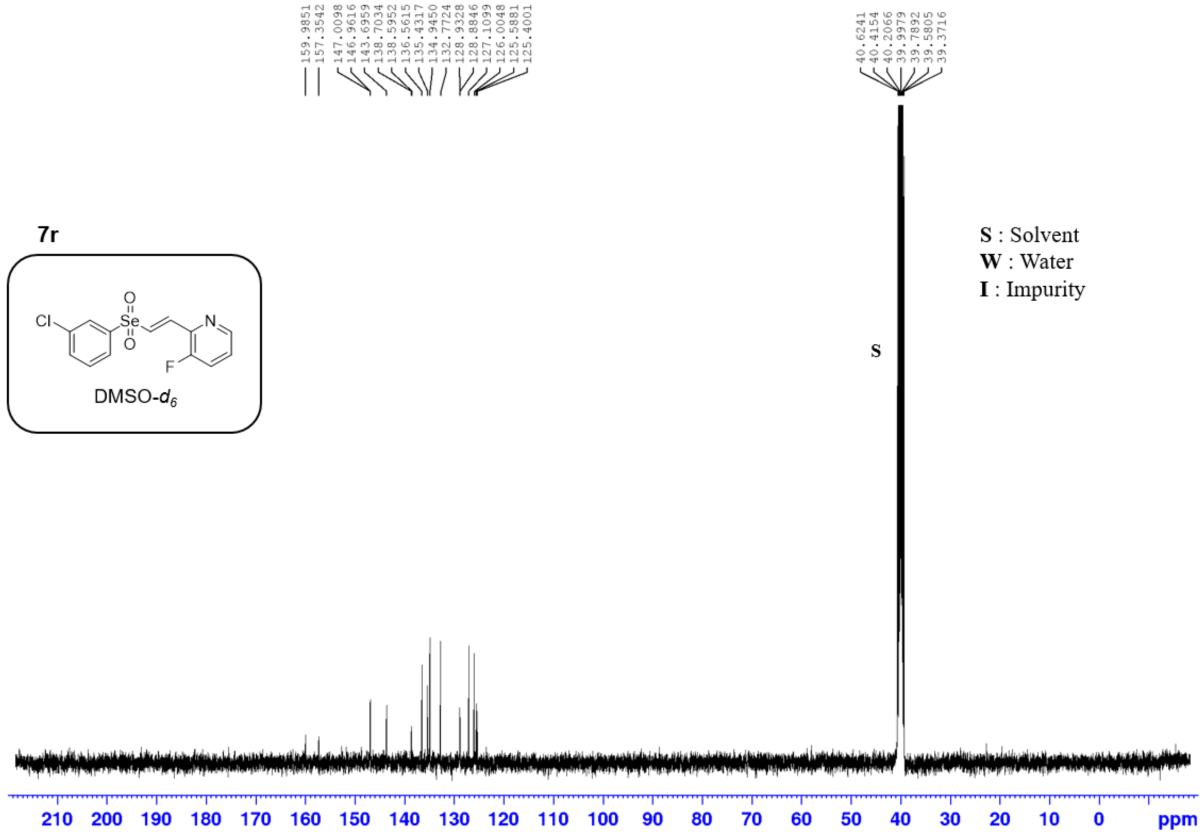
159.9851
157.3542
147.0098
146.9616
143.6959
138.7034
138.5952
136.5615
135.4317
134.9450
132.7724
128.9328
128.8846
127.1099
126.0048
125.5881
125.4001
40.6241
40.4154
40.2066
39.9979
39.7892
39.5805
39.3716
7r
Cl
Se
O
O
N
F
DMSO-d6
S
S : Solvent
W : Water
I : Impurity
210
200
190
180
170
160
150
140
130
120
110
100
90
80
70
60
50
40
30
20
10
0
ppm

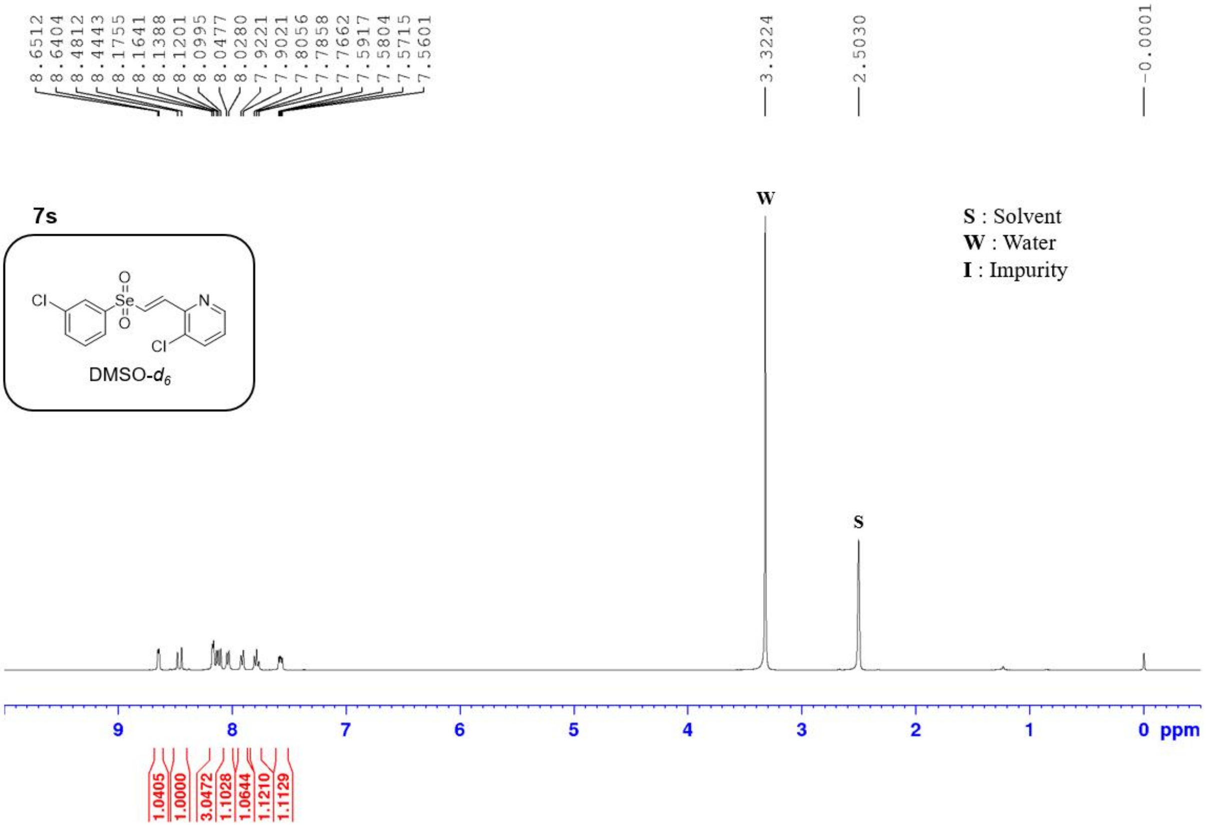
8.6512
8.6404
8.4812
8.4443
8.1755
8.1641
8.1388
8.1201
8.0995
8.0477
8.0280
7.9221
7.9021
7.8056
7.7858
7.7662
7.5917
7.5804
7.5715
7.5601
3.3224
2.5030
-0.0001
7s
DMSO-d_6
W
S
S : Solvent
W : Water
I : Impurity
9
8
7
6
5
4
3
2
1
0 ppm
1.0405
1.0000
3.0472
1.1028
1.0644
1.1210
1.1129

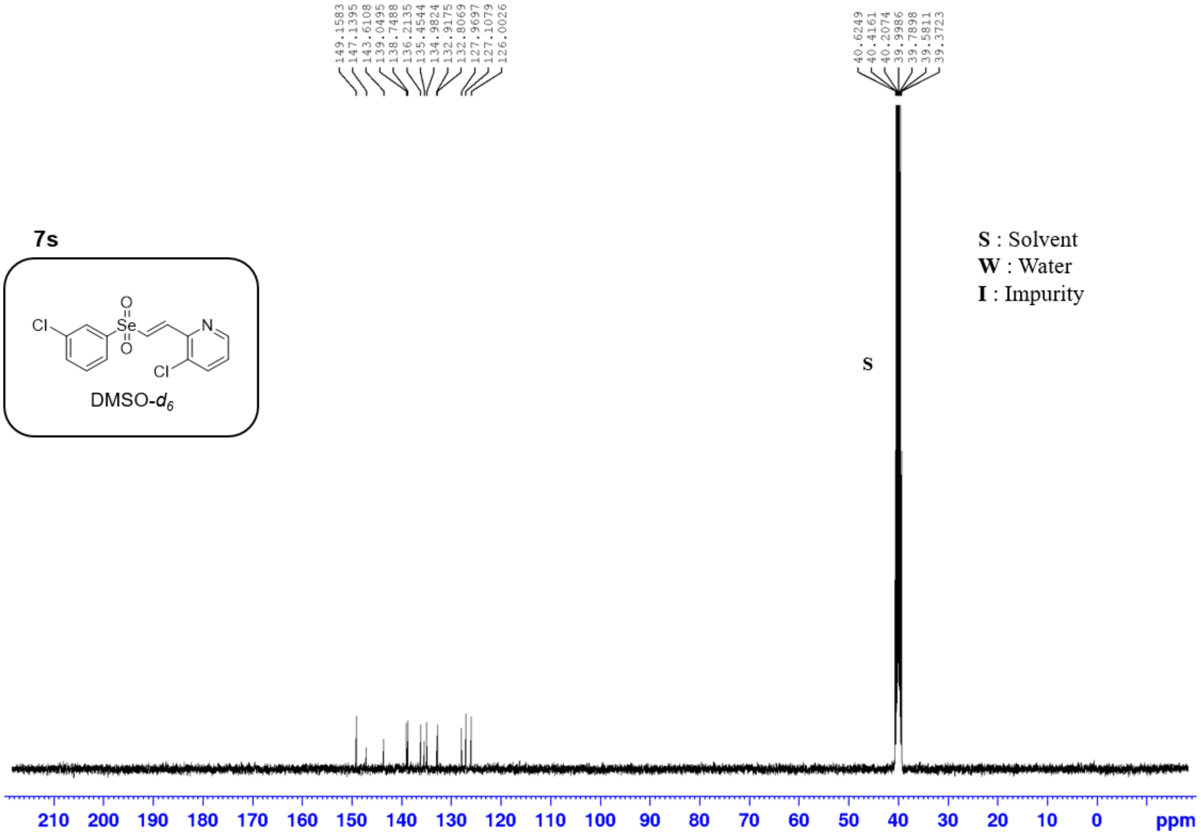
149.1583
147.1395
143.6108
139.0495
138.7488
136.2135
135.4544
134.9824
132.9175
132.8069
127.9697
127.1079
126.0026
40.6249
40.4161
40.2074
39.9986
39.7898
39.5811
39.3723
7s
DMSO-d_6
S : Solvent
W : Water
I : Impurity
S
210
200
190
180
170
160
150
140
130
120
110
100
90
80
70
60
50
40
30
20
10
0
ppm

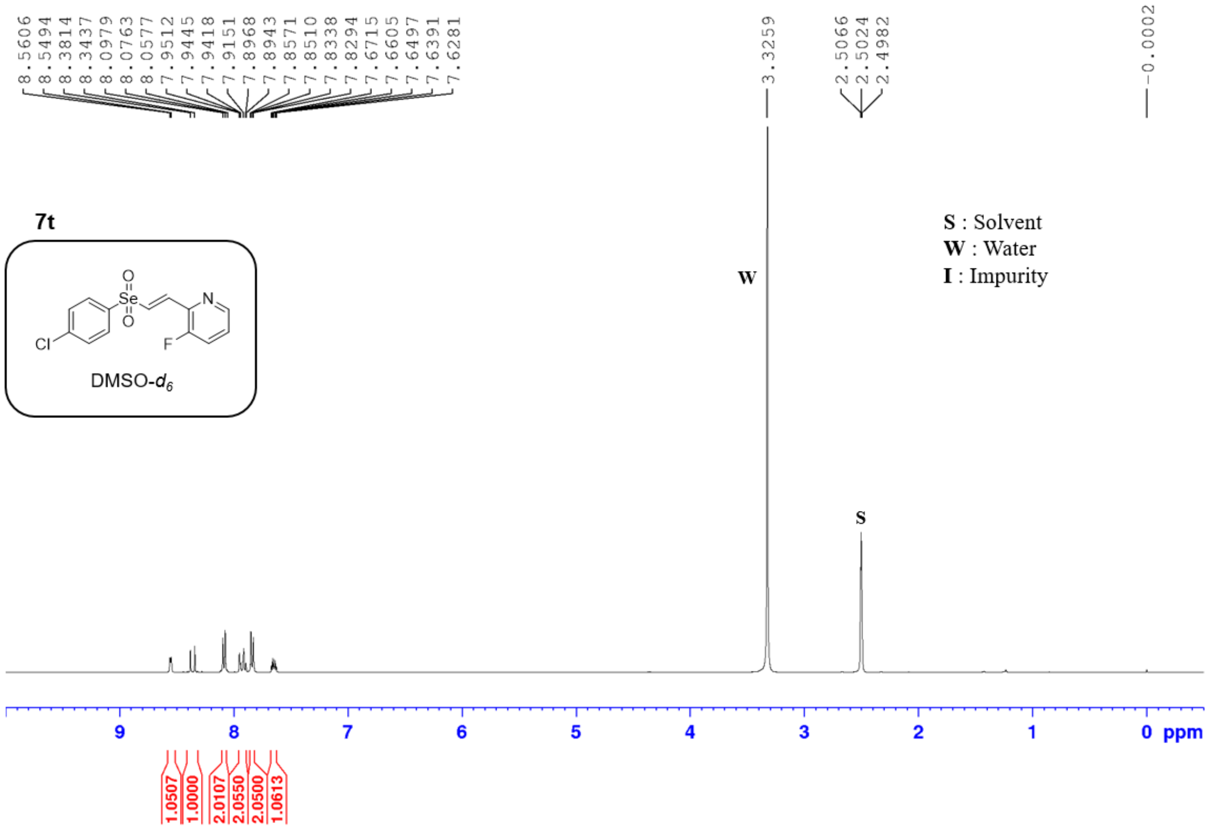

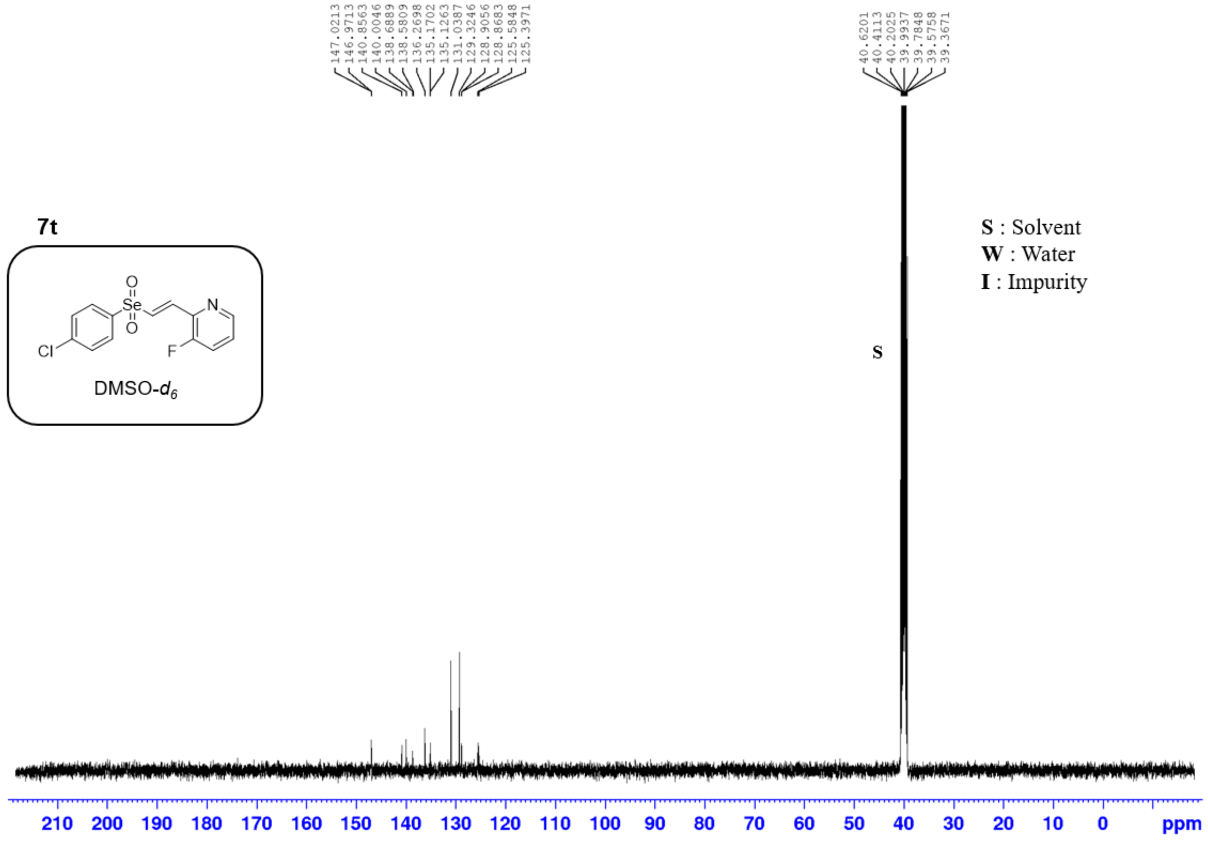

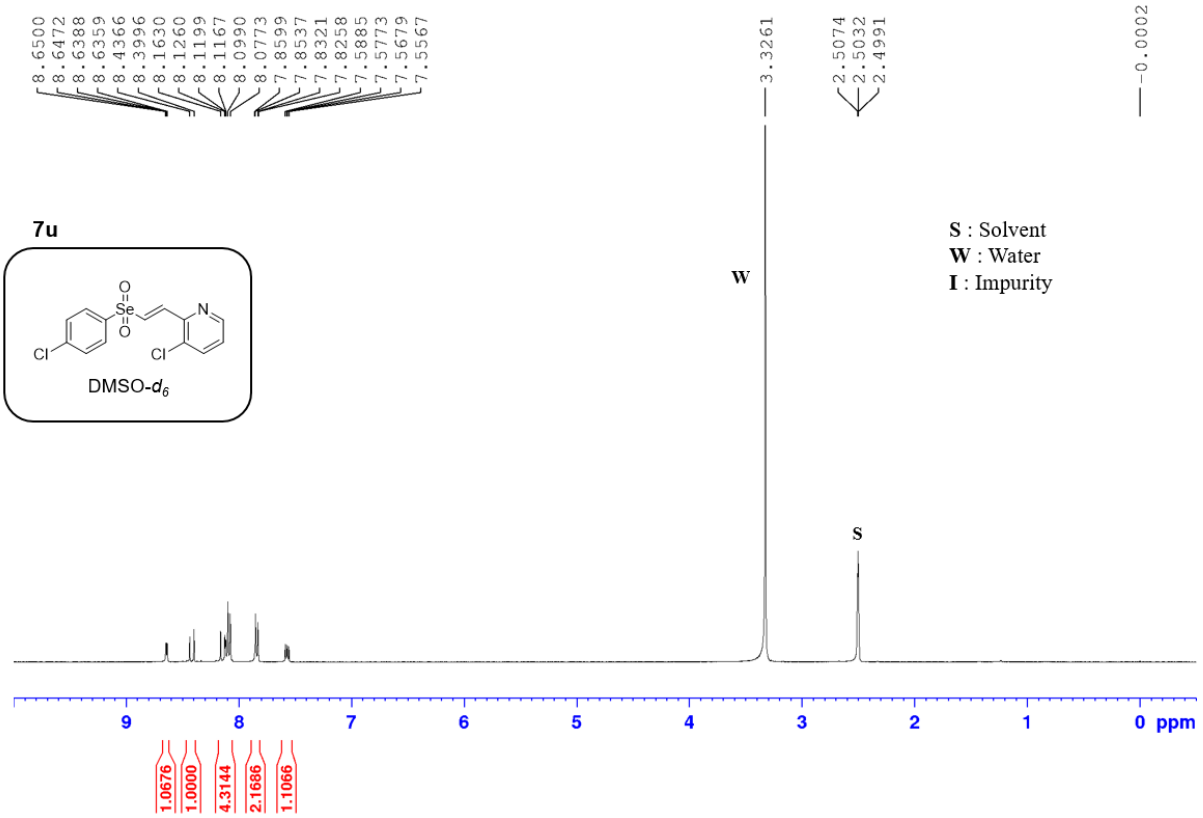
8.6500
8.6472
8.6388
8.6359
8.4366
8.3996
8.1630
8.1260
8.1199
8.1167
8.0990
8.0773
7.8599
7.8537
7.8321
7.8258
7.5885
7.5773
7.5679
7.5567
3.3261
2.5074
2.5032
2.4991
-0.0002
7u
DMSO-d_6
W
S
S : Solvent
W : Water
I : Impurity
1.0676
1.0000
4.3144
2.1686
1.1066
ppm

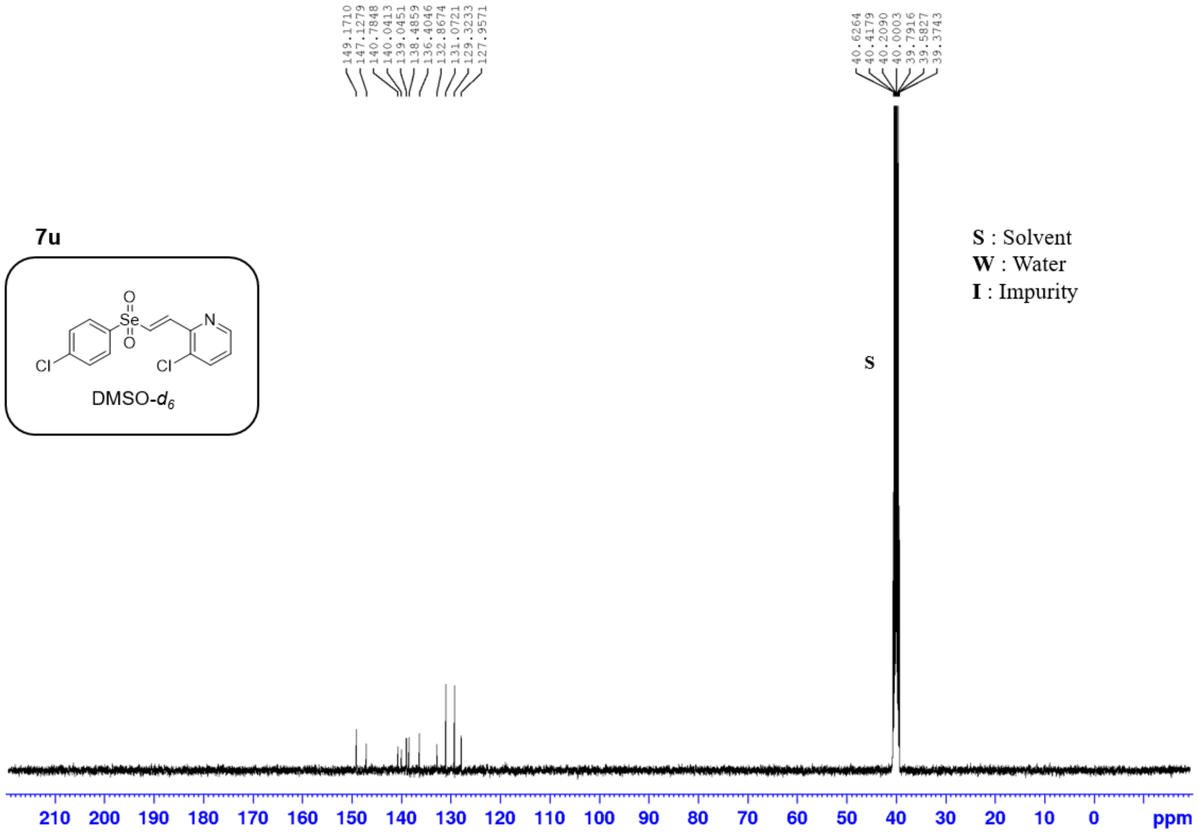
149.1710
147.1279
140.7848
140.0413
139.0451
138.4859
136.4046
132.8674
131.0721
129.3233
127.9571
40.6264
40.4179
40.2090
40.0003
39.7916
39.5827
39.3743
7u
DMSO-d_6
S
S : Solvent
W : Water
I : Impurity
ppm

4. HPLC Trace for the Final Compounds

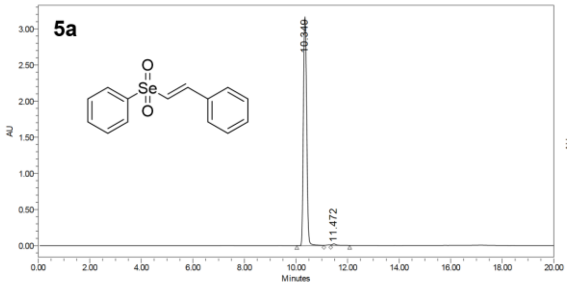

	RT	Area	% Area	Height
1	10.349	28127763	99.08	3169130
2	11.472	260374	0.92	22429

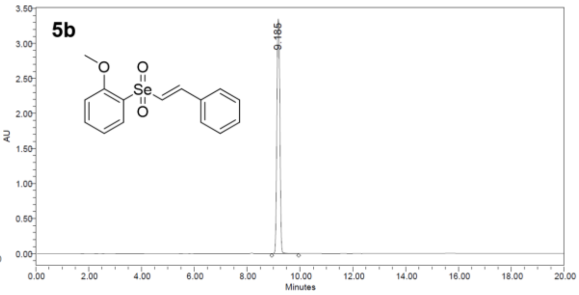

	RT	Area	% Area	Height
1	9.185	26432693	100.00	3355986

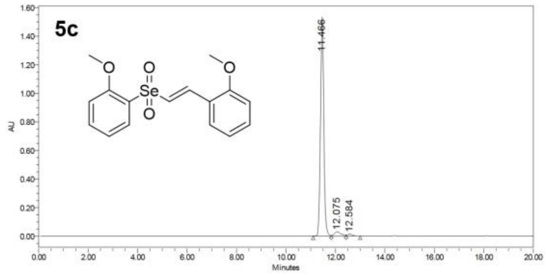

	RT	Area	% Area	Height
1	11.466	14291363	95.13	1530979
2	12.075	549909	3.66	29676
3	12.584	180992	1.20	13925

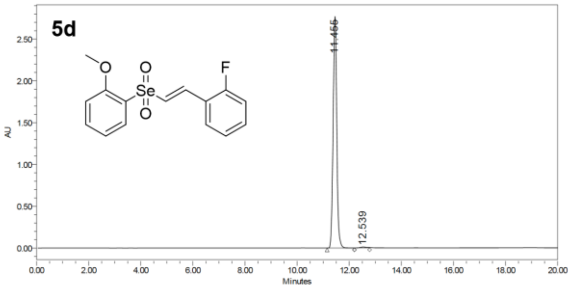

	RT	Area	% Area	Height
1	11.455	26978948	99.32	2775289
2	12.539	183584	0.68	10357

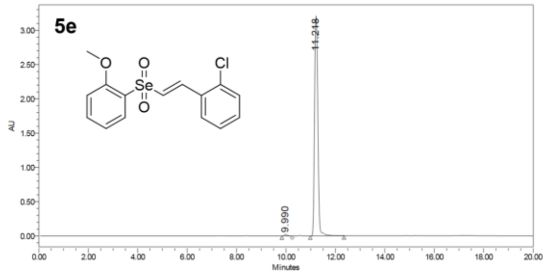

	RT	Area	% Area	Height
1	9.990	166715	0.56	21667
2	11.218	29613231	99.44	3213151

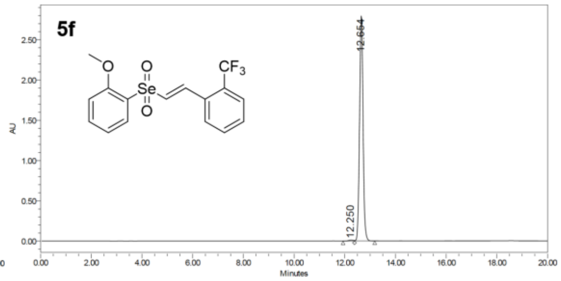

	RT	Area	% Area	Height
1	12.250	142640	0.53	10965
2	12.654	26706145	99.47	2798258

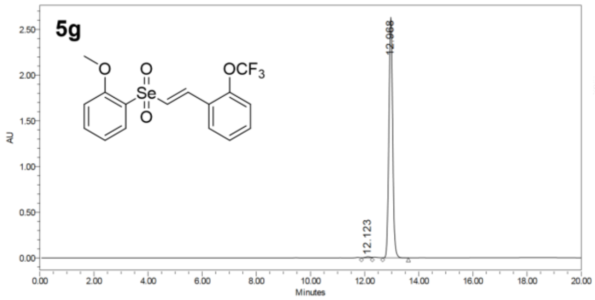

	RT	Area	% Area	Height
1	12.123	162102	0.63	11371
2	12.968	25421120	99.37	2628757

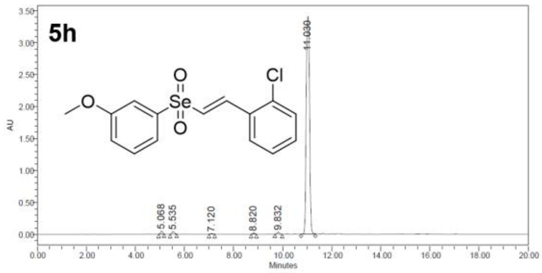

	RT	Area	% Area	Height
1	5.068	257657	0.78	45469
2	5.535	269069	0.81	38312
3	7.120	72777	0.22	12704
4	8.820	133681	0.40	22950
5	9.832	223546	0.67	30335
6	11.030	32220195	97.12	3419662

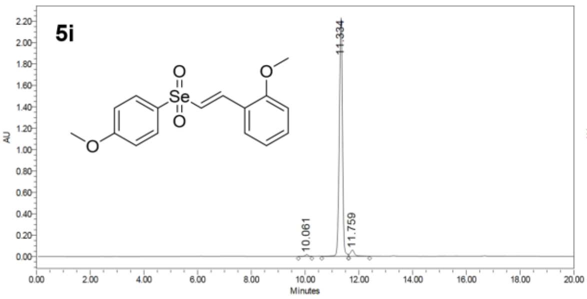

	RT	Area	% Area	Height
1	10.061	144280	0.76	17548
2	11.334	18157143	95.62	2223850
3	11.759	687587	3.62	59979

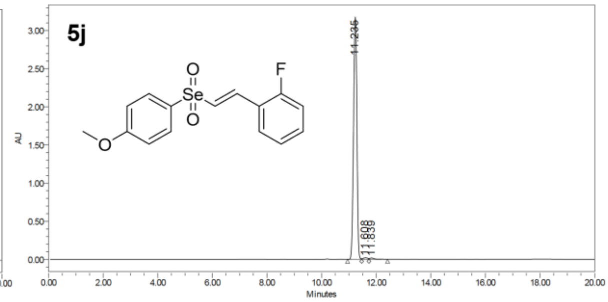

	RT	Area	% Area	Height
1	11.235	27177556	98.34	3179046
2	11.608	244283	0.88	20363
3	11.839	213149	0.77	17580

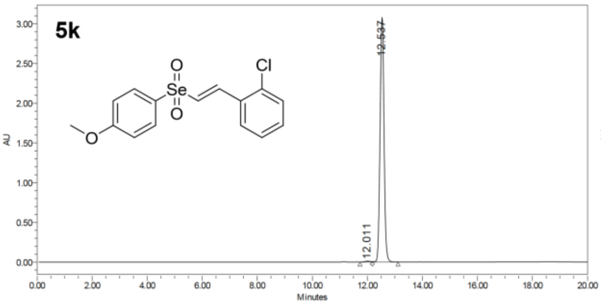

	RT	Area	% Area	Height
1	12.011	162659	0.53	10487
2	12.537	30666020	99.47	3083447

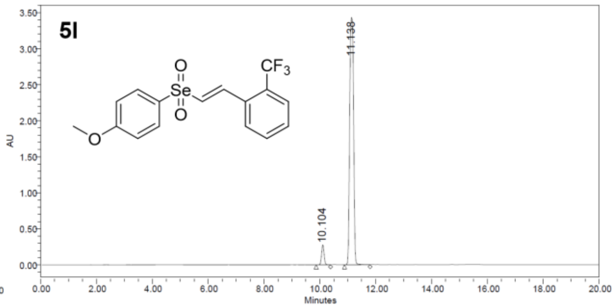

	RT	Area	% Area	Height
1	10.104	1723887	4.96	277541
2	11.138	33058415	95.04	3442210

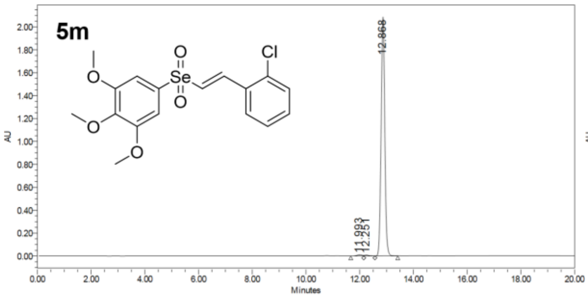

	RT	Area	% Area	Height
1	11.993	131359	0.66	9598
2	12.251	84543	0.42	7191
3	12.868	19695624	98.92	2083061

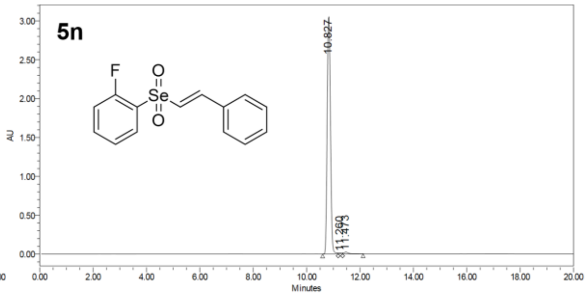

	RT	Area	% Area	Height
1	10.827	26027011	98.38	3065855
2	11.260	122027	0.46	12679
3	11.473	306646	1.16	24690

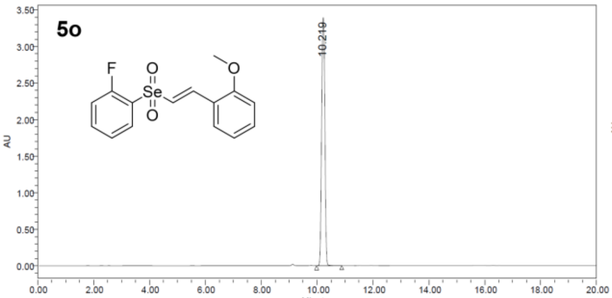

	RT	Area	% Area	Height
1	10.219	27282570	100.00	3406711

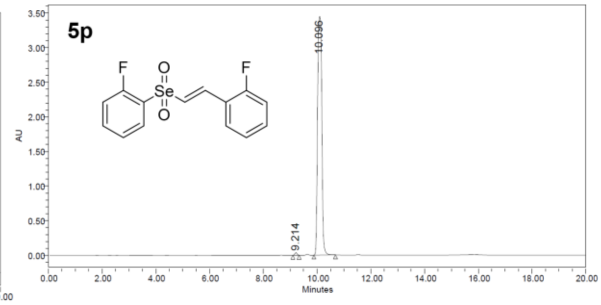

	RT	Area	% Area	Height
1	9.214	213732	0.57	35611
2	10.096	37447569	99.43	3455817

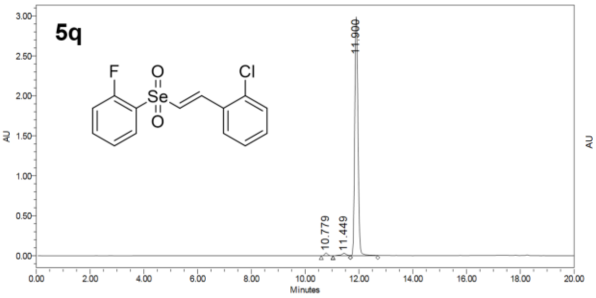

	RT	Area	% Area	Height
1	10.779	259672	1.01	34482
2	11.449	391691	1.52	30462
3	11.900	25123093	97.47	2984639

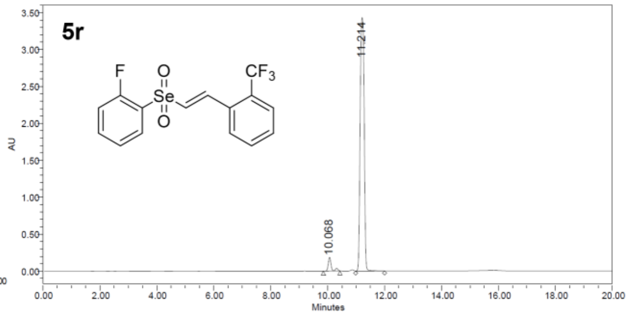

	RT	Area	% Area	Height
1	10.068	1378472	3.94	185111
2	11.214	33596542	96.06	3436660

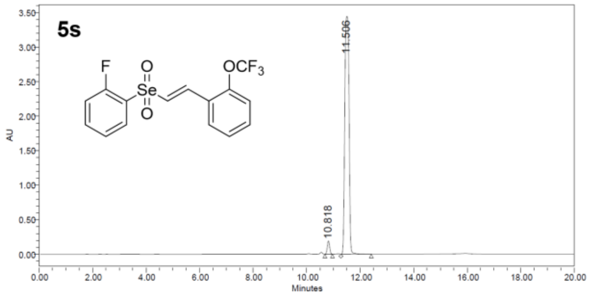

	RT	Area	% Area	Height
1	10.818	1179231	3.02	192141
2	11.506	37838738	96.98	3457816

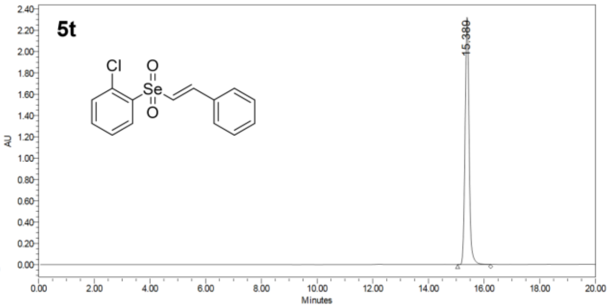

	RT	Area	% Area	Height
1	15.389	23325931	100.00	2328078

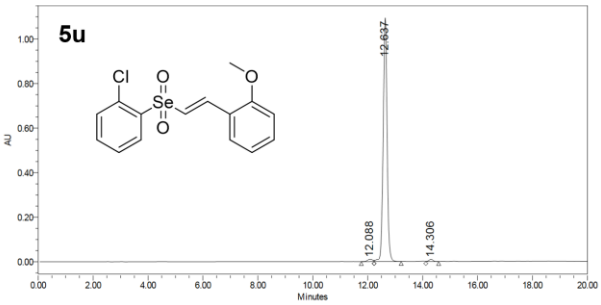

	RT	Area	% Area	Height
1	12.088	141657	1.27	10844
2	12.637	10865668	97.78	1094441
3	14.306	104924	0.94	10411

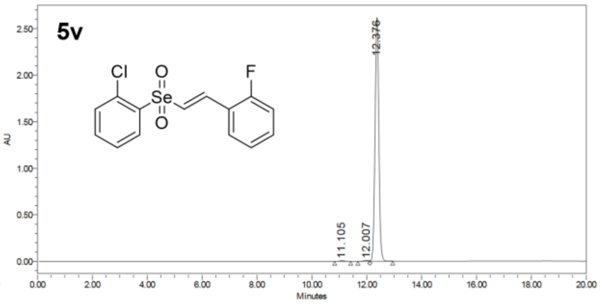

	RT	Area	% Area	Height
1	11.105	75317	0.30	8274
2	12.007	111571	0.44	9191
3	12.376	25034078	99.26	2634414

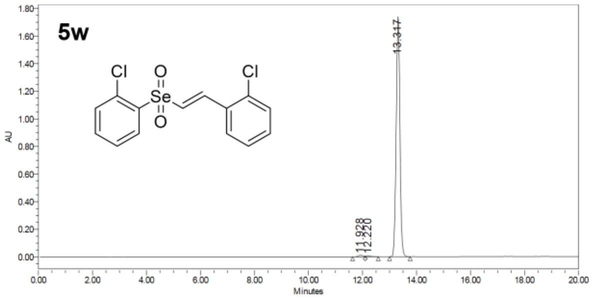

	RT	Area	% Area	Height
1	11.928	147945	0.88	10285
2	12.220	90755	0.54	7726
3	13.317	16545067	98.58	1737985

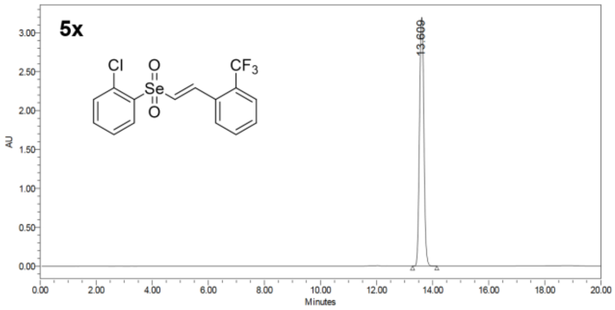

	RT	Area	% Area	Height
1	13.609	34930988	100.00	3212186

5y

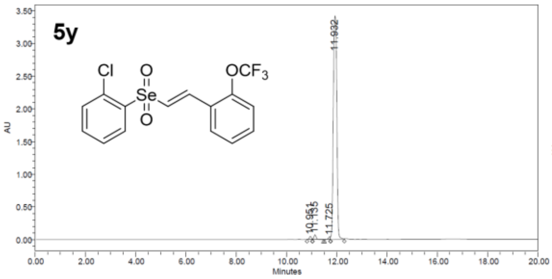

	RT	Area	% Area	Height
1	10.951	284974	0.84	46370
2	11.135	443081	1.31	69878
3	11.725	221993	0.66	43333
4	11.932	32858499	97.19	3430385

5z

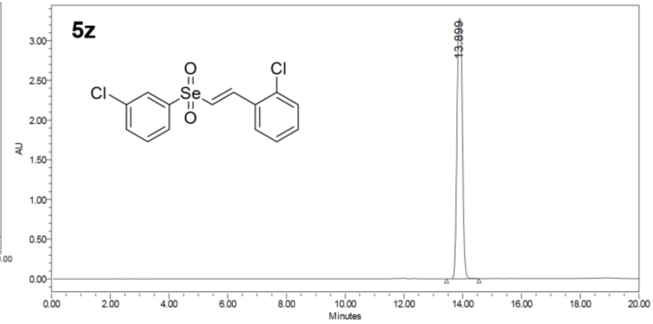

	RT	Area	% Area	Height
1	13.899	41728011	100.00	3283500

5aa

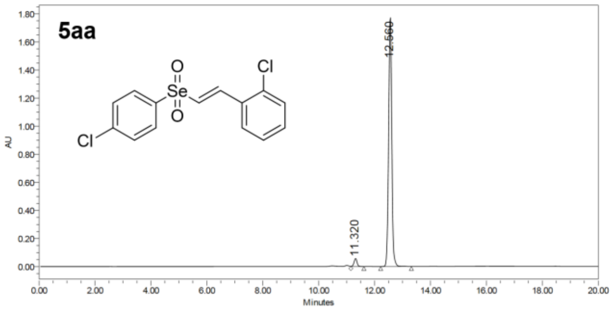

	RT	Area	% Area	Height
1	11.320	424189	2.96	57014
2	12.560	13914763	97.04	1779140

7a

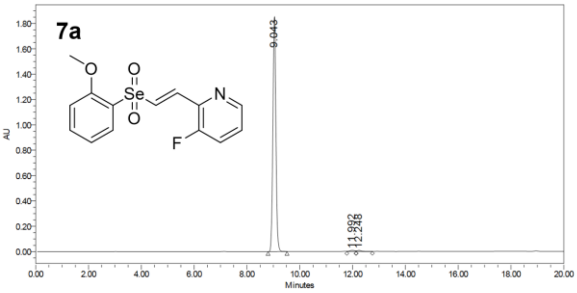

	RT	Area	% Area	Height
1	9.043	14581148	98.44	1864686
2	11.992	121785	0.82	9051
3	12.248	109870	0.74	8684

7b

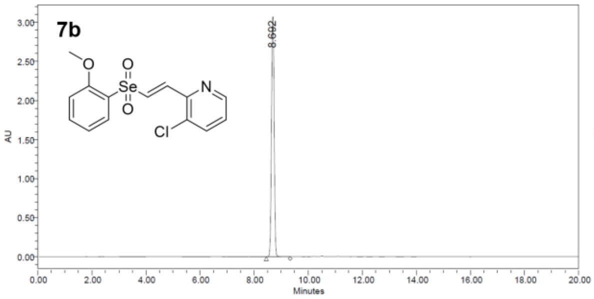

	RT	Area	% Area	Height
1	8.692	20960862	100.00	3100579

7c

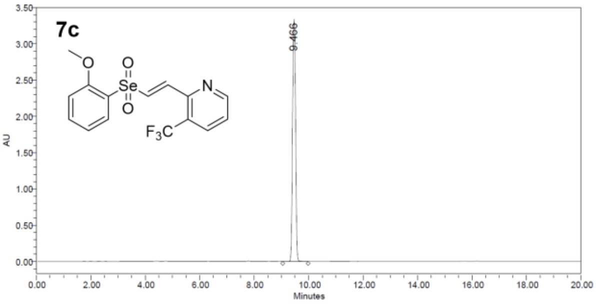

	RT	Area	% Area	Height
1	9.466	25579012	100.00	3342530

7d

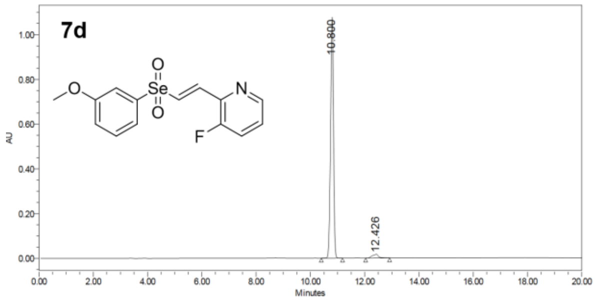

	RT	Area	% Area	Height
1	10.800	8050271	96.83	1073302
2	12.426	263628	3.17	16963

7e

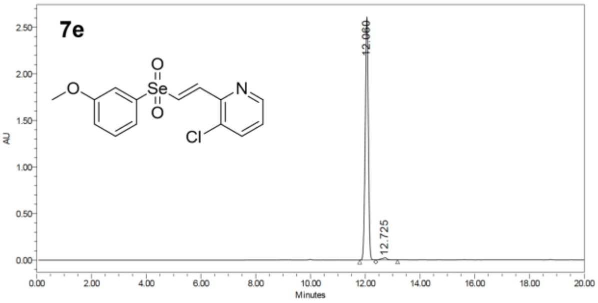

	RT	Area	% Area	Height
1	12.060	20363892	98.46	2622656
2	12.725	318293	1.54	23096

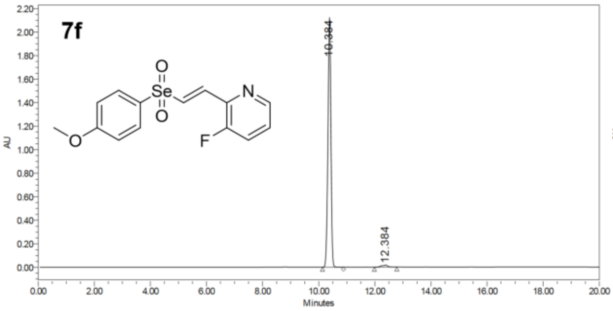

	RT	Area	% Area	Height
1	10.384	15598226	98.35	2113716
2	12.384	261669	1.65	16810

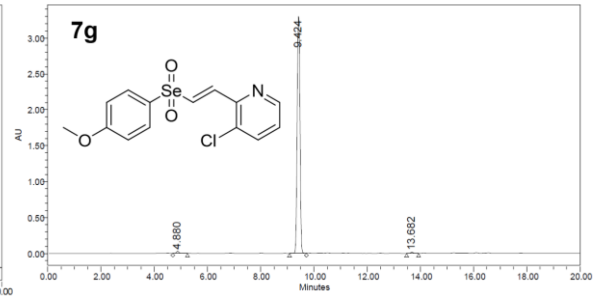

	RT	Area	% Area	Height
1	4.880	202691	0.82	29783
2	9.424	24278337	98.70	3327736
3	13.682	117193	0.48	16283

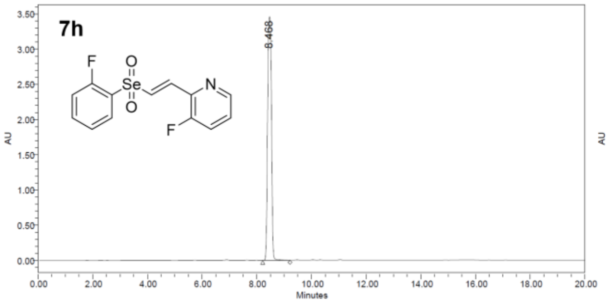

	RT	Area	% Area	Height
1	8.468	33824006	100.00	3465440

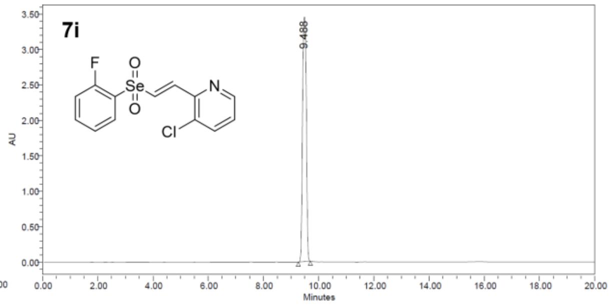

	RT	Area	% Area	Height
1	9.488	32886281	100.00	3464112

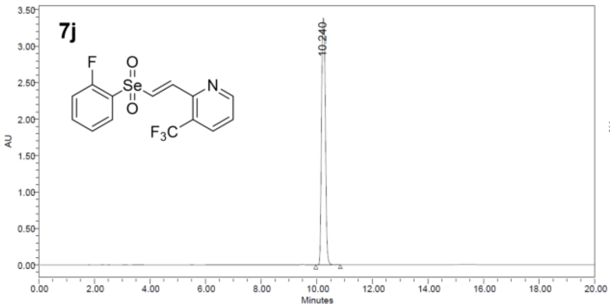

	RT	Area	% Area	Height
1	10.240	32829026	100.00	3400927

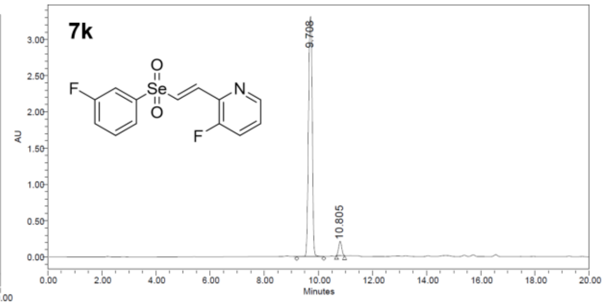

	RT	Area	% Area	Height
1	9.708	31710683	95.31	3335461
2	10.805	1558905	4.69	198301

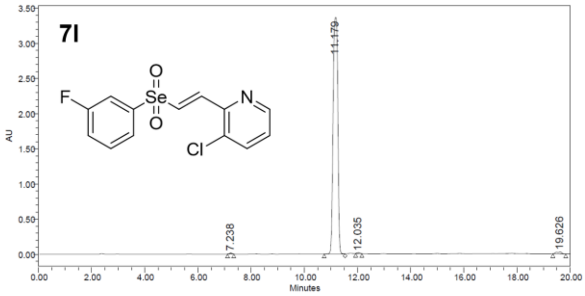

	RT	Area	% Area	Height
1	7.238	107883	0.26	17251
2	11.179	41497933	98.57	3373074
3	12.035	96593	0.23	12505
4	19.626	396918	0.94	28542

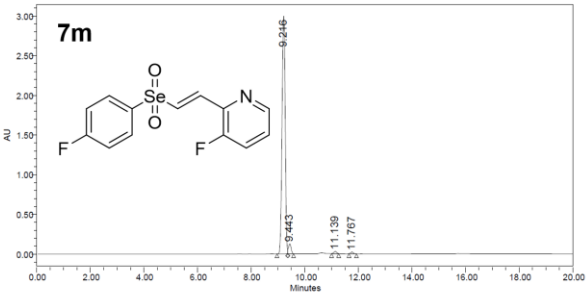

	RT	Area	% Area	Height
1	9.216	24542368	95.27	2996380
2	9.443	861971	3.35	126112
3	11.139	206584	0.80	27743
4	11.767	149766	0.58	21423

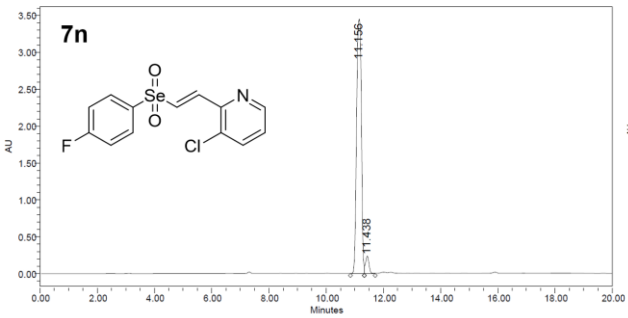

	RT	Area	% Area	Height
1	11.156	39833888	95.22	3454982
2	11.438	2000406	4.78	233981

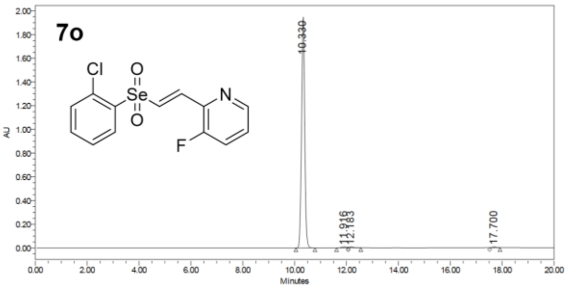

	RT	Area	% Area	Height
1	10.330	16591672	98.31	1945943
2	11.916	123087	0.73	9119
3	12.183	92725	0.55	8362
4	17.700	69003	0.41	8377

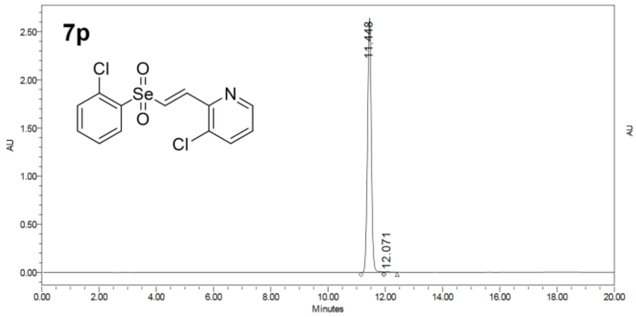

	RT	Area	% Area	Height
1	11.448	24446156	99.64	2641823
2	12.071	87235	0.36	8000

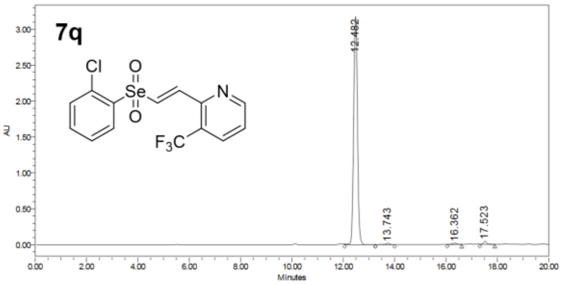

	RT	Area	% Area	Height
1	12.482	33106359	97.52	3180175
2	13.743	215893	0.64	18376
3	16.362	228285	0.67	17042
4	17.523	398018	1.17	42770

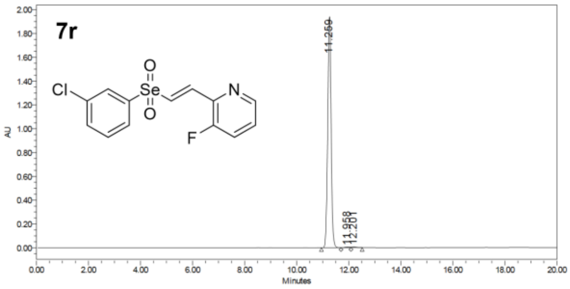

	RT	Area	% Area	Height
1	11.259	17579677	99.28	1952130
2	11.958	61165	0.35	4449
3	12.201	66044	0.37	6053

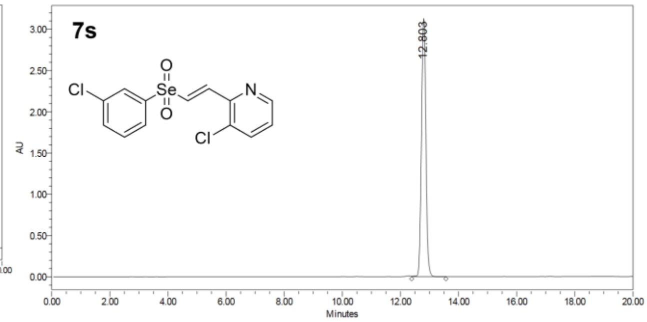

	RT	Area	% Area	Height
1	12.803	33230890	100.00	3134439

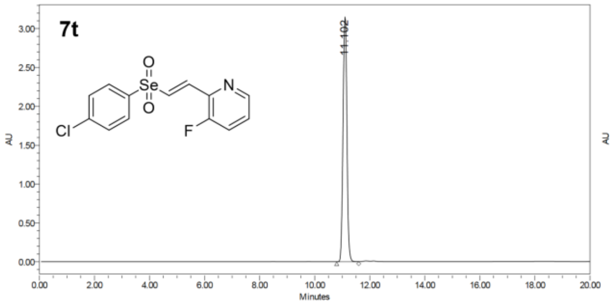

	RT	Area	% Area	Height
1	11.102	30966152	100.00	3151628

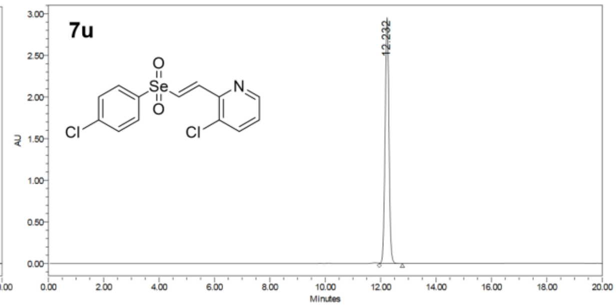

	RT	Area	% Area	Height
1	12.232	28415767	100.00	2952698

5. HRMS Analysis for the Final Compounds

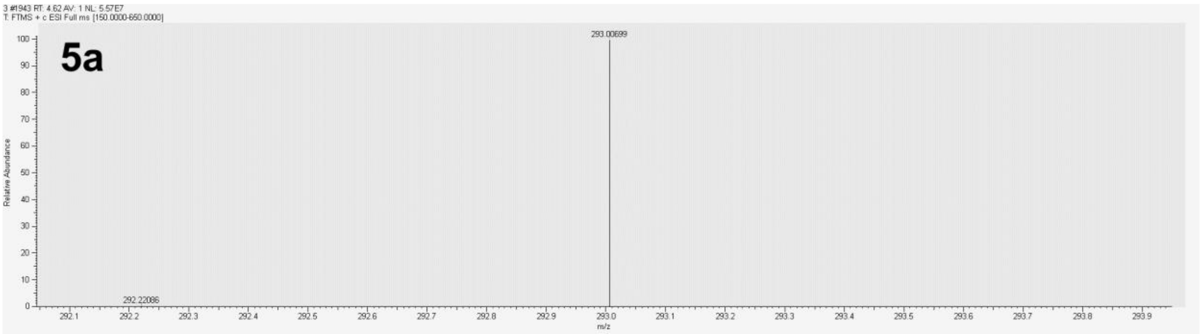

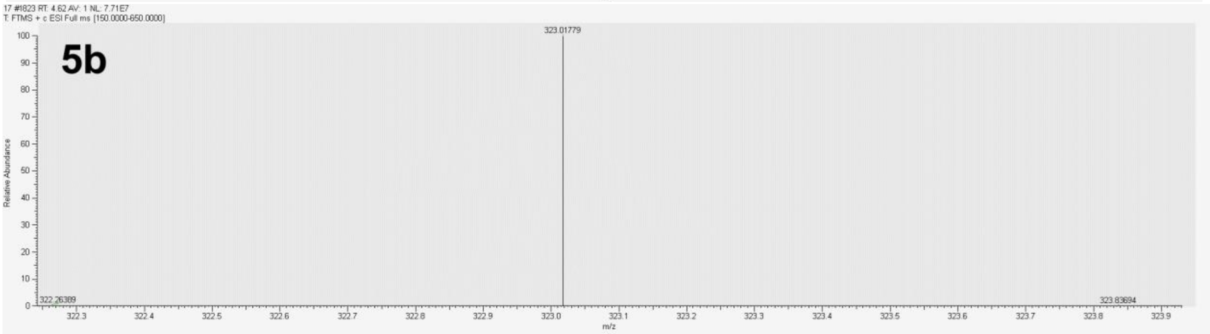

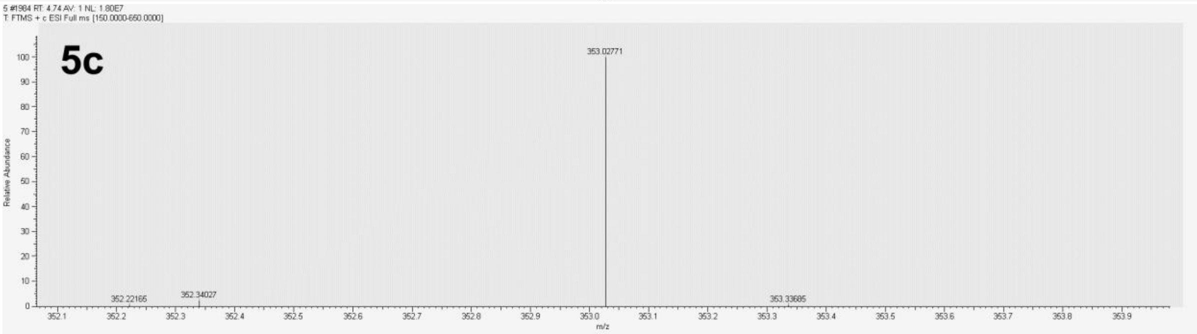

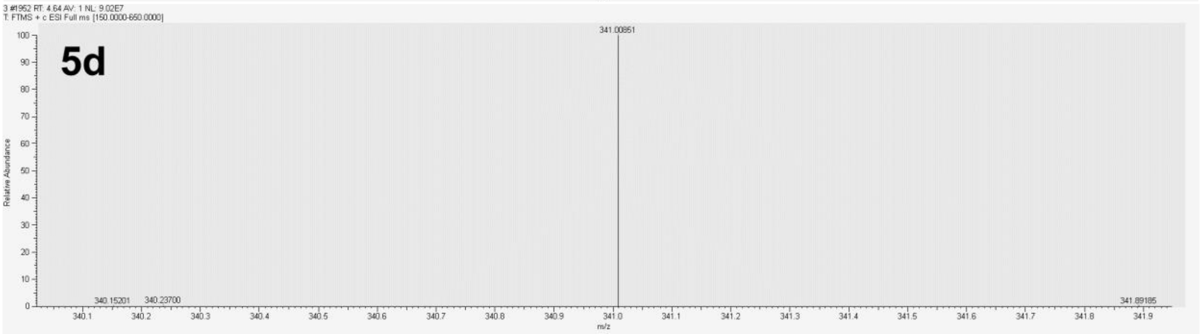

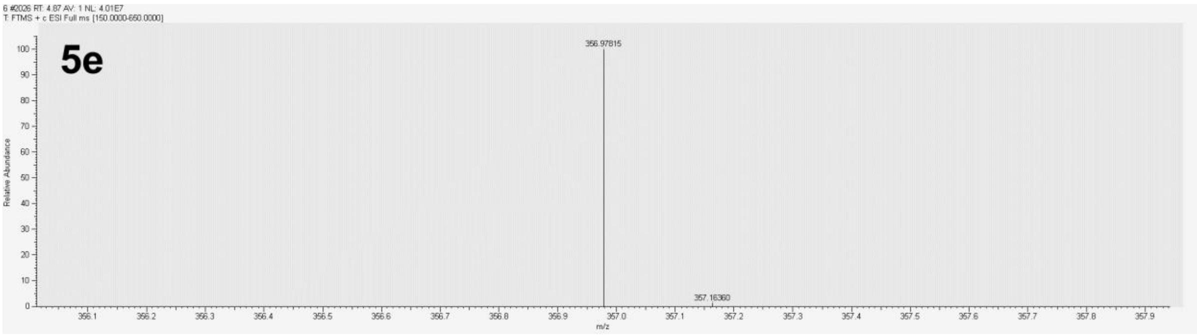
6 #2026 RT: 4.87 AV: 1 NL: 4.01E7
T: FTMS + c ESI Full ms [150.0000-650.0000]
5e
356.97815
357.16360
Relative Abundance
m/z

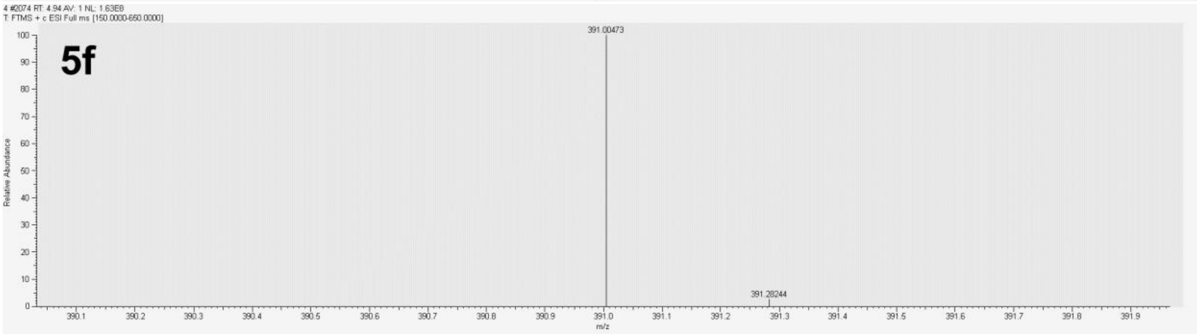
4 #2074 RT: 4.94 AV: 1 NL: 1.63E8
T: FTMS + c ESI Full ms [150.0000-650.0000]
5f
391.00473
391.28244
Relative Abundance
m/z

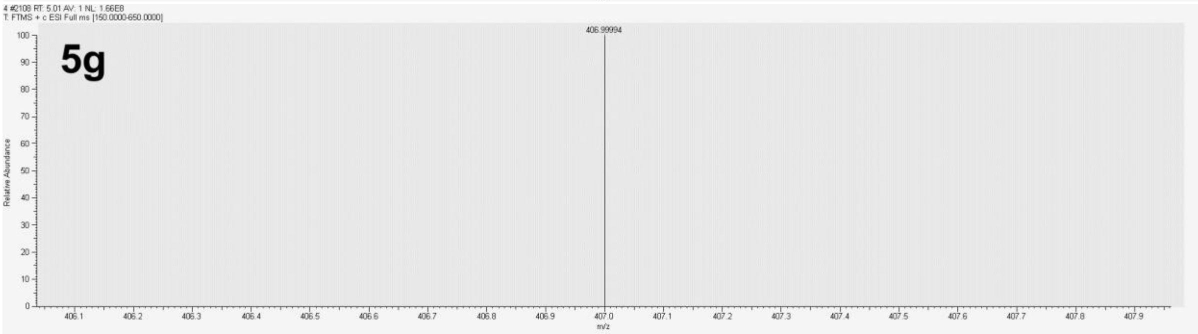
4 #2108 RT: 5.01 AV: 1 NL: 1.66E8
T: FTMS + c ESI Full ms [150.0000-650.0000]
5g
406.99994
Relative Abundance
m/z

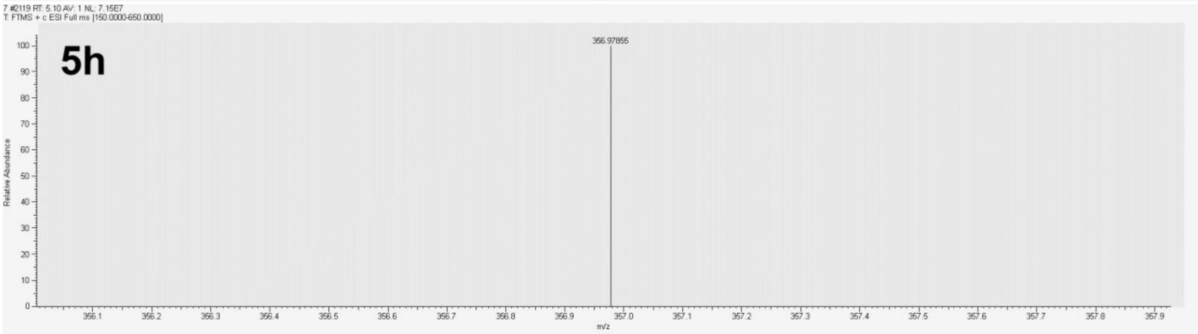
7 #2119 RT: 5.10 AV: 1 NL: 7.15E7
T: FTMS + c ESI Full ms [150.0000-650.0000]
5h
356.97855
Relative Abundance
m/z

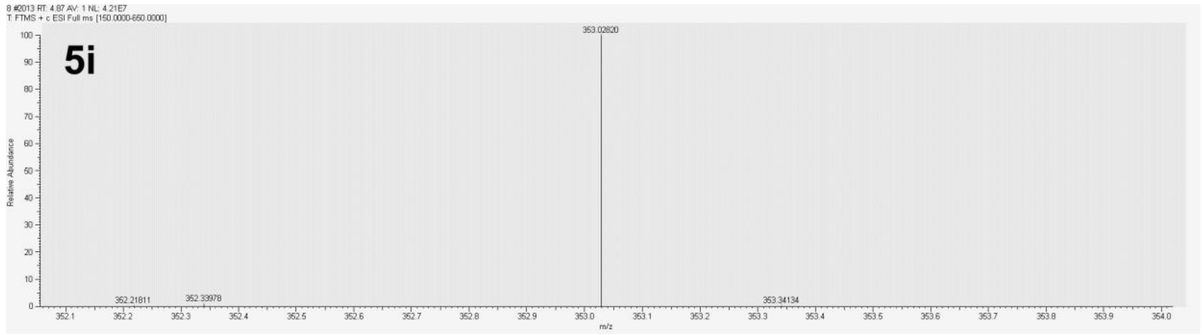
8 #2013 RT: 4.87 AV: 1 NL: 4.21E7
T: FTMS + c ESI Full ms [150.0000-650.0000]
5i
353.02820
352.21811
352.33978
353.34134
Relative Abundance
m/z

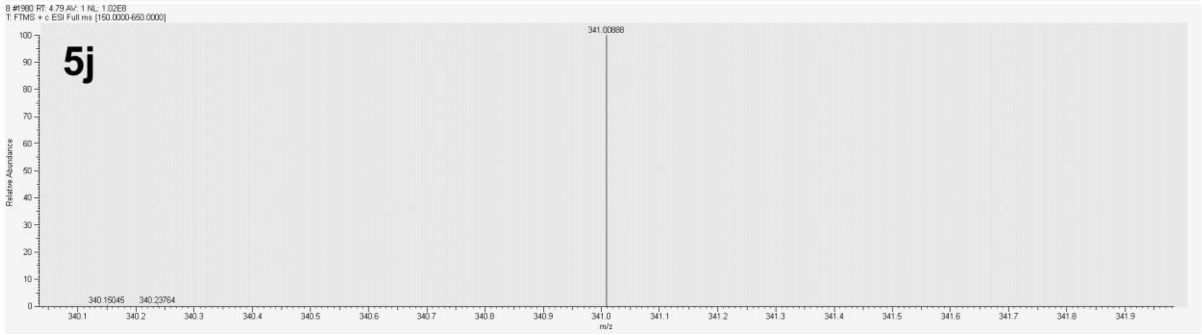
8 #1980 RT: 4.79 AV: 1 NL: 1.02E8
T: FTMS + c ESI Full ms [150.0000-650.0000]
5j
341.00888
340.15045
340.23764
Relative Abundance
m/z

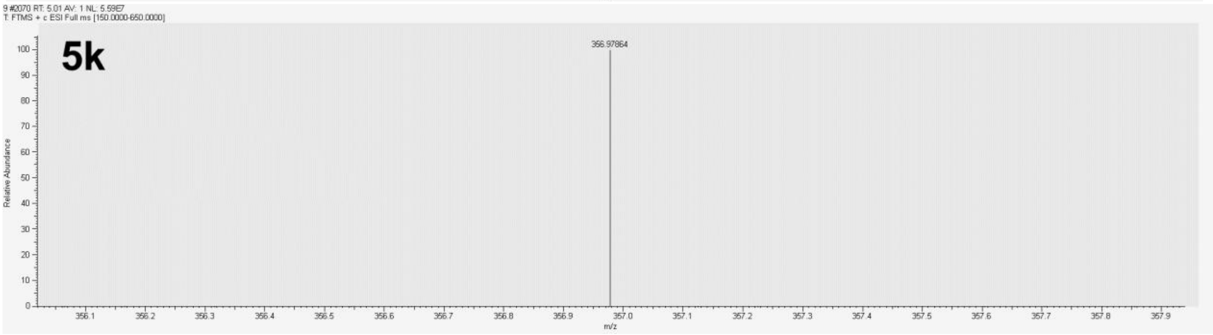
9 #2070 RT: 5.01 AV: 1 NL: 5.59E7
T: FTMS + c ESI Full ms [150.0000-650.0000]
5k
356.97864
Relative Abundance
m/z

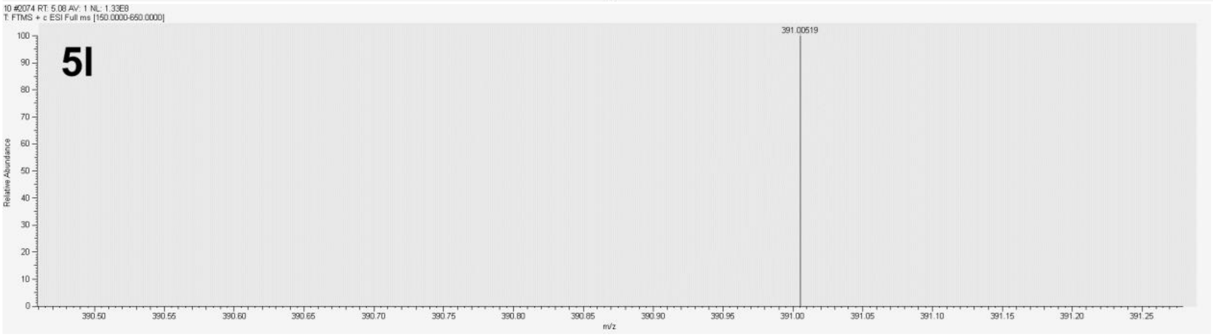
10 #2074 RT: 5.08 AV: 1 NL: 1.33E8
T: FTMS + c ESI Full ms [150.0000-650.0000]
5l
391.00519
Relative Abundance
m/z

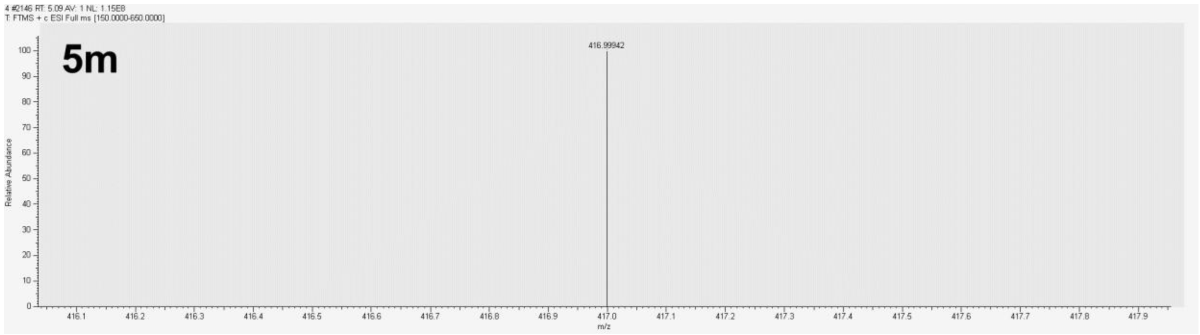
5m
416.99942
Relative Abundance
m/z

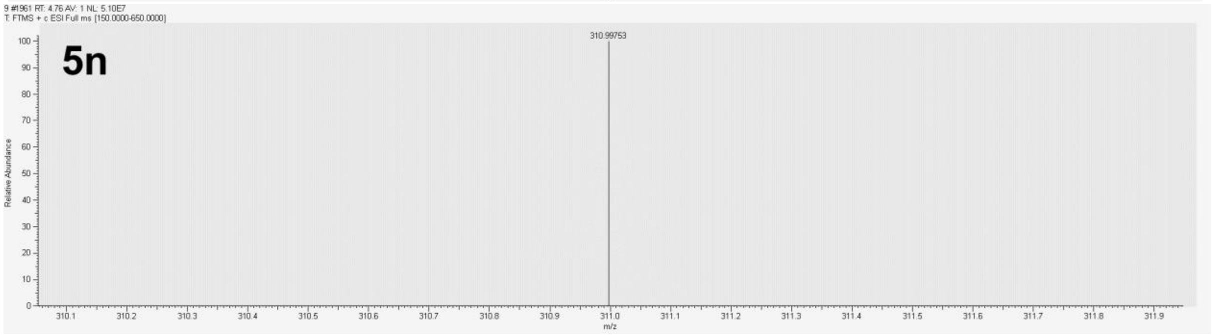
5n
310.99753
Relative Abundance
m/z

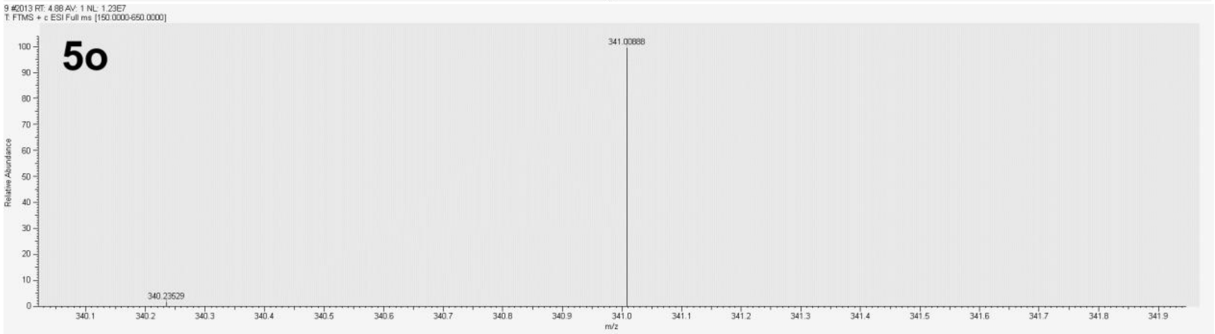
5o
341.00888
340.23529
Relative Abundance
m/z

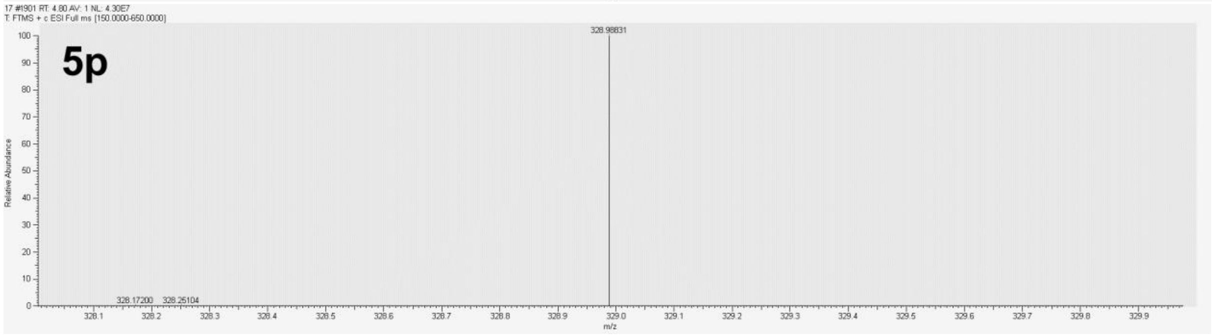
5p
328.98831
328.17200
328.25104
Relative Abundance
m/z

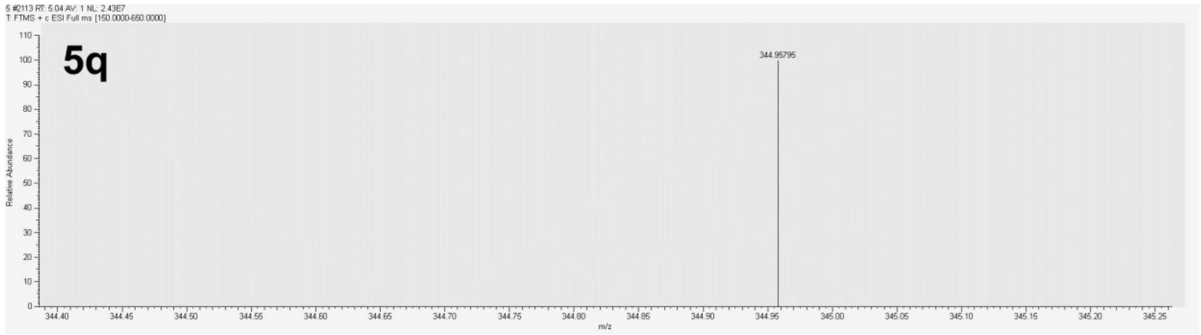
5 #2113 RT: 5.04 AV: 1 NL: 2.43E7
T: FTMS + c ESI Full ms [150.0000-650.0000]
5q
344.95795
Relative Abundance
m/z

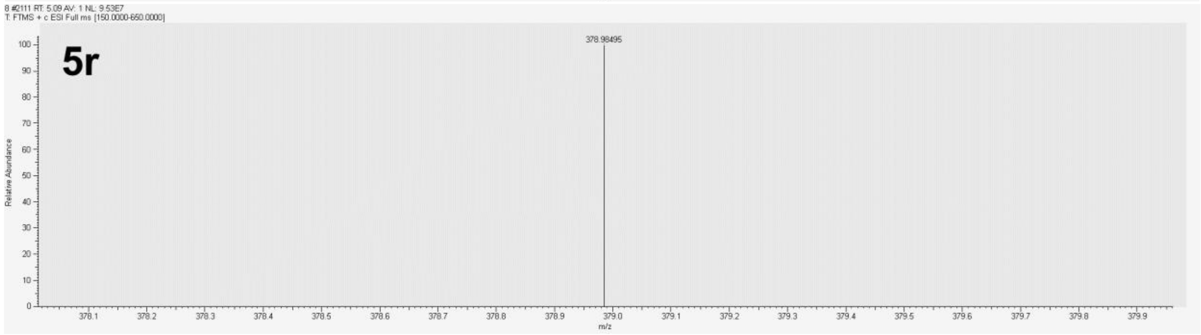
8 #2111 RT: 5.09 AV: 1 NL: 9.53E7
T: FTMS + c ESI Full ms [150.0000-650.0000]
5r
378.98495
Relative Abundance
m/z

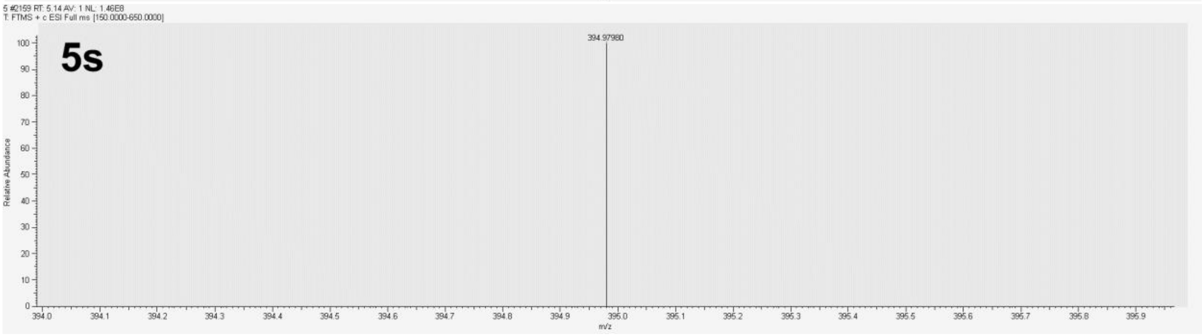
5 #2159 RT: 5.14 AV: 1 NL: 1.46E8
T: FTMS + c ESI Full ms [150.0000-650.0000]
5s
394.97980
Relative Abundance
m/z

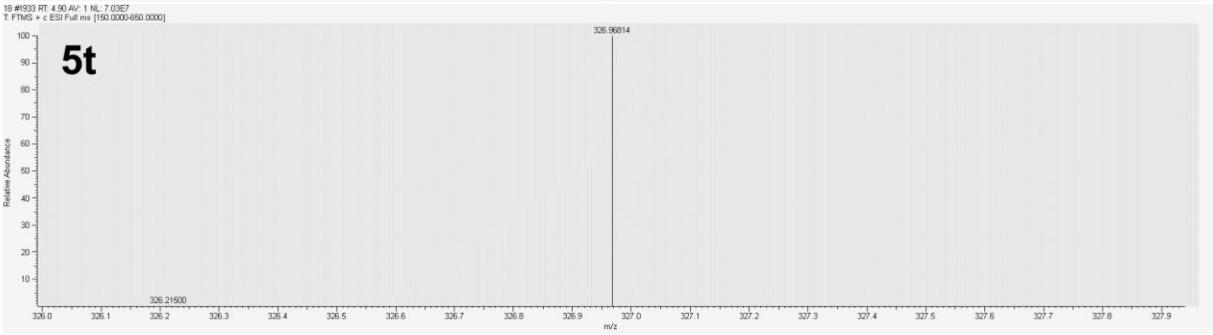
18 #1933 RT: 4.90 AV: 1 NL: 7.03E7
T: FTMS + c ESI Full ms [150.0000-650.0000]
5t
326.96814
326.21500
Relative Abundance
m/z

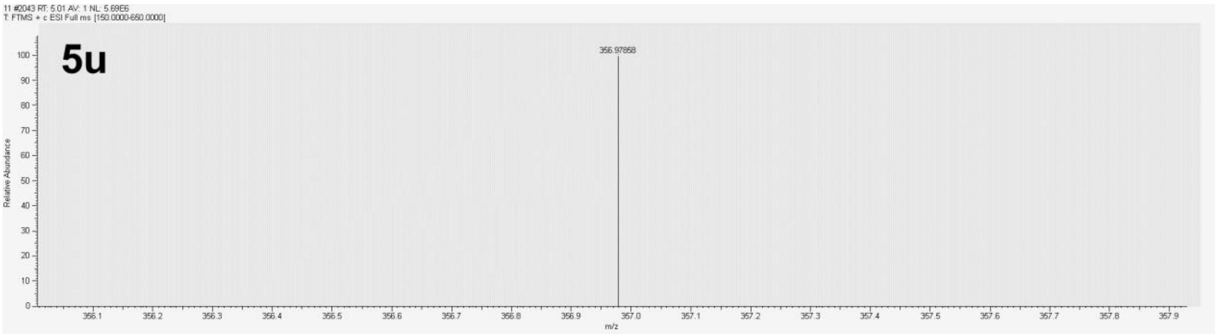
11 #2043 RT: 5.01 AV: 1 NL: 5.69E6
T: FTMS + c ESI Full ms [150.0000-650.0000]
5u
356.97858
Relative Abundance
m/z

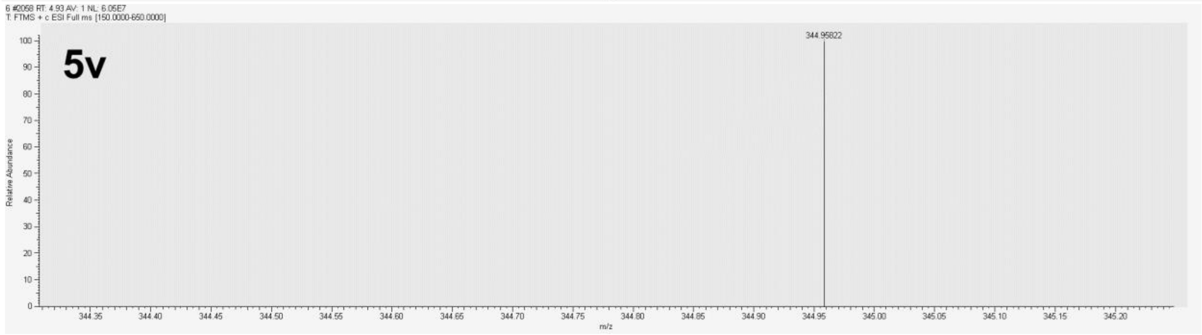
6 #2058 RT: 4.93 AV: 1 NL: 6.05E7
T: FTMS + c ESI Full ms [150.0000-650.0000]
5v
344.95822
Relative Abundance
m/z

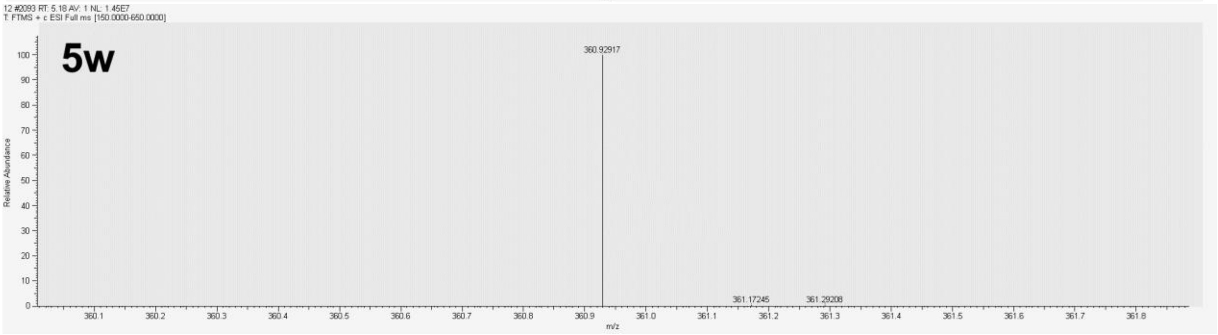
12 #2093 RT: 5.18 AV: 1 NL: 1.45E7
T: FTMS + c ESI Full ms [150.0000-650.0000]
5w
360.92917
361.17245
361.29208
Relative Abundance
m/z

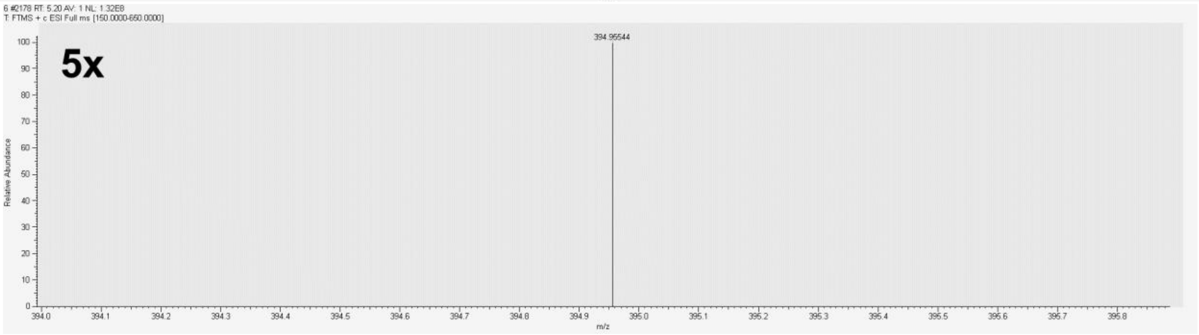
6 #2178 RT: 5.20 AV: 1 NL: 1.32E8
T: FTMS + c ESI Full ms [150.0000-650.0000]
5x
394.95544
Relative Abundance
m/z

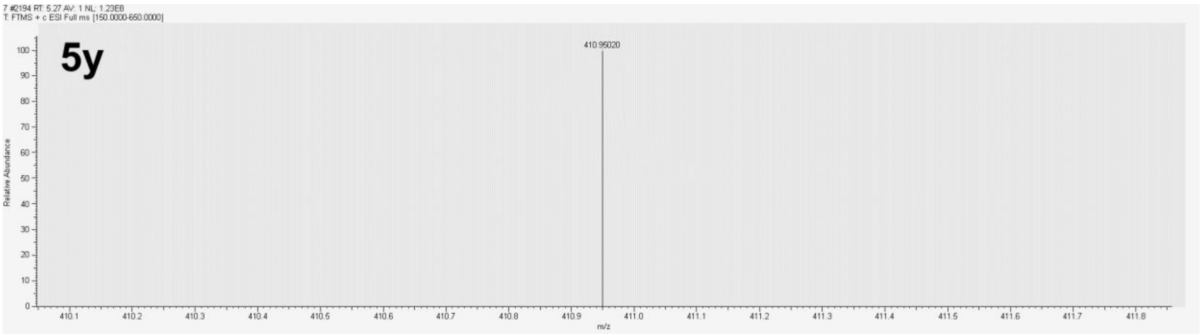
7 #2194 RT: 5.27 AV: 1 NL: 1.23E8
T: FTMS + c ESI Full ms [150.0000-650.0000]
5y
410.95020
Relative Abundance
m/z

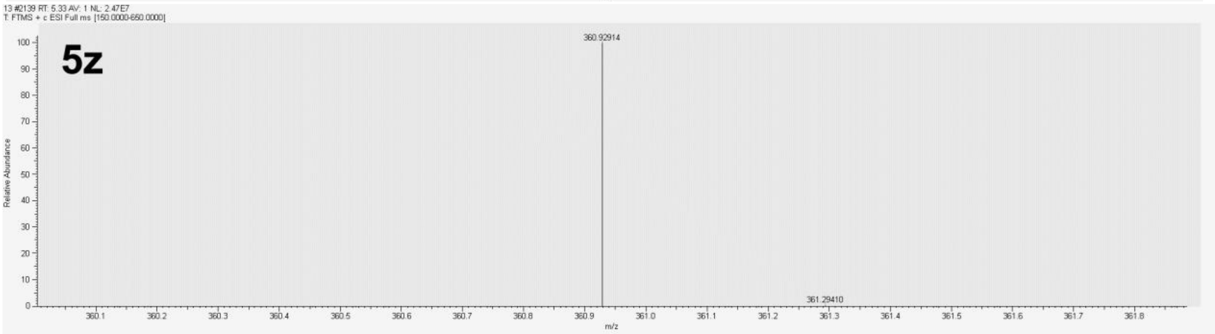
13 #2139 RT: 5.33 AV: 1 NL: 2.47E7
T: FTMS + c ESI Full ms [150.0000-650.0000]
5z
360.92914
361.29410
Relative Abundance
m/z

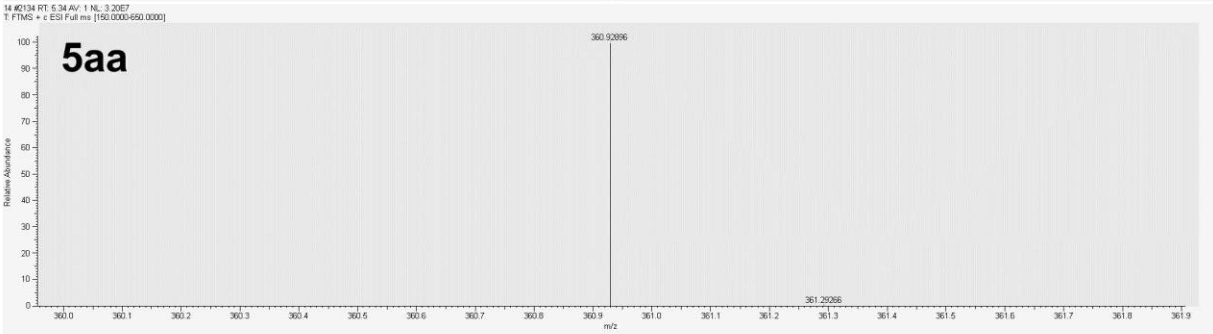
14 #2134 RT: 5.34 AV: 1 NL: 3.20E7
T: FTMS + c ESI Full ms [150.0000-650.0000]
5aa
360.92896
361.29266
Relative Abundance
m/z

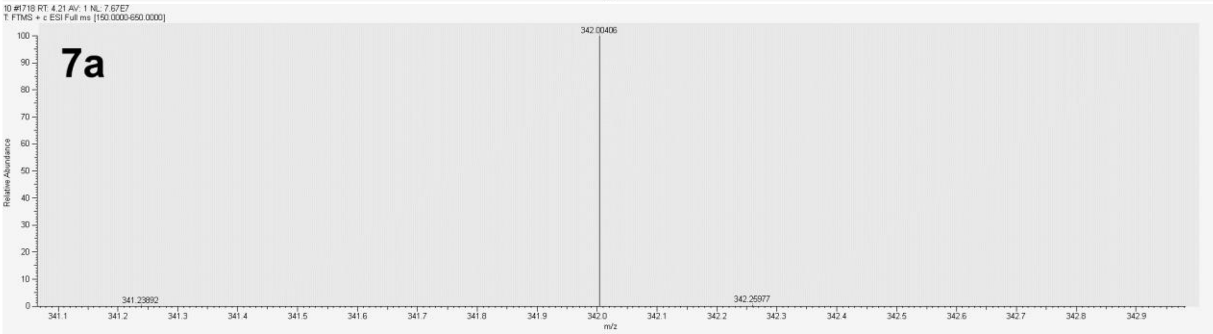
10 #1718 RT: 4.21 AV: 1 NL: 7.67E7
T: FTMS + c ESI Full ms [150.0000-650.0000]
7a
342.00406
341.23892
342.25977
Relative Abundance
m/z

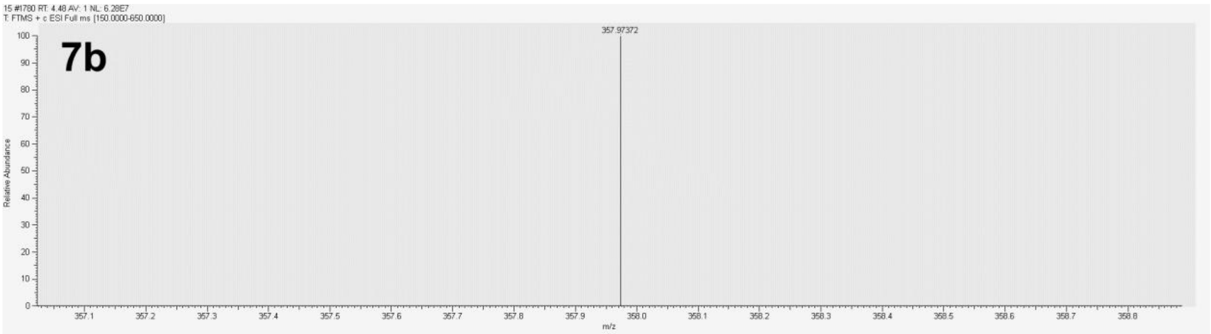
15 #1780 RT: 4.48 AV: 1 NL: 6.28E7
T: FTMS + c ESI Full ms [150.0000-650.0000]
7b
357.97372
Relative Abundance
m/z

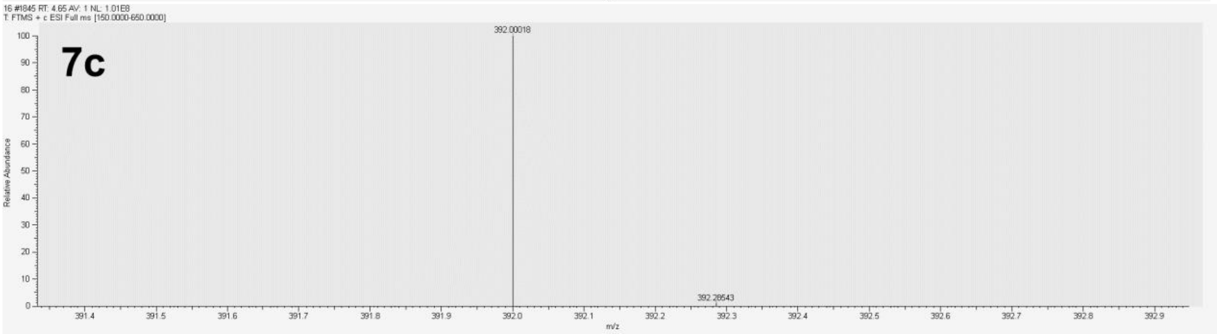
16 #1845 RT: 4.65 AV: 1 NL: 1.01E8
T: FTMS + c ESI Full ms [150.0000-650.0000]
7c
392.00018
392.28543
Relative Abundance
m/z

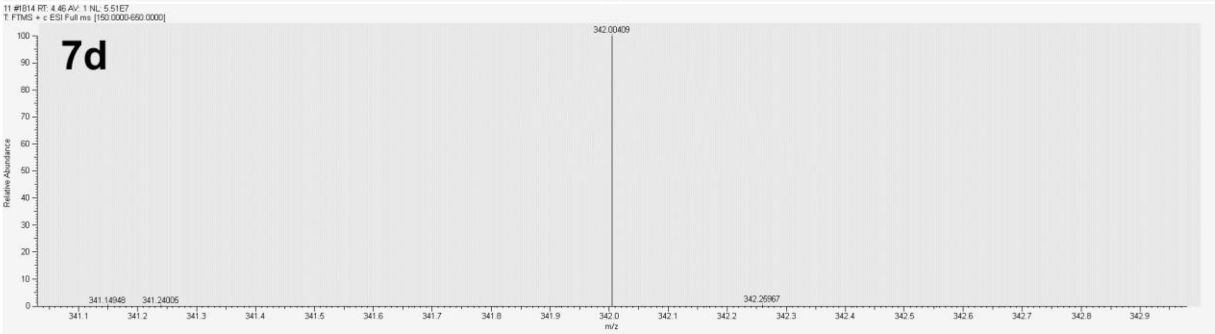
11 #1814 RT: 4.46 AV: 1 NL: 5.51E7
T: FTMS + c ESI Full ms [150.0000-650.0000]
7d
342.00409
341.14948
341.24005
342.25967
Relative Abundance
m/z

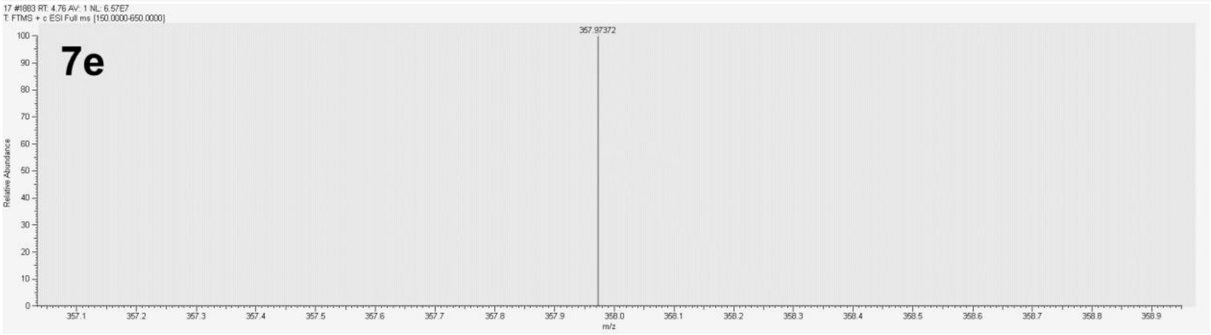
17 #1883 RT: 4.76 AV: 1 NL: 6.57E7
T: FTMS + c ESI Full ms [150.0000-650.0000]
7e
357.97372
Relative Abundance
m/z

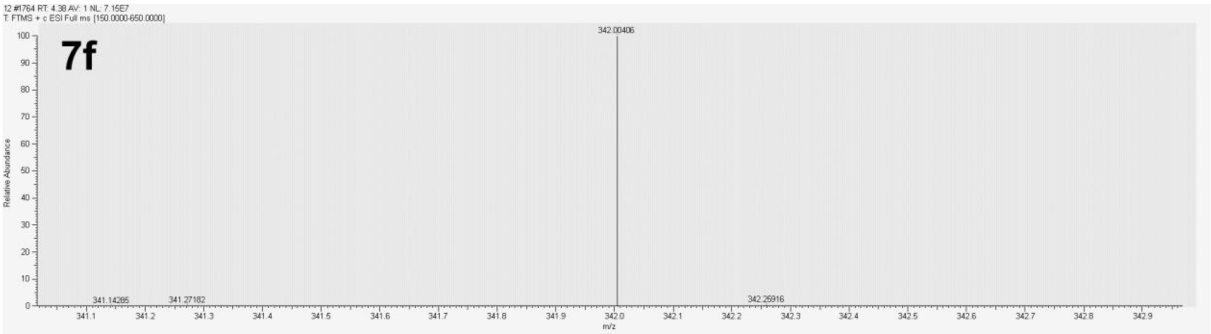
12 #1764 RT: 4.38 AV: 1 NL: 7.15E7
T: FTMS + c ESI Full ms [150.0000-650.0000]
7f
342.00406
341.14285
341.27182
342.25916
Relative Abundance
m/z

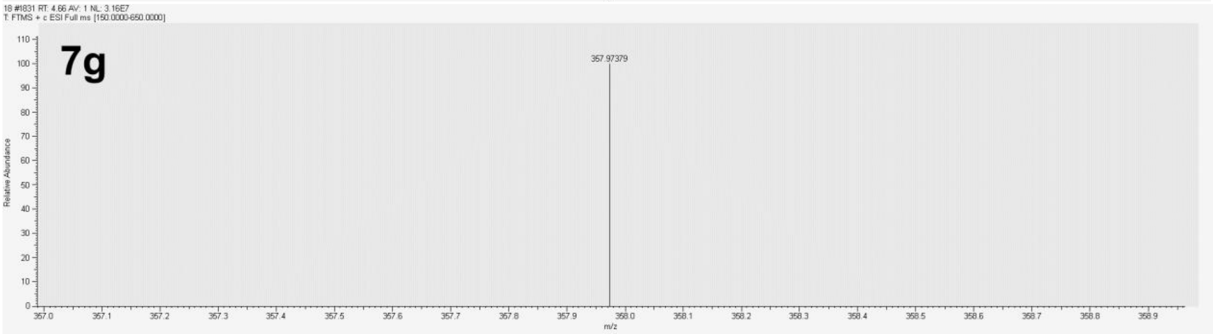
18 #1831 RT: 4.66 AV: 1 NL: 3.16E7
T: FTMS + c ESI Full ms [150.0000-650.0000]
7g
357.97379
Relative Abundance
m/z

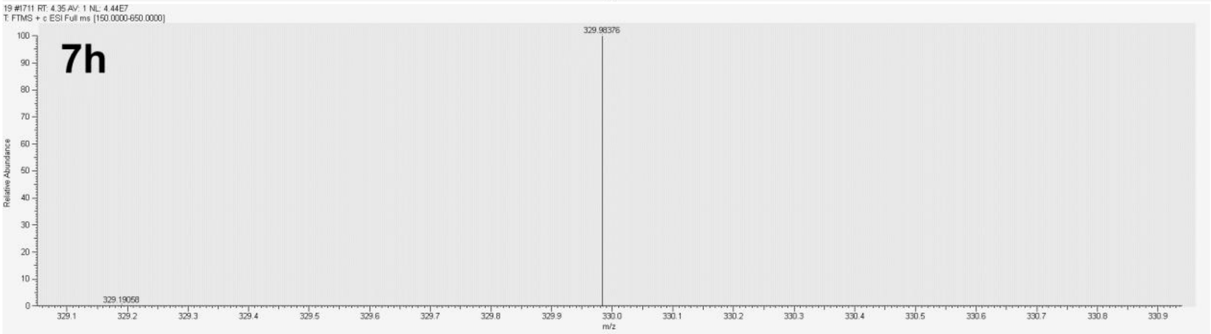
19 #1711 RT: 4.35 AV: 1 NL: 4.44E7
T: FTMS + c ESI Full ms [150.0000-650.0000]
7h
329.98376
329.19058
Relative Abundance
m/z

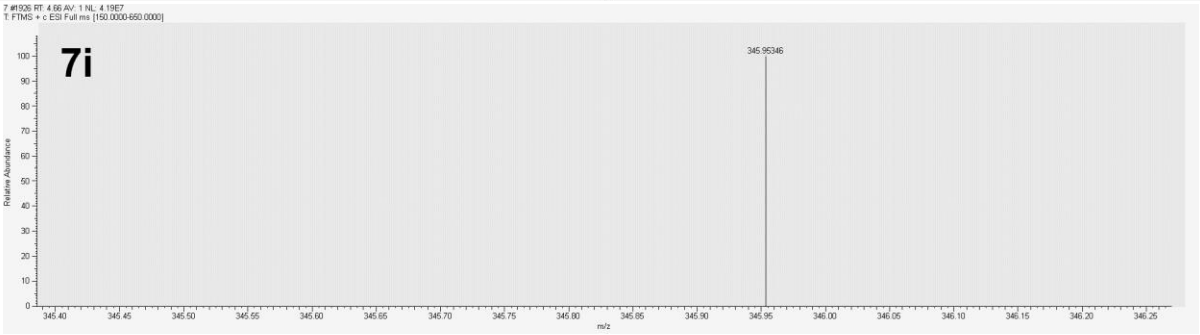
7 #1926 RT: 4.66 AV: 1 NL: 4.19E7
T: FTMS + c ESI Full ms [150.0000-650.0000]
7i
345.95346
Relative Abundance
m/z

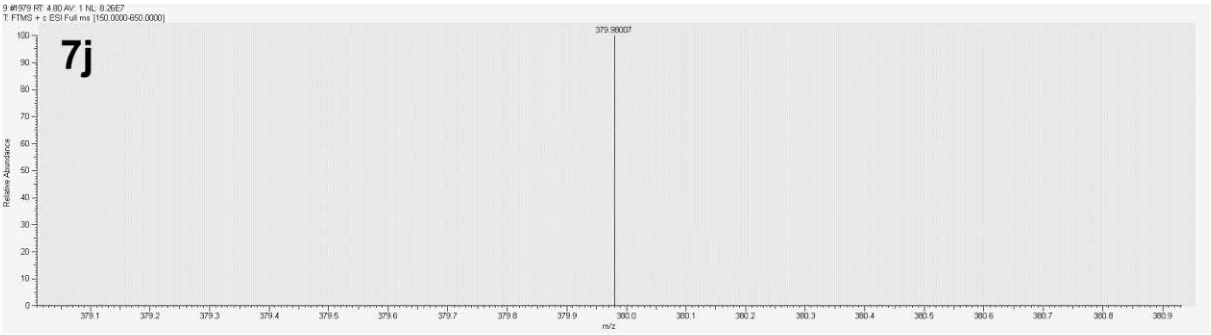
9 #1979 RT: 4.80 AV: 1 NL: 8.26E7
T: FTMS + c ESI Full ms [150.0000-650.0000]
7j
379.98007
Relative Abundance
m/z

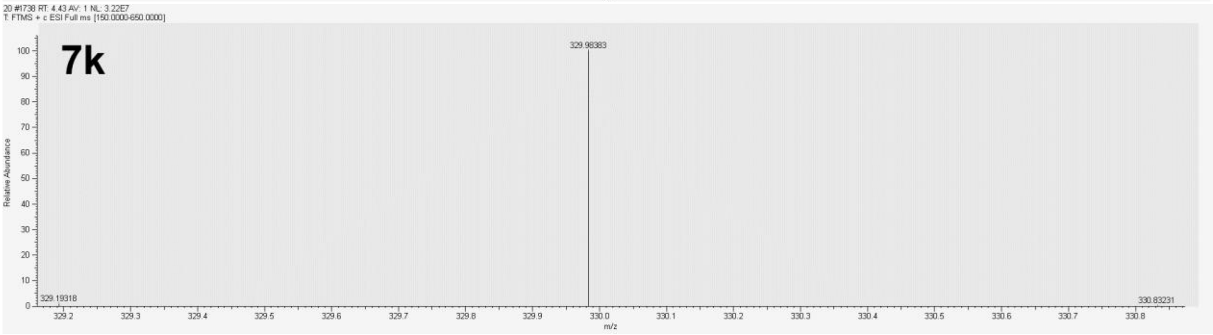
20 #1738 RT: 4.43 AV: 1 NL: 3.22E7
T: FTMS + c ESI Full ms [150.0000-650.0000]
7k
329.98383
329.19318
330.83231
Relative Abundance
m/z

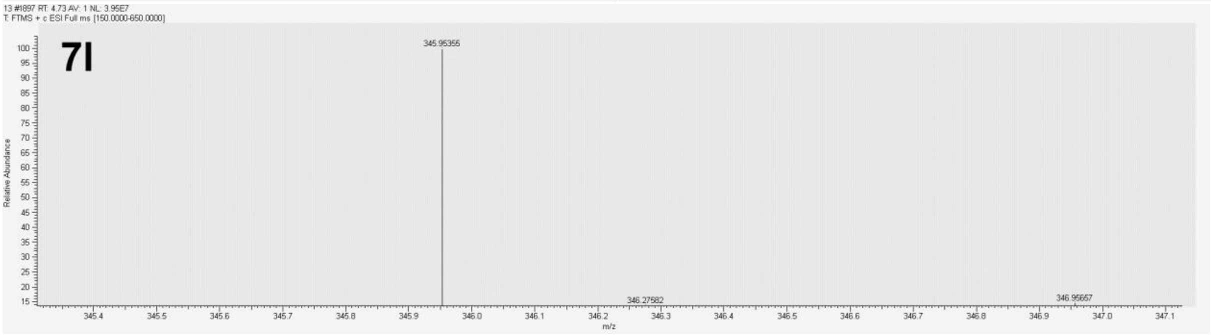
13 #1897 RT: 4.73 AV: 1 NL: 3.95E7
T: FTMS + c ESI Full ms [150.0000-650.0000]
7l
345.95355
346.27582
346.95657
Relative Abundance
m/z

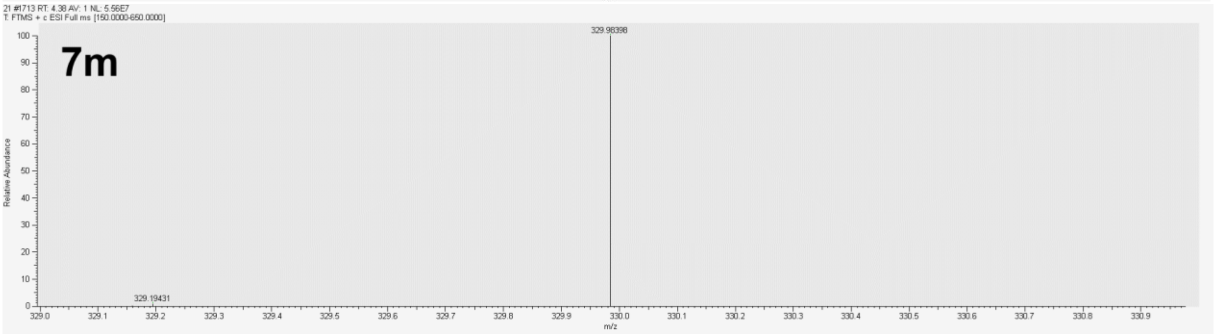
21 #1713 RT: 4.38 AV: 1 NL: 5.56E7
T: FTMS + c ESI Full ms [150.0000-650.0000]
7m
329.98398
329.19431
Relative Abundance
m/z

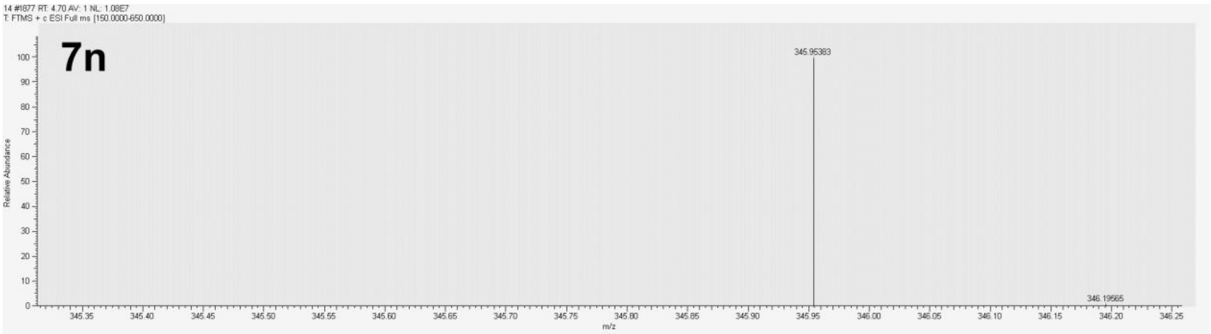
14 #1877 RT: 4.70 AV: 1 NL: 1.08E7
T: FTMS + c ESI Full ms [150.0000-650.0000]
7n
345.95383
346.19565
Relative Abundance
m/z

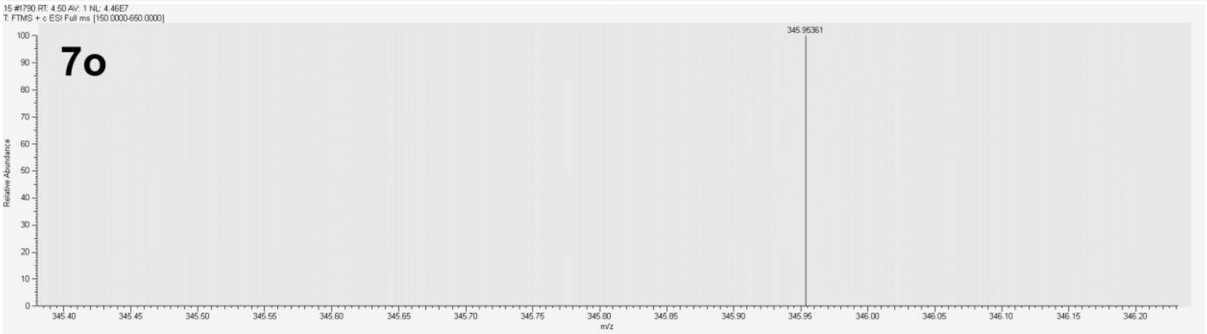
15 #1790 RT: 4.50 AV: 1 NL: 4.46E7
T: FTMS + c ESI Full ms [150.0000-650.0000]
7o
345.95361
Relative Abundance
m/z

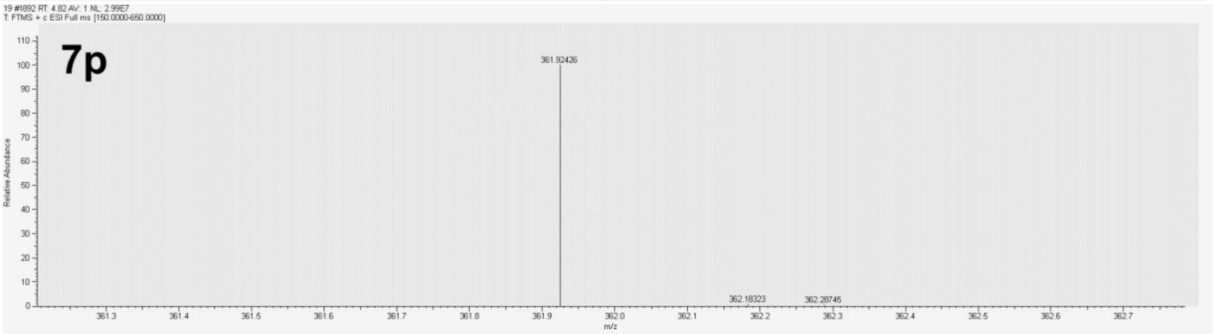
19 #1892 RT: 4.82 AV: 1 NL: 2.99E7
T: FTMS + c ESI Full ms [150.0000-650.0000]
7p
361.92426
362.18323
362.28745
Relative Abundance
m/z

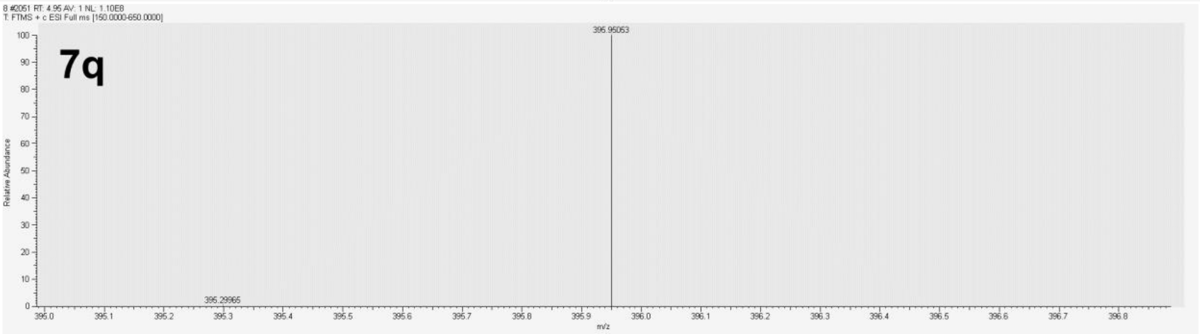
8 #2051 RT: 4.95 AV: 1 NL: 1.10E8
T: FTMS + c ESI Full ms [150.0000-650.0000]
7q
395.95063
395.29965
Relative Abundance
m/z

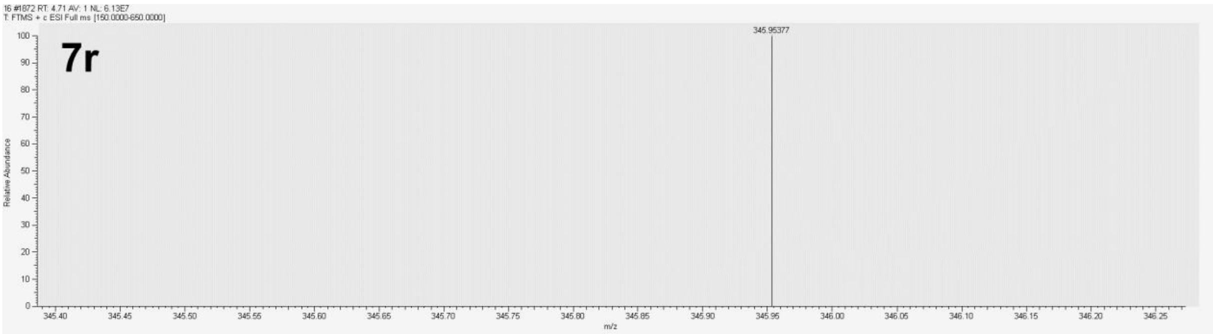
16 #1872 RT: 4.71 AV: 1 NL: 6.13E7
T: FTMS + c ESI Full ms [150.0000-650.0000]
7r
345.95377
Relative Abundance
m/z

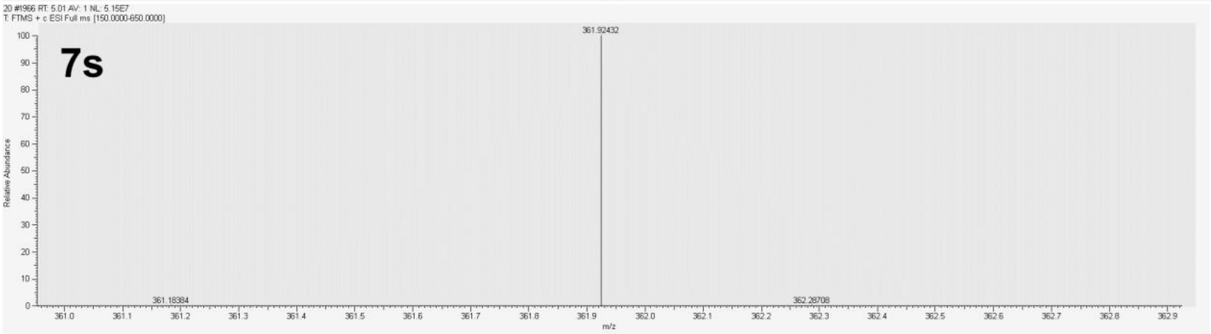
20 #1966 RT: 5.01 AV: 1 NL: 5.15E7
T: FTMS + c ESI Full ms [150.0000-650.0000]
7s
361.92432
361.18384
362.28708
Relative Abundance
m/z

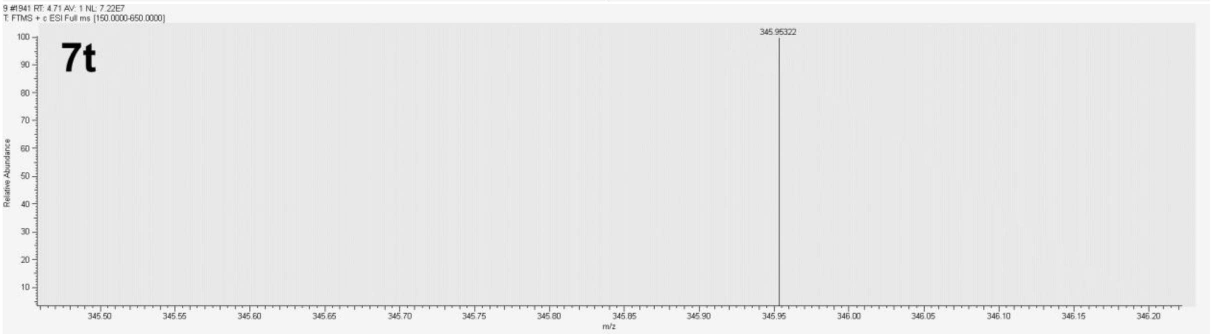
9 #1941 RT: 4.71 AV: 1 NL: 7.22E7
T: FTMS + c ESI Full ms [150.0000-650.0000]
7t
345.95322
Relative Abundance
m/z

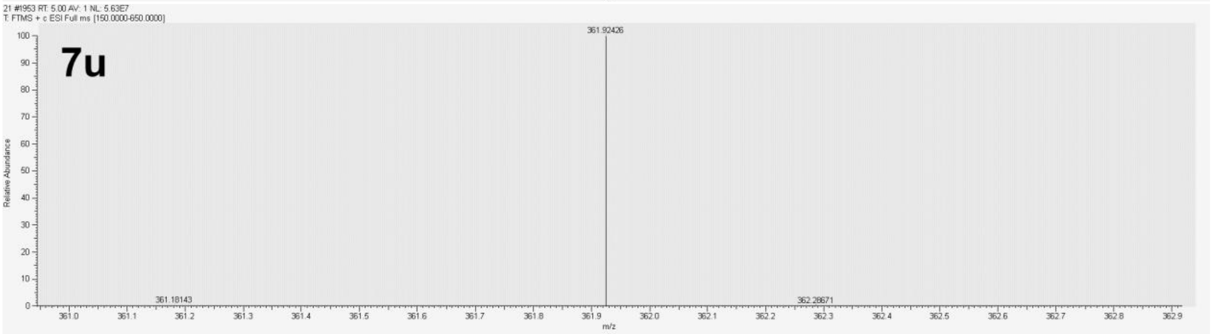
21 #1953 RT: 5.00 AV: 1 NL: 5.63E7
T: FTMS + c ESI Full ms [150.0000-650.0000]
7u
361.92426
361.18143
362.28671
Relative Abundance
m/z
